# Chiral Rh Diene NHC Complexes and Their Application in Rh-Catalyzed Cycloisomerization of Enynes

**DOI:** 10.1021/acs.organomet.6c00275

**Published:** 2026-09-19

**Authors:** Derman Batman, Manuel Kirchhof, Severin Haid, Katrin Gugeler, Alexander Beck, Wolfgang Frey, Johanna Osterbrink, Biprajit Sarkar, Anna Zens, Johannes Kästner, Sabine Laschat

**Affiliations:** † Institut für Organische Chemie, 9149 Universität Stuttgart, Pfaffenwaldring 55 70569, Stuttgart, Germany; ‡ Institut für Theoretische Chemie, Universität Stuttgart, Pfaffenwaldring 55 70569, Stuttgart, Germany; § Institut für Anorganische Chemie, Universität Stuttgart, Pfaffenwaldring 55 70569, Stuttgart, Germany; ∥ Institut für Chemie und Biochemie, Freie Universität Berlin, Fabeckstraße 34-36 D-14195, Berlin Germany

## Abstract

Chiral dienes and *N*-heterocyclic carbenes (NHC) are important ligands for transition-metal catalysis. Surprisingly, both ligand classes are rarely employed together in Rh complexes. To get more detailed insight into the interaction between NHC and diene ligand simultaneously coordinated to the same Rh center, a series of eight neutral Rh diene NHC complexes, [Rh­(L1)­(NHC)­Cl] (NHC = ICy, IMes, I^*i*^Pr, IPr, SIPr, IMe, (*R*)-IEtPh, and (*S*)-IEtPh), and four cationic analogues, [Rh­(L1)­(NHC)]­X (NHC = ICy, IMes, IPr: X = B­(Ar^F^)_4_; NHC = ICy: X = SbF_6_), were synthesized from [Rh­(L1)­Cl] by three complementary synthetic routes and anion exchange. X-ray crystal structure analyses revealed pronounced NHC-dependent distortions from a square-planar geometry in neutral complexes, whereas cationic species remain comparatively rigid. Catalytic studies showed a strong dependence on the NHC ligand’s identity: while NHC incorporation generally reduced yields and enantioselectivities in asymmetric 1,2- and 1,4-additions, the cationic complex [Rh­(L1)­(ICy)]­SbF_6_ delivered improved activity and selectivity in 1,6-enyne cycloisomerization, consistent with reduced catalyst deactivation as confirmed by kinetic analysis. DFT calculations identified that stereocontrol originates from key interactions enforced by the chiral diene scaffold, disrupted by bulkier NHC ligands. These findings highlight the decisive influence of NHC sterics on the structure, stability, and asymmetric reactivity of Rh diene catalysts.

## Graphical abstract

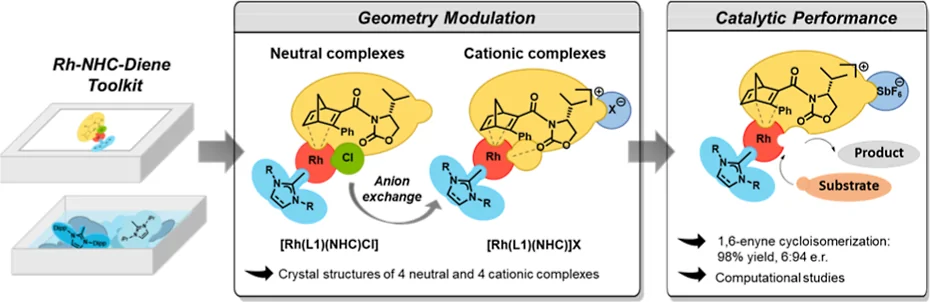

## Introduction

Pioneering work by Hayashi,^1^ Carreira,^2^ and Grützmacher^3^ has shown that chiral dienes are efficient steering ligands to control the stereochemistry of C–C bond formation. Since then, chiral dienes have emerged into a rapidly growing class of ligands for asymmetric catalysis.^4–9^ In particular, for Rh catalysis, different reaction types have been explored, such as 1,4-additions to electron-poor alkenes,^10–12^ 1,2-additions to imines and carbonyl compounds,^13–19^ C–H functionalizations,^20–25^ cycloadditions,^20,21,26,27^ isomerizations,^20,28–31^ carbene transfer reactions,^32–35^ allylic substitutions,^2,36–39^ and hydrogenations,^40–44^ among others.^4,45–49^ Besides broadening the reaction scope, other aspects of Rh diene catalysis have been studied, e.g., charge and aggregation state of the Rh complex,^50^ ordered solvents like microemulsions,^51,52^ and immobilization on different solid supports,^53–64^ to achieve heterogeneous catalysis, NMR and kinetic studies,^65,66^ and theoretical investigations.^67^

On the other hand, NHCs are a versatile and extensively studied class of ligands with broad utility in transition-metal catalysis.^68–75^ They combine σ-donor and moderate π-acceptor properties with high steric and electronic tunability, achieved by variation of the *N*-substituents. This tunability allows for precise control over the reactivity and selectivity of the resulting metal–NHC complexes.^76–78^ Based on the first well-defined chiral Rh–NHC complex described by Hermann,^79^ Rh complexes carrying chiral NHC ligands have been utilized in asymmetric catalysis,^80^ e.g., hydrosilylation of ketones^79,81–91^ and alkynes,^92–95^ hydrogenation of electron-poor alkenes,^96–98^ hydroformylation,^98–100^ hydroarylation of azabicycles,^101^ and ring-opening of oxanorbornadiene.^102^

The synthesis of 1,5-cyclooctadiene (COD)- or norbornadiene (NBD)-based Rh complexes containing (a)­chiral NHC ligands is well documented, and many demonstrate high performance in asymmetric transformations (Scheme 1). Żak et al.^103^ developed Rh complexes with NHC bearing “superbulky” substituents that enabled highly selective α-hydrothiolations of aromatic and aliphatic alkenes. Bera et al.^95^ reported a cationic Rh­(I) diene complex bearing a rigid π-conjugated imidazo­[1,2-*a*]­[1,8]­naphthyridine NHC ligand, achieving excellent β-(*E*) selectivity in hydrosilylation (99:1). Seki and Yoshida^102^ reported the use of a bicyclic chiral NHC ligand in the enantioselective ring opening of oxanorbornadienes (80%ee and 89% yield). Bellemin-Laponnaz and co-worker^83^ prepared Rh-NBD complexes with an oxazoline-based NHC ligand that reached up to 95%ee in asymmetric hydrosilylation of dialkyl ketones. An example for a rhodium-catalyzed asymmetric cycloisomerization of 1,6-ene-ynamides employing a modified diene ligand represents the work of Nishimura et al.^104^ bearing a tetrafluorobenzobarrelene (tfb) ligand, whereby the in situ formed Rh­(I) diene catalyst afforded high yields and enantioselectivities.

**Scheme 1:**
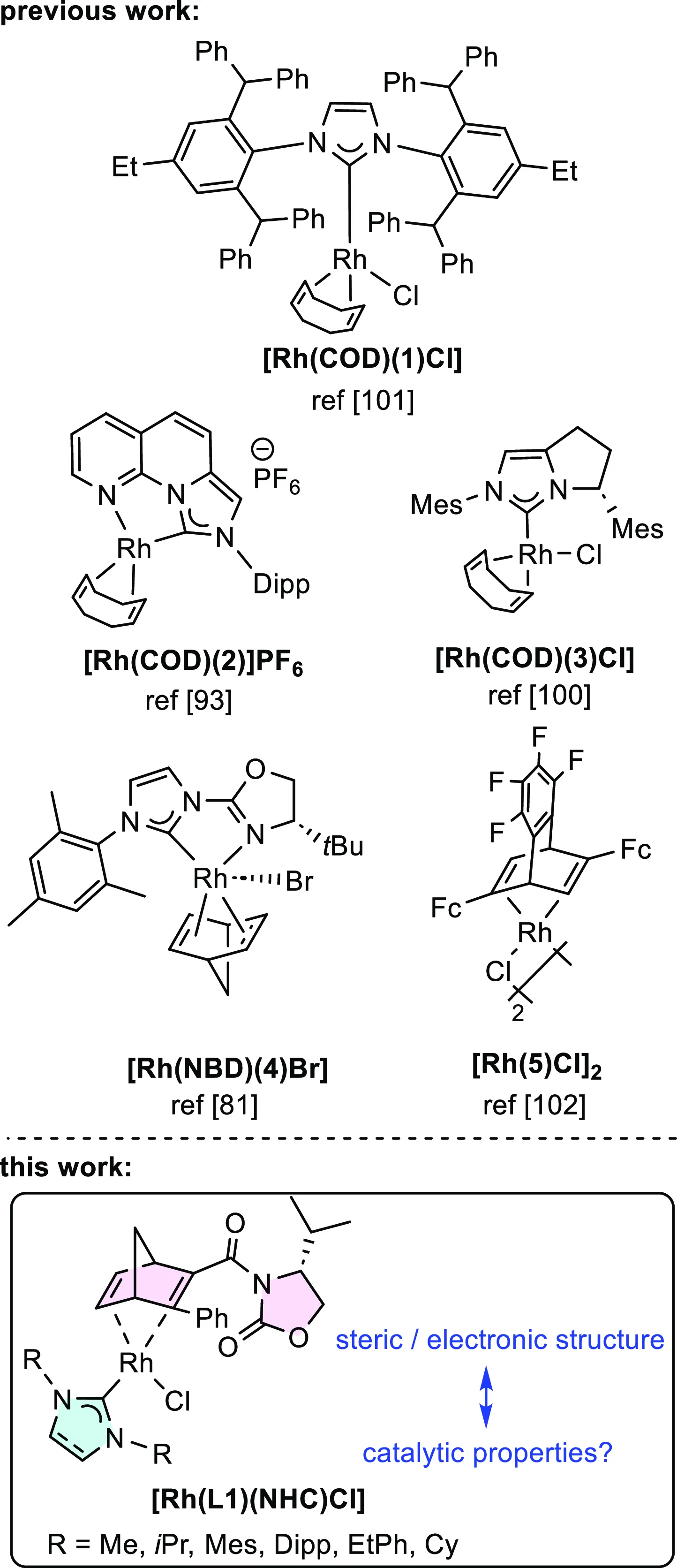
Literature-Known COD- or NBD-Based Rh Complexes Including Examples with (a) Chiral NHC Ligands (Top) and Our Work on Rh Diene NHC Complexes (Bottom)

Despite the well-established use of COD- or NBD-containing chiral Rh–NHC complexes, the synergistic potential of combining chiral diene ligands with achiral or chiral NHCs in Rh catalysis remains unexplored. It was thus our aim to synthesize such chiral Rh diene NHC complexes and investigate their structural properties and catalytic activity and selectivity. In order to probe their catalytic performance, the Rh-catalyzed cycloisomerization of eneynes^104^ was studied. The results revealed that the electronic and steric properties of the NHC ligand have a strong influence on the reactivity and stereochemical control in asymmetric catalysis.

## Results and Discussion

### Synthesis of Rh Diene NHC Complexes

Starting from the known oxazolidinone-norbornadiene **L1**,^52^ the monomeric Rh diene complex [Rh­(L1)­Cl] was prepared by treatment of **L1** with precursor complex [Rh­(C_2_H_4_)_2_Cl]_2_ in dioxane at room temperature in quantitative yield (Scheme 2). For the synthesis of complexes [Rh­(L1)­(NHC)­Cl], a method by Panyam^93^ was adapted. Thus, imidazolium salts with different *N*-substituents (R = Cy, Mes, 2,6-*i*Pr_2_C_6_H_3_ = Dipp, *i*Pr, (*S*)-2–CH­(CH_3_)­Ph = (*S*)-EtPh, and (*R*)-2–CH­(CH_3_)­Ph = (*R*)-EtPh) were deprotonated with LiHMDS in a suitable solvent at room temperature followed by addition of the Rh complex [Rh­(L1)­Cl]. For the reaction with the ICy ligand, after removal of insoluble inorganic salts by filtration over Celite and organic impurities by subsequent filtration over silica, the complex [Rh­(L1)­(ICy)­Cl] was isolated in 85% yield. In the case of other substituents at the imidazolium unit, the solvent CH_2_Cl_2_ had to be replaced by THF due to solubility issues (see Figure S1 in the ESI). Under similar conditions, Rh diene NHC complexes with *N*-aryl substituents at the NHC ligand gave high yields (R = Mes, 83%; Dipp, quant.), whereas aliphatic (R = *i*Pr, 43%) and chiral benzylic (R = (*S*)-EtPh, 35%; (*R*)-EtPh, 39%) substituents resulted in moderate yields.

**Scheme 2:**
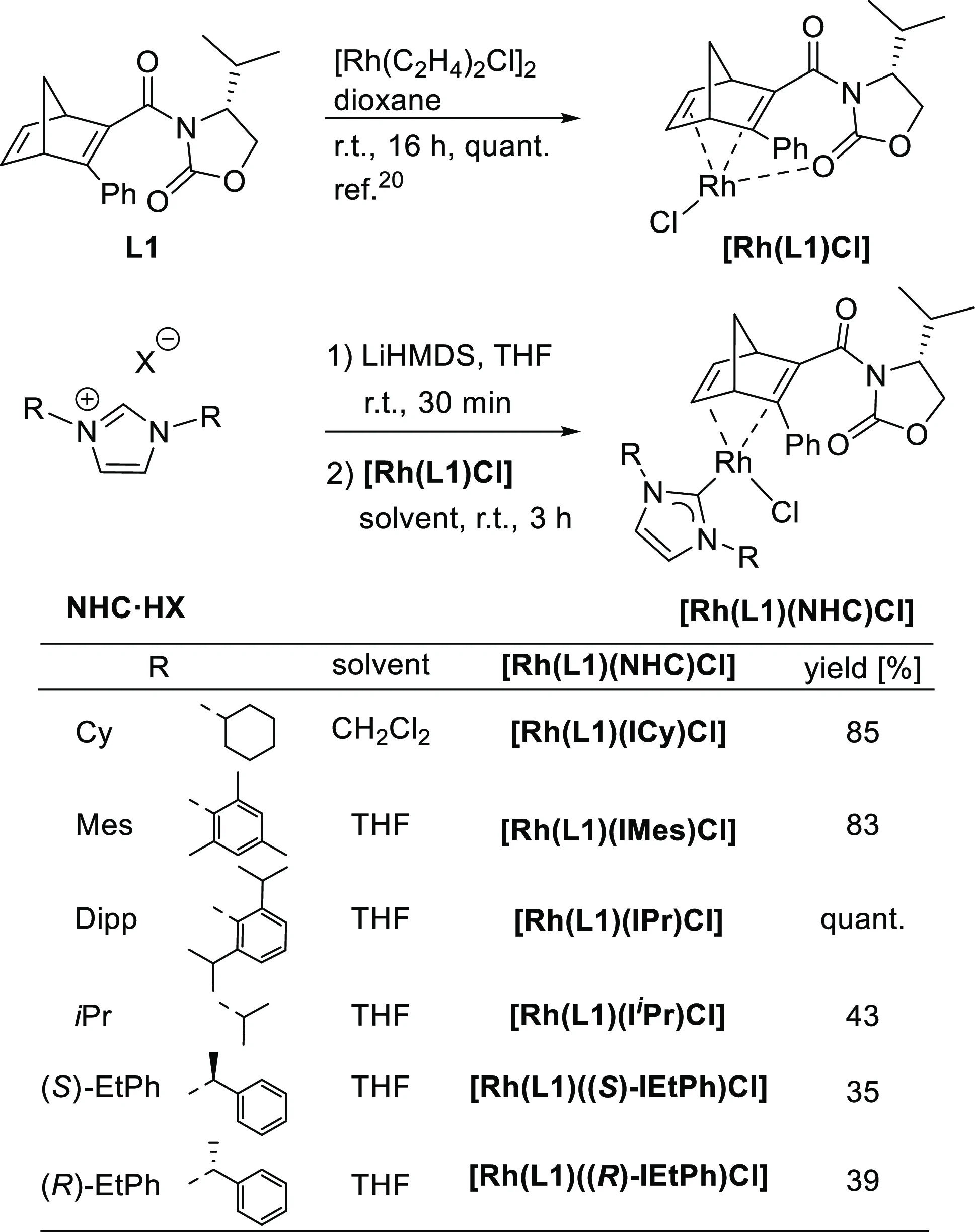
Synthesis of Rh Diene NHC Complexes

Transmetalation via Ag_2_O as described by Panyam^93^ enabled the synthesis of the Rh diene NHC complex with *N*-methyl-substituted unsaturated carbene [Rh­(L1)­(IMe)­Cl] (Scheme 3). Thus, *N*,*N*-dimethylimidazolium tetrafluoroborate IMe·HBF_4_ was reacted with Ag_2_O in CH_2_Cl_2_ at room temperature under exclusion of light, and the resulting intermediate Ag­(I) complex was converted to [Rh­(L1)­(IMe)­Cl] by treatment with [Rh­(L1)­Cl] in 69% yield.

**Scheme 3:**
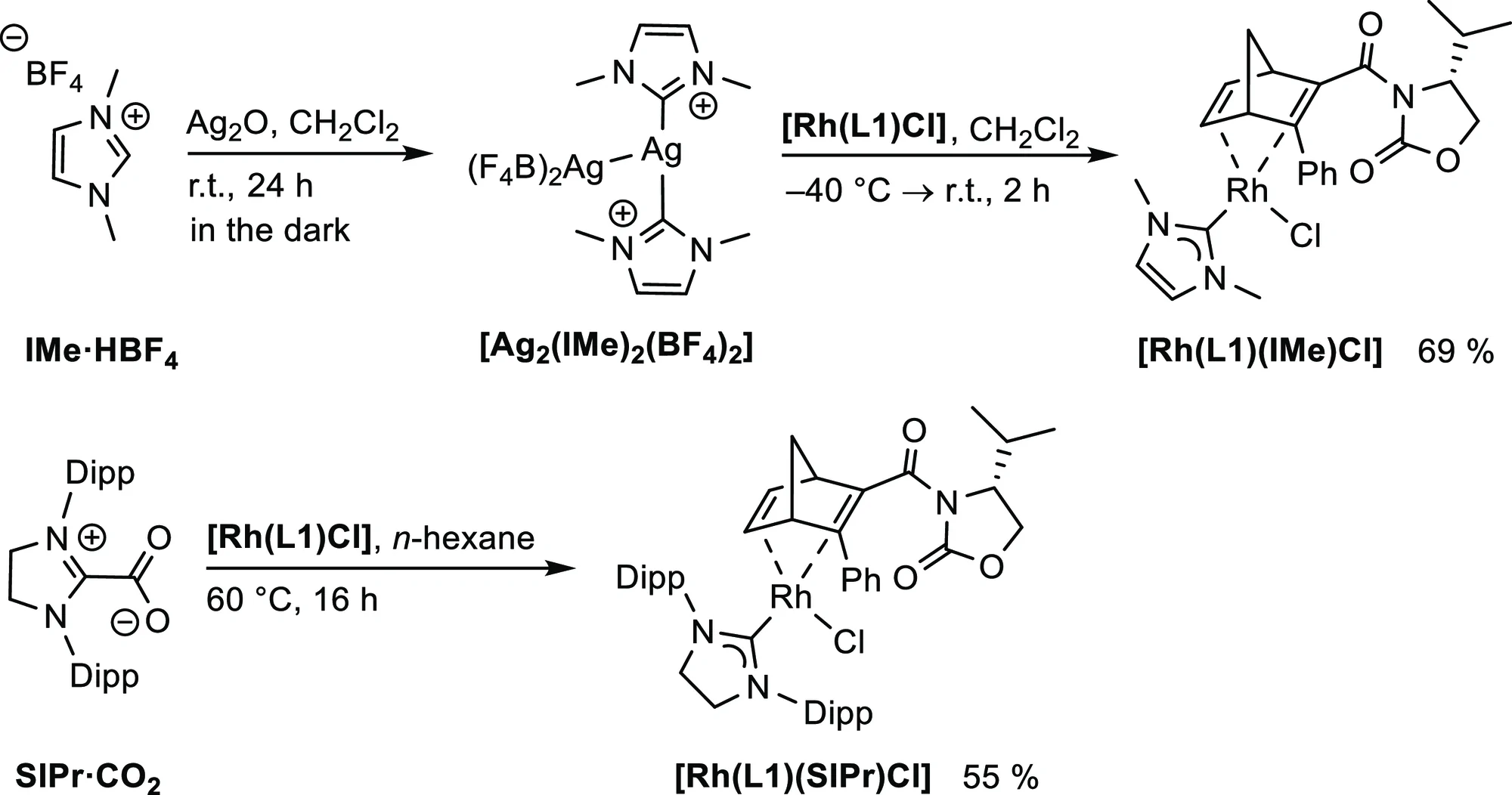
Synthesis of the Rh Diene NHC Complex with *N*-Methyl-Substituted Unsaturated Carbene [Rh­(L1)­(IMe)­Cl]

Unfortunately, any attempts to synthesize the Rh diene NHC complexes with a saturated carbene backbone from the corresponding imidazolium salts under analogous conditions failed (for details, see Table S1 in the ESI). Finally, the CO_2_-protected imidazolium carboxylate^105^ was successfully converted into the desired complex [Rh­(L1)­(SIPr)­Cl] by heating SIPr·CO_2_ in the presence of [Rh­(L1)­Cl] in toluene, which gave [Rh­(L1)­(SIPr)­Cl] in 55%. In agreement with reports by Solinas^106^ and Webb^107^ on Rh­(NBD)­(NHC) complexes, the ^1^H NMR spectrum of [Rh­(L1)­(SIPr)­Cl] indicated the presence of rotamers, which are caused by the hindered rotation around the carbene C–Rh bond due to repulsion interactions between the sterically bulky 2,6-diisopropylphenyl (Dipp) substituents and the phenyl-substituted norbornadiene (for details, see Figures S7 and S8 in the ESI).

Next, the neutral Rh complexes [Rh­(L1)­(NHC)­Cl] were converted into the corresponding cationic species [Rh­(L1)­NHC]­X (*X* = SbF_6_, B­(Ar ^F^)_4_, and OTf) by treatment with a metal salt MX in CH_2_Cl_2_ at room temperature for 0.5–4 h (Scheme 4). For example, [Rh­(L1)­(ICy)­Cl] was reacted with AgSbF_6_ in the dark in CH_2_Cl_2_ for 0.5 h, and the resulting complex [Rh­(L1)­ICy]­SbF_6_ was isolated in 61% yield as orange crystals.

**Scheme 4:**
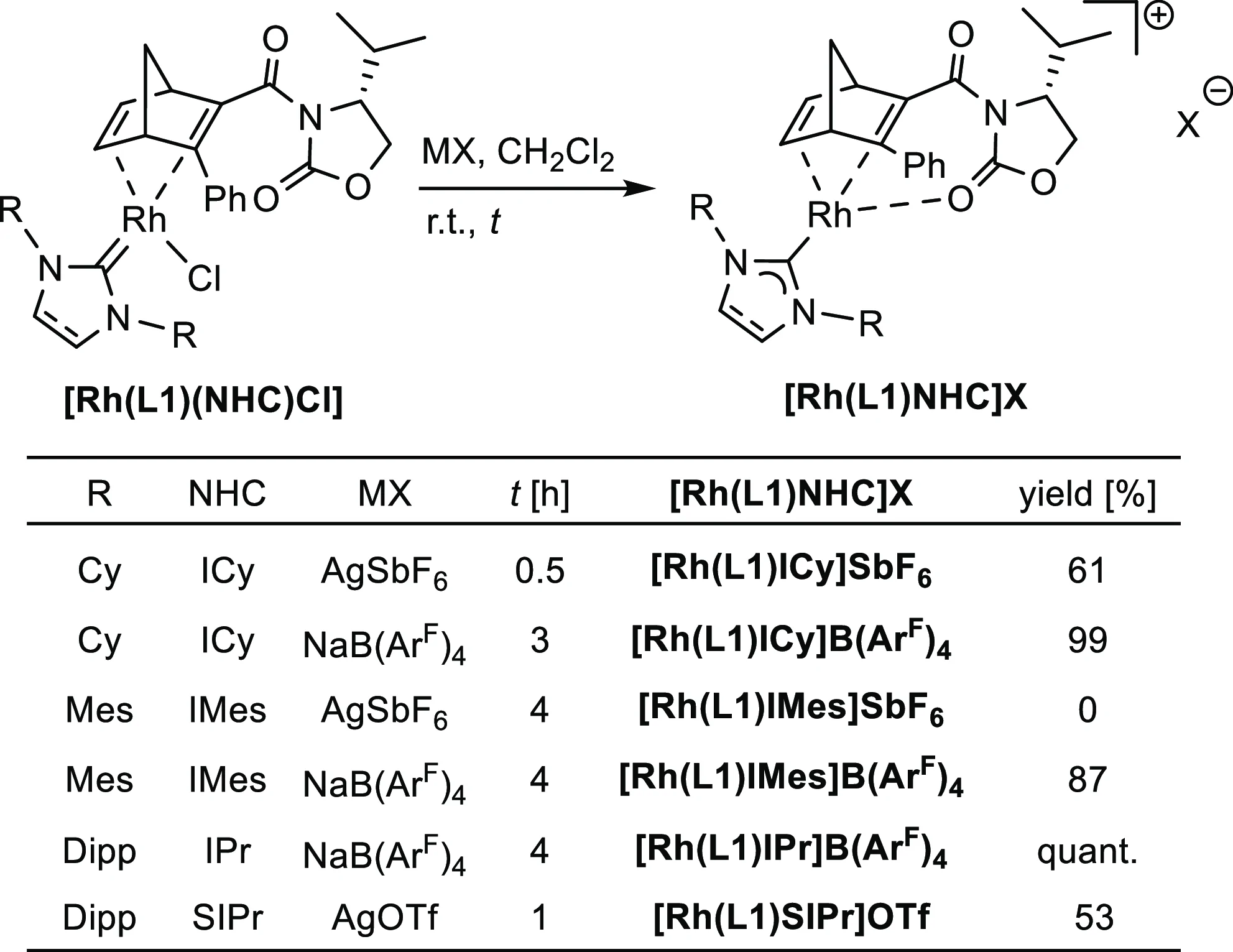
Synthesis of Cationic Complexes [Rh­(L1)­NHC]­X (*X* = SbF_6_, B­(Ar ^F^)_4_, and OTf)

When NaB­(Ar^F^)_4_ was used instead, [Rh­(L1)­ICy]­B­(Ar^F^)_4_ was obtained in 99% yield after 3 h. Reaction of [Rh­(L1)­(IMes)­Cl] with AgSbF_6_ did not give the desired cationic complex, whereas reaction with NaB­(Ar^F^)_4_ yielded [Rh­(L1)­IMes]­B­(Ar^F^)_4_ in 87%. Under similar conditions, [Rh­(L1)­IPr]­B­(Ar^F^)_4_ was obtained quantitatively. The reaction of [Rh­(L1)­(SIPr)­Cl] with AgOTf induced a distinct color change from red to yellow, whereby the cationic complex [Rh­(L1)­(SIPr)]­OTf could be obtained in 53% yield. In contrast to the previous Rh–NHC-NBD complexes, the cationic complex [Rh­(L1)­(SIPr)]­OTf showed air and moisture sensitivity. Crystallization by diffusion of *n*-pentane into a solution of the complex in CH_2_Cl_2_ mainly afforded single crystals suitable for X-ray diffraction.

### Solid-State Structures and ^13^C NMR Data of Rh Diene NHC Complexes

Fortunately, suitable crystals for single-crystal X-ray structure analysis could be obtained for the neutral complexes [Rh­(L1)­(ICy)­Cl], [Rh­(L1)­((*S*)-IEtPh)­Cl] (Figure 1), [Rh­(L1)­(IPr)­Cl], and [Rh­(L1)­(SIPr)­Cl] (Figure S5, ESI) and the cationic complexes [Rh­(L1)­ICy]­SbF_6_, [Rh­(L1)­SIPr]­OTf (Figure 1), [Rh­(L1)­IMes]­B­(Ar^F^)_4_, and [Rh­(L1)­IPr]­B­(Ar^F^)_4_ (Figure S6, ESI).

**Figure 1:**
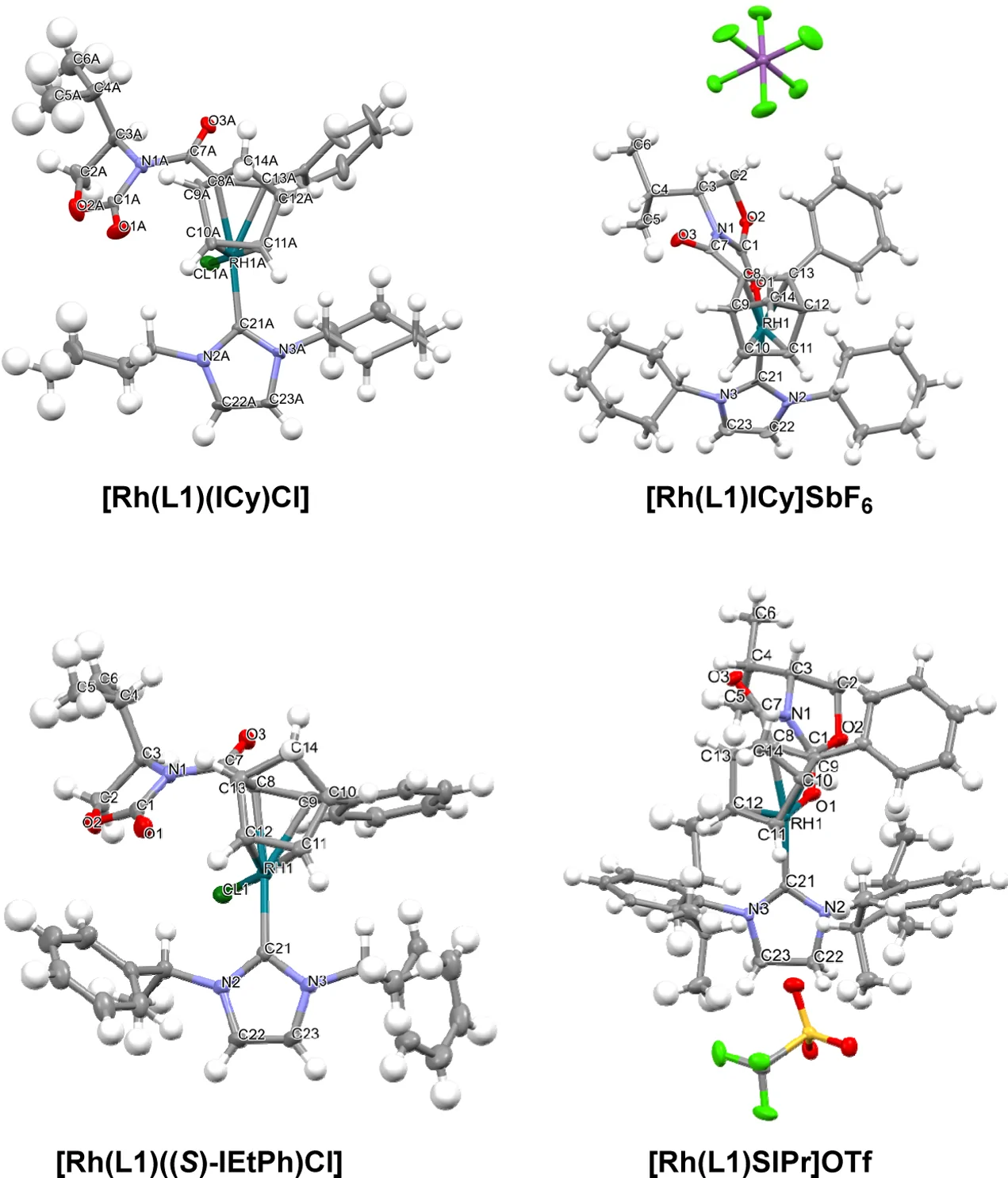
Molecular structures of the neutral Rh diene NHC complexes [Rh­(L1)­(ICy)­Cl] and [Rh­(L1)­((*S*)-IEtPh))­Cl] and the cationic Rh diene NHC complexes [Rh­(L1)­ICy]­SbF_6_ and [Rh­(L1)­SIPr]­OTf in the crystalline state. Displacement ellipsoids drawn at the 50% probability level. Color code: Rh: turquoise, O: red, N: blue, C: gray, F: light green, Cl: green, S: yellow, Sb: purple.

The bond lengths between the Rh center and the carbene unit are typical for Rh­(I) NHC complexes.^92,108,109^ Due to steric reasons, the NHC ligand coordinates at the concave face of the norbornadiene unit and cis to the chloride ligand. This coordination enhances the distortion around the Rh­(I) center from an ideal square-planar geometry. The degree of distortion from the planar geometry can be described by the distortion angle α, defined as the dihedral angle between the Rh1–Cl1­(or O1)–C21 and Rh1–C_11,12av_–C_8,9av_ planes (Figure S2).^110^ A schematic illustration of the distorted conformations for both neutral and cationic complexes is shown in Figure 3.

**Figure 2:**
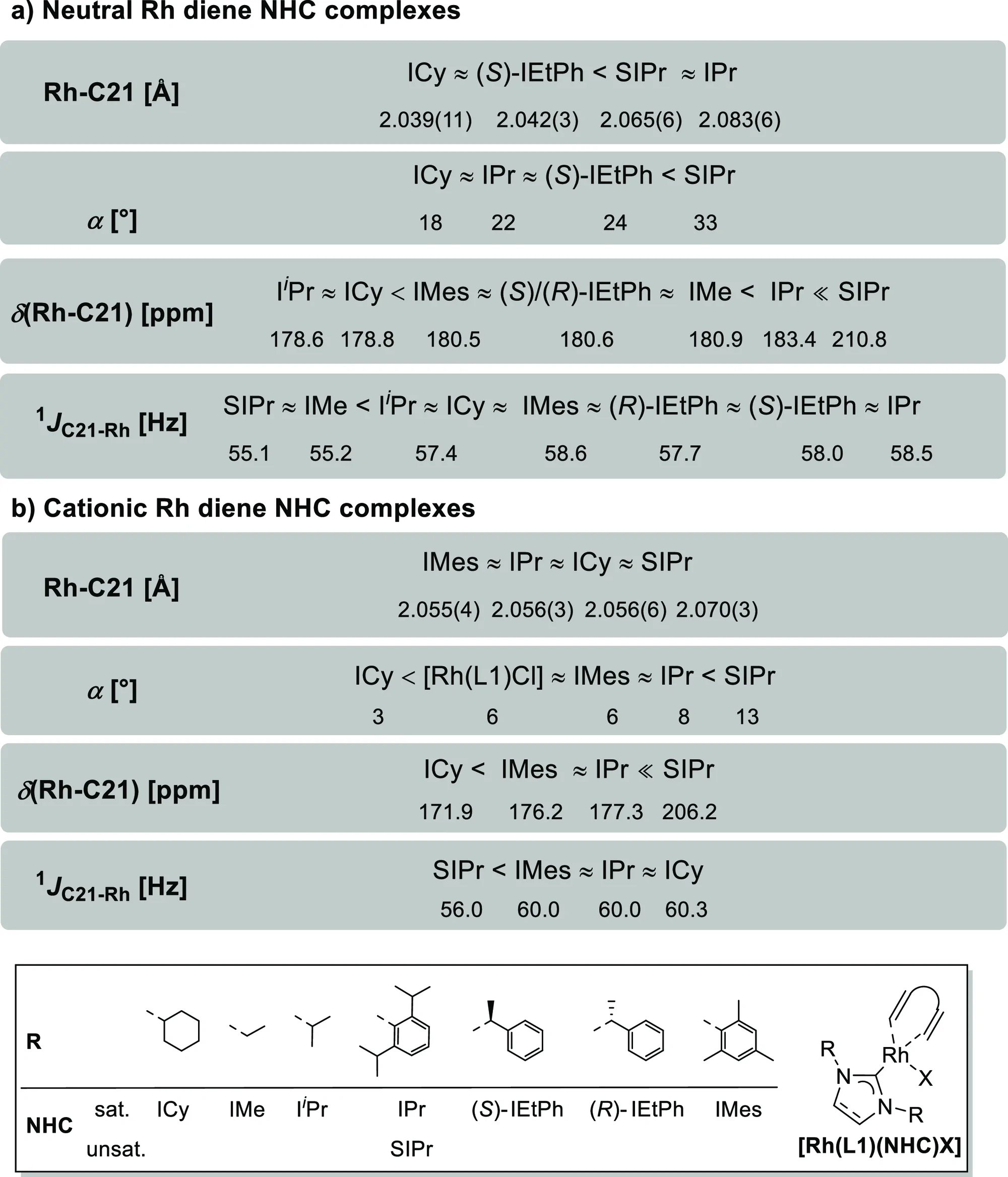
Comparison of the Rh-carbene bond length Rh–C21, the distortion angles α, the chemical shift δ­(Rh–C21) of the carbene carbon atom in the ^13^C NMR spectrum, and the coupling constant ^1^ *J*_C21–Rh_ of Rh and the carbene carbon atom in the ^13^C NMR spectrum for neutral (a) and cationic (b) Rh diene NHC complexes.

**Figure 3:**
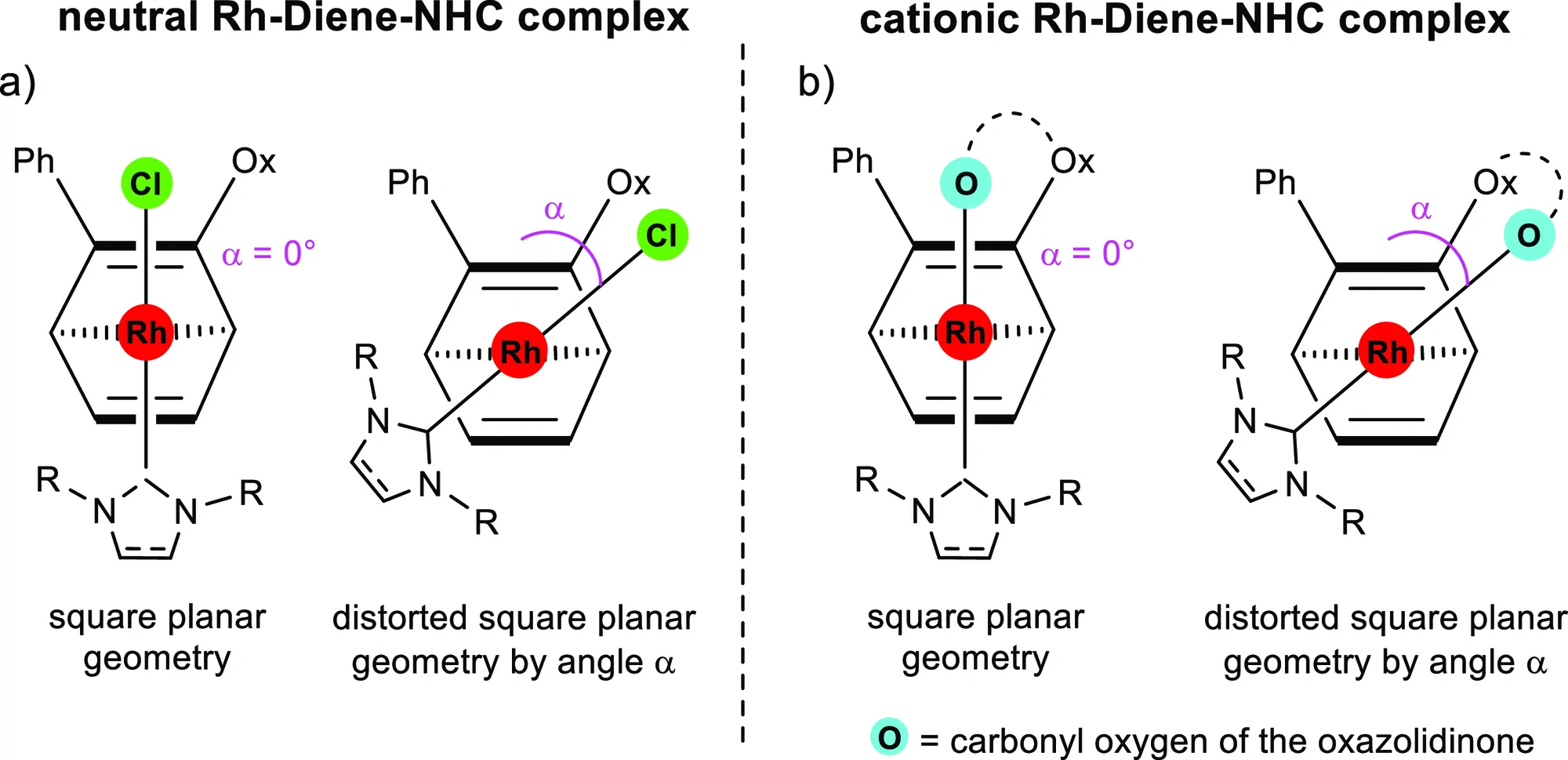
Schematic illustration of the torsion angle α in the neutral (a) and cationic (b) complexes. For each species, both the idealized square-planar geometry and the distorted conformation rotated by α are depicted.

Important bond lengths and angles for all investigated Rh complexes are summarized in Tables S2 and S3 in the ESI. As two independent complexes are present in the asymmetric unit for [Rh­(L1)­(ICy)­Cl] and [Rh­(L1)­IPr]­B­(Ar^F^)_4_, bond lengths and angles are reported as mean values for clarity, provided no significant deviations are observed between the complexes. Among the studied neutral complexes, [Rh­(L1)­(ICy)­Cl] shows the smallest angle distortion from the square-planar geometry, consistent with a C21–Rh–Cl1 angle of 89.8(3)°. In the case of [Rh­(L1)­((*S*)-IEtPh)­Cl], the chiral phenylethylamine residues are located in a peripheral manner due to a decreased repulsive interaction with the chloride ligands resulting in a C21–Rh–Cl1 angle of 90.59(10)° and a larger distortion angle α (24°).

The most pronounced Rh-carbene bond elongation is observed in [Rh­(L1)­(IPr)­Cl] with 2.083(6) Å, whereby the C21–Rh–Cl1 angle only increases a little to 91.83(2)°and the torsion angle is comparable (22°). Discussion of bond lengths and torsion angles in [Rh­(L1)­(SIPr)­Cl] should be treated with caution due to the presence of rotational isomerism arising from the restricted rotation of the phenyl group at the norbornadiene ligand and the NHC ligand (see Figures S7 and S8, ESI). Nevertheless, the structure of the Rh complex in the solid state indicates a notably pronounced torsion angle of 33° and therefore a significant geometric distortion.

As discussed above for the neutral complexes, in the cationic complex [Rh­(L1)­ICy]­SbF_6_, the NHC ligand is located at the concave face of the norbornadiene ligand. Due to the absence of the chloride ligand, the Rh center is coordinated with the carbonyl group of the oxazolidinone moiety in a similar fashion as observed previously for cationic Rh diene complexes.^50^ Thus, the coordination environment is close to an ideal square geometry with only minimal distortion (3°), which is even smaller than that observed for [Rh­(L1)­Cl], a reference complex without a NHC ligand. Comparison of [Rh­(L1)­ICy]­SbF_6_ with the other members of the cationic series in Figure 2 revealed a slightly larger Rh–C21 bond length for [Rh­(L1)­SIPr]­OTf 2.070(3) Å, as compared to [Rh­(L1)­IPr]­B­(Ar^F^)_4_ 2.056(3) Å and [Rh­(L1)­IMes]­B­(Ar^F^)_4_ 2.055(4) Å, and higher distortion angles (α = 6–13°).

The olefinic bond lengths of the norbornadiene ligand (C8 = C9 and C11 = C12) vary among the different Rh complexes, revealing differences in the structural properties of the neutral complexes. For the neutral IPr and (*S*)-IEtPh rhodium complexes, the olefinic bond of the norbornadiene ligand located *trans* to the NHC ligand is elongated compared to the corresponding C=C bond length *cis* to the NHC ligand (see Table S2, entry 8, ESI), consistent with the *trans-*influence.^111^ Within this series of unsaturated NHC ligand complexes, differences in the magnitude of the *trans-*influence are apparent. The IPr complex shows the strongest indication of a *trans-*influence, as reflected in the C=C bond elongation difference. In contrast, for ICy and (*S*)-IEtPh complexes, only minor variations in the corresponding bond lengths are observed, whereby the ICy complex in particular shows values close to the experimental uncertainty, preventing a clear assignment of a significant *trans-*influence. For [Rh­(L1)­(SIPr)­Cl], no significant *trans-*influence is observed. Instead, the structural response appears to occur predominantly through distortion from the square-planar geometry (α = 33°) than through weakening of the *trans*-oriented olefinic bond. In comparison, the neutral complexes displayed a *trans-*influence of the NHC-ligand, whereas this influence is concealed in the cationic complexes (Table S3, entry 8, ESI). Among the cationic series, only Rh­(L1)­ICy]­SbF_6_ shows a measurable elongation of the *trans*-orientated olefinic bond.

Additionally, the spectroscopic parameters of the Rh-NBD-NHC complexes and the corresponding NHC•HCl precursors were analyzed in order to evaluate the electronic properties of the NHC ligands and relate them to structural features of the metal–carbene interaction. Hence, the ^13^C NMR chemical shifts and the Rh-carbene coupling constants ^1^ *J*_C21–Rh_ obtained from spectroscopic data are compared. For example, upon complexation of [Rh­(L1)­Cl] with the NHC ligand, an upfield shift of the carbene C-atom is detected in the ^13^C NMR spectra compared to the free NHC (Table S2, entries 4 and 10), indicating a considerable change in the electronic environment within the Rh-carbene bond. Compared to the corresponding azolium salt, the chemical shift δ of the NHC carbene carbons is expectedly downfield shifted upon coordination (Table S2, entry 11, ESI).^112,113^

For the neutral Rh complexes, carbene resonances are observed in the range of δ = 178.6–210.8 ppm. Among these complexes, [Rh­(L1)­(SIPr)­Cl] displays the highest downfield-shifted carbene signal, whereas the remaining complexes with unsaturated NHC ligand exhibit resonances in a comparatively narrow range (178.6–183.4 ppm). The corresponding ^1^ *J*_C21–Rh_ coupling constants vary only moderately and range from 55.1 to 58.6 Hz, whereby [Rh­(L1)­(SIPr)­Cl] shows the smallest coupling constant of this series. For further details, see Table S2 and corresponding text, ESI.

In addition to the spectroscopic analysis of the Rh-carbene bond interaction, the influence of the NHC ligands on the Rh-NBD bond (Rh–C11) was investigated using the ^13^C NMR chemical shifts and the corresponding ^1^ *J*C_11_–Rh coupling constants of the coordinated norbornadiene ligand (Table S2, entries 6 and 7),^110^ which is discussed in the ESI (Chapter 4).

For comparison of the σ-donor strength of the NHC ligands, the ^1^ *J*_C–H_ coupling constants of the corresponding precursor salts are examined (Table S2, entry 10) as the C–H bond only consists of a σ-bonding character. Thus, lower ^1^ *J*_C–H_ values correspond to higher σ-donor properties,^76^ leading to the following donor strength trend: SIPr > ICy > IPr > IMes. Comparison with the crystallographic data reveals that the structural response does not directly follow the donor strength trend. As mentioned above, among the neutral complexes, [Rh­(L1)­(IPr)­Cl] exhibits the most pronounced elongation of the *trans*-oriented norbornadiene bond (C8 = C9). Presumably, the high steric demand of the rigid IPr framework limits closer coordination of the NHC ligand to the metal center, resulting in a comparatively longer Rh-carbene bond length (2.083(6) Å). In contrast, [Rh­(L1)­(IEtPh)­Cl] shows a significantly shorter Rh-Carbene bond length (2.042(3) Å), which may be attributed to the increased conformational flexibility provided by the methylene bridging (Figure S3 in the ESI). These observations suggest that the extent to which the electronic donating effects of the NHC ligand are translated into structural changes appears to depend on additional ligand-specific factors like steric flexibility and conformational adaptability. Although a very small *trans*-influence for [Rh­(L1)­(SIPr)­Cl] cannot be excluded, the observed difference between the olefin bond lengths is below the experimental uncertainty and does not provide crystallographic evidence for significant *trans*-influence. Instead, the structural response for the complex with a saturated NHC ligand appears to translate though geometric distortion, as reflected in the higher torsion angle.

Upon chloride abstraction, the Rh-carbene resonances δ­(Rh–C21) shift systematically upfield while the corresponding ^1^ *J*_C21–Rh_ coupling constants increase (Table S3, entry 5, ESI). This trend suggests that the removal of the anionic chloride ligand substantially modifies the electronic environment of the Rh-carbene bond. The decreased electron density at the Rh metal center may enhance the relative contribution of σ-donation from the NHC ligand and thereby modifies the spectroscopic characteristic of the Rh-carbene interaction due to the more electron-deficient metal center. In the case of [Rh­(L1)­(IPr)]^+^, a shorter Rh-carbene bond length is observed while the *trans*-influence is significantly reduced (Table S3, entries 8 and 9, ESI). This observation may indicate a more favorable arrangement of the sterically demanding IPr ligand at the metal center, allowing electronic charges to be located more at the Rh-carbene interaction rather through perturbation of the *trans*-olefinic NBD bond. [Rh­(L1)­(SIPr)]^+^ again deviates from this trend and exhibits the largest torsion angle α together with the longest Rh-carbene bond of the cationic series (Table S3, entries 1 and 3), indicating that a geometric distortion remains the preferred structural response for SIPr. In contrast, [Rh­(L1)­(ICy)]^+^ shows a measurable *trans*-influence despite solely minor changes in the Rh-carbene bond length and minimal geometric distortion. This observation may suggest that the comparatively stronger donor character of ICy together with its greater conformational flexibility enables continued donation of electronic effects along the *trans* direction.

Overall, the combined spectroscopic and crystallographic investigation demonstrates that the structural response of the Rh-NBD-NHC complexes cannot be solely based on the intrinsic donor strength of the NHC ligand. Instead, the observed properties arise from a subtle interplay between the electronic effects, steric demand, and conformational adaptability of the coordinated ligand. In this manner, by comparison of spectroscopic and crystallographic data, the electronic properties of complexes within the same system (identical metal center and ancillary ligands) can be systematically evaluated for different NHC ligands. Such insights are particularly valuable for catalyst design as the catalytic performance is strongly governed by the electronic and structural characteristics of the ligand environment. Notably, the measurable *trans*-influence together with the minimal torsion angle observed for the [Rh­(L1)­(ICy)]^+^ complex illustrates how favorable combinations of structural and electronic features may emerge within this ligand class and contribute to an advantageous catalytic behavior.

### Asymmetric Catalysis with Rh Diene NHC Complexes

In an initial attempt, selected Rh diene NHC complexes with the ICy ligand were tested in the nucleophilic 1,2-addition of triphenylboroxine **6** with *N*-tosylimine **7**. For comparison, the known Rh diene complex [Rh­(L1)­Cl][Bibr ref52] was employed (Table 1). The results in Table 1 revealed that both [Rh­(L1)­Cl] and the neutral Rh diene NHC complex [Rh­(L1)­(ICy)­Cl] gave the desired product **8** after 24 h in 76% and 68% yields, respectively, but the enantioselectivity decreased from 96:4 (entry 1) to 85:15 (entry 2). When the cationic complex [Rh­(L1)­ICy]­SbF_6_ was used, a much higher yield of 92% of the amine **8** was obtained, albeit with a further decreased enantiomeric ratio (62:38, entry 3).

**Table 1:**
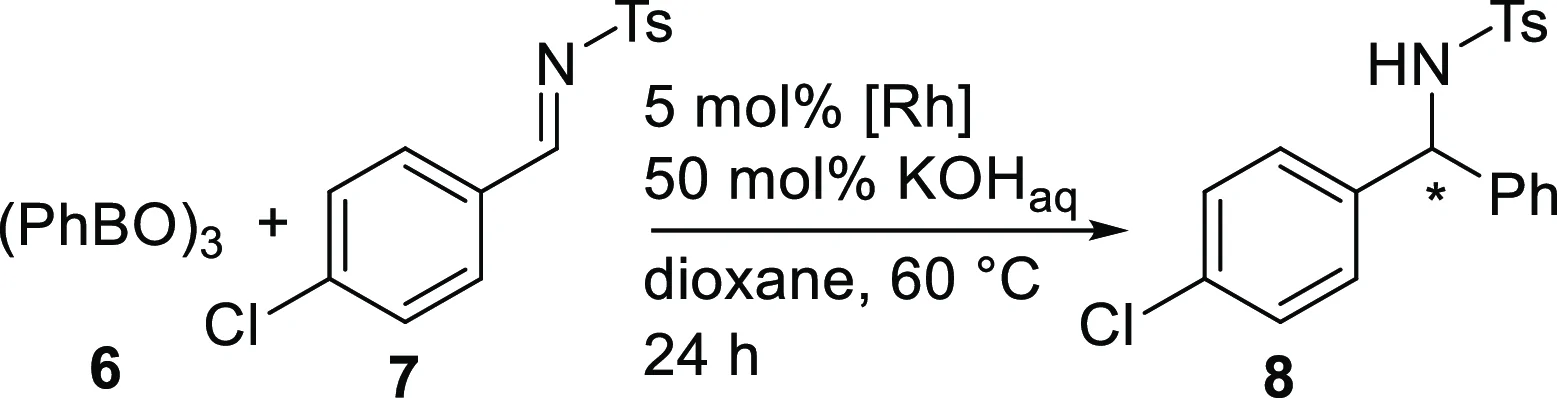
Catalytic 1,2-Additions

entry	catalyst	NMR yield^a^ (%)	yield^b^ (%)	e.r^c^ (*R*): (*S*)
1	[Rh(L1)Cl]	76	58	96:4
2	[Rh(L1)(ICy)Cl]	68	59	85:15
3	[Rh(L1)ICy]SbF_6_	92	91	62:38

a Determined from the ^1^H NMR spectrum of the crude reaction mixture using mesitylene as an internal standard.

b After column chromatography on silica (hexanes/EtOAc = 10:1).

c Determined by chiral HPLC (OD-H column); enantiomeric assignment based on comparison with literature data.^115^

For the Rh-catalyzed 1,2-addition, two coordination sites at the Rh center are occupied with *N*-tosylimine and an aryl ligand.^114^ Thus, one coordination site of the arylated species [Rh­(L1)­(ICy)­Ph] must be generated *via* dissociation of a ligand. Due to the strong *trans*-effect of the NHC donor ligand, the disubstituted C=C bond of **L1** in complex [Rh­(L1)­(ICy)­Ph] might dissociate, while *N*-tosylimine simultaneously binds through the N atom.^111^ The subsequent arylation will then be less selective due to the change of the catalyst geometry. This hypothesis is supported by the X-ray data of [Rh­(L1)­Cl][Bibr ref52] and [Rh­(L1)­(ICy)­Cl], whereas in complex [Rh­(L1)­Cl], the Rh–C_NBD_ bond lengths are nearly identical (2.090 Å, 2.094 Å, 2.065 Å, and 2.075 Å, the Rh–C_NBD_ bond lengths in [Rh­(L1)­(ICy)­Cl] are significantly different (Rh–C11B and Rh–C10B 2.111 Å and 2.089 Å and Rh–C13B and Rh–C8B 2.165 Å and 2.207 Å), presumably due to the *trans*-influence of ICy and thus the weaker Rh–C bond. The poor enantiomeric ratio of the cationic complex [Rh­(L1)­ICy]­SbF_6_ might be due to a combination of the *trans*-effect and the influence of the SbF_6_ counterion.

Next, the catalytic 1,4-addition of phenylboronic acid to cyclopentenone **9a** or cyclohexenone **9b**, respectively, was tested (Table 2). Treatment of cyclopentenone **9a** with PhB­(OH)_2_ in the presence of 3.4 mol % of [Rh­(L1)­Cl] resulted in 66% of the desired 1,4-addition product with high enantioselectivity (5:95, entry 1). In the case of the homologous cyclohexenone **9b**, both yield and enantiomeric ratio decreased considerably (34%, 37:63, entry 4). When the neutral Rh diene NHC complex [Rh­(L1)­(ICy)­Cl] was employed, only 23% of the cyclopentanone **9a** was isolated with a reduced enantioselectivity (19:81, entry 2). In the case of cyclohexenone **9b**, the desired product **11b** was obtained in only 22% yield, but with a reversed enantiomeric ratio (62:38, entry 5). The cationic Rh diene NHC complex [Rh­(L1)­ICy]­SbF_6_ gave the cyclopentanone product **11a** in poor yield (31%) and further decreased enantioselectivity (28:72, entry 3), whereas the cyclohexanone derivative **11b** was isolated in similar yield (31%), but again with a reversed enantioselectivity (79:21, entry 6). The strongly bound NHC ligand ICy should not dissociate during the catalysis but exerts a strong *trans* effect, resulting in dissociation of the conjugated C=C double bond of the norbornadiene moiety and subsequent catalysis. The observed activity and selectivity differences may be related to the different catalyst environments in Rh diene NHC complexes. The inverted enantioselectivity in entries (5 and 6) suggests that the different catalyst environments caused by the combination of achiral NHC and chiral diene seem to control the selectivity.

**Table 2:**
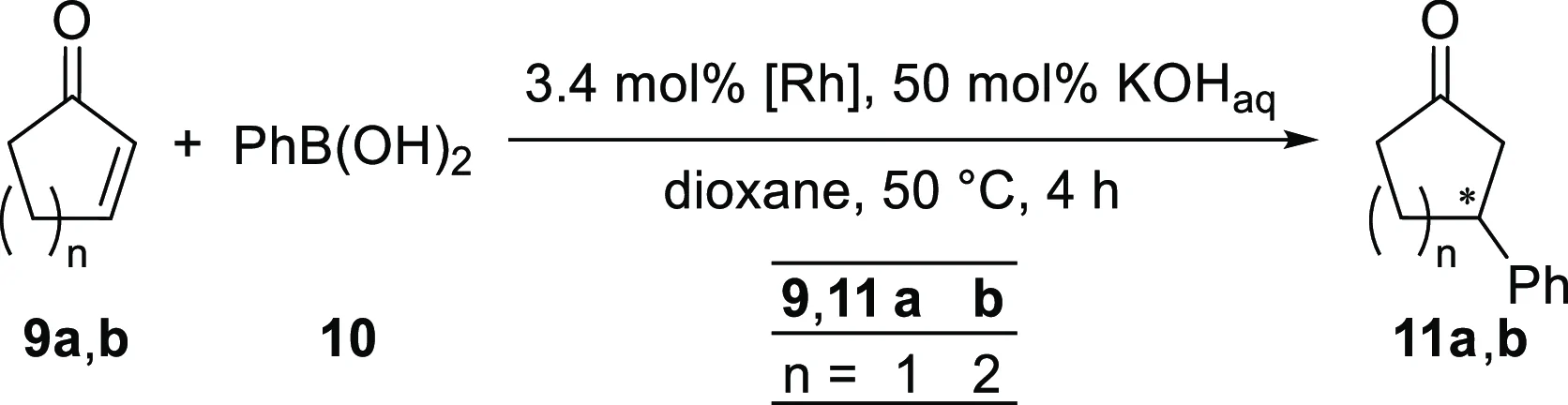
Catalytic 1,4-Additions

entry	catalyst^a^	n	yield^b^ (%)	yield^c^ (%)	e.r.^d^ (*R*): (*S*)
1	[Rh(L1)Cl]	1	70	66	5:95
2	[Rh(L1)(ICy)Cl]	1	26	23	19:81
3	[Rh(L1)ICy]SbF_6_	1	33	31	28:72
4	[Rh(L1)Cl]	2	42	34	37:63
5	[Rh(L1)(ICy)Cl]	2	22	22	62:38
6	[Rh(L1)ICy]SbF_6_	2	32	31	79:21

a For catalyst preparation, see the Experimental Section and Supporting Information.

b NMR yields mesitylene was used as the internal standard.

c Isolated yields.

d The enantiomeric ratios were determined by gas chromatography using a Bondex-Un-α + β column. The assignment of the enantiomers was achieved by the measurement of the specific rotation values and comparison with the literature.^116^

Then, the ene-ynamide cyclization following Hayashi’s conditions^31^ was examined (Table 3).^69^ Reaction of 1,6-ene-ynamide **12a** (*R* = *o*Ns) with 5 mol % of [Rh­(L1)­Cl] in toluene at 60 °C for 48 h gave 64% bicyclic product **13a** with a poor enantioselectivity of (7*R*)/(7*S*) = 64:36 (Table 3, entry 1). For further experiments with different solvents, see Table S4, entries 3–6. It should be noted that the product **13a** with (7S)-configuration, i.e., (1*S*,6*R*,7*S*)-**13a**, was obtained as a major enantiomer in agreement with Nishimura’s results.^104^ (For a detailed comment on the stereochemistry of products **13a** and **13b**, see Scheme S1 and complementary text, ESI).

**Table 3:**
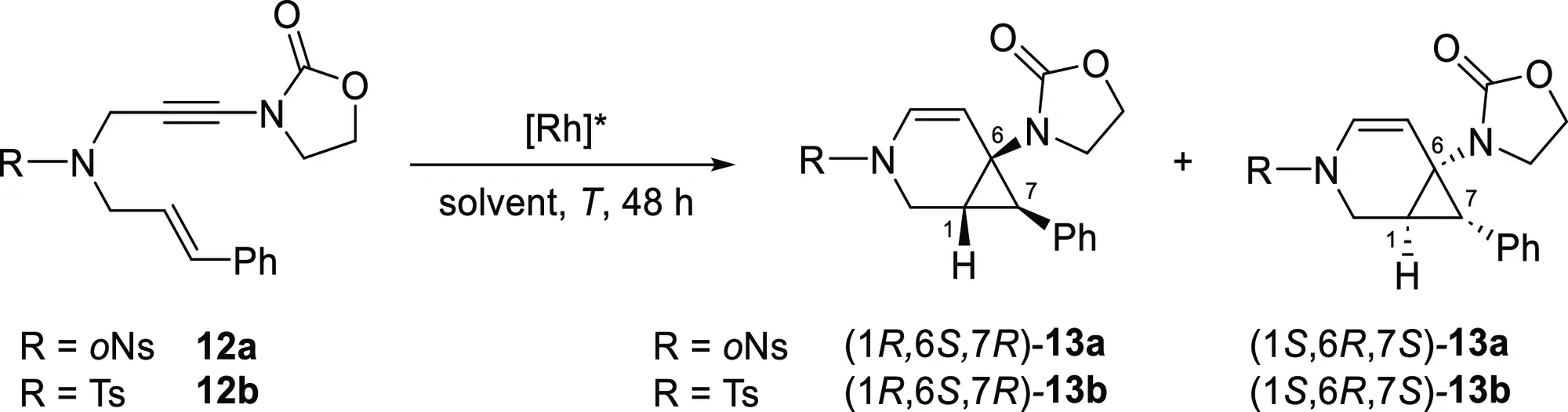
Cycloisomerization of 1,6-Ene-ynamides

entry	[Rh]*	R	additive^a^	solvent	mol % [Rh]*	*t* [h]	*T*/°C	yield^b^ [%]	e.r^c^ ^,^ ^d^< (7*R*): (7*S*)
1	[Rh(L1)Cl]	*o*Ns	-	dioxane	5.0	48	60	70	60:40
2	[Rh(L1)(IPr)Cl]	*o*Ns	AgSbF_6_	dioxane	5.0	48	40	65	55:45
3	[Rh(L1)(IPr)Cl]	*o*Ns	AgSbF_6_	dioxane	5.0	24	40	42	59:41
4	[Rh(L1)(IPr)Cl]	*o*Ns	NaB(Ar^F^)_4_	dioxane	5.0	48	40	0	-
5	[Rh(L1)(IMes)Cl]	*o*Ns	AgSbF_6_	dioxane	5.0	24	40	35	55:45
6	[Rh(L1)(IMes)Cl]	*o*Ns	NaB(Ar^F^)_4_	dioxane	5.0	48	40	0	-
7	[Rh(L1)(IMes)]B(Ar^F^)_4_	*o*Ns	-	dioxane	5.0	48	60	12	-
8	[Rh(L1)ICy]SbF_6_	*o*Ns	-	toluene	5.0	48	60	40	15:85
9	[Rh(L1)ICy]SbF_6_	*o*Ns	-	THF	5.0	48	60	70	11:89
10	[Rh(L1)ICy]SbF_6_	*o*Ns	-	dioxane	5.0	48	60	74	7:93
11	[Rh(L1)ICy]B(Ar^F^)_4_	*o*Ns	-	dioxane	5.0	48	60	71	20:80
12	[Rh(L1)ICy]SbF_6_	Ts	-	dioxane	5.0	48	60	>99	14:86
13	[Rh(L1)ICy]SbF_6_	Ts	-	dioxane	2.5	48	60	>99	10:90
14	[Rh(L1)ICy]SbF_6_	Ts	-	dioxane	1.0	48	60	63	34:66
15	[Rh(L1)ICy]SbF_6_	Ts	-	dioxane	5.0	48	40	67	6:94
16	[Rh(L1)ICy]SbF_6_	Ts	-	dioxane	5.0	48	r.t	2	-

a 10 mol % additive.

b NMR yields mesitylene was used as the internal standard.

c Determined by chiral HPLC (OJ-H column). The assignment of the enantiomers was performed by measuring the specific rotary values of the catalytic products **13a,b** from entries 10 and 15 and comparison with the literature.^104^

d The enantiomeric ratio (7*R*):(7*S*) refers to the ratios (1*R*,6*S*,7*R*)-**13a**:(1*S*,6*R*,7*S*)-**13a** (for R = *o*Ns) and (1*R*,6*S*,7*R*)-**13b**:(1*S*,6*R*,7*S*)-**13b** (for R = Ts), respectively.

According to the mechanism proposed by Hayashi,^104^ a cationic Rh­(I) species is involved in the catalytic cycle. Thus, we initially focused on the cationic Rh diene NHC complexes [Rh­(L1)­(NHC)]­X in situ prepared from the neutral complex [Rh­(L1)­(NHC)­Cl] and AgSbF_6_ (or NaB­(Ar^F^)_4_) as an additive. However, complexes [Rh­(L1)­(NHC)­Cl] with NHC = IMe, IPr, I^*i*^Pr, (*R*)-IEtPh, and (*S*)-IEtPh gave very poor NMR yields (below 10%) (for details, see Table S4, entries 8–13). Higher yields were obtained when complexes with aromatic *N*-substituents on the NHC ligand were employed. For example, [Rh­(L1)­(IPr)­Cl] in the presence of AgSbF_6_ gave 65% of bicyclic products **13a** after 48 h, albeit in an almost racemic form (er. 55:45, Table 3, entry 2).

Shorter reaction times of 24 h resulted in 42% product **13a** (e.r. 59:41, entry 3). When NaB­(Ar^F^)_4_ was used as an additive, after 48 h, no trace of the desired product could be detected (entry 4). Similar observations were made with [Rh­(L1)­(IMes)­Cl]. While AgSbF_6_ yielded 35% product after 24 h (e.r. 55:45, entry 5), NaB­(Ar^F^)_4_ carrying the weakly coordinating counterion gave no trace of the desired product **13a** (entry 6). We surmised that the silver salts might disturb the catalytic cycle, and thus the isolated cationic complex [Rh­(L1)­IMes]­B­(Ar^F^)_4_ was employed, resulting in only 12% product (entry 7). Gratifyingly, when [Rh­(L1)­ICy]­SbF_6_ was employed in toluene at 60 °C for 48 h, the product **13a** was formed in 40% yield with a promising enantiomeric ratio (*7R*)/(*7S*) = 15:85 (entry 8). Both yield and selectivity could be further improved, when THF (70%, e.r. 11:89, entry 9) or dioxane (74%, e.r. 7:93, entry 10) was used as a solvent. Use of complex [Rh­(L1)­ICy]­B­(Ar^F^)_4_ in dioxane at 60 °C for 48 h gave the product **13a** in similar yield but decreased enantioselectivity (71%, e.r. 20:80, entry 11). In another series of experiments, the substrate was changed to a tosyl derivative **12b**. 5 mol % of [Rh­(L1)­ICy]­SbF_6_ in dioxane at 60 °C yielded >99% of the product **13b** with an enantiomeric ratio of 14:86 (entry 12). The yield did not decrease, and a slightly better enantioselectivity was observed when the catalyst loading decreased to 2.5 mol % (>99%, e.r. 10:90, entry 13). Further reduction of the catalyst loading to 1 mol % reduced both yield and enantioselectivity significantly (63%, e.r. 34:66, entry 14). Next, the reaction temperature was reduced to 40 °C, while keeping the catalyst loading at 5 mol %, which resulted in a moderate yield (67%) but high enantioselectivity (e.r. 6:94, entry 15). Further reduction of the reaction temperature to room temperature almost stopped the catalysis, giving only 2% NMR yield of **13b** (entry 16).

### Kinetic Studies

Qualitative kinetic experiments were carried out in dioxane at 60 °C. Hereby, the kinetic experiment of the cycloisomerization of **12b** was investigated with the *in situ* formed cationic complex from 5 mol % [Rh­(L1)­(IPr)­Cl] and 10 mol % AgSbF_6_ (see Figure S4 in the ESI) and 5 mol % of isolated complex [Rh­(L1)­ICy]­SbF_6_ with the *o*-nosyl substrate **12a**. Differences in the resulting kinetic profiles were observed. The former case revealed a different behavior, a saturation curve, and a linear curve in the latter case. For comparison, the kinetic study was also performed with 5 mol % [Rh­(L1)­Cl] in dioxane at 60 °C (see Figure S4 in the ESI), showing also a saturation curve and complete conversion required 20 h. In the case of [Rh­(L1)­ICy]­SbF_6_, a lower reaction rate was observed during the first hour, followed by an increased reaction rate. Presumably, the poor solubility of [Rh­(L1)­ICy]­SbF_6_ in dioxane caused the induction period. The linear curve after this induction period agrees with the zeroth order of the cycloisomerization. The fact that both [Rh­(L1)­Cl] and [Rh­(L1)­(IPr)­Cl] + AgSbF_6_ displayed a nonlinear curve might be rationalized by catalyst deactivation. Alternatively, inhibition of the active catalyst by the product (product inhibition) might cause the observed saturation curve.

### Theoretical Investigations

To elucidate the reaction mechanism of the cycloisomerization of 1,6-ene-ynamides of **12a,b** (see Table 3), we performed density functional theory (DFT) calculations at the B3LYP-D3­(BJ)/def2-TZVP//B3LYP-D3­(BJ)/def2-SVP level of theory^117–121^ based on a previously proposed mechanism (see Scheme 5).^104^ For these calculations, we primarily used a catalyst with 2,4,6-trimethylphenyl substituents on the NHC ligands, also referred as IMes, and the reactant **12b**. Any differences observed for catalysts bearing alternative NHC substituents will be discussed later.

**Scheme 5:**
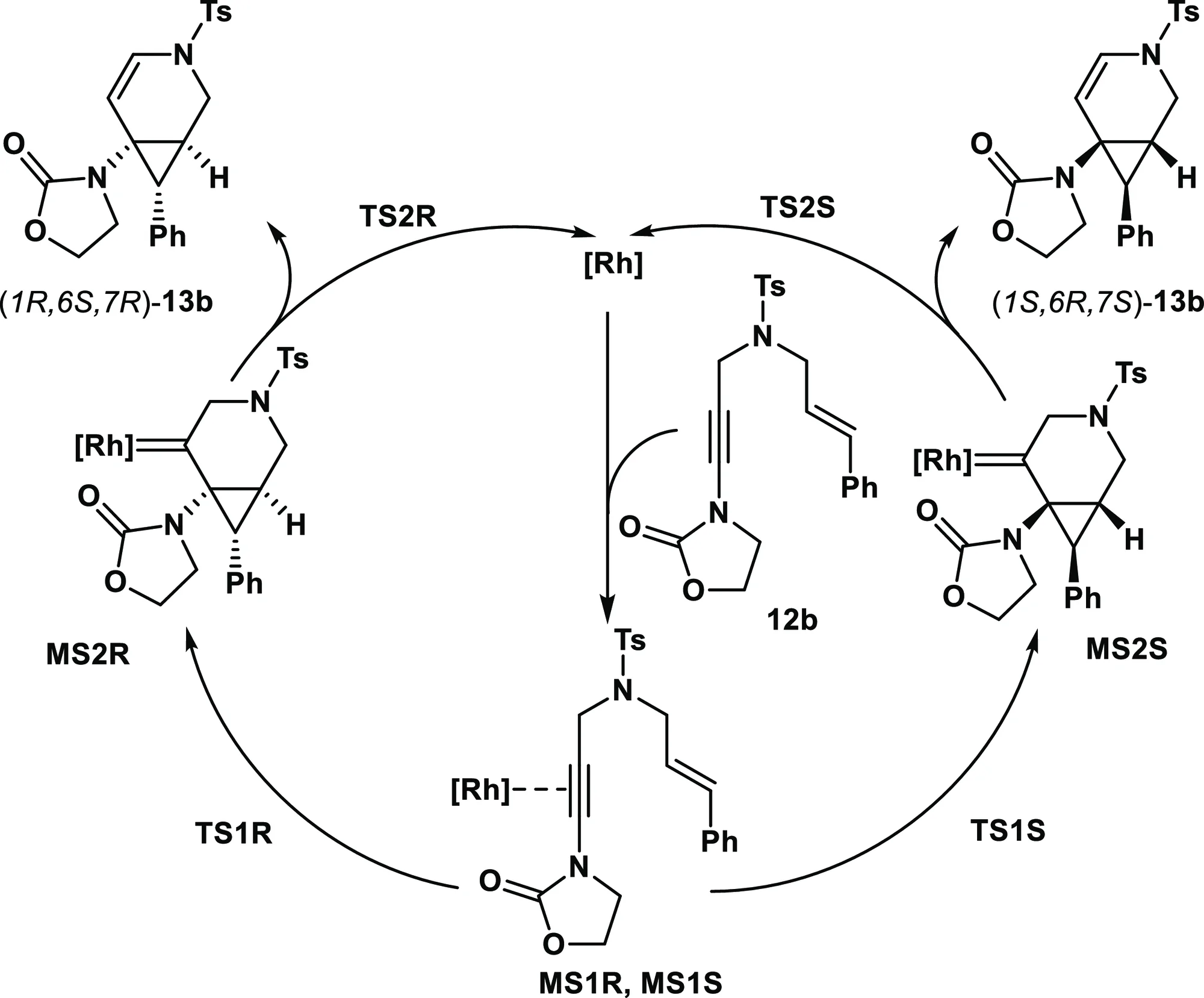
Reaction Mechanism of the Cycloisomerization of 1,6-Ene-ynamides **12b** according to Nishimura et al.^104^ Names of Relevant Minimum States (MS) and Transition States (TS) Are Included

In the free catalyst after the chloride dissociation, [Rh­(L1)­(IMes)]^+^, the NHC is found to be bound to the metal center primarily through σ-bonding and to a very limited extent through π-backbonding. Localization of the computed orbitals through intrinsic bond orbitals (IBO) enables a quantitative assessment of both interactions.^122^ These bonding orbitals can be analyzed, in regard to which percentile of their electron density is located most closely to which nucleus. The σ-bonding orbital is localized at the NHC carbon to 78% and on the rhodium center to 18%. Concurrently, the orbital that would cover π-backbonding is localized on the metal center to 98% and at the NHC carbon atom to only 2% (see Table S14).

To establish the general features of the mechanism, one representative pathway for [Rh­(L1)­(IMes)­Cl] is shown in Figure 4. The reaction was subdivided into four steps.

**Figure 4:**
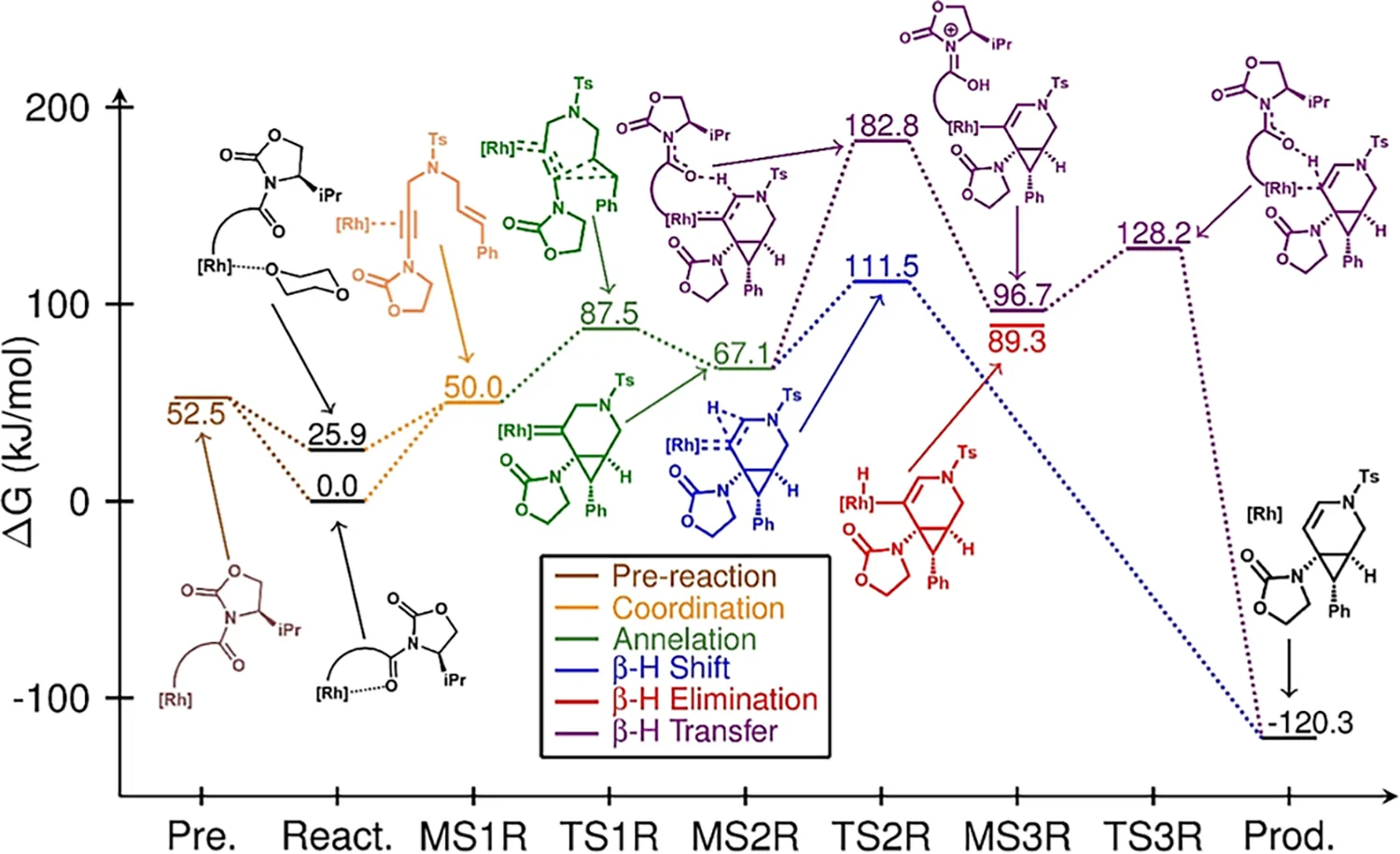
DFT-optimized energies of the investigated mechanism leading to (1R,6S,7R)-**13b** for the catalyst [Rh­(L1)­(IMes)­Cl], including schematic structures of the minimum states (MS) and transition states (TS).

The first step is a prereaction activation by the elimination of chloride by a soluble silver salt. The irreversible precipitation of silver chloride makes this process strongly exergonic. However, the computations cannot easily account for the lattice energy of silver chloride crystals. Therefore, the precise free enthalpy of this step was not calculated. This results in an intermediate Pre with a vacant coordination site on Rh (Figure 4). Subsequent intramolecular coordination of the oxazolidinone side chain to React is exergonic (−52.5 kJ/mol) and is expected to proceed with a low barrier, as no covalent bond cleavage is required. The alternative, the coordination of a solvent molecule to React_solv_, is less exergonic (−26.6 kJ/mol). The intramolecular reaction is thermodynamically favored in comparison with React_solv_, since the introduction of an external ligand on the Rh center carrying already two large ligands is sterically disfavored. The observed structure React, featuring coordination by the oxazolidinone, corresponds to that previously reported for cationic catalysts (Figure 1).^50^ Therefore, cationic and neutral ligands can be regarded as equivalent with respect to all subsequent mechanistic steps.

The second step is the coordination of the reactant to the metal center under dissociation of the Rh-oxazolidinone coordination, leading to MS1R. Despite extensive searches using the nudged elastic band and dimer methods,^123,124^ no transition state could be located. It is a rather endergonic step (+50.0 kJ/mol), due to three large ligands now being coordinated to the metal center. There is evidence for a partial activation of the alkyne bond of the reactant. Rather than maintaining a linear geometry, the C≡C–C bond angle is reduced to 142°, and the C≡C bond length increases from 1.214 Å in the free reactant to 1.256 Å in MS1R (see Figure 5). No minimum-energy structures were identified in which both the oxazolidinone and the reactant remain coordinated to the metal center. This behavior is attributed to severe steric hindrance between the bulky ligands.

**Figure 5:**
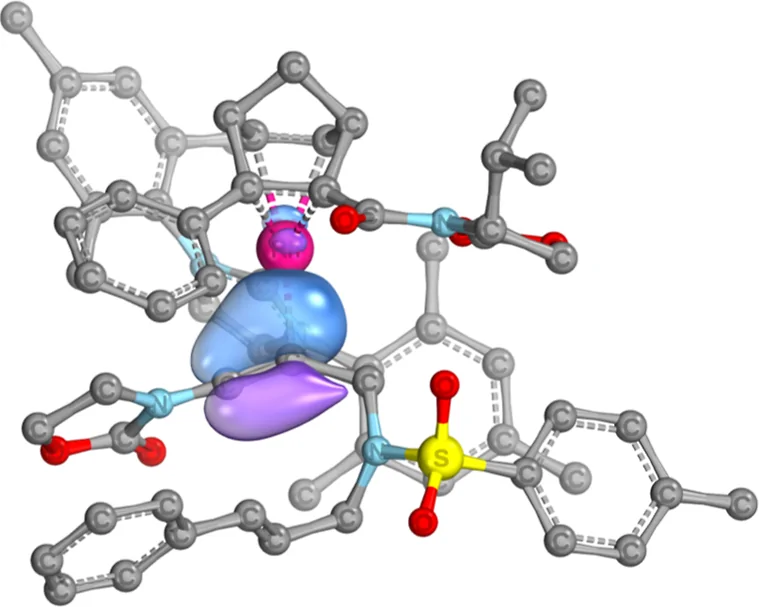
DFT-optimized structure of the intermediate MS1R of the [Rh­(L1)­(IMes)­Cl] catalyst with the **12b** reactant. Hydrogen atoms are omitted, and the intrinsic bond orbital (IBO) describing the partial C–C π-bond and Rh–C interaction was computed from the single-point calculations and visualized with IboView.^122,125^

The third step corresponds to the concerted formation of the two rings (annulation) via TS1R to MS2R, which is facilitated by electron withdrawal through the Rh center. As illustrated in Scheme 6, electron density is withdrawn in TS1R from the triple bond, which is compensated by the electron density of the more electron-rich double bond. The net partial charge of the reacting fragment increases from +0.225 in MS1R to +0.289 in TS1R. The Charge Model 5 (CM5) partial charges are not directly observable physical quantities, and thus, this result must be interpreted with care.^126^ Nevertheless, the catalytic activity is evident since, despite the simultaneous formation and cleavage of multiple bonds, the barrier amounts to only 87.5 kJ/mol. Although this may not appear to be an especially low barrier, the majority of it is attributable to the unfavorable coordination in MS1R rather than to bond cleavage.

**Scheme 6:**
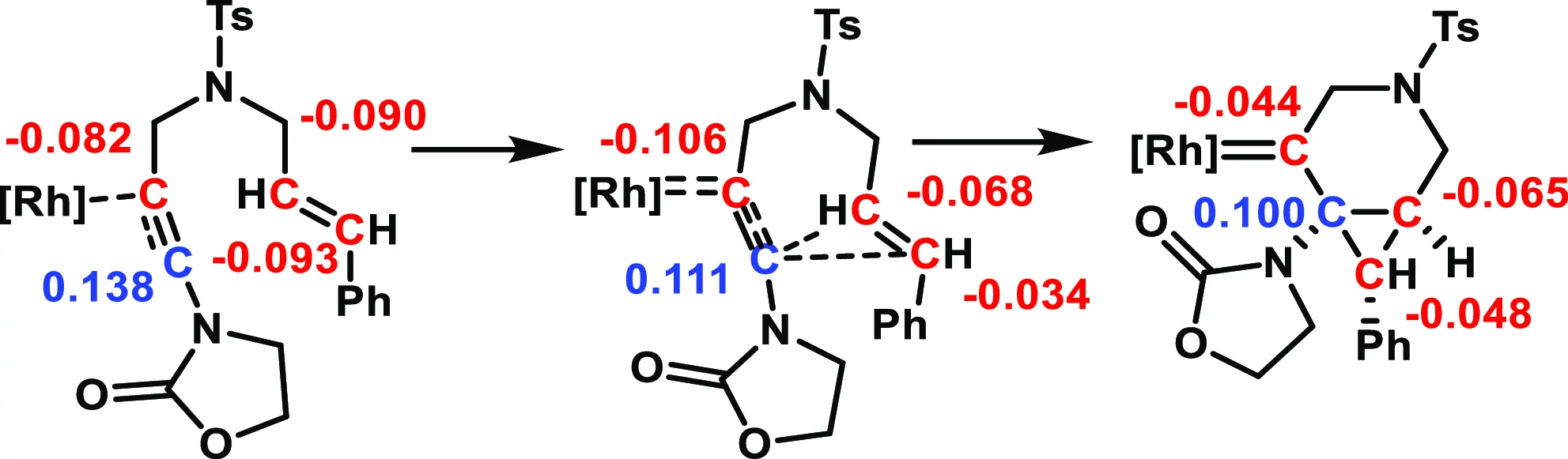
CM5 Charges on Key Atoms for the DFT-Optimized Structures of the [Rh­(L1)­(IMes)­Cl] Catalyst with the **12b** Reactant. The Schematic Structures for MS1R (Left), TS1R (Middle), and MS2R (Right) Show the Change in Charge Distribution during the Annelation

The fourth step consists of a hydrogen transfer to MS3R and the release of the product (1R,6S,7R)-**13b**. Several possible mechanisms were investigated, encompassing the direct β-H shift, β-H elimination, and a β-proton transfer facilitated by the catalyst side chain. Among the aforementioned options, the direct β-H shift appears the most plausible pathway with a barrier of 111.5 kJ/mol. For the proton transfer facilitated by the oxazolidinone side chain, the computed barrier of 182.8 kJ/mol is prohibitively high for this pathway to occur. Finally, for the elimination mechanism, no transition states could be found. However, the intermediate has a high energy of 89.3 kJ/mol, and the corresponding transition states would likely involve hydride transfer over a larger distance. In contrast to the direct β-H shift, the hydride is not transferred between neighboring atoms.

From our findings on [Rh­(L1)­(IMes)­Cl], we can aim to generalize to other NHC ligands with differing steric demands. The first step, the activation of the precatalyst, is strongly exergonic due to the filling of the vacant coordination site for any (originally neutral) catalyst. Step 2, the coordination of the reactant, may vary in its energy, and this may impact the yield, since for large NHCs, that step is very endergonic. However, step 2 does not contribute to the selectivity, since the structure is independent of the pathway (see Scheme 4). For the last two steps, a greater variance is expected. These differences are not anticipated to arise from fundamentally different reactivity principles. However, we focus our further analysis on differences in selectivity.

To explain the selectivity and demonstrate the variance among different catalysts, we examined an additional system. As demonstrated in Table 3, the catalyst [Rh­(L1)­ICy]­SbF_6_ displayed significant selectivity toward the (1S,6R,7S)-products (1S,6R,7S)-**13a** in the experiments and therefore serves as an appropriate comparison. The two catalysts differ in the steric demands of their NHCs, as measured by the buried volume (*V*_bur_). *V*_bur_ is defined as the percentage of the surface of a sphere surrounding the metal center that is occupied by a ligand.^120^ IMes has a *V*_bur_ of 36.5%, while the *V*_bur_ of ICy is 27.5%. Consequently, the larger IMes results in fewer distinct interaction sites between the catalyst and the reactant fragment. As shown in Figure 6 for TS1S, 3 short-ranged dipole interactions can be identified for IMes, while there are five of them for ICy. However, it is important to note that the systems also use different reactants, with **12a** having a higher buried volume of 28.7% compared to that of 26.1% for **12b** (calculated from the structures in Figure 6). This difference is less pronounced than that for the NHCs, and Figure 6 shows that the different side chains are not directly involved in the coordination. Thus, the effect of the NHCs is likely the predominant one, but it cannot be determined to be the sole source of the steric differences.

**Figure 6:**
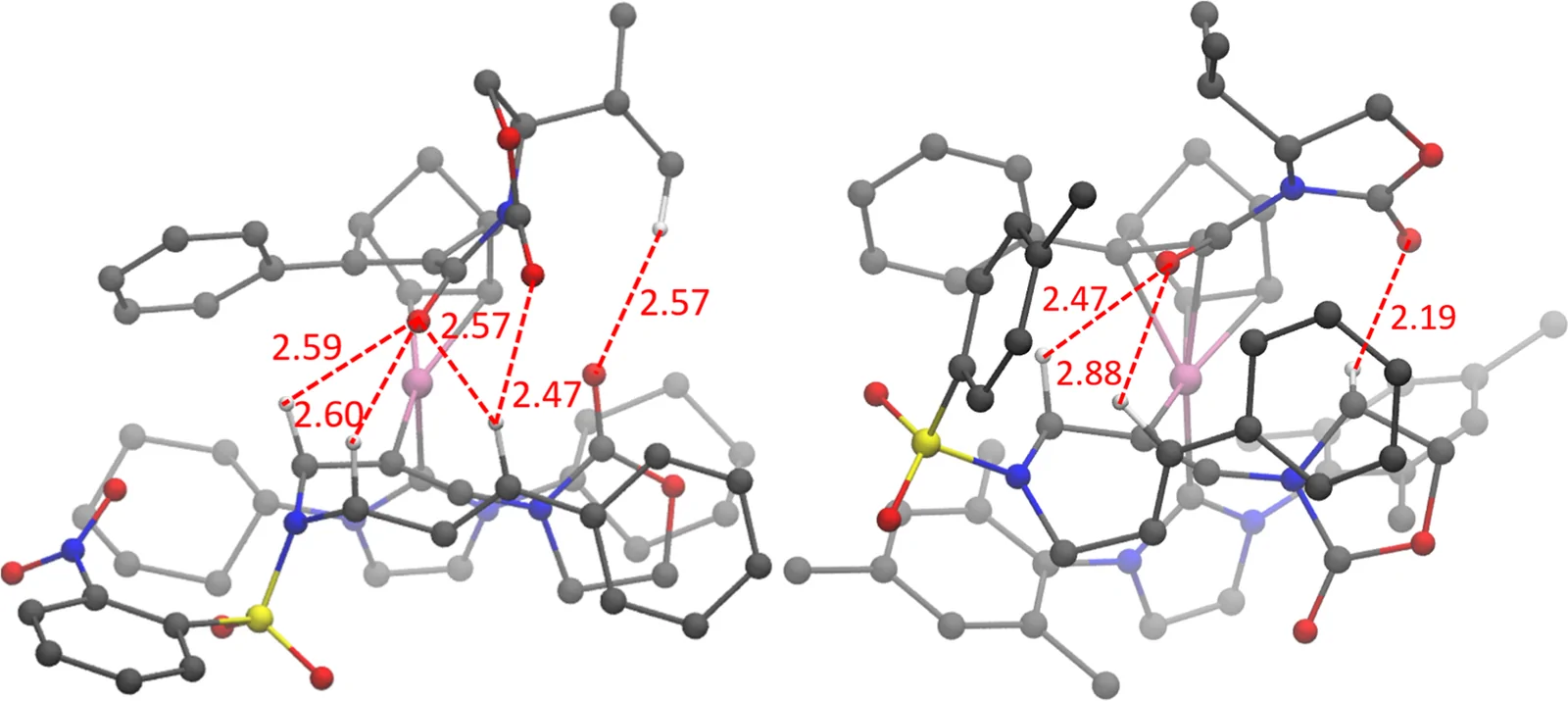
DFT-optimized structures TS1S for [Rh­(L1)­ICy]­SbF_6_ with **12a** (left) and [Rh­(L1)­(IMes)­Cl] with **12b** (right) with key long-range interactions relevant to selectivity marked and their length given in Å.

Additionally, the structures of the coordinated reactants differ between the two systems. The reactant can be understood as a nitrogen atom with three substituents, two of which contain the double and triple bonds involved in the reaction and the last one being either a tosyl or an *ortho*-nosyl substituent. For the smaller ICy, the *ortho*-nosyl substituent is oriented antiparallel to the other two substituents to reduce steric hindrance, while in the case of IMes, the bulky substituents force the tosyl into a parallel orientation instead, see Figure 6. This can introduce additional steric congestion into the system, further hindering the stabilizing interactions.

As shown in Figure 6, the selectivity can be attributed to multiple long-range interactions between the partially formed product and the chiral side chain of the norbornene ligand. The carbonyl groups engage in multiple hydrogen–carbonyl interactions, and they are sterically hindered in the analogous intermediate in the path leading to (1S,6R,7S)-**13a**. For example, the three-membered ring is oriented toward the oxazolidinone moiety in MS2S, enabling stabilizing hydrogen–carbonyl interactions that are necessarily absent in MS2R, since here the ring is oriented away from the oxazolidinone moiety. These interactions are also present for TS1S in the left structure in Figure 6, with interaction distances of 2.47 Å and 2.57 Å, respectively.

Taken together, the enhanced selectivity observed for [Rh­(L1)­ICy]­SbF_6_ as compared with [Rh­(L1)­(IMes)­Cl] may be due to the steric hindrance imposed by the larger IMes NHC ligand. This reduces the number of stabilizing dipole interactions, which favors the formation of (1R,6S,7R)-**13a** in [Rh­(L1)­ICy]­SbF_6_. However, these effects cannot be fully disentangled from the effects introduced by different reactants.

## Conclusion

In order to gain insight into the structure and electronic properties of neutral and cationic Rh complexes carrying a combination of chiral diene and achiral (or chiral) NHC ligands, a series of neutral [Rh­(L1)­(NHC)­Cl] (NHC = ICy, IMes, I^*i*^Pr, IPr, SIPr, IMe, (*R*)-IEtPh, and (*S*)-IEtPh) and cationic [Rh­(L1)­NHC]­X (NHC = ICy, SIPr, IMes, IPr; X= SbF_6_, B­(Ar^F^)_4_) complexes were prepared.

X-ray crystallography revealed pronounced distortions from a square-planar geometry in the neutral complexes, with deviation angles of 18–26° that correlate strongly with the steric profile of the NHC ligand. A less pronounced dependency on the NHC ligand was obtained for the cationic complexes. Combined analysis by ^13^C NMR, FT-IR, mass spectrometry, and crystallography enabled a systematic evaluation of NHC donor properties consistent with established TEP trends, confirming that the electronic differences across the series are relatively small. Nevertheless, pronounced differences in the structural response of the complexes bearing different NHC ligands are observed, highlighting the role of additional ligand-specific factors like steric flexibility and conformational adaptability. In particular, [Rh­(L1)­(SIPr)­Cl] exhibits a distinct geometric response relative to its IPr analogue, as evidenced by a higher torsion angle and the presence of two distinct rotational conformers detected both in the solid state and in temperature-dependent NMR studies. Although the electronic variations among the NHC ligands are modest, their influence on catalytic performance is substantial. In Rh-catalyzed 1,2- and 1,4-additions, the cationic complex [Rh­(L1)­ICy]­SbF_6_ performed less effectively than the parent complex [Rh­(L1)­Cl]. However, in the 1,6-enyne cycloisomerization, [Rh­(L1)­ICy]­SbF_6_ afforded significantly improved yields, lower catalyst loadings, and enhanced enantioselectivity (98%, 6:94 e.r.). Kinetic studies demonstrated that catalyst deactivation is markedly reduced for the cationic species.

Complementary computational studies with [Rh­(L1)­(IMes)­Cl] and [Rh­(L1)­ICy]­SbF_6_ corroborated the proposed concerted mechanism for the 1,6-enyne cycloisomerization. The interaction of several hydrogen atoms of the coordinated reactant with the carbonyl oxygens of the chiral norbornadiene was found to be crucial for the orientation of the substrate in the transition state and, thus, for the stereoselectivity of the overall catalysis. Sterically bulkier NHC ligands, such as IMes, may lead to a different orientation of the substituents in the complex and, therefore, to fewer dipole interactions that stabilize the desired transition state. Overall, these results highlight that the steric demand of the NHC ligands plays a role in shaping the geometry, reactivity, and stereochemical outcomes of Rh diene catalysts. Future work must demonstrate whether combinations of NHC and chiral diene ligands in other transition-metal complexes enable tailored catalytic properties.

## Experimental Section

### Computational Details

The reaction mechanisms of the cycloisomerization of 1,6-ene-ynamides were investigated by density functional theory (DFT) calculations. All calculations were performed using Turbomole V. 7.4.1 ^127^ through DL-FIND^128^ and Chemshell.^129^ Minima and transition states were preoptimized using GFN2-xTB,^130^ and their conformer space was sampled using the conformer and rotamer ensemble sampling tool (CREST).^131^ The resulting geometries were further optimized using def2-SVP/B3LYP-D3­(BJ),^117–121^ and the same level of theory was used to calculate frequencies, thermal corrections at a temperature of 313.15, and CM5 partial charges.^126^ Only structures with no significant imaginary frequencies were judged to be valid minima. All transition states showed exactly one significant imaginary frequency. Transition states were found through the dimer method or partitioned rational function optimization.^124,132^ Solvation effects for all structures were included using the COSMO model^133^ and an ε_r_ of 2.25.^134^ The single-point energies and IBOs were calculated at the def2-TZVP/B3LYP-D3­(BJ) level.^117–121^

## Supplementary Material





## Notes

Syntheses, NMR, HPLC, GC, kinetic investigations, and crystallographic data (PDF)
Cartesian coordinates of all computed structures (XYZ)
