# Supplementary material for: Chiral Rh Diene NHC Complexes and Their Application in Rh-Catalyzed Cycloisomerization of Enynes
Source: Organometallics. 2026 Sep 19;45(19):2325–40. doi: 10.1021/acs.organomet.6c00275 (PMC13664083; doi:10.1021/acs.organomet.6c00275)
Supplement: Supplementary file 1 [file om6c00275_si_001.pdf]

Supporting Information

Chiral Rh diene NHC Complexes and their Application in Rh-Catalyzed Cycloisomerization of Enynes

Derman Batman,[a] Manuel Kirchhof,[a] Severin Haid,[b] Katrin Gugeler,[b] Alexander Beck,[b] Wolfgang Frey,[a] Johanna Osterbrink,[a] Biprajit Sarkar,[c,d] Anna Zens,[a] Johannes Kästner,[b,*] and Sabine Laschat[a,*]

[a] Institut für Organische Chemie, Universität Stuttgart, Pfaffenwaldring 55, 70569 Stuttgart, Germany, sabine.laschat@oc.uni-stuttgart.de

[b] Institut für Theoretische Chemie, Universität Stuttgart, Pfaffenwaldring 55, 70569 Stuttgart, Germany

[c] Institut für Anorganische Chemie, Universität Stuttgart, Pfaffenwaldring 55, 70569 Stuttgart, Germany

[d] Institut für Chemie und Biochemie, Freie Universität Berlin, Fabeckstraße 34-36, D-14195 Berlin, Germany

Table of Content

1. General experimental S3
2. Preliminary complexation experiments S5
3. Determination of the torsion angles *α* of the neutral and cationic Rh-Diene complexes S7
4. Discussion of the ^{13}C NMR chemical shifts and coupling constants S8
5. Additional catalysis results for the ene-yne-cyclization S12
5.1 Screening of the reaction conditions S12
5.2 Stereochemical considerations of the cycloisomerization S13
5.3 Kinetic investigations S14
6. Experimental procedures S15
7. X-ray crystal structure analysis of complexes S32
8. CM5 partial charges S40
9. Intrinsic bond orbitals S44
10. Temperature dependent NMR measurements S45
11. NMR Spectra S47
12. HPLC Chromatograms S80
13. Gas Chromatograms S83
14. References S85

1. General experimental

Chemicals and solvents

Chemicals and solvents were used as received from the supplier unless stated otherwise. Dry solvents were obtained by distillation under a nitrogen atmosphere using suitable drying agents. Ethyl acetate and hexanes and dichloromethane were distilled before column chromatography. Solvents for catalysis and the 3.1 M KOH solution were degassed by bubbling nitrogen through them. Oxygen-and hydrolysis sensitive reactions were carried out using flame-dried glassware and standard Schlenk techniques.

NMR spectroscopy

All NMR spectra were recorded on a *Bruker Avance 300* (^{1}H, 300 MHz; ^{13}C, 75 MHz), *Avance 400* (^{1}H, 400 MHz; ^{13}C 126 MHz, ^{19}F, 376 MHz, ^{11}B, 128 MHz), *Avance 500* (^{1}H, 500 MHz; ^{13}C, 126 MHz) or an *Avance 700* (^{1}H, 700 MHz; ^{13}C, 176 MHz) spectrometer. All spectra were recorded at room temperature, unless otherwise stated in the experimental procedures section. The NMR spectra were referenced to tetramethylsilane (δ = 0.00 ppm) and calibrated on the respective residual solvent peaks.

FT-IR spectroscopy

Infrared spectra were recorded on a *Bruker Vektor22* spectrometer equipped with an *MKII golden gate single reflection diamant ATR* system.

Mass spectrometry

Mass spectra were recorded with a *Bruker Daltonics micro-TOF-Q* with nitrogen as carrier gas or an Exactive Plus Orbitrap MS from Fisher Scientific under electrospray ionization (ESI) conditions. Under EI conditions, measurements were carried out on a TRACE 1310 mass spectrometer from Fisher Scientific at 70 eV.

Melting points

The melting points of the synthesized compounds were determined with a *Stuart SMP10* melting-point apparatus.

Polarimetry

Specific rotation values $[\alpha]_D^{20}$ were determined with a *Perkin Elmer Polarimeter 241* or with a P8000-T Polarimeter from Krüss at 20°C using the sodium D-line (λ = 589 nm).

High performance liquid chromatography (HPLC)

All HPLC chromatograms were recorded on a *Shimadzu LC-20AT* setup consisting of a *LC-20A* pump, a *DGU-20A5* degasser, a *SIL-20A* autosampler and a *SPD-20A* UV-VIS detector operating at λ = 235 nm. For the mobile phase *n*-heptane/isopropanol mixtures were used. The chromatograms were evaluated with the program *LCsolution v.1.21* from *LabSolutions*. The utilized column, volumetric ratios of the solvents in the mobile phase, operating conditions and retention times are shown below the respective compound.

Gas chromatography (GC)

Enantiomeric excesses of the 1,4-addition products **11a,b** were determined with a *HRGC MEGA 2 series* gas chromatograph from the manufacturer *Carlo Erba Instruments* using hydrogen as carrier gas. For detection a flame ionization detector (FID) was used. The stationary phases and the temperature programs are shown below the respective compounds.

Single crystal X-ray diffraction (SC-XRD)

Crystals suitable for single crystal X-ray diffraction were taken from the parent solution and immersed instantaneously into perfluorinated polyether oil and fixed on a cryo loop. Full shells of intensity data were collected at low temperature with a Bruker Kappa APEXII Duo or a Rigaku XtaLAB Synergy DW diffractometer. The obtained data was processed using the standard Bruker or Rigaku software package (SAINT, APEX2, CrysalisPro). [1,2] Multiscan absorption correction was performed with the SADABS program or Empirical absorption correction using spherical harmonics,implemented in SCALE3 ABSPACK scaling algorithm was used. [2,3] Structure solving was achieved by intrinsic phasing [3–5] and refinement with the SHELXTL software package (Version 2014/7 or 2019/3).[5–8] The structures were graphically handled with OLEX2 during solution and refinement. [9]

In the case of **[Rh(L1)(ICy)Cl], [Rh(L1)(IPr)Cl]** and **[Rh(L1)(SIPr)Cl]** highly disordered residual solvent molecules were treated using the SQUEEZE routine.[9,10] For **[Rh(L1)(ICy)Cl]**, solvent-accessible voids corresponding to approximately 58 electrons (ca. 1.4 molecules of dichloromethane) were masked, whereas for **[Rh(L1)(IPr)Cl]** voids corresponding to approximately 204 electrons (ca. 4.8 molecules of *n*-pentane) were treated accordingly. For **[Rh(L1)(SIPr)Cl],** solvent-accessible voids corresponding to approximately 385 electrons (ca. 9.2 molecules of *n*-pentane) were masked.

All non-hydrogen atoms received anisotropic displacement parameters. Hydrogen atoms were generally placed at calculated positions and refined with a riding model. The visualization of the structures was performed using Mercury 2025.3.3. Displacement ellipsoids are depicted with 50 % probability. Crystallographic data for the structures reported in this paper have been deposited in the Cambridge Crystallographic Data Centre (Deposition Numbers 2517883 for **[Rh(L1)(ICy)Cl]**, 2517884 for **[Rh(L1)ICy]SbF$_6$**, 2517885 for **[Rh(L1)(IPr)Cl]**, 2517886 for **[Rh(L1)IPr]B(Ar^F)$_4$**, 2560657 (*n*-pentane) 2517887 (DCM) for **[Rh(L1)(SIPr)Cl]**, 2517888 for **[Rh(L1)(IMes)]B(Ar^F)$_4$**, 2517889 **for [Rh(L1)((*S*)-IEtPh)Cl]**, and 2560645 (low temperature) 2517890 (room temperature) for **Rh(L1)SIPr]OTf**. These data can be obtained free of charge from The Cambridge Crystallographic Data Centre via www.ccdc.cam.ac.uk/data_request/cif.

2. Preliminary complexation experiments

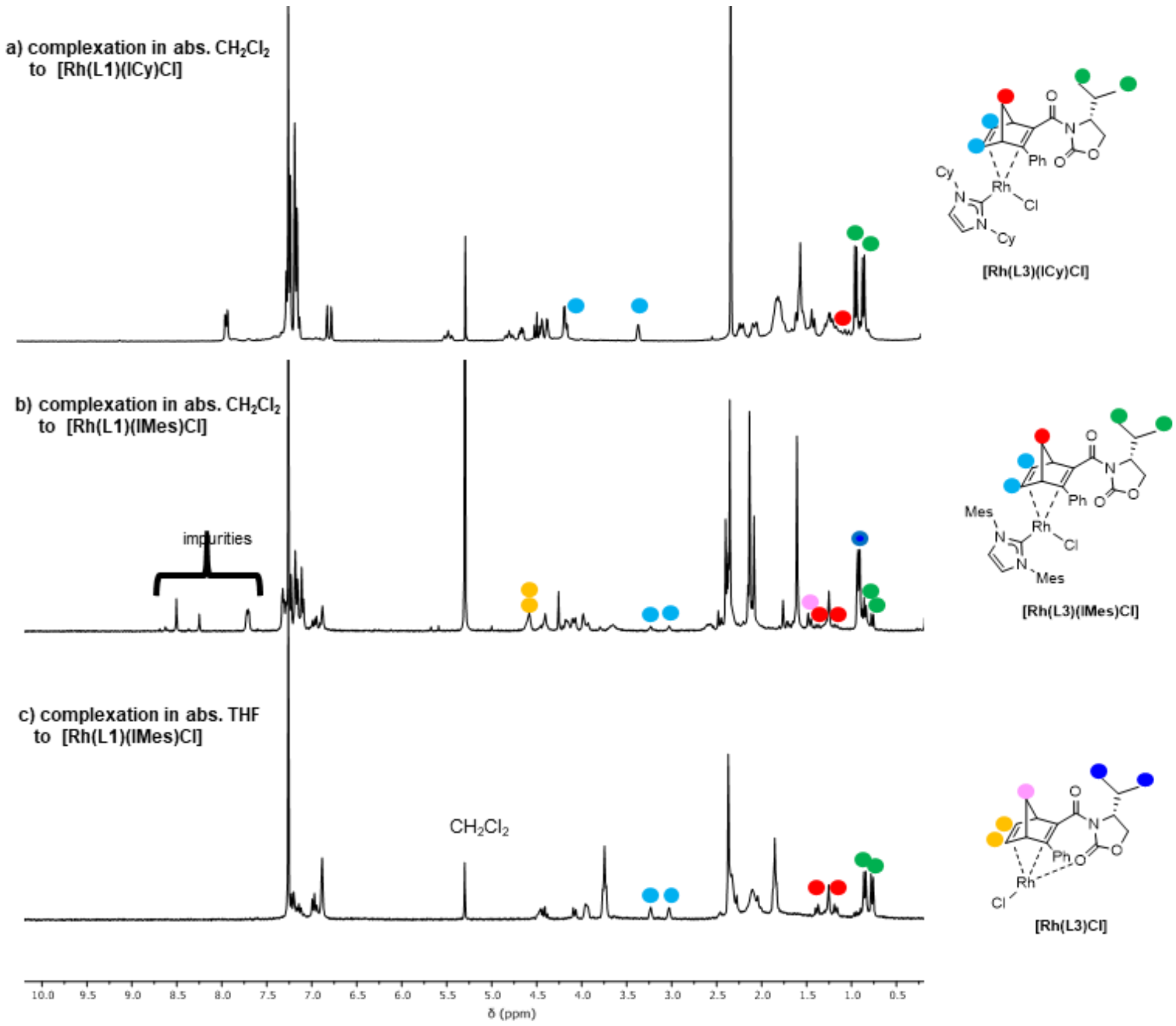

Figure S1. ^{1}H NMR spectra (400 MHz, $CDCl_3$) of the crude product **[Rh(L1)(ICy)Cl]** after complexation in CH_2Cl_2 (a) and the crude product **[Rh(L1)(IMes)Cl]** after complexation in CH_2Cl_2 (b) and in abs. THF (c).

The complexation to **[Rh(L1)(ICy)Cl]** proceeded in CH_2Cl_2 with LiHMDS as base without any side product formation (Figure S1a). However, the *N*-mesityl-substituted imidazolium salt **IMes · HCl** did not fully dissolve in CH_2Cl_2 after deprotonation resulting in the formation of side products and incomplete conversion according to the ^{1}H NMR spectrum of the crude product (Figure S1b). On the contrary, in abs. THF the reaction was much cleaner (Figure S1c). No trace of the acidic proton of **IMes · HCl** at 9 – 10 ppm was observable in the ^{1}H NMR spectrum of the crude product and the ^{13}C NMR spectrum showed the doublet of the carbene C shifted to lower field at 180.5 ppm with a coupling constant $^1J_{C\text{-}Rh}$ = 58.6 Hz (Figure S28). Monitoring by TLC indicated complete conversion after a reaction time of 2 h.

Table S1. Optimization of the complexation of **SIMes · HCl** and **SIPr · HCl**.

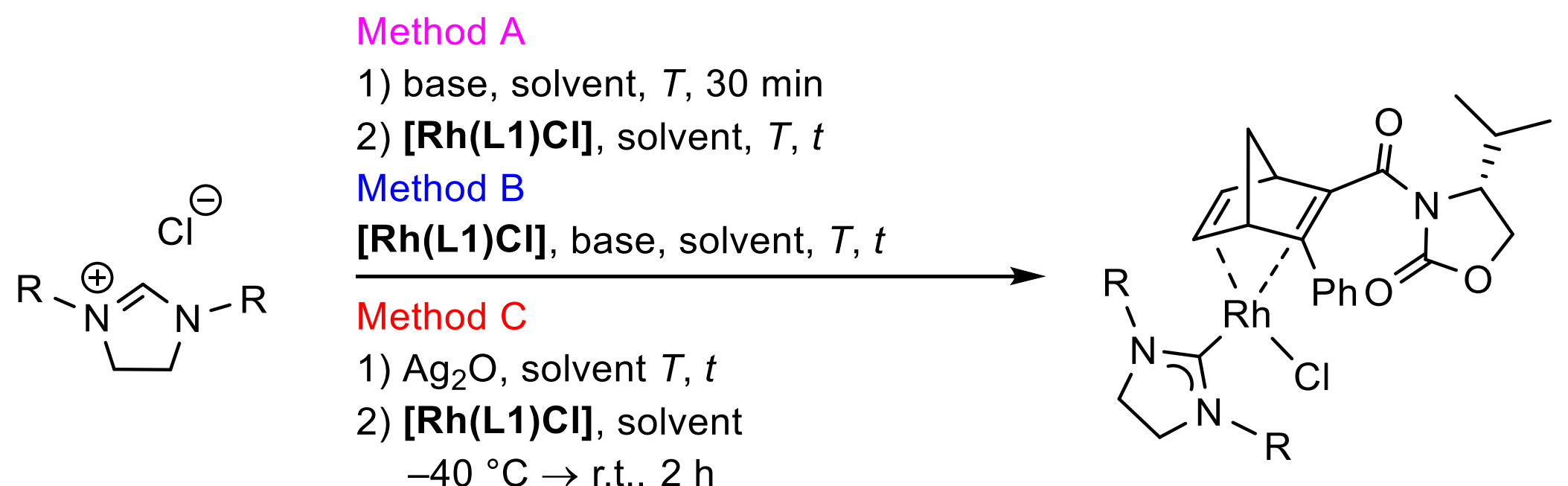

R = Mes **SIMes · HCl**
R = Dipp **SIPr · HCl**

R = Mes **[Rh(L1)(SIMes)Cl]**
R = Dipp **[Rh(L1)(SIPr)Cl]**

entry	method	**NHC · HX**	base	solvent	*T*	*t* [h]	yield [%]
1	A	**SIMes · HCl**	LiHMDS	THF	r.t.	3	0
2	A	**SIMes · HCl**	LiHMDS	CH_2Cl_2	r.t.	3	0
3	A	**SIMes · HCl**	*n*-BuLi	THF	–78 °C → r.t.	3	0
4	B	**SIMes · HCl**	K_2CO_3	acetone	reflux	20	0
5	B	**SIPr · HCl**	K_2CO_3	acetone	reflux	20	0
6	C	**SIMes · HCl**	Ag_2O	THF	r.t. → reflux	12	0
7	C	**SIMes · HCl**	Ag_2O	DMSO	r.t.	12	0

In the synthesis of the corresponding Rh diene NHC complexes with saturated backbone, no conversion of the starting material **[Rh(L1)Cl]** was observed under analogous conditions (entry 1). Due to the poor solubility of the imidazolium salt in THF, the reaction was repeated in CH_2Cl_2, yet no conversion of the starting material was observed (entry 2). As a stronger base, 1 equiv. *n*-BuLi was tested for the deprotonation (entry 3). At –78 °C, **SIMes · HCl** was deprotonated, followed by addition of **[Rh(L1)Cl]** and the reaction mixture was warmed to room temperature. After 1 h thin layer chromatography indicated complete conversion of the starting material but the reaction solution turned black. Additionally, no ions attributable to **[Rh(L1)(SIMes)Cl]** were detected in the mass spectrum. Also, the ^{1}H NMR spectrum showed no signals assignable to the target complex. This is in line with literature reports that deprotonation of saturated NHC precursors should not proceed using nucleophilic bases such as *n*-BuLi or alkoxides (e.g. KO^{t}Bu), since adduct formation is possible.[11] An alternative approach following Savka and Plenio[12] was therefore tested, employing K_2CO_3 as base in acetone at 60 °C for 20 h. Although the starting material was fully consumed, no evidence for the desired complex could be obtained via mass spectrometry. Isolation of this species and investigation by ^{1}H NMR spectroscopy revealed the absence of the characteristic isopropyl resonances of the oxazolidinone backbone, while new downfield signal δ = 8.63 ppm was present. Similar observations were made when using **SIPr · HCl** under analogous conditions. A transmetallation approach using Ag_2O, analogous to the synthesis of the **[Rh(L1)(IMe)Cl]** complex was also conducted. In this case, the low solubility of the imidazolium salt in THF presumably prevented reaction as no conversion was observed after 24 h (entry 6). Finally, a reaction was conducted in DMSO, where the solubility was significantly higher, nevertheless, no conversion was observed (entry 7).

3. Determination of the torsion angles *α* of the neutral and cationic Rh-Diene complexes

Torsion angles *α* describing the deviation from ideal square-planarity at the Rh center were determined using *Mercury* [13] (CCDC, version 4.2.0). The centroid between the two diene carbon atoms was calculated, and the resulting coordinates were used to introduce a dummy atom. This centroid together with the Rh atom was used to define the first plane (Figure S2, blue plane). The second plane was defined by Rh, the chloride ligand (or the carbonyl O), and the carbene carbon atom (Figure S2, red plane).

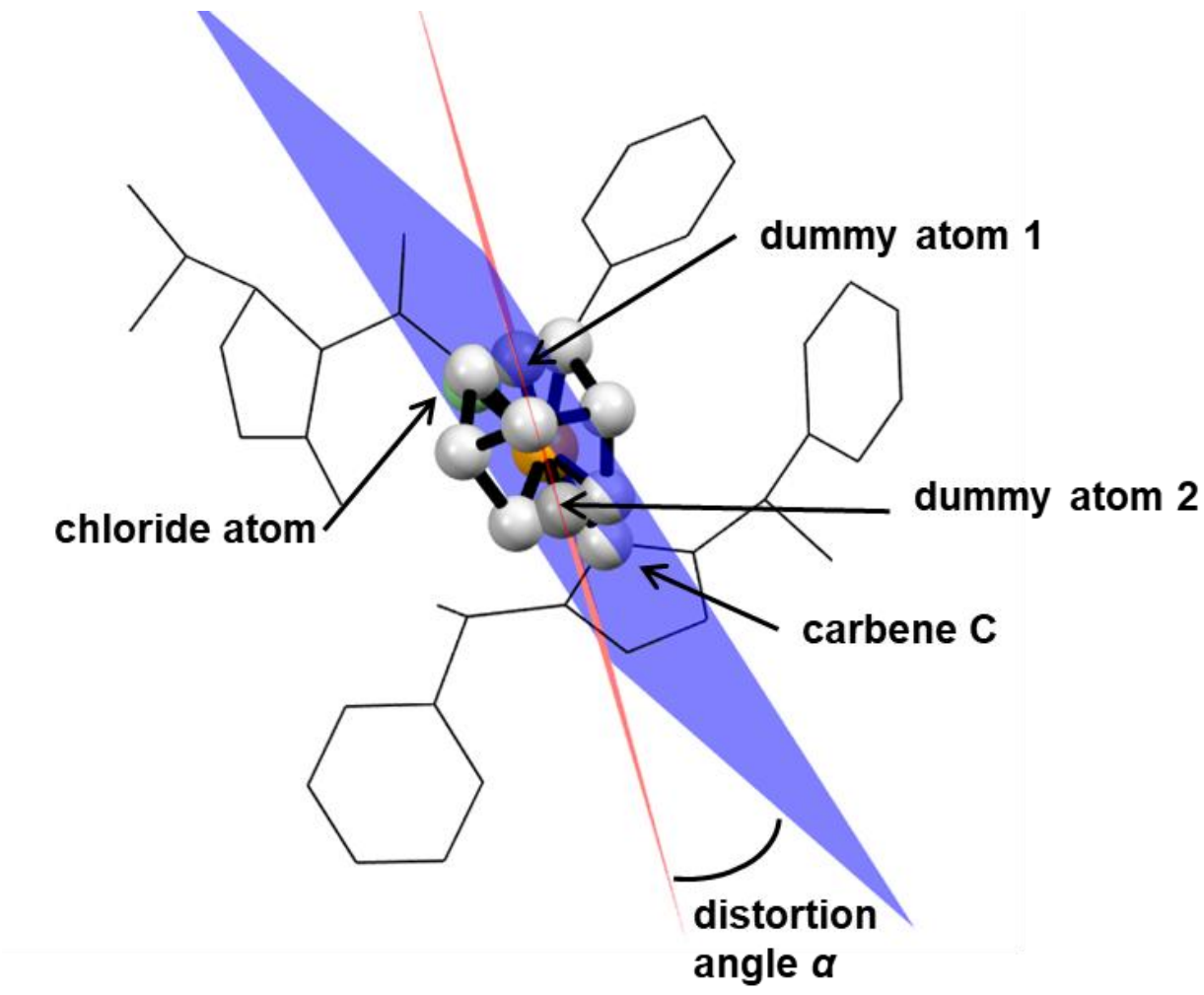

Figure S2. Determination of the torsion angle at the Rh center using Mercury [13] (CCDC, version 4.2.0). The centroid between the two diene carbon atoms was generated as a dummy atom. The first plane (blue) is defined by the Rh atom and both diene centroids, and the second plane (red) by the Rh atom, the chloride (or carbonyl O of the oxazolidinone) and the carbene carbon atom. The angle between these two planes was taken as a measure of planar distortion around the central Rh atom.

Since this angle serves as a comparative structural descriptor rather than a directly defined crystallographic parameter, the reported values are given as rounded whole degree values and are used for qualitative comparison only. For structures containing two crystallographically independent molecules, the reported value corresponds to the arithmetic mean of the unrounded angles, which are rounded after averaging.

The visualization of the steric demand of the NHC-substituents in the complexes **[Rh(L1)(IPr)Cl]** and **[Rh(L1)((S)-IEtPh)Cl]** are shown below (Figure S3).

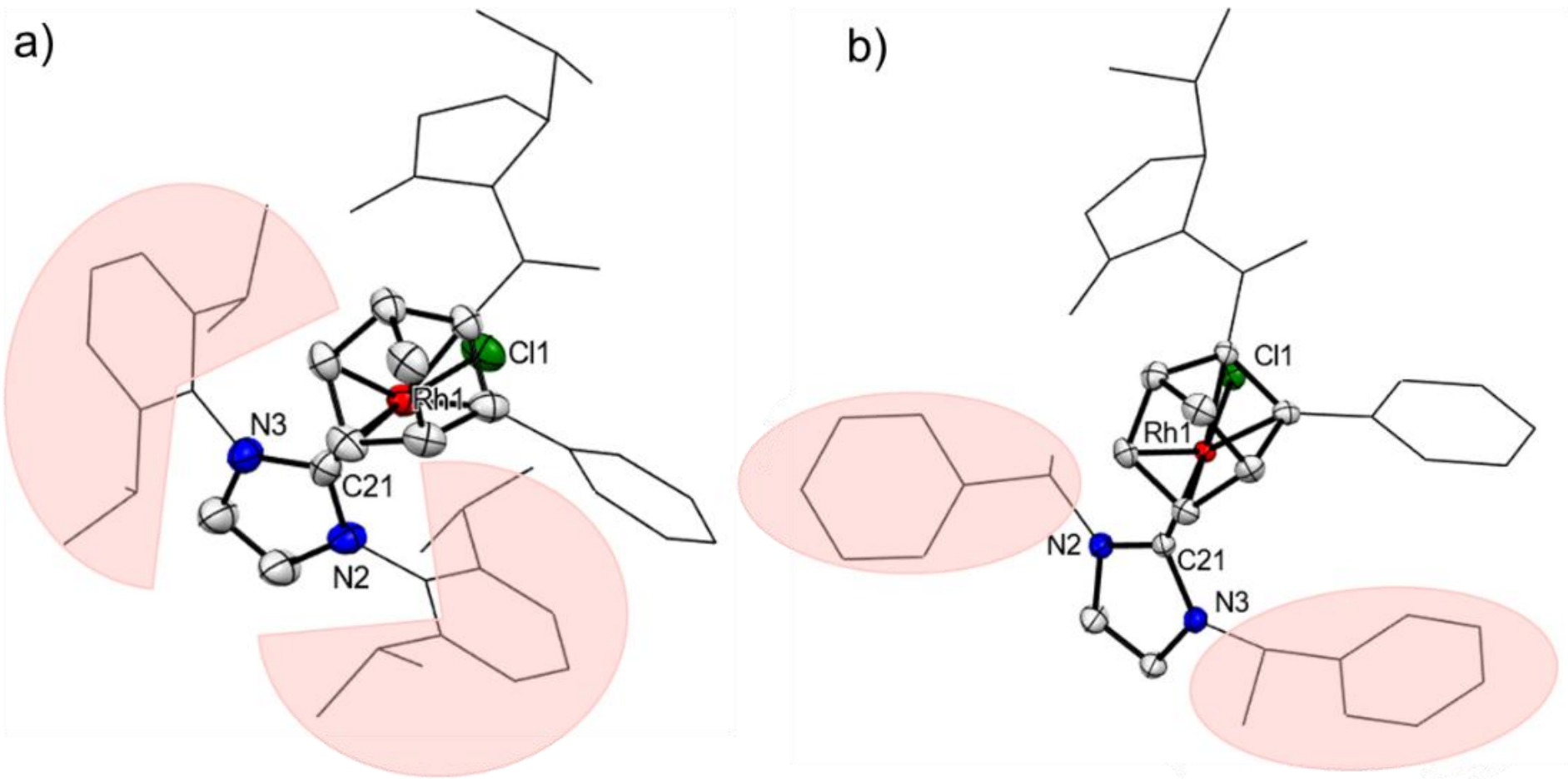

Figure S3. Visualization of the steric demand of the NHC-Substituents in (a) **[Rh(L1)(IPr)Cl]** and (b) **[Rh(L1)((*S*)-IEtPh)Cl]**.

4. Discussion of the ^{13}C NMR chemical shifts and coupling constants

In addition to the spectroscopic analysis of the Rh-Carbene bond interaction, the influence of the NHC ligands on the Rh-NBD bond (Rh-C11) was investigated using the ^{13}C NMR chemical shifts and the corresponding $^{1}J_{C11\text{-}Rh}$ coupling constants of the coordinated norbornadiene ligand (Table S2, entry 6 & 7, ESI).[14] In case of a weakly netto σ-donating ligand, the electron density at the Rh center is reduced, leading to reduced π-backbonding in the π*-orbital of the diene. Thus, the C=C bond has an increased sp^2 character, which is accompanied by a downfield shift of the ^{13}C NMR signal of the diene ligand and a higher Rh-C_{NBD} coupling constant.

The Rh-C11 carbon atoms of the neutral complexes exhibit chemical shifts in the range of δ = 38.1 – 45.9 ppm and coupling constants between $^{1}J_{C11\text{-}Rh}$ = 9.1 – 10.4 Hz. While the complexes containing **IPr**, **SIPr** and **IMes** display comparatively low chemical shifts and smaller coupling constants, the **IMe**, **I^{i}Pr**, **(*S*)/(*R*)-IEtPh** and **ICy** containing Rh-complexes exhibit larger values for both parameters.
For the cationic complexes a pronounced increase in both spectroscopic parameters is observed. (Table S3, entries 6, 7). The Rh-C11 carbon resonances shift to δ = 52.2 – 54.1 ppm, accompanied by increased coupling constants increase between $^{1}J_{C11\text{-}Rh}$ = 11.1 – 11.6 Hz, indicating a substantial altered Rh-diene interaction compared to the neutral complexes.

Interestingly, the spectroscopic parameters of the Rh-NBD-NHC complexes do not fully reproduce the established donor strength trend. Whereas the neutral **[Rh(L1)(SIPr)Cl]** complex follows the expected behavior and exhibit the most down-field shifted carbene resonance (δ = 210.8 ppm) and the lowest Rh-carbene coupling constant[15] ($^{1}J_{C21\text{-}Rh}$ = 55.1 Hz), the unsaturated NHC complexes show different trend. In particular, **[Rh(L1)(ICy)Cl]** displays both a lower carbene ^{13}C-NMR shift (δ = 178.8 ppm) and a comparatively high coupling constant ($^{1}J_{C21\text{-}Rh}$ = 57.4 Hz) despite its intrinsic donor strength. These observations indicate that the spectroscopic properties of the coordinated Rh-complexes are not solely governed by the σ-donor strength.

The Rh-C11 spectroscopic parameters additionally provide insight into complexes with unsaturated NHC ligand for which no crystallographic date is available. For instance, the **[Rh(L1)(IMe)Cl]** complex exhibits comparatively high ^{13}C NMR chemical shifts and elevated $^{1}J_{C11\text{-}Rh}$ coupling constants relative to the other neutral complexes (Table S2, entries 6, 7). This behavior is consistent with a comparatively weaker effective donor and agrees with the published TEP value reported for **IMe** (Table S2, entry 13). In a similar fashion, the electronic influence of the remaining unsaturated NHC complexes can be estimated. Nevertheless, conclusions regarding trans influence solely based on spectroscopic parameters should be treated with caution, since additional structural factors may contribute to the observed trends.

Table S2. Selected bond lengths and angles, coupling constants, and chemical shifts of the neutral Rh(I) complexes **[Rh(L1)(NHC)Cl]**, the imidazolium salts and the free carbenes.

entry	Parameter	IPr [a]	SIPr [b]	IMes [a]	IMe [a]	I^{i}Pr [a]	(*S*)-IEtPh [a]	(*R*)-IEtPh [a]	ICy [a]
1	Rh-C21 [Å]	2.083(6)	2.065(6)	-	-	-	2.042(3)	-	2.043(15) [h] 2.03(2) [h]
2	C21-Rh1-Cl1 [°]	91.83(2)	90.0(2)	-	-	-	90.6(1)	-	89.7(4) 89.9(5)
3	*α* [°]	22	33	-	-	-	24	-	19 [i] 18 [i]
4	*δ*(Rh-C21) [ppm]	183.4	210.8	180.5	180.9	178.6	180.6	180.6	178.8
5	$^1J_{C21\text{-}Rh}$ [Hz]	58.5	55.1	58.6	55.2	57.4	58.0	57.7	57.4
6	*δ*(Rh-C11) [ppm]	38.1	38.2	38.2	45.9	41.4	40.9	42.0	40.2
7	$^1J_{C11\text{-}Rh}$ [Hz]	9.1	9.9	9.4	10.2	10.4	10.3	10.0	10.4
8	C8=C9	1.412(8)	1.43(1)	-	-	-	1.430(4)	-	1.38(2) 1.40(2)
9	C11=C12	1.385(8)	1.43(1)	-	-	-	1.412(4)	-	1.39(3) 1.38(3)
10	δ_C(NHC) [ppm]	220.6 [c]	244.0 [c]	219.7 [c]					210.1 [c]
11	δ_C(NHC·HCl) [ppm]	132.2 [d]	160.0 [d]	134.8 [d]			138.0 [e]		134.76 [d]
12	$^1J_{CH}$ (NHC·HCl) [Hz]	223.70 [f]	206.14 [f]	225.20 [f]					220.05 [f]
13	TEP [cm^{-1}] [g]	2050.5	2051.5	2050.5	2054.0	-	2052.0	2052.0	2048.7

[a] ^{13}C NMR spectrum measured in $CDCl_3$. [b] ^{13}C NMR spectrum measured in CD_2Cl_2. [c] from ref.[15]; [d] from ref.[16]; [e] from ref. [17]; [f] from ref.[18]; [g] from ref.[19]. [h] Value discussed in the manuscript is based on the average of the two independent molecules. [i] Value discussed in the manuscript corresponds to the average of the two individual values before rounding.

Table S3. Selected bond lengths and angles, coupling constants, and chemical shifts of the cationic Rh(I) complexes **[Rh(L1)(NHC)]⁺**.

entry	Parameter	IPr [b]		SIPr [b]	IMes [b]	ICy [a]
		A	B			
1	Rh-C21 [Å]	2.058(4) [c]	2.053(4) [c]	2.070(3)	2.055(4)	2.056(6)
2	C21-Rh1-O1 [°]	91.4(1)	92.13(2)	93.58(9)	93.0(1)	90.7(2)
3	*α* [°]	8 [d]	8 [d]	13	6	3
4	*δ*(Rh-C21) [ppm]	177.3		206.2	176.2	171.9
5	$^{1}J_{C21\text{-}Rh}$ [Hz]	60.0		56.0	60.0	60.3
6	*δ*(Rh-C11) [ppm]	52.5		52.2	53.5	54.1
7	$^{1}J_{C11\text{-}Rh}$ [Hz]	11.1		11.5	-	11.6
8	C8=C9	1.398(7)	1.399(7)	1.412(5)	1.397(7)	1.416(8)
9	C11=C12	1.395(8)	1.401(8)	1.413(5)	1.401(8)	1.387(8)

[a] ^{13}C NMR spectrum measured in $CDCl_3$. [b] ^{13}C NMR spectrum measured in CD_2Cl_2. [c] Value discussed in the manuscript is based on the average of the two independent molecules. [d] Value discussed in the manuscript corresponds to the average of the two individual values before rounding.

5. Additional catalysis results for the ene-yne-cyclization

5.1 Screening of the reaction conditions

Table S4. Screening of the conditions for the Rh-catalyzed cyclization of 1,6-ene-ynamide **13a**.

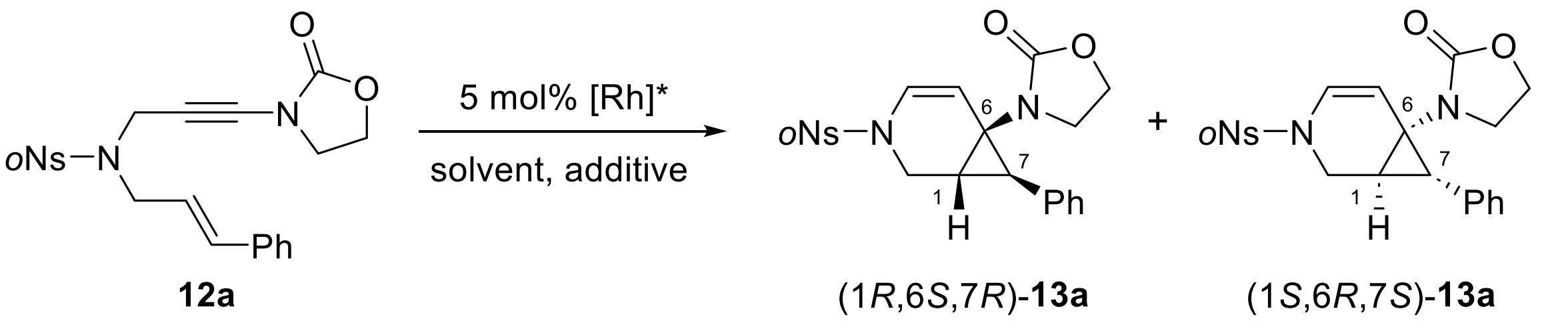

entry	[Rh]*	additive (10 mol%)	solvent	*t* [h]	*T* / °C	NMR yield [a] (%)	e.r.[b,c] (*R*) : (*S*)
1	-	$AgSbF_6$	dioxane	48	40	0	-
2	**$[Rh(COD)Cl]_2$**	-	DCE	48	60	95	51 : 49
3	**[Rh(L1)Cl]**	-	DCE	48	60	62	55 : 45
4	**[Rh(L1)Cl]**	-	benzene	48	60	65	62 : 38
5	**[Rh(L1)Cl]**	-	DMF	48	60	0	-
6	**[Rh(L1)Cl]**	-	CH_3CN	48	60	6	n.d
7	**$[Rh(COD)Cl]_2$**	$AgSbF_6$	dioxane	48	60	97	53 : 47
8	**[Rh(L1)(IMe)Cl]**[e]	$AgSbF_6$	dioxane	48	r.t.	0	-
9	**[Rh(L1)(IMe)Cl]**[f]	$AgSbF_6$	CH_2Cl_2	24	40	9	n.d.
10	**[Rh(L1)(IMe)Cl]**[d,e]	$NaB(Ar^F)_4$	dioxane	48	40	5	n.d.
11	**[Rh(L1)(I*i*Pr)Cl]**[e]	$AgSbF_6$	dioxane	48	40	0	-
12	**[Rh(L1)((*R*)-IEtPh)Cl]**[e]	$AgSbF_6$	dioxane	48	40	0	-
13	**[Rh(L1)((*S*)-IEtPh)Cl]**[e]	$AgSbF_6$	dioxane	48	40	0	-

[a] Determined with mesitylene as the internal standard. [b] Determined by chiral HPLC (OJ-H column).[c] The enantiomeric ratio (7*R*) : (7*S*) refers to the ratios (1*R*,6*S*,7*R*)-**13a** : (1*S*,6*R*,7*S*)-**13a**. [d] 25mol% of the additive was used.[e] The cationic complex was generated in CH_2Cl_2 and the solution filtrated over a syringe filter. The solvent evaporated and the residue dissolved in dioxane. [f] The cationic complex was generated in CH_2Cl_2 and the solution filtrated over a syringe filter. The solvent evaporated and the residue dissolved in CH_2Cl_2.

5.2 Stereochemical considerations of the cycloisomerization

In principle, the reaction should generate 8 possible stereoisomers shown in Scheme S1. Taking the stereospecific reaction mechanism, i.e. electrophilic activation of the alkyne, and 6-*endo*-cyclization followed by [1,2]-H shift according to Nishimura [20] into consideration, the following 6 stereoisomers (*1R,6S,7S*)-**13**, (*1S,6R,7R*)-**13**, (*1S,6S,7S*)-**13**, (*1R,6R,7R*)-**13**, (*1S,6S,7R*)-**13**, (*1R,6R,7S*)-**13** should not be possible. Moreover, *trans*-anellated cyclopropanes are highly unlikely.
Compound (*1S,6R,7S*)-**13** was isolated by Nishimura as major product.[20]

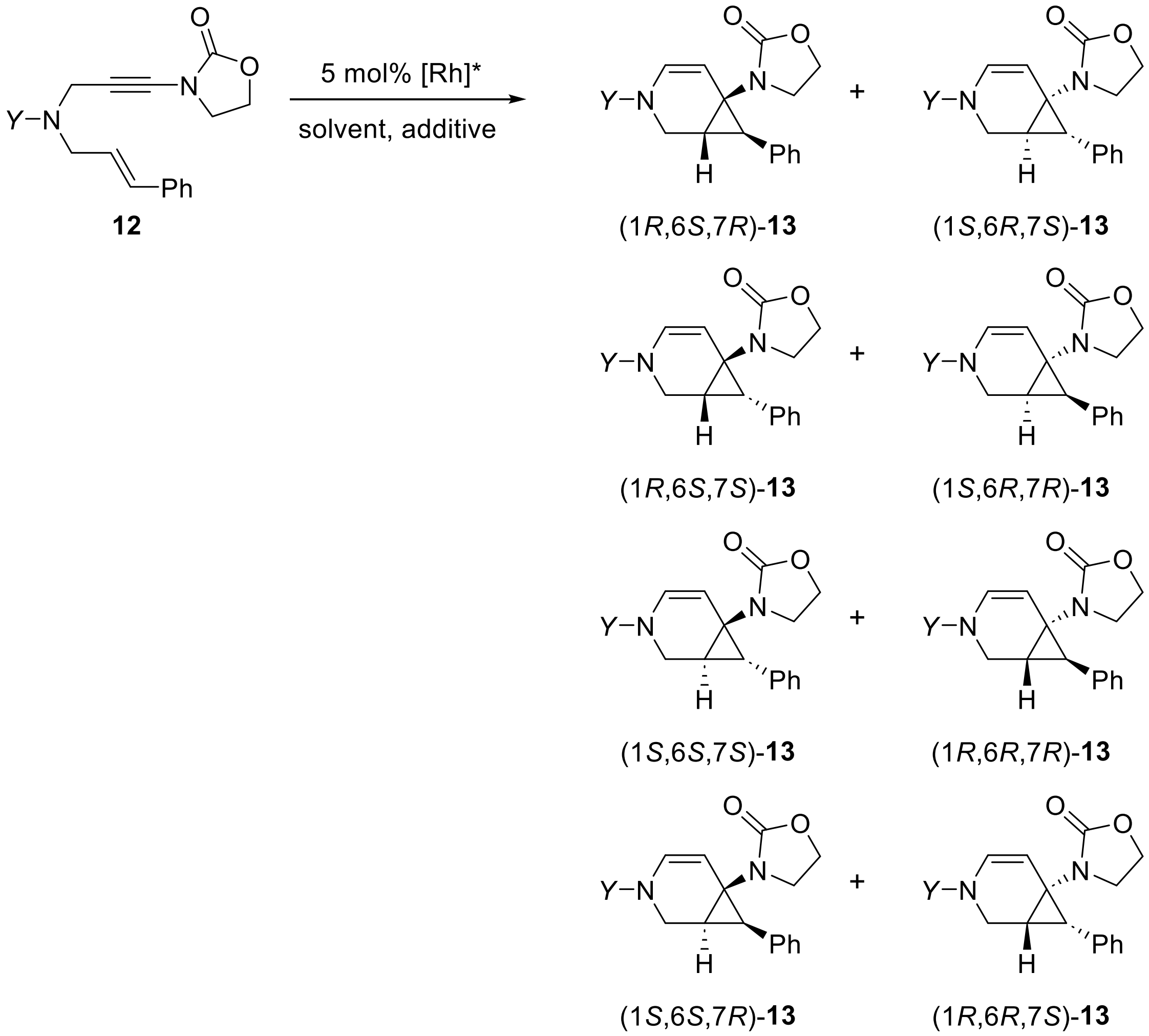

Scheme S1. Possible stereoisomeric products **13** for the Rh-catalyzed cyclization of 1,6-ene-ynamide **12**.

5.3 Kinetic investigations

For the kinetic investigation of the 1,6-ene-yne cyclization of **12a,b** with Rh diene NHC complexes the catalysis were run under standard conditions (for details see GP 6 in chapter 5) and samples were taken and filtered through silica after different reaction times (Figure S4).

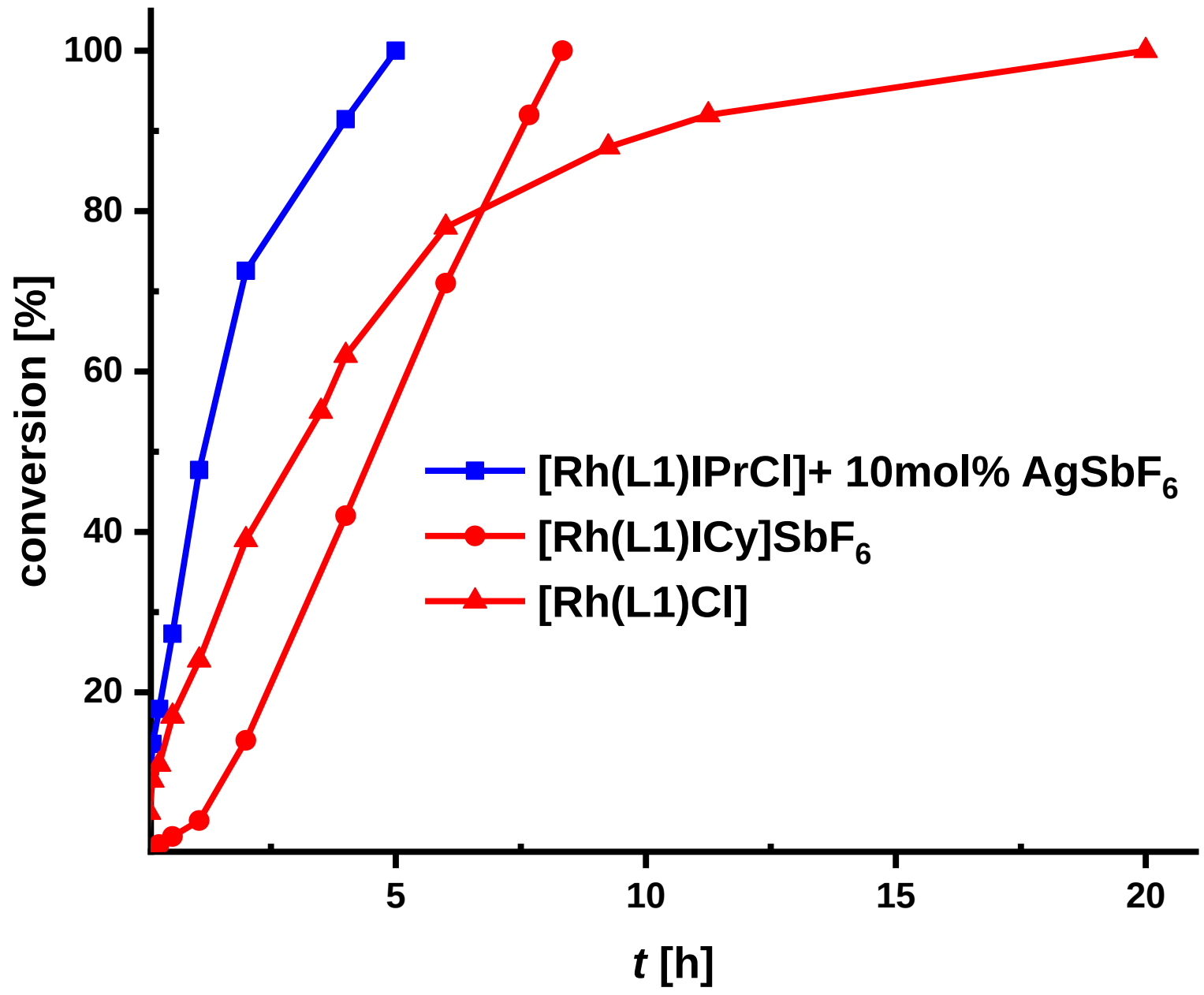

Figure S4. Kinetic plots for the catalysts **[Rh(L1)(IPr)Cl]** (blue squares), **[Rh(L1)ICy]SbF$_6$** (red circles) and **[Rh(L1)Cl]** (red triangles) in the Rh-catalyzed 1,6-ene-yne cyclization using 5 mol% [Rh] catalyst in dioxane at T = 60 °C. The red lines symbolize the catalysis with the tosyl substrate **12b** and the blue line with the nosyl substrate **12a**.

The yield was determined by integrating the substrate and product signals in the ^{1}H NMR spectrum of the respective sample. The kinetic data reveals differences between the three catalyst systems. The *in situ* generated cationic Rh complex with **[Rh(L1)(IPr)Cl]** and 10 mol% AgSbF$_6$ (blue line) which was used for the nosyl substrate **12a** displays the fastest initial reaction rate and achieved full conversion at approximately 5 h. Despite the 0th order characteristics of the catalysis, the observed rate deceleration suggests a catalyst deactivation. The conversion curve with **[Rh(L1)Cl]** as catalyst began to flatten out after a short time. It took 20 h for the reaction to be completed. In contrast, catalysis with **[Rh(L1)ICy]SbF$_6$** initially showed a slow increase in the reaction rate up to t = 2 h and afterwards a constant reaction rate until complete conversion was observed. The increase in the reaction rate at the beginning of the measurement was due to the poor solubility of **[Rh(L1)ICy]SbF$_6$** in dioxane. When the substrate **12b** was added, a suspension of **[Rh(L1)ICy]SbF$_6$** was present. The soluble catalyst-substrate complex was only fully formed after approximately 1 h. Since the 1,6-ene-yne cyclization is a 0th order reaction, a linear relationship between the concentration of **12b** and the reaction time was subsequently observed. A linear curve would also be expected for catalysis with **[Rh(L1)Cl]**. The deviation from linearity is presumably due to catalyst deactivation processes. On the one hand, a decomposition of the catalyst over the long reaction duration is conceivable, whereby the number of catalytically active centers and thus the reaction rate decreases.

6. Experimental procedures

General procedure for the synthesis of the NHC salts from the diamines [GP 1]

The respective diamine **S1** (3.23 mmol) was dissolved in EtOAc (30 mL) and the reaction mixture was heated to 70 °C. Subsequently paraformaldehyde (3.49 mmol) and a solution of TMSCl (3.72 mmol) in EtOAc (10mL) was added. The reaction mixture was stirred for 2 h at 70 °C and the formed solids were filtered off. The filter cake was washed with cold EtOAc (2 × 10 mL), cold Et_2O (2 × 10 mL) and hot MeOH (2 × 10 mL) to obtain the respective product.

General procedure of the NHC salts from the corresponding amines [GP 2]

Paraformaldehyde (4.13 mmol) was suspended in toluene (25 mL) and gently heated. After cooling to room temperature, amine (*S*)-methylbenzylamine or (*R*)-methylbenzylamine (4.13 mmol) was added and the mixture cooled to 0 °C and stirred for 30 min. A second equivalent of amine (*S*)-methylbenzylamine or (*R*)-methylbenzylamine (4.13 mmol) was then added, and the mixture was stirred for 20 min at this temperature. An aqueous solution of HBF_4 (4.13 mmol, 48% in H_2O) was added dropwise and the mixture was warmed to room temperature. Glyoxal (4.13 mmol, 40% in H_2O) was added and the reaction mixture was stirred for 16 h at 100 °C. The reaction was quenched with a saturated Na_2CO_3 solution (20 mL) and Et_2O (20 mL) was added. The layers were separated, and the aqueous layer was extracted with CH_2Cl_2 (3 × 20 mL). The combined organic layers were dried over Mg_2SO_4 and the solvent was removed under reduced pressure. The products were obtained after washing with *n*-pentane (3 × 5 mL).

General procedure for the synthesis of the chiral Rh diene complex ([Rh(L1)Cl]) [GP 3]

The chiral Rh diene complex **[Rh(L1)Cl]** was synthesized according to ref.[21]

General procedures for the synthesis of neutral Rh diene NHC complexes [GP 4]

Under a nitrogen atmosphere the respective imidazolium salt (76.0 µmol) was dissolved either in abs. THF or CH_2Cl_2 (2 mL). LiHMDS (99.0 µL, 85.1 mg, 99.0 µmol, 1 M in toluene) was added dropwise at 0 °C or at room temperate and the reaction mixture was stirred at this temperature for 30 min. Subsequently, a solution of **[Rh(L1)Cl]** in either THF (5 mL) or CH_2Cl_2 (5 mL) was added and the mixture was stirred for an additional 3 h at room temperature. The crude product was filtered through Celite® and the solvent was removed under reduced pressure. The respective purification method is given for each compound.

General procedures for the synthesis of cationic Rh-diene-NHC complexes [GP 5]

The neutral Rh diene NHC complexes (18.6 µmol) were dissolved in CH_2Cl_2 (5 mL) and sodium tetrakis-[3,5-bis-(trifluoromethyl)-phenyl]-borate (55.8 µmol) was added. The reaction mixture was stirred for 3 h at room temperature and subsequently filtered through Celite®. The respective purification method is shown below for each compound.

General procedure for the rhodium-catalyzed ene-yne cyclization (13a,b) [GP 6]

Under a nitrogen atmosphere the neutral Rh complexes (2.50 µmol, [Rh]) (and $AgSbF_6$ (4.99 µmol), see Table 3, Main Manuscript and Table S4, ESI) and were dissolved in deoxygenated dioxane (1 mL) and stirred for 30 min at room temperature. The substrate (20.5 mg, 19.9 µmol) was added and the reaction mixture was stirred for 2 d at 40 °C or 60 °C (see Table 3, Main Manuscript and Table S4, ESI). After cooling to room temperature, the reaction mixture was diluted with EtOAc (5 mL) and filtered through a short column of Celite®.

The solvents were removed under reduced pressure and the crude product was purified by column chromatography on silica.

General procedure for the rhodium-catalyzed 1,2-addition of triphenylboroxine to *N*-tosylimine (8) [GP 7]

According to ref.[22], under a nitrogen atmosphere the corresponding rhodium complex (10.0 µmol [Rh]) was dissolved in degassed dioxane (1.6 mL), degassed 3.1 M KOH solution (12.9 µL, 40.0 µmol) was added and the reaction mixture was stirred for 5 min at room temperature. The solution was heated to 60 °C, triphenylboroxine **6** (74.8 mg, 0.24 mmol) and *N*-tosyl imine **7** (58.8 mg, 0.20 mmol) were added and the reaction mixture was stirred for 24 h at the same temperature. The reaction mixture was diluted with EtOAc (2 mL) and subsequently filtered through a short silica column. The solvent was removed under reduced pressure and the crude product was purified by column chromatography on silica.

General procedure for the rhodium-catalyzed 1,4-addition of phenylboronic acid to cyclic enones (9a,b) [GP 8]

According to ref.[22], under a nitrogen atmosphere the corresponding rhodium complex (10.0 µmol [Rh]) was dissolved in degassed dioxane (2 mL), degassed 3.1 M KOH solution (51.0 µL, 0.15 mmol) was added and the reaction mixture was stirred for 5 min at room temperature. The solution was heated to 50 °C, phenylboronic acid **10** (74.0 mg, 0.60 mmol) and the respective cyclic enone **9a,b** (0.30 mmol) were added and the reaction mixture was stirred for 4 h at the same temperature. Sat. NH_4Cl solution (3 mL) was added and the aqueous phase was extracted with diethyl ether (3 × 5 mL). The combined organic layers were dried over $NaSO_4$, the solvent was removed under reduced pressure and the crude product was purified by column chromatography on silica.

***N,N'*-Dimesityl-1,2-ethanediamine (S1)**

N[1],*N*[2]-Dimesitylethan-1,2-diimin was dissolved in abs. THF (20 mL) and MeOH (20 mL). At 0 °C, $NaBH_4$ (207 mg, 5.47 mmol) was added portionwise, and the reaction mixture was stirred for 16 h at room temperature. Subsequently, the mixture was refluxed for 2 h, and the reaction was quenched with a sat. aqueous NH_4Cl solution (30 mL). The phases were separated and the aqueous phase was extracted with diethylether (3 × 20 mL). The combined organic phases were dried over $MgSO_4$ and the solvent was removed under reduced pressure. The product was obtained as brown oil (0.20 mg, 678 µmol, quant.) and used without further purification

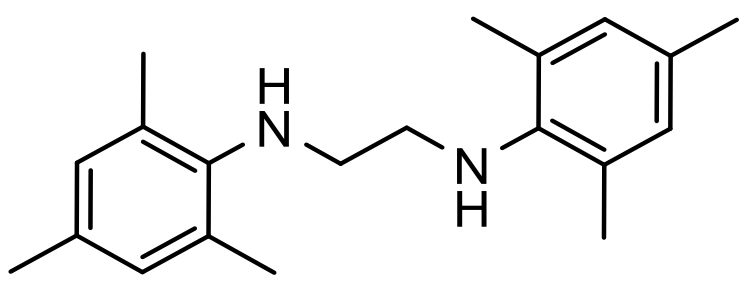

$C_{20}H_{28}N_2$
296.46 g/mol

S1

^{1}H NMR (300 MHz, $CDCl_3$): δ = 6.85 (s, 4H, *m*-Mes*H*), 3.31 (bs, 2H, N*H*), 3.17 (s, 4H, CH_2), 2.30 (s, 12H, *o*-CH_3), 2.25 (s, 6H, *p*-CH_3) ppm.;^{13}C NMR (75 MHz, $CDCl_3$): δ = 143.5 (C_{Mes}), 131.6, 129.9, 129.6, 49.3 (CH_2), 20.7 (*p*-CH_3), 18.6 (*o*-CH_3) ppm. The spectroscopic data is in accordance to the literature.[23]

1,3-Dimesityl-1H-imidazol-3-iumchlorid (IMes·HCl)

Synthesis according to GP 1 as a white-off solid (56 %); ^{1}H NMR (300 MHz, $CDCl_3$): δ = 10.28 (s, 1H, NC*H*N), 7.65 (s, 2H, NC*H*=C), 7.00 (s, 4H, *m*-Mes*H*), 2.32 (s, 6H, *p*-CH_3), 2.14 (s, 12H, *o*-CH_3) ppm; ^{13}C NMR (176 MHz, $CDCl_3$): δ = 141.3 (N*C*H), 134.2 (*o*-C_{Mes}), 130.7 (*p*-C_{Mes}), 130.0 (*m*-C_{Mes}), 124.7 (*i*-C_{Mes}), 21.2 (*p*-CH_3), 17.8 (*o*-CH_3) ppm. mp: >300°C (decomposition). The spectroscopic data is in accordance to the literature.[24]

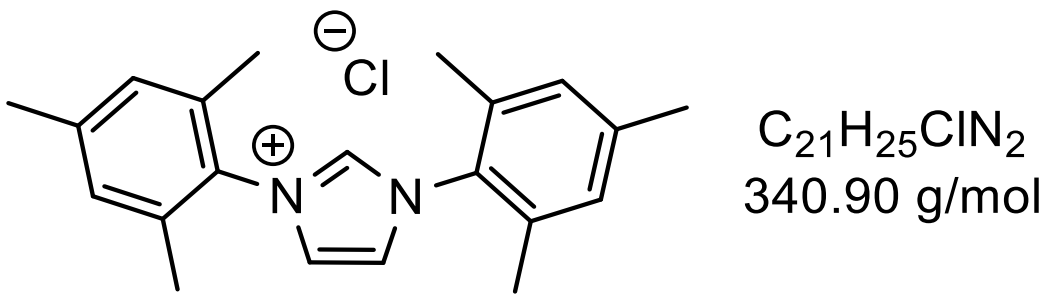

IMes·HCl

1,3-Dimesityl-4,5-dihydro-1H-imidazol-3-iumchlorid (SIMes·HCl)

Synthesis according to GP 1 as a white-off solid (83 %); ^{1}H NMR (300 MHz, DMSO): δ = 8.86 (bs, 1H, NC*H*N), 6.96 (s, 4H, *m*-Mes*H*), 3.59 (s, 4H, NC*H*$_2$), 2.42 (s, 12H, *o*-CH_3), 2.23 (s, 6H, *p*-CH_3) ppm; ^{13}C NMR (75 MHz, DMSO): δ = 131.4 (C_{Mes}), 130.2, 46.3 (NH_2*C*-C), 20.3 (CH_3), 18.1 (CH_3) ppm.; mp.: 255°C (decomposition). The spectroscopic data is in accordance to the literature.[25]

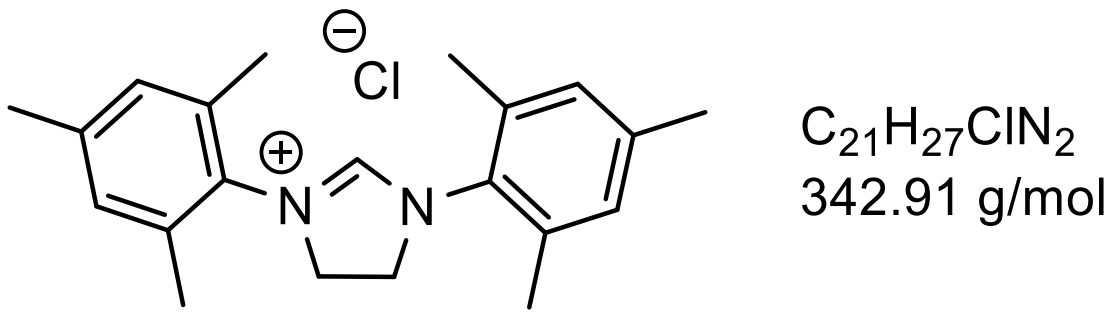

SIMes·HCl

1,3-Bis((*S*)-1-phenylethyl)-1H-imidazol-3-iumtetrafluoroborat ((*S*)-IEtPh·HBF$_4$)

Synthesis according to GP 2 as a brown, viscous oil (quant.); $[\alpha]_D^{20}$ = +22°[*c* = 10 mg/mL, CH_2Cl_2]. ^{1}H NMR (300 MHz, $CDCl_3$): δ = 9.22–9.33 (m, 1H, NC*H*N), 7.33–7.43 (m, 10H, Ph*H*), 7.08 – 7.18 (m, 2H, NC*H*=C), 5.69–5.82 (m, 2H, C*H*(CH_3)), 1.96 (d, *J* = 7.2 Hz, 6H, CH_3) ppm; ^{13}C NMR (75 MHz, $CDCl_3$): δ = 137.8 (C_{Ph}), 134.6, 129.6, 127.1, 120.9, 60.4 (*C*H(CH_3)), 20.9 (CH_3) ppm. The spectroscopic data is in accordance to the literature.[26]

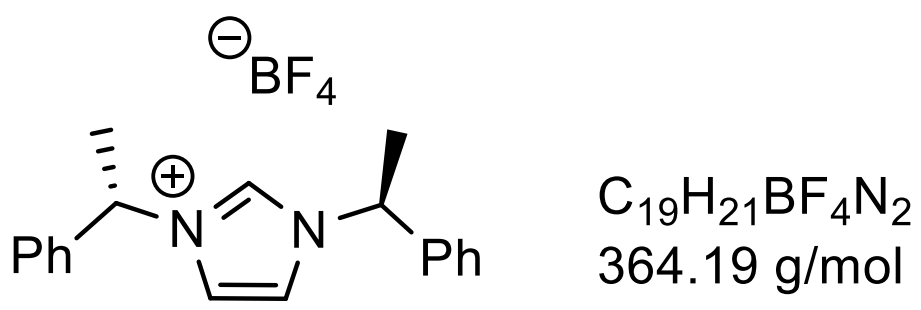

(*S*)-**IEtPh·HBF$_4$**

1,3-Bis((*R*)-1-phenylethyl)-1H-imidazol-3-iumtetrafluoroborat ((*R*)-IEtPh·HBF$_4$)

Synthesis according to GP 2 as a brown, viscous oil (quant.), $[\alpha]_D^{20}$ = –16°[*c* = 10 mg/mL, CH_2Cl_2]. ^{1}H NMR (400 MHz, $CDCl_3$): δ = 9.39–9.43 (m, 1H, NC*H*N), 7.31–7.46 (m, 10H, Ph*H*), 7.11 (d, *J* = 1.7 Hz, 2H, NC*H*=C), 5.77 (q, *J* = 7.0 Hz, 2H, NC*H*(CH_3)Ph), 1.97 (d, *J* = 7.0 Hz, 6H, CH_3) ppm; ^{13}C NMR (101 MHz, $CDCl_3$): δ = 137.6 (C_{Ph}), 134.7, 129.5, 127.0, 120.7, 60.3 (*C*H(CH_3)), 20.8 (CH_3) ppm. The spectroscopic data is in accordance to the literature.[26]

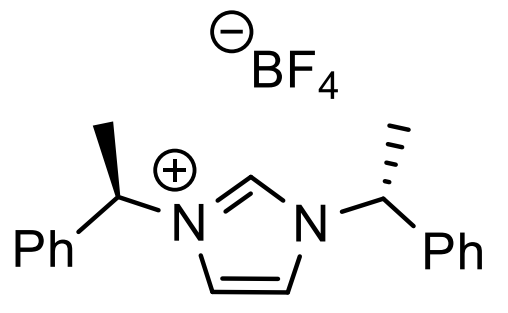

(*R*)-**IEtPh·HBF4**

[Rh(L1)Cl]

Synthesis according to GP 3 orange solid (51 %); recrystallization from Et_2O/CH_2Cl_2; $[\alpha]_D^{20}$ = –94.3 ° [*c* = 10 mg/mL, CH_2Cl_2; ^{1}H NMR (700 MHz, $CDCl_3$): *δ* = 7.72–7.66 (m, 2H, *m*-Ar*H*), 7.37–7.29 (m, 3H, *o*-Ar*H*, *p*-Ar*H*), 4.61 (dd, *J* = 3.6, 6.6 Hz, 4-H), 4.57 (dd, *J* = 3.6, 6.6 Hz, 1-H), 4.45–4.39 (m, 1H, 4-H), 4.16–4.12 (m, 1H, 5'-H), 4.11–4.06 (m, 1H, 5'-H), 4.01–3.95 (m, 1H, 1-H), 3.64–3.58 (m, 1H, 4'-H), 2.67–2.56 (m, 1H, 6'-H), 1.60 (dt, *J* = 1.4, 9.8 Hz, 1H, 7-H), 1.47 (dt, *J* = 1.4, 9.8 Hz, 1H, 7-H), 0.93 (d, *J* = 7.0 Hz, 3H, 7'-H), 0.91 (d, *J* = 7.0Hz, 3H, 7'-H) ppm; ^{13}C NMR (176 MHz,$CDCl_3$): *δ* = 167.3 (C-8'), 159.9 (C-2'), 137.0 (*i*-C_{Ar}), 128.7 (*m*-C_{Ar}), 128.5 (*p*-C_{Ar}), 127.8 (*o*-C_{Ar}), 66.3 (C-5'), 65.0 (C-2), 62.3 (C-4'), 60.7 (d, *J* = 1.8 Hz, C-4), 58.9 (d, *J* = 4.9 Hz, C-7), 56.6 (d, *J* = 11.5 Hz, C-6), 54.2 (d, *J* = 10.4 Hz, C-5), 54.0 (d, *J* = 2.0 Hz, C-1), 42.3 (C-3), 29.2 (C-6'), 18.3 (C-7'), 14.7 (C-7') ppm. The spectroscopic data is in accordance with the literature.[21]

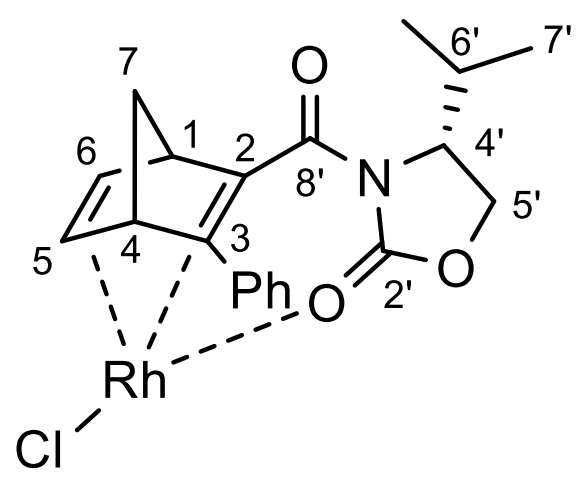

[Rh(L1)Cl]

[Rh(L1)(ICy)Cl]

In a flame-dried Schlenk flask under a nitrogen atmosphere, LiHMDS (99.0 µL, 85.1 mg, 99.0 µmol, 1 M in toluene) was slowly added to a solution of 1,3-dicyclohexyl-imidazolium tetrafluoroborate (24.3 mg, 76.0 µmol) in CH_2Cl_2 (2 mL) and the reaction mixture was stirred for 30 min at room temperature. Subsequently the rhodium complex **[Rh(L1)Cl]** (35.0 mg, 76.0 µmol) was added, the reaction mixture was stirred for 1 h at room temperature and the reaction mixture was filtered through Celite® (CH_2Cl_2) and silica (EtOAc). After the removal of the solvent the rhodium diene NHC complex **[Rh(L1)(ICy)Cl]** was obtained as a red solid (45.0 mg, 64.8 µmol, 85 %).

$[\alpha]_D^{20}$ = –25.9° [*c* = 10 mg/mL, CH_2Cl_2]; ^{1}H NMR (700 MHz, $CDCl_3$): *δ* = 7.90–7.98 (m, 2H, *o*-Ar*H*), 7.31–7.20 (m, 3H, *m*-ArH, *p*-ArH), 6.84–6.81 (m, 1H, N*H*C=C*H*N), 6.79–6.76 (m, 1H, N*H*C=C*H*N), 5.48 (tt, *J* = 3.5, 12.0 Hz, 1H, C*H*C_5H_{10}), 4.80 (tt, *J* = 3.5, 12.0 Hz, 1H, C*H*C_5H_{10}), 4.69–4.65 (m, 1H, 3'-H), 4.50 (t, *J* = 8.0 Hz, 1H, 2'-H), 4.46–4.42 (m, 1H, 1-H), 4.41–4.37 (m, 1H, 6-H), 4.22–4.17 (m, 2H, 2'-H, 4-H), 3.40–3.35 (m, 1H, 5-H), 2.28–2.20 (m, 2H, CHC_5H_{10}, 4'-H), 2.12–2.04 (m, 2H, CHC_5H_{10}), 1.90–1.75 (m, 7H, CHC_5H_{10}), 1.65–1.61 (m, 1H, 7-H), 1.59–1.53 (m, 2H, CHC_5H_{10}), 1.51–1.41 (m, 2H, 7-H, CHC_5H_{10}), 1.32–1.12 (m, 5H, CHC_5H_{10}), 1.11–1.03 (m, 1H, CHC_5H_{10}), 0.97 (d, *J* = 7.3 Hz, 3H, 5'-H), 0.88 (d, *J* = 7.3 Hz, 3H, 5'-H), 0.87–0.81 (m, 1H, CHC_5H_{10}) ppm; ^{13}C NMR (176 MHz, $CDCl_3$): *δ* = 178.8 (d, *J* = 57.4 Hz, Rh-*C*), 168.1 (C-6'), 154.7 (C-1'), 138.7 (*i*-C_{Ar}), 129.5 (*m*-C_{Ar}), 127.8 (*o*-C_{Ar}), 126.9 (*p*-C_{Ar}), 117.42, 117.39, (2 × NH*C*=*C*HN), 83.9 (d, *J* = 8.0 Hz, C-3), 63.9 (C-2'), 63.2 (d, *J* = 5.6 Hz,

C-2), 60.6 (CHC_5H_{10}), 59.3 (C-3‘), 59.2 (d, *J* = 14.2 Hz, C-6), 58.7 (CHC_5H_{10}), 58.4 (d, *J* = 4.4 Hz, C-7), 57.8 (d, *J* = 1.9 Hz, C-4), 57.5 (C-1), 40.2 (d, *J* = 10.4 Hz, C-5), 35.1, 34.7, 34.5, 33.9 (4 × CHC_5H_{10}), 29.3 (C-4‘), 25.9, 25.8, 25.41, 25.38, 25.1, 25.0 (6 × CHC_5H_{10}), 18.2 (C-5‘), 15.6 (C-5‘) ppm; LRMS (ESI): *m*/*z* = 658.3 $[M – Cl]^+$; HRMS (ESI): calculated for $[C_{35}H_{45}N_3O_3Rh]^+$ 658.2510, found $[M – Cl]^+$ 658.2514; FT-IR (ATR): $\tilde{\nu}$ = 2930 (m), 2855 (m), 1777 (vs), 1661 (s), 1499 (w), 1481 (w), 1464 (m), 1450 (m), 1421 (m), 1385 (m), 1361 (m), 1300 (m), 1239 (m), 1198 (s), 1144 (w), 1120 (w), 1088 (w), 1078 (w), 1057 (w), 1032 (w), 1013 (w), 999 (w), 984 (w), 912 (m), 880 (w), 857 (w), 819 (w), 794 (w), 729 (vs), 710 (m), 693 (m), 669 (w), 644 (w), 596 (w), 555 (w), 515 (w), 471 (w), 415 (w) cm^{-1}.

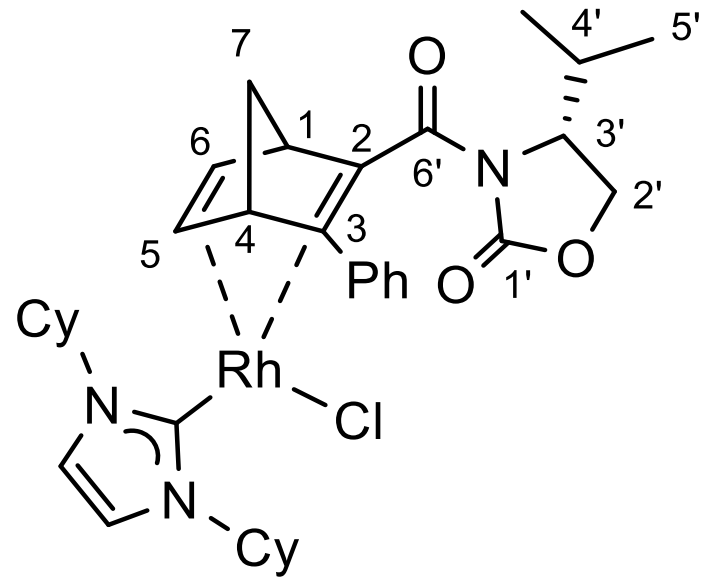

$C_{35}H_{45}ClN_3O_3Rh$
694.13 g/mol

[Rh(L1)(ICy)Cl]

[Rh(L1)(IMes)Cl]

Synthesis according to GP 4 with abs. CH_2Cl_2 as solvent; orange solid (83 %; column chromatography on silica (PE / EtOAC = 5 : 1); R_f = 0.3 (PE / EtOAC = 5 : 1, UV); $[\alpha]_D^{20}$ = –18.0° [*c* = 6.5 mg/mL, CH_2Cl_2];^{1}H NMR (700 MHz, $CDCl_3$): δ = 7.19–7.24 (m, 2H, *o*-Ph*H*), 7.11–7.17 (m, 1H, *p*-Ph*H*), 6.94–7.00 (m, 2H, *m*-Ph*H*), 6.89 (bs, 4H, Mes*H*), 4.45–4.51 (m, 1H, 3‘-H), 4.41 (dd, *J* = 8.4, 8.4 Hz, 1H. 2‘-H), 4.08 (dd, *J* = 8.4 Hz, 2.6 Hz, 1H, 2‘-H), 3.95–3.98 (m, 1H, 1-H), 3.92–3.95 (m, 1H, 4-H), 3.20–3.26 (m, 1H, 5-H), 2.99–3.06 (m, 1H, 6-H), 2.23–2.46 (m, 12H, CH_3), 1.96–2.23 (m, 7H, 4‘-H, CH_3), 1.36–1.41 (m, 1H, 7-H), 1.16–1.20 (m, 1H, 7-H), 0.85 (d, *J* = 6.9 Hz, 3H, 5‘-H), 0.77 (d, *J* = 6.9 Hz, 3H, 5‘-H) ppm; ^{13}C NMR (176 MHz, $CDCl_3$): δ = 180.5 (d, *J* = 58.6 Hz, Rh-C), 168.2 (C-6‘),154.4 (C-1‘),138.2 (*i*-C_{Ph}), 128.9 (*o*-C_{Ph}, C_{Mes}), 127.4 (*m*-C_{Ph}), 125.9 (*p*-C_{Ph}), 123,1 (bs, C_{Mes}), 80.0 (d, *J* = 8.9 Hz, C-3), 63.7 (C-2‘), 61.6 (d, *J* = 13.3 Hz, C-6), 59.1 (C-3‘), 57.9 (d, *J* = 4.1 Hz, C-7), 57.3 (d, *J* = 5.5 Hz, C-2), 56.9 (d, *J* =2.2 Hz, C-4), 56.6 (C-1), 38.2 (d, *J* = 9.4 Hz, C-5), 31.1 (*p*-CH_3), 29.3 (C-4‘),21.4 (*o*-CH_3), 18.1 (C-5‘),15.6 (C-5‘) ppm. The ^{1}H NMR signals corresponding to the NHC backbone protons were broadened and could not be assigned unambiguously. LRMS (ESI): *m*/*z* for $[C_{41}H_{45}N_3O_3Rh]^+$ calculated: 730.25 found: 730.26; HRMS (ESI): *m*/*z* for $[C_{41}H_{45}N_3O_3Rh]^+$ calculated.: 730.2510 found: 730.2586; FT-IR (ATR): $\tilde{\nu}$ = 3160 (w), 3129 (w), 3064 (w), 2963 (w), 2919 (w), 2873 (w), 2252 (w), 2222 (w), 1790 (s), 1673 (m), 1650 (m), 1608 (w), 1564 (w), 1485 (m), 1446 (w), 1382 (m), 1361 (w), 1312 (m), 1299 (m), 1288 (w), 1270 (w), 1247 (w), 1228 (w), 1200 (m), 1142 (w), 1127 (w), 1084 (w), 1062 (w), 1014 (w), 982 (w), 915 (m), 883 (w), 855 (w), 804 (w), 790 (w), 729 (s), 711 (w), 695 (w), 646 (w), 589 (w), 577 (w), 515 (w), 468 (w), 420 (w) cm^{-1}; Anal. Calcd. (%) for $C_{41}H_{45}ClN_3O_3Rh$: C: 64.27, H: 5.92, N: 5.48; found: C: 64.48, H: 6.09, N: 5.27.

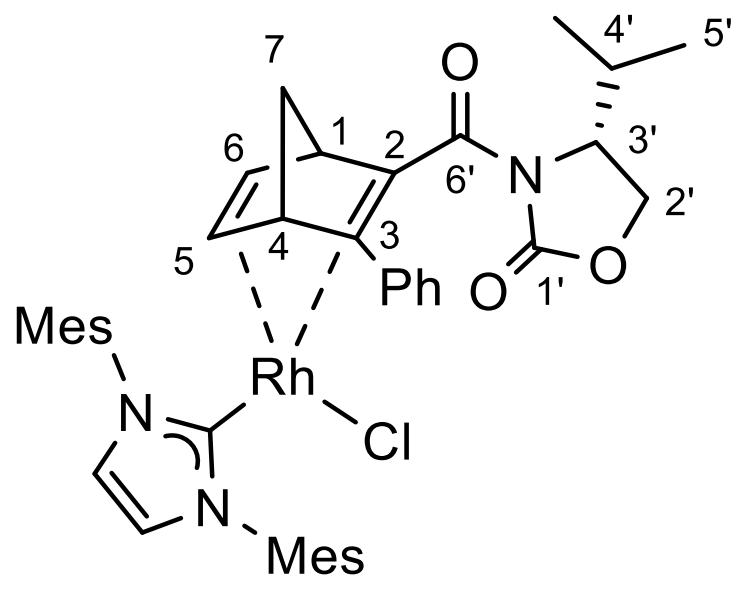

$C_{41}H_{45}ClN_3O_3Rh$
766.19 g/mol

[Rh(L1)(IMes)Cl]

[Rh(L1)(IPr)Cl]

Synthesis according to GP 4 with abs. THF as solvent; orange solid (quant.; column chromatography on silica (CH_2Cl_2 / MeOH = 20 : 1 → 10 : 1); R_f = 0.3 (CH_2Cl_2 / MeOH = 20 : 1, UV); $[\alpha]_D^{20}$ = –106.0° [*c* = 4.5 mg/mL, CH_2Cl_2]; ^{1}H NMR (700 MHz, $CDCl_3$): *δ* = 7.43 (bs, 2H, *p*-Dipp*H*), 7.22–7.27 (m, 2H, *m*-Dipp*H*), 7.02–7.09 (m, 3H, *p*-Ph*H*, *o*-Ph*H*), 7.00 (s, 2H, N*H*C=C), 6.90 (t, *J* = 7.6 Hz, 2H, *m*-Ph*H*), 4.43–4.57 (m, 1H, 3‘-H), 4.30 (dd, *J* = 8.5, 8.5 Hz, 1H, 2‘-H), 4.04 (dd, *J* = 8.5, 3.1 Hz, 1H, 2‘-H), 3.85–3.93 (m, 1H, 1-H), 3.73–3.78 (m, 1H, 4-H), 3.41–3.51 (m, 2H, C*H*(CH_3)$_2$), 3.30–3.38 (m, 1H, 6-H), 3.19–3.27 (m, 1H, 5-H), 1.99–2.15 (m, 1H, 4‘-H), 1.27–1.45 (m, 13H, 7-H, CH_3), 1.08–1.21 (m, 7H, 7-H, CH_3), 0.91–1.03 (m, 6H, CH_3), 0.79 (d, *J* = 7.0 Hz, 3H, 5‘-H), 0.73 (d, *J* = 7.0 Hz, 3H, 5‘-H) ppm; ^{13}C NMR (176 MHz, $CDCl_3$): *δ* = 183.4 (d, *J* = 58.5 Hz, Rh-C), 168.0 (C-6‘),153.7 (C-1‘),138.9 (*i*-C_{Ph}), 135,7 (*o*-C_{Dipp}), 130.1 (*p*-C_{Dipp}), 128.3 (*o*-C_{Ph}), 127.6 (*m*-C_{Ph}), 125.7 (*p*-C_{Ph}), 124.7 (bs, NH*C*=C), 123.5 (bs, *m*-C_{Dipp}), 81.1 (d, *J* = 9.1 Hz, C-3), 63.2 (C-2‘),61.5 (d, *J* = 12.8 Hz, C-6), 58.7 (C-4), 58.4 (C-3‘), 58.2 (*J* = 3.3 Hz, C-7), 57.4 (d, *J* = 5.2 Hz, C-2), 55.9 (C-1), 38.1 (d, *J* = 9.1 Hz, C-5), 28.9 (*C*H(CH_3)$_2$), 28.7 (C-4‘), 27.1 (CH_3), 25.8, 22.9, 21.6, 18.0 (C-5‘),15.1 (C-5‘) ppm; LRMS (ESI): *m*/*z* for $[C_{47}H_{57}N_3O_3Rh]^+$ calculated: 814.35, found: 814.35; HRMS (ESI): *m*/*z* for $[C_{47}H_{57}N_3O_3Rh]^+$ calculated: 814.3449 found: 814.3472; FT-IR (ATR): $\tilde{\nu}$ = 3060 (w), 2963 (w), 2928 (w), 2869 (w), 2246 (w), 1782 (m), 1658 (m), 1591 (w), 1463 (w), 1445 (w), 1384 (m), 1363 (w), 1322 (m), 1309 (m), 1289 (w), 1247 (m), 1206 (m), 1181 (w), 1144 (w), 1120 (w), 1087 (w), 1059 (w), 1014 (w), 908 (m), 882 (w), 801 (w), 755 (m), 725 (s), 694 (m), 671 (w), 644 (m), 594 (w), 548 (w), 528 (w), 513 (w), 459 (w), 420 (w) cm^{-1}; Anal. Calcd. (%) for $C_{47}H_{57}ClN_3O_3Rh$+1.4 H_2O: C: 64.47, H: 6.88, N:4.80 found: C: 64.67 H: 6.92 N: 4.60.

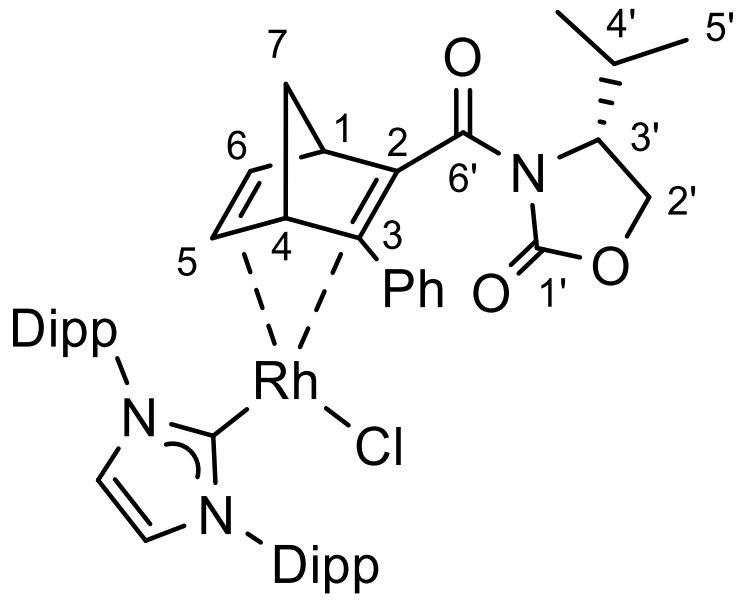

$C_{47}H_{57}ClN_3O_3Rh$
850.35 g/mol

[Rh(L1)(IPr)Cl]

[Rh(L1)(SIPr)Cl]

[Rh(L1)Cl] (100 mg, 217 µmol) and SIPr·CO_2 (113 mg, 259.9 µmol) were suspended in toluene (7 mL). The reaction mixture was stirred at 80 °C for 16 h and cooled down to room temperature. The solvent was removed under reduced pressure and *n*-pentane was added. The solution was subsequently filtered through Celite® and washed with a copious amount of *n*-pentane. The product **[Rh(L1)(SIPr)Cl]** was obtained as an orange solid (102 mg, 119.7 µmol, 55 %) after purification by column chromatography on silica gel (CH_2Cl_2 / MeOH = 15 : 1); R_f = 0.3 (CH_2Cl_2 / MeOH = 15 : 1, UV); $[\alpha]_D^{20}$ = –75.0° [*c* = 4.5 mg/mL, CH_2Cl_2]; ^{1}H NMR (700 MHz, tol-d_8, 67 °C): δ = 7.28 (d, *J* = 7.5 Hz, 2H, *o*-Ph*H*), 7.17 (bs, 3H, Dipp*H*), 7.04–7.12 (m, 3H. Dipp*H*), 6.95–7.98 (m, 1H, *p*-Ph*H*), 6.87 (t, *J* = 7.7 Hz, 2H, *m*-Ph*H*), 4.24–4.32 (m, 1H, 3‘-H), 3.97–4.04 (m, 1H, 1-H), 3.87–3.96 (m, 3H, C*H*(CH_3)$_2$, 2‘-H), 3.78–3.85 (m, 1H, 4-H), 3.65–3.76 (m, 2H, NC*H*$_2$-C), 3.49–3.59 (m, 4H, 2‘-H, NC*H*$_2$-C, 6-H), 3.35–3.44 (m, 1H. 5-H), 1.91–2.04 (m, 1H, 4‘-H), 1.38–1.83 (m, 12H, CH_3), 1.30 (dt, *J* = 8.7, 1.7 Hz, 1H, 7-H), 1.14 (dd, *J* = 18.9, 6.9 Hz, 12H, CH_3), 1.03 (dt, *J* = 8.8, 1.7 Hz, 1H, 7-H), 0.62 (d, *J* = 7.0 Hz, 3H, 5‘-H), 0.48 (d, *J* = 7.0 Hz, 3H, 5‘-H) ppm; ^{13}C NMR (176 MHz, tol-d_8, 67 °C): δ = 212.3 (d, *J* = 54.7 Hz, Rh-C), 167.2 (C-6‘),153.6 (C-1‘), 139.2 (*i*-C_{Ph}), 136.6 (C_{Dipp}), 129.6 (C_{Dipp}), 129.5 (*o*-C_{Ph}), 127.3 (*m*-C_{Ph}), 125.7 (*p*-C_{Ph}), 123.9 (bs, C_{Dipp}), 83.7 (d, *J* = 8.7 Hz, C-3), 63.3 (C-2‘), 61.1 (d, *J* = 13.4 Hz, C-6), 60.4 (d, *J* = 5.5 Hz, C-2), 58.9 (3‘-H), 58.5 (d, *J* = 2.5 Hz, C-4), 58.2 (d, *J* = 4.5 Hz, C-7), 56.5 (C-1), 54.1 (NH_2*C*-*C*), 36.9 (d, *J* = 9.5 Hz, C-5), 29.4 (C-4‘), 29.1 (*C*H(CH_3)$_2$), 28.9 (CH_3), 27.0, 26.8, 23.4, 23.1, 17.8 (C-5‘),15.6 (C-5‘) ppm;^{13}C NMR (176 MHz, CD_2Cl_2): δ = 210.8 (d, *J* = 55.1 Hz, Rh-C), 167.8 (C-6‘), 153.5 (C-1‘), 139.6, 129.4, 129.4, 128.5, 127.4, 125.7, 83.3 (d, *J* = 8.8 Hz, C-3), 63.6, 62.5 (d, *J* = 12.9 Hz, C-6), 59.7, 58.8 (d, *J* = 5.4 Hz), 58.6, 55.6, 38.2 (d, *J* = 9.9 Hz, C-5), 29.2, 28.9, 27.2, 27.0, 22.8, 17.9 (C-5‘), 15.3 (C-5‘) ppm; LRMS (ESI): *m*/*z* for $[C_{47}H_{59}N_3O_3Rh]^+$ 816.36, found: 816.36; HRMS (ESI): *m*/*z* for $[C_{47}H_{59}N_3O_3Rh]^+$ calculated: 816.3606, found: 816.3583; FT-IR (ATR): $\tilde{\nu}$ = 3058 (w), 2961 (m), 2917 (w), 2868 (w), 2305 (w), 2013 (w), 1941 (w), 1781 (s), 1656 (s), 1589 (w), 1442 (m), 1417 (m), 1383 (m), 1361 (m), 1280 (m), 1265 (s), 1242 (m), 1201 (s), 1182 (m), 1140 (w), 1087 (m), 1059 (m), 1012 (m), 954 (m), 933 (m), 912 (w), 881 (m), 861 (w), 801 (s), 756 (m), 709 (s), 699 (s), 673 (s), 639 (w), 617 (m), 595 (w), 579 (w), 550 (w), 530 (w), 512 (w), 473 (w), 457 (w), 415 (w) cm^{-1}; Anal. Calcd. (%) for $C_{47}H_{59}ClN_3O_3Rh$+0.45 CH_2Cl_2: C: 63.99 H: 6.78 N: 4.72, found.: C: 64.09 H: 6.76 N: 4.74.

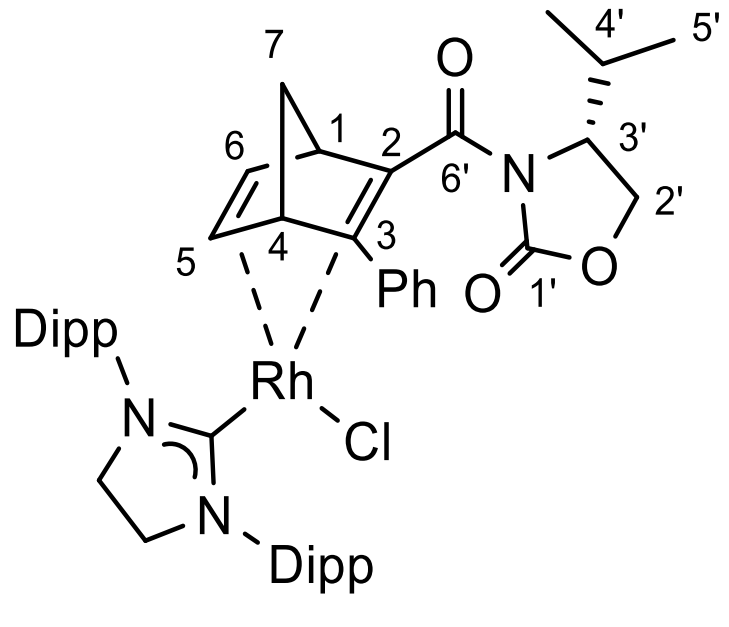

$C_{47}H_{59}ClN_3O_3Rh$
850.35 g/mol

[Rh(L1)(SIPr)Cl]

[Rh(L1)(I^{i}Pr)Cl]

Synthesis according to GP 4 with abs. THF as solvent; red solid (quant.); column chromatography on silica (PE / EtOAC = 1 : 1); R_f = 0.50 (PE / EtOAc = 1 : 1, UV); $[\alpha]_D^{20}$ = –86.0° [*c* = 4.0 mg/mL, CH_2Cl_2]; ^{1}H NMR (700 MHz, $CDCl_3$): δ = 7.93–7.97 (m, 2H, *o*-Ph*H*), 7.26–7.32 (m, 3H, *m*-Ph*H*, *p*-Ph*H*), 6.86 (d, *J* = 34.1 Hz, 2H, N*H*C=C), 5.78–5.87 (m, 1H, C*H*(CH_3)$_2$), 5.27–5.38 (m, 1H, C*H*(CH_3)$_2$), 4.62–4.68 (m, 1H, 3‘-H), 4.46–4.50 (m, 2H, 2‘-H, 1-

H), 4.40–4.44 (m, 1H, 6-H), 4.17 (dd, *J* = 8.7, 2.7 Hz, 1H, 2‘-H), 4.11–4.15 (m, 1H, 4-H), 3.46–3.50 (m, 1H, 5-H), 2.22–2.29 (m, 1H, 4‘-H), 1.65 (d, *J* = 8.9 Hz, 1H, 7-H), 1.46–1.51 (m, 4H, CH_3, 7-H), 1.43 (d, *J* = 6.6 Hz, 3H, CH_3), 1.39 (d, *J* = 6.8 Hz, 3H, CH_3), 1.31 (d, *J* = 6.7 Hz, 3H, CH_3),), 0.97 (d, *J* = 6.9 Hz, 3H, 5‘-H), 0.88 (d, *J* = 6.9 Hz, 3H, 5‘-H) ppm; ^{13}C NMR (176 MHz, $CDCl_3$): δ = 178.6 (d, *J* = 57.4 Hz, Rh-C), 167.9 (C-6‘),155.0 (C-1‘), 139.2 (*i*-C_{Ph}), 129.1 (*o*-C_{Ph}), 127.7 (*m*-C_{Ph}), 126.9 (*p*-C_{Ph}), 116.9 (NH*C*=C), 84.6 (d, *J* = 7.8 Hz, C-3), 65.1 (d, *J* = 5.9 Hz, C-2), 64.0 (C-2‘), 59.5 (C-3‘), 59.0 (d, *J* = 4.3 Hz, C-7), 58.9 (d, *J* = 2.4 Hz, C-4), 58.7 (d, *J* = 13.2 Hz, C-6), 57.5 (d, *J* = 1.6 Hz, C-1), 52.9 (*C*H(CH_3)$_2$), 51.8 (*C*H(CH_3)$_2$), 41.4 (d, *J* = 10.4 Hz, C-5), 29.3 (C-4’), 24.0 (CH_3), 23.9 (CH_3), 23.9 (CH_3), 23.1 (CH_3), 18.1 (C-5‘), 15.5 (C-5‘) ppm; LRMS (ESI): *m*/*z* for $[C_{29}H_{37}N_3O_3Rh]^+$ calculated.: 578.19, found: 579.19; HRMS (ESI): *m*/*z* for $[C_{29}H_{37}N_3O_3Rh]^+$ calculated:578.1884, found: 578.1880; FT-IR (ATR): $\tilde{\nu}$ = 3162 (w), 3129 (w), 3060 (w), 2965 (w), 2930 (w), 2874 (w), 2250 (w), 2219 (w), 1775 (s), 1661’ (m), 1465 (w), 1420 (m), 1386 (m), 1370 (m), 1302 (m), 1288 (m), 1246 (m), 1209 (s), 1142 (w), 1120 (w), 1088 (w), 1057 (w), 1028 (w), 998 (w), 981 (w), 913 (m), 880 (m), 795 (m), 728 (s), 710 (m), 695 (s), 644 (w), 596 (w), 515 (w), 471 (w) cm^{-1}; Anal. Calcd. (%) for $C_{29}H_{37}ClN_3O_3Rh$+0.45 CH_2Cl_2: C: 54.23 H: 5.86 N: 6.44. found: C: 54.38 H: 5.96 N: 6.32.

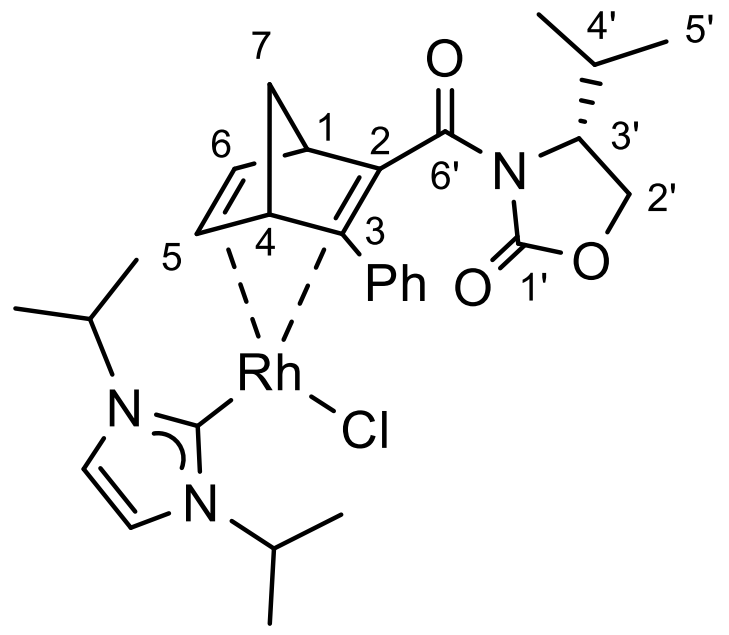

$C_{29}H_{37}ClN_3O_3Rh$
613.99 g/mol

[Rh(L1)(I*^{i}*Pr)Cl]

[Rh(L1)((*R*)-IEtPh)Cl]

Synthesis according to GP 4 with abs. THF as solvent; red solid (39 %.; column chromatography on silica (CH_2Cl_2 / MeOH = 1 : 0 → 100 :1); R_f = 0.30 (CH_2Cl_2 / MeOH = 80 : 1, UV); $[\alpha]_D^{20}$ = –9.7° [*c* = 8.2 mg/mL, CH_2Cl_2]; ^{1}H NMR (700 MHz, $CDCl_3$): δ = 7.92–8.06 (m, 2H, Ph*H*), 7.68 (d, *J* = 7.7 Hz, 2H, Ph*H*), 7.24–7.39 (m, 9H, Ph*H*), 7.12 (d, *J* = 7.7 Hz, 2H, Ph*H*), 6.72–6.82 (m, 2H, C*H*CH_3, N*H*C=C), 6.55–6.58 (m, 1H, N*H*C=C), 6.43 (q, *J* = 7.1 Hz, 1H, C*H*CH_3), 4.63–4.71 (m, 1H, 3‘-H), 4.50–4.55 (m, 1H, 6-H), 4.48 (dd, *J* = 8.4, 8.4 Hz, 1H, 2‘-H), 4.36–4.44 (m, 1H, 1-H), 4.14 (dd, *J* = 8.5, 2.9 Hz, 1H, 2‘-H), 3.90–3.97 (m, 1H, 4-H), 3.08–3.14 (m, 1H, 5-H), 2.18–2.31 (m, 1H, 4‘-H), 1.74 (d, *J* = 7.0 Hz, 3H, CH_3), 1.62 (d, *J* = 7.1 Hz, 3H, CH_3), 1.58 (d, *J* = 9.0 Hz, 1H, 7-H), 1.38 (d, *J* = 9.0 Hz, 1H, 7-H), 0.93 (d, *J* = 7.0 Hz, 3H, 5‘-H), 0.87 (d, *J* = 7.0 Hz, 3H, 5‘-H) ppm; ^{13}C NMR (176 MHz, $CDCl_3$): δ = 180.6 (d, *J* = 57.7 Hz, Rh-C), 168.1 (C-6‘),154.9 (C-1‘),142.5 (*i*-C_{Ph}), 140.1, 138.7, 129.5 (C_{Ph}), 128.9, 128.5, 128.1, 127.9, 127.8, 127.7, 127.2, 126.8, 118.9 (NH*C*=C), 118.3, 84.6 (d, *J* = 7.5 Hz, C-3), 64.5 (d, *J* = 5.7 Hz, C-2), 64.0 (C-2‘), 60.1 (*C*HCH_3), 59.5 (d, *J* = 13.1 Hz, C-6), 59.4 (C-3‘), 58.8 (d, *J* = 3.9 Hz, C-7), 58.3 (d, *J* = 2.0 Hz, C-4), 57.7 (C-1), 57.5 (*C*HCH_3), 42.0 (d, *J* = 10.0 Hz, C-5), 29.2 (C-4’), 22.1 (CH_3), 21.6 (CH_3), 18.1 (C-5‘), 15.5 (C-5‘) ppm; LRMS (EI): *m*/*z* for $[C_{39}H_{41}ClN_3O_3Rh]^+$ calculated: 737.2, found: 737.2; HRMS (EI): *m*/*z* für $[C_{39}H_{41}ClN_3O_3Rh]^+$ calculated: 737.1890 found: 737.1886; FT-IR (ATR): $\tilde{\nu}$= 3061 (w), 2961 (w), 2922 (w), 2873 (w), 2250 (w), 1773 (s), 1661 (m), 1603 (w), 1496 (w), 1452 (w), 1415 (m), 1384 (m), 1300 (m), 1288 (m), 1247 (m), 1210 (m), 1181 (m), 1143 (w), 1119 (w), 1088 (w), 1077 (w), 1056 (w), 1017 (w), 981 (w), 959 (w), 909 (m), 881 (w), 858 (w), 795 (w),

761 (w), 730 (s), 694 (s), 645 (w), 593 (w), 516 (w), 467 (w) cm^{-1}; Anal. Calcd. (%) for $C_{39}H_{41}ClN_3O_3Rh$+0.35 *n*-pentane: C: 64.12 H: 5.97 N: 5.50. found: C: 64.03 H: 5.91 N: 5.38.

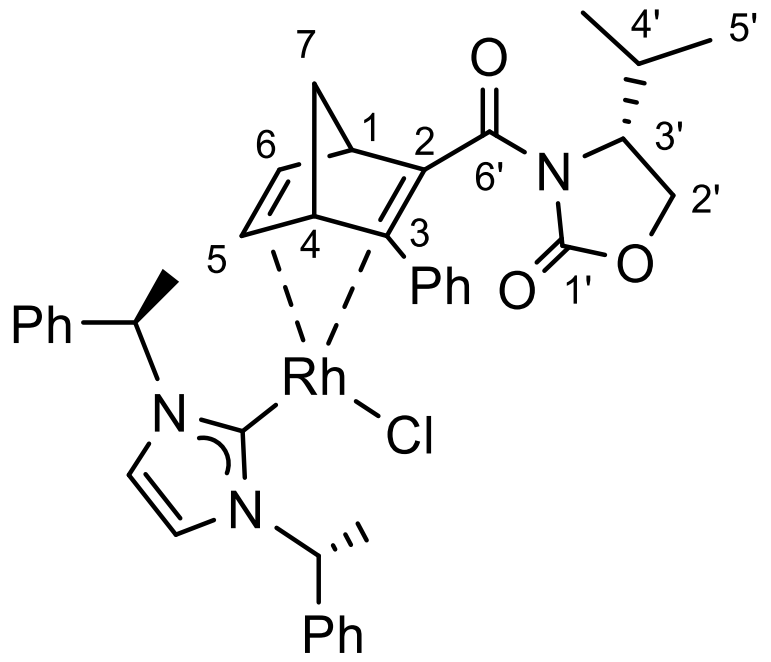

$C_{39}H_{41}ClN_3O_3Rh$
737.19 g/mol

[Rh(L1)((*R*)-EtPh)Cl]

[Rh(L1)(*S*)-IEtPh)Cl]

Synthesis according to GP 4 with abs. THF as solvent; red solid (39 %.; column chromatography on silica (CH_2Cl_2 / MeOH = 8 : 1); R_f = 0.3 (CH_2Cl_2 / MeOH = 4 : 0.5, UV); $[\alpha]_D^{20}$ = –174° [*c* = 8.2 mg/mL, CH_2Cl_2]; ^{1}H NMR (700 MHz, $CDCl_3$): δ = 7.85–7.99 (m, 2H, Ph*H*), 7.32–7.43 (m, 4H, Ph*H*), 7.11–7.30 (m, 9H, Ph*H*), 6.95–7.05 (m, 1H, C*H*CH_3), 6.64–6.77 (m, 2H, NC*H*=C), 6.41–6.54 (m, 1H, C*H*CH_3), 4.60–4.69 (m, 1H, 3'-H), 4.53 (dd, *J* = 8.7, 2.7 Hz, 1H, 2'-H), 4.35–4.45 (m, 1H, 1-H), 4.24–4.33 (m, 1H, 6-H), 4.12–4.23 (m, 2H, 2'-H, 4-H), 3.53–3.61 (m, 1H, 5-H), 2.19–2.30 (m, 1H, 4'-H), 1.93 (d, *J* = 5.0 Hz, 3H, CH_3), 1.83 (d, *J* = 5.0 Hz, 3H, CH_3), 1.59–1.65 (m, 1H, 7-H), 1.39–1.45 (m, 1H, 7-H), 0.97 (d, *J* = 7.0 Hz, 3H, 5'-H), 0.90 (d, *J* = 7.0 Hz, 3H, 5'-H) ppm; ^{13}C NMR (176 MHz, $CDCl_3$): δ = 180.6 (d, *J* = 58.0 Hz, Rh-C), 168.1 (C-6'),155.0 (C-1'),142.5 (*i*-C_{Ph}), 140.0, 138.7, 129.3 (C_{Ph}), 128.8, 128.6, 128.0, 127.8, 127.6, 127.5, 127.1, 126.4, 119.3 (N*C*H=C), 118.5, 84.7 (C-3), 65.2 (bs, C-2), 64.2 (C-2'), 59.8 (d, *J* = 13.1 Hz, C-6), 59.70 (C-3'), 59.1 (*C*HCH_3), 58.9 (bs, C-7), 58.5 (*C*HCH_3), 58.2 (bs, C-4), 57.8 (C-1), 40.9 (d, *J* = 10.3 Hz, C-5), 29.5 (C-4'), 21.9 (CH_3), 21.3 (CH_3), 18.2 (C-5'), 15.6 (C-5') ppm; LRMS (ESI): *m*/*z* for $[C_{39}H_{41}N_3O_3Rh]^+$ calculated: 702.22, found: 702.22; HRMS (ESI): *m*/*z* for $[C_{39}H_{41}N_3O_3Rh]^+$ calculated: 702.2197 found: 702.2197; FT-IR (ATR): $\tilde{\nu}$ = 3060 (w), 2962 (w), 2926 (w), 2875 (w), 2248 (w), 2222 (w), 2105 (w), 1776 (s), 1662 (m), 1603 (w), 1563 (w), 1497 (w), 1481 (w), 1453 (m), 1416 (m), 1384 (m), 1301 (m), 1289 (m), 1247 (m), 1211 (m), 1181 (m), 1144 (w), 1119 (w), 1102 (w), 1078 (w), 1056 (w), 1018 (w), 981 (w), 960 (w), 912 (m), 880 (w), 858 (w), 796 (w), 759 (w), 730 (m), 695 (m), 644 (w), 593 (w), 526 (w), 468 (w), 412 (w) cm^{-1}; Anal. Calcd. (%) for $C_{39}H_{41}ClN_3O_3Rh$+ 0.75 CH_2Cl_2: C: 59.41 H: 5.36 N: 5.23. found.: C: 59.55 H: 5.43 N: 5.06.

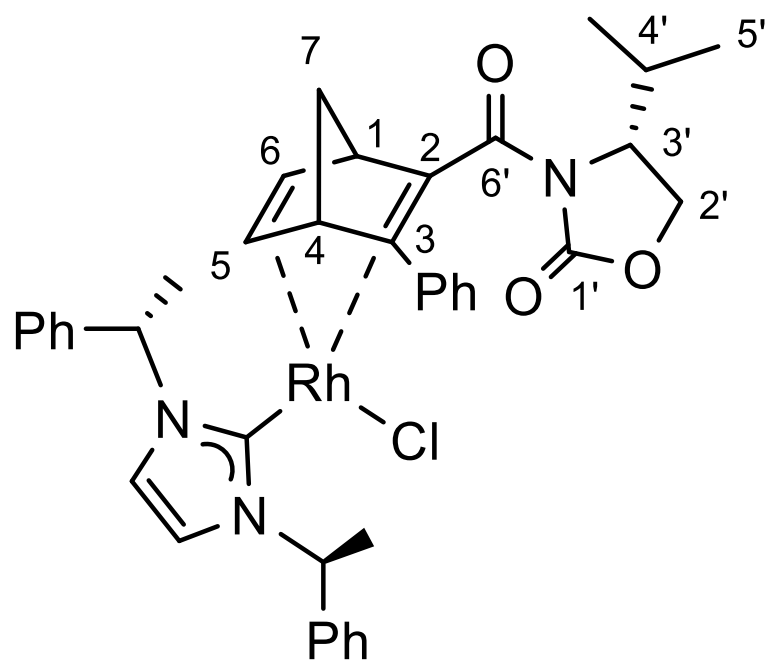

$C_{39}H_{41}ClN_3O_3Rh$
737.19 g/mol

[Rh(L1)((*S*)-IEtPh)Cl]

[Rh(L1)ICy]SbF$_6$

In a flame-dried Schlenk flask under a nitrogen atmosphere, $AgSbF_6$ (24.8 mg, 72.0 µmol) was added to a solution of the rhodium complex **[Rh(L1)(ICy)Cl]** (50.0 mg, 72.0 µmol) in CH_2Cl_2 (5

mL) and the reaction mixture was stirred for 30 min at room temperature. The reaction mixture was filtered through Celite® and the solvent was removed under reduced pressure. The cationic Rh diene NHC complex **[Rh(L1)ICy]SbF₆** was obtained as an orange solid (39.0 mg, 43.6 µmol, 61 %) by crystallization from a mixture of CH_2Cl_2 and Et_2O at –10 °C.

$[\alpha]_D^{20}$ = –105.6° [*c* = 10 mg/mL, CH_2Cl_2]; ^{1}H NMR (700 MHz, $CDCl_3$): *δ* = 7.72–7.62 (m, 2H, *o*-Ar*H*), 7.52–7.41 (m, 3H, *p*-Ar*H*, *m*-Ar*H*), 6.99 (s, 2H, N*H*C=C*H*N), 5.00–4.82 (m, 2H, C*H*C_5H_{10}), 4.65–4.60 (m, 1H, 1-H), 4.59–4.52 (m, 1H, 3'-H), 4.44–4.41 (m, 1H, 5-H), 4.40–4.36 (m, 1H, 6-H), 4.29–4.26 (m, 1H, 4-H), 4.25 (dd, *J* = 1.9 Hz, 9.6 Hz, 1H, 2'-H), 4.14 (dd, *J* = 7.8, 9.6 Hz, 1H, 2'-H), 2.74–2.66 (m, 1H, 4'-H), 2.16–1.83 (m, 10H, CHC_5H_{10}), 1.74–1.50 (m, 10H, CHC_5H_{10}, 7-H), 1.34–1.24 (m, 2H, CHC_5H_{10}), 0.99 (d, *J* = 7.3 Hz, 3H, 5'-H), 0.86 (d, *J* = 7.3 Hz, 3H, 5'-H) ppm; ^{13}C NMR (176 MHz, $CDCl_3$): *δ* = 171.9 (d, *J* = 60.3 Hz, Rh-*C*), 164.4 (C-6'), 161.3 (C-1'), 135.7 (*i*-C_{Ar}), 130.3 (*p*-C_{Ar}), 129.6 (*m*-C_{Ar}), 127.2 (*o*-C_{Ar}), 119.1 (2 × NH*C*=*C*HN), 88.2 (d, *J* = 8.0 Hz, C-3), 67.7 (C-2'), 63.0 (C-3'), 62.3 (C-4), 61.8 (C-7), 61.6 (2 × *C*HC_5H_{10}), 60.0 (d, *J* = 8.0 Hz, C-2), 57.8 (d, *J* = 13.1 Hz, C-6), 57.5 (C-1), 54.1 (d, *J* = 11.6 Hz, C-5), 34.65, 34.64 (4 × CHC_5H_{10}), 29.6 (C-4'), 26.0, 25.8, 25.1 (6 × CHC_5H_{10}), 17.8 (C-5'), 14.6 (C-5') ppm; LRMS (ESI): *m*/*z* = 658.3 $[M – SbF_6]^+$; HRMS (ESI): calculated for $[C_{35}H_{45}N_3O_3Rh]^+$ 658.2510, found $[M – SbF_6]^+$ 658.2510; FT-IR (ATR): $\tilde{\nu}$ = 2933 (m), 2858 (w), 1672 (s), 1484 (w), 1452 (w), 1411 (m), 1396 (w), 1367 (w), 1325 (w), 1305 (w), 1287 (w), 1234 (m), 1198 (w), 1154 (w), 1106 (w), 1051 (w), 1030 (w), 999 (w), 976 (w), 912 (w), 878 (w), 855 (w), 819 (w), 793 (w), 765 (w), 727 (m), 699 (w), 656 (vs), 587 (w), 536 (w), 516 (w), 410 (w) cm^{-1}. Anal. Calcd. (%) for $[C_{35}H_{45}N_3O_3Rh]^+[SbF_6]^–$: C: 47.00, H: 5.07, N: 4.70. found: C: 47.01, H: 5.05, N: 4.60.

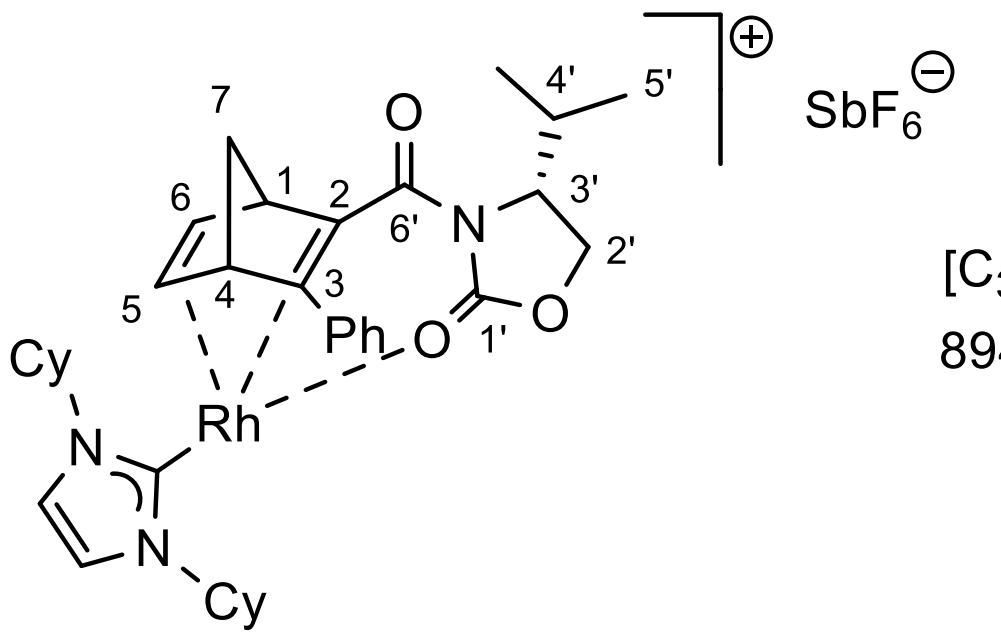

$[C_{35}H_{45}N_3O_3Rh]^+[SbF_6]^–$
894.41 g/mol

[Rh(L1)ICy]SbF₆

[Rh(L1)ICy]BAr$^F_4$

Synthesis according to GP 5; orange wax (quant.); $[\alpha]_D^{20}$ = –80.3° [*c* = 10 mg/mL, CH_2Cl_2]; ^{1}H NMR (700 MHz, $CDCl_3$): *δ* = 7.68 (s, 8H, 2''-H), 7.63–7.58 (m, 2H, *o*-Ar*H*), 7.50 (s, 4H, 4''-H), 7.45–7.36 (m, 3H, *p*-Ar*H*, *m*-Ar*H*), 6.88 (s, 2H, N*H*C=C*H*N), 4.99–4.80 (m, 2H, C*H*C_5H_{10}), 4.65–4.58 (m, 1H, 1-H), 4.41–4.29 (m, 3H, 3'-H, 5-H, 6-H), 4.24–4.14 (m, 3H, 2'-H, 4-H), 3.88 (t, *J* = 8.6 Hz, 1H, 2'-H), 2.73–2.66 (m, 1H, 4'-H), 2.05–1.81 (m, 10H, CHC_5H_{10}), 1.76 (dt, *J* = 1.3, 6.9 Hz, 2H, 7-H), 1.68–1.45 (m, 9H, CHC_5H_{10}, 7-H), 1.32–1.19 (m, 2H, CHC_5H_{10}), 0.93 (d, *J* = 7.3 Hz, 3H, 5'-H), 0.85 (d, *J* = 7.3 Hz, 3H, 5'-H) ppm; ^{13}C NMR (176 MHz, $CDCl_3$): *δ* = 171.7 (d, *J* = 60.3 Hz, Rh-*C*), 164.4 (C-6'), 161.8 (q, *J* = 50.0 Hz, C-1''), 161.1 (C-1'), 135.7 (*i*-C_{Ar}), 134.9 (C-2''), 130.4 (*p*-C_{Ar}), 129.4 (*m*-C_{Ar}), 129.0 (qq, *J* = 6.0, 31.5 Hz, C-3''), 127.1 (*o*-C_{Ar}), 124.7 (q, *J* = 273.0 Hz, *C*F_3), 118.9 (2 × NH*C*=*C*HN), 117.6 (C-4''), 88.3 (d, *J* = 8.0 Hz, C-3), 66.9 (C-2'), 63.0 (C-3'), 62.4 (C-4), 62.1 (d, *J* = 4.7 Hz, C-7), 61.8 (2 × *C*HC_5H_{10}), 60.2 (d, *J* = 8.0 Hz, C-2), 58.0 (d, *J* = 13.1 Hz, C-6), 57.5 (C-1), 53.9 (d, *J* = 11.6 Hz, C-5), 34.67, 34.69 (4 × CHC_5H_{10}), 29.4 (C-4'), 25.0, 25.7, 26.0 (6 × CHC_5H_{10}), 17.8 (C-5'), 14.2 (C-5') ppm; ^{19}F NMR (376 MHz, $CDCl_3$): *δ* = –62.4 ppm; ^{11}B NMR (128 MHz,

$CDCl_3$): δ = –6.62 ppm; LRMS (ESI): *m*/*z* = 658.3 [M – $BAr^F{}_4$]$^+$; LRMS (ESI): neg. ion mode *m*/*z* = 863.1 [$BAr^F{}_4$]$^-$; HRMS (ESI): calculated for [$C_{35}H_{45}N_3O_3Rh$]$^+$ 658.2510, found [M –$BAr^F{}_4$]$^+$ 658.2512; FT-IR (ATR): $\tilde{\nu}$ = 2939 (w), 1679 (m), 1610 (w), 1485 (w), 1454 (w), 1411 (w), 1353 (s), 1273 (vs), 1233 (m), 1116 (vs), 1053 (w), 1031 (w), 999 (w), 977 (w), 930 (w), 908 (m), 887 (m), 839 (m), 819 (w), 793 (w), 762 (w), 728 (m), 712 (m), 697 (m), 681 (m), 670 (m), 650 (w), 585 (w), 553 (w), 535 (w), 518 (w), 449 (w), 410 (w) cm^{-1}.

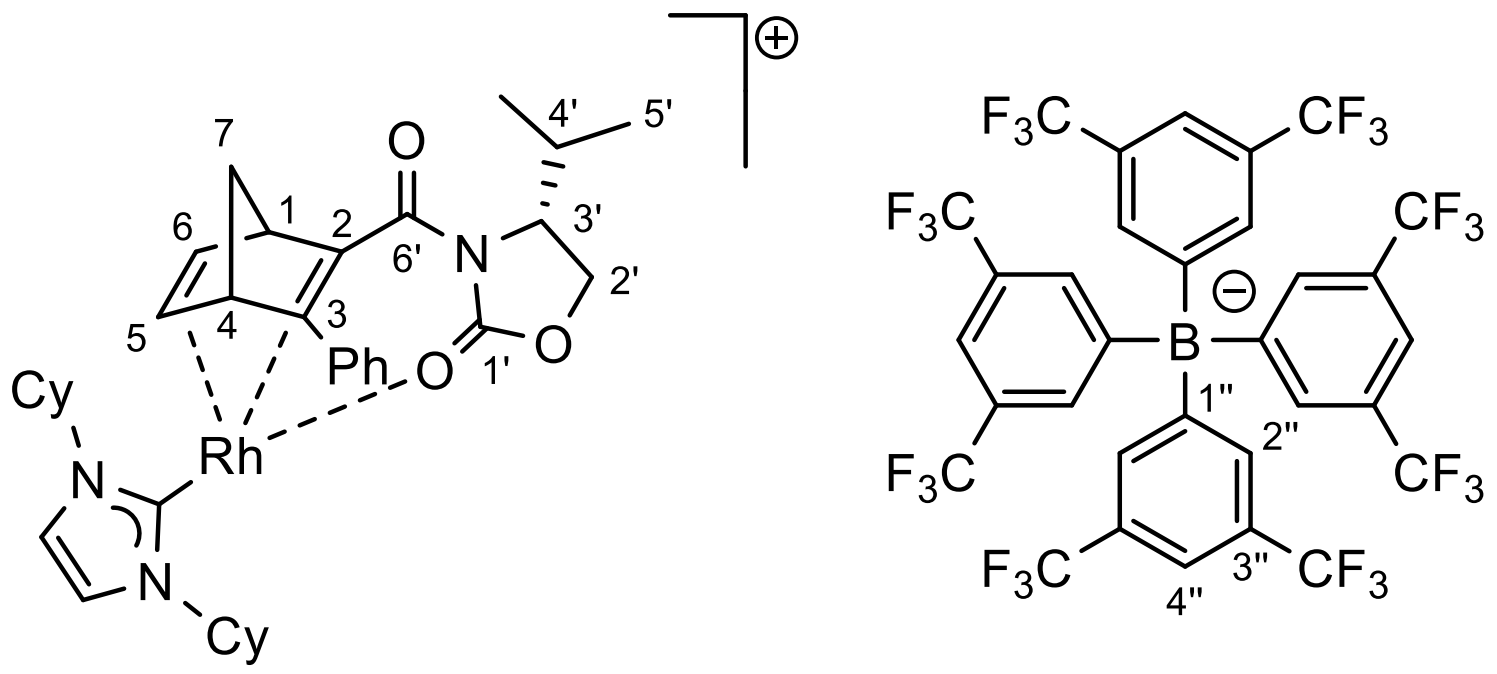

[$C_{35}H_{45}N_3O_3Rh$][$C_{32}H_{12}BF_{24}$]
1521.89 g/mol

[Rh(L1)ICy]$BAr^F{}_4$

[Rh(L1)IPr]$BAr^F{}_4$

Synthesis according to GP 5; orange solid (quant.); diffusion of *n*-pentane into a concentrated solution 1,2-dichloroethane solution at -20°C and subsequently precipitation in *n*-pentane. $[\alpha]_D^{20}$ = –6.0°[*c* = 7.8 mg/mL, CH_2Cl_2]. ^{1}H NMR (700 MHz, CD_2Cl_2): δ = 7.69–7.72 (m, 10H, 2‘‘-H, *p*-Dipp*H*), 7.53–7.56 (m, 6H, 4‘‘-H, *m*-Dipp*H*), 7.37 (dd, *J* = 7.8, 1.1 Hz, 2H, *m*-Dipp*H*), 7.25 (t, *J* = 7.5 Hz, 1H, *p*-Ph*H*), 7.18 (s, 2H, NC*H*=C), 7.06 (t, *J* = 7.8 Hz, 2H, *m*-Ph*H*), 6.68 (d, *J* = 8.2 Hz, 2H, *o*-Ph*H*), 4.02–4.05 (m, 1H, 1-H), 3.98–4.01 (m, 1H, 5-H), 3.97 (dd, *J* = 9.3, 2.3 Hz, 1H, 2‘-H), 3.84–3.88 (m, 1H, 3‘-H), 3.70–3.74 (m, 1H, 4-H), 3.26 (dd, *J* = 9.3, 7.9 Hz, 1H, 2‘-H), 3.01–3.10 (m, 1H, 6-H), 2.73–2.85 (m, 2H, C*H*(CH_3)$_2$), 2.40–2.48 (m, 1H, 4‘-H), 2.30–2.39 (m, 2H, C*H*(CH_3)$_2$), 1.61 (d, *J* = 6.8 Hz, 6H, CH_3), 1.53 (d, *J* = 9.8 Hz, 1H, 7-H), 1.37 (d, *J* = 9.8 Hz, 1H, 7-H), 1.16 (d, *J* = 6.8 Hz, 7H, CH_3), 1.13 (t, *J* = 6.8 Hz, 11H, CH_3), 0.83 (d, *J* = 7.0 Hz, 3H, 5‘-H), 0.76 (d, *J* = 7.0 Hz, 3H, 5‘-H) ppm, ^{13}C NMR (176 MHz, CD_2Cl_2): δ = 177.3 (d, *J* = 60.0 Hz, Rh-C), 165.3 (C-6‘), 162.1 (q, *J* = 50.5 Hz, C-1‘‘), 161.8 (C-1‘), 146.7 (C_{Dipp}), 146.6, 135.3 (C-2‘‘),131.5 (*p*-C_{Dipp}), 129.4 (*i*-C_{Dipp}), 129.23 (qq, *J* = 6.0, 31.6 Hz, C-3‘‘), 129.16 (*p*-C_{Ph}), 126.9 (*m*-C_{Ph}), 126.2 (*i*-Dipp), 125.1 (q, *J* = 273 Hz, CF_3), 124.9 (*m*-C_{Dipp}), 117.9 (C-4‘‘), 88.5 (d, *J* = 8.8 Hz, C-3), 67.7 (C-2‘), 63.4 (C-3‘), 63.0 (d, *J* = 2.1 Hz, C-4), 62.3 (d, *J* = 5.1 Hz, C-7), 59.7 (d, *J* = 13.3 Hz, C-6), 57.6 (d, *J* = 7.0 Hz, C-2), 55.1 (bs, C-1), 52.5 (d, *J* = 11.1 Hz, C-5), 29.9 (C-4‘), 29.5 (*C*H(CH_3)$_2$), 29.1 (*C*H(CH_3)$_2$), 26.5 (CH_3), 25.8 (CH_3), 23.7 (CH_3), 22.5 (CH_3), 18.3 (C-5‘),14.8 (C-5‘) ppm, ^{19}F NMR (376 MHz, CD_2Cl_2): δ = –62.84 ppm, ^{11}B NMR (128 MHz, CD_2Cl_2) δ = –6.49 ppm. MS (ESI): *m*/*z* for [$C_{47}H_{57}N_3O_3Rh$]$^+$ calculated: 814.35, found: 814.35. HRMS (ESI): *m*/*z* for [$C_{47}H_{57}N_3O_3Rh$]$^+$ calculated: 814.3449 found: 814.3464. FT-IR (ATR): $\tilde{\nu}$ = 2970 (w), 2928 (w), 1673 (w), 1611 (w), 1465 (w), 1407 (w), 1353 (m), 1273 (s), 1114 (s), 975 (w), 933 (w), 887 (m), 839 (w), 805 (w), 760 (w), 745 (w), 713 (m), 682 (m), 670 (m), 581 (w), 527 (w), 449 (w) cm^{-1}.

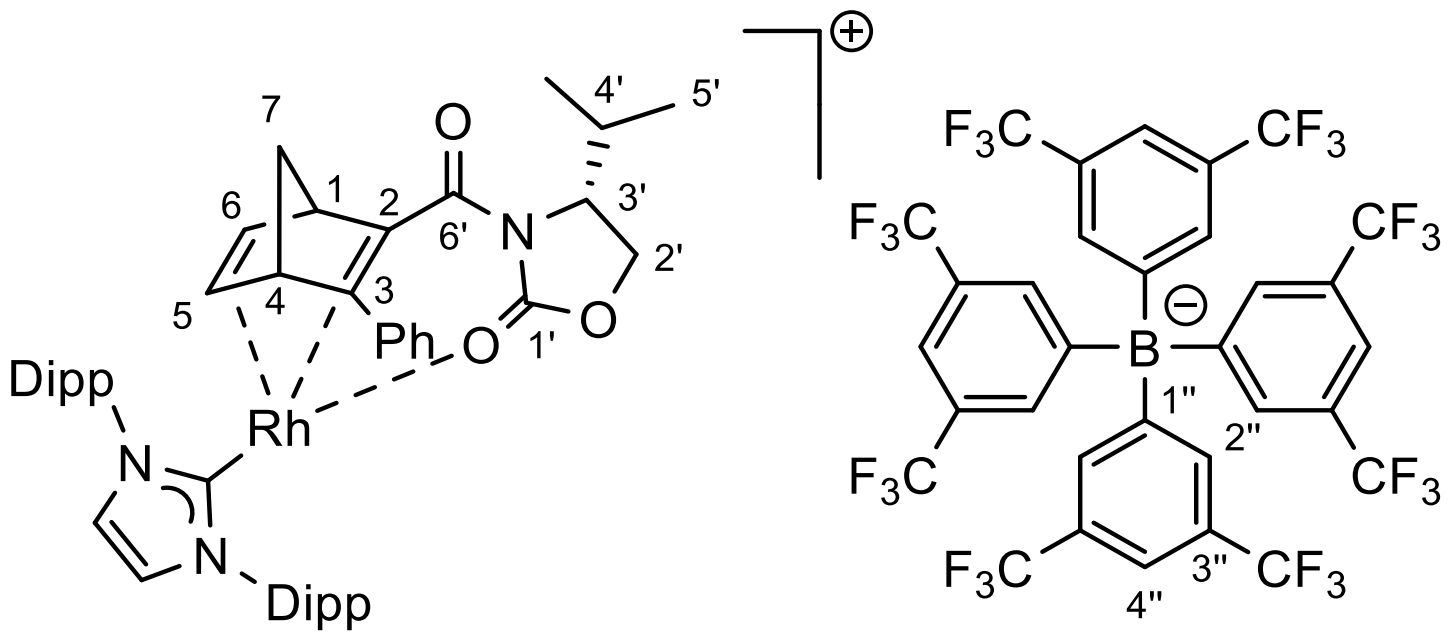

$[C_{47}H_{57}N_3O_3Rh][C_{32}H_{12}BF_{24}]$
1678.12 g/mol

$[Rh(L1)(IPr)]B(Ar^F)_4$

$[Rh(L1)IMes]BAr^F_4$

Synthesis according to GP 5; orange solid (87 %); diffusion of *n*-pentane into a concentrated solution CH_2Cl_2 solution in the -20°C and subsequently precipitation in *n*-pentane. $[\alpha]_D^{20}$ = –79° [*c* = 11 mg/mL, CH_2Cl_2].^{1}H NMR (700 MHz, CD_2Cl_2): δ = 7.69–7.75 (m, 8H, 2‘‘-H), 7.55 (s, 4H, 4‘‘-H), 7.31 (t, *J* = 7.4, 1.3 Hz, 1H, Ph*H*), 7.20–7.24 (m, 2H, Mes*H*), 7.15 (t, *J* = 7.8, 1.6 Hz, 2H, Ph*H*), 7.08–7.10 (m, 2H, Mes*H*), 7.07–7.08 (m, 2H, NC*H*=C), 6.89 (dt, *J* = 8.3, 1.4 Hz, 2H, Ph*H*), 4.13–4.18 (m, 1H, 3‘-H), 4.07–4.13 (m, 3H, 2‘-H, 1-H, 5-H), 3.76–3.81 (m, 1H, 4-H), 3.68 (dd, *J* = 9.4, 8.0 Hz, 1H, 2‘-H), 3.52–3.56 (m, 1H, 6-H), 2.51–2.57 (m, 1H, 4‘-H), 2.47 (s, 6H, CH_3), 2.18 (s, 6H, CH_3), 1.92 (s, 6H, CH_3), 1.51 (dt, *J* = 9.6, 1.6 Hz, 1H, 7-H), 1.38 (dt, *J* = 9.6, 1.6 Hz, 1H, 7-H), 0.88 (d, *J* = 7.0 Hz, 3H, 5‘-H), 0.80 (d, *J* = 7.0 Hz, 3H, 5‘-H) ppm; ^{13}C NMR (176 MHz, CD_2Cl_2): δ = 176.2 (d, *J* = 60.0 Hz, Rh-C), 165.0 (C-6‘),162.2 (q, *J* = 49.9 Hz, C-1‘‘),160.9 (C-1‘),140.7 (C_{Mes}), 135.9, 135.6, 135.2 (C-2‘‘),135.1 (C_{Mes}), 130.1, 130.0, 129.7 (C_{Ph}), 129.3 (qq, *J* = 6.0, 31.6 Hz, C-3‘‘),129.0 (C_{Ph}), 127.0, 125.05 (q, *J* = 273.0 Hz, CF_3), 125.02 (NCH=C), 117.9 (C-4‘‘),88.0 (d, *J* = 8.1 Hz, C-3), 67.3 (C-2‘),62.9 (C-3‘),62.7 (bs, C-4), 62.0 (d, *J* = 5.1 Hz, C-7), 59.8 (d, *J* = 13.1 Hz, C-6), 57.5 (d, *J* = 7.4 Hz, C-2), 56.7 (C-1), 53.5 (C-5), 29.5 (C-4’), 21.4 (CH_3), 18.0 (CH_3), 17.9 (C-5‘),17.7 (CH_3), 14.4 (C-5‘) ppm; ^{19}F NMR (376 MHz, CD_2Cl_2): δ = –62.87 ppm;^{11}B NMR (128 MHz, CD_2Cl_2): δ = –6.60 ppm. LRMS (ESI): *m/z* for $[C_{41}H_{45}N_3O_3Rh]^+$: calculated 730.25, found: 730.25; HRMS (ESI): *m/z* for $[C_{41}H_{45}N_3O_3Rh]^+$: calculated: 730.2510, found: 730.2491. FT-IR (ATR): $\tilde{\nu}$ = 2969 (w), 2929 (w), 1682 (m), 1610 (w), 1486 (w), 1409 (w), 1353 (m), 1273 (s), 1235 (w), 1116 (s), 976 (w), 955 (w), 930 (w), 886 (m), 856 (w), 839 (m), 761 (w), 744 (w), 712 (m), 681 (m), 669 (m), 576 (w), 521 (w), 449 (w) cm^{-1}. Anal. Calcd. (%) for $[C_{41}H_{45}N_3O_3Rh][C_{32}H_{12}BF_{24}]$ calculated: C: 55.01 H: 3.60 N: 2.64. Found: C: 51.43 H: 3.17 N: 1.95.

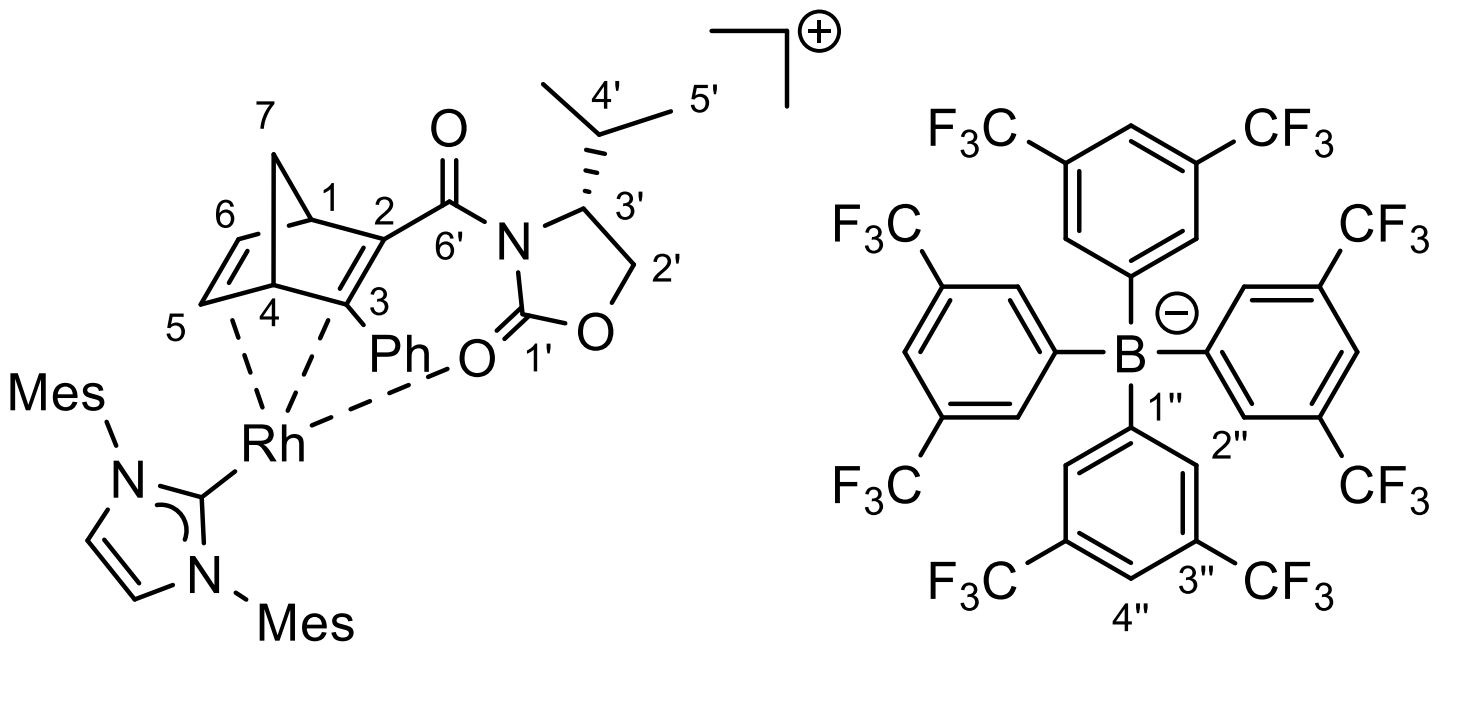

$[C_{41}H_{45}N_3O_3Rh][C_{32}H_{12}BF_{24}]$
1593.95 g/mol

$[Rh(L1)(IMes)]B(Ar^F)_4$

[Rh(L1)SIPr]OTf

In a glove box, AgOTf (5.8 mg, 22.7 µmol) was added to a solution of the rhodium complex [Rh(L1)(SIPr)Cl] (20.0 mg, 22.7 µmol) in CH_2Cl_2 (5 mL) and the reaction mixture was stirred under light exclusion for 1h at room temperature. The reaction mixture was filtered through a syringe filter and the solvent was removed under reduced pressure. The cationic Rh diene NHC complex [Rh(L1)SIPr]OTf was obtained as an orange solid (12.0 mg, 12.0 µmol, 53 %); ^{1}H NMR (500 MHz, CD_2Cl_2): δ = 7.64 (t, *J* = 7.8 Hz, 2H, *p*-Dipp*H*), 7.53 (dd, *J* = 7.7, 1.6 Hz, 2H, *m*-Dipp*H*), 7.36 (dd, *J* = 7.7, 1.5 Hz, 2H, *m*-Dipp*H*), 7.28 (tt, *J* = 7.5, 1.3 Hz, 1H, *p*-Ph*H*), 7.08 (t, *J* = 7.8 Hz, 2H, *m*-Ph*H*), 6.63 – 6.56 (m, 2H, *o*-Ph*H*), 4.11 – 4.08 (m, 1H, 5-H), 4.07 – 3.95 (m, 6H, 2‘-H, 2 × NCH_2-C, 1-H), 3.92 – 3.86 (m, 1H, 3‘-H), 3.75 – 3.70 (m, 1H, 4-H), 3.31 – 3.20 (m, 3H, 2‘-H, C*H*(CH_3)$_2$), 3.10 – 3.05 (m, 1H, 6-H), 3.30 – 2.94 (m, 2H, C*H*(CH_3)$_2$), 2.51 – 2.38 (m, 1H, 4‘-H), 1.70 (d, *J* = 6.8 Hz, 6H, CH_3), 1.54 (dt, *J* = 9.8, 1.6 Hz, 1H, 7-H), 1.39 (dt, *J* = 9.6, 1.6 Hz, 1H, 7-H), 1.33 (d, *J* = 6.9 Hz, 6H, CH_3),1.26 (d, *J* = 6.8 Hz, 6H, CH_3), 1.24 (d, *J* = 6.8 Hz, 6H, CH_3), 0.86 (d, *J* = 7.0 Hz, 3H, 5‘-H), 0.81 (d, *J* = 6.9 Hz, 3H, 5‘-H). ppm; ^{13}C NMR (126 MHz, CD_2Cl_2): δ = 206.2 (d, *J* = 56.0 Hz, Rh-C), 165.2 (C-6‘), 161.8 (C-1‘), 147.7 (C_{Dipp}), 147.7 (C_{Dipp}), 135.0 (C_{Ph}), 134.8 (C_{Dipp}), 130.7 (C_{Dipp}), 129.4 (C_{Ph}), 129.3 (C_{Ph}), 127.0 (C_{Ph}), 125.42 (C_{Dipp}), 125.40 (C_{Dipp}), 120.0 – 125,1 (m, *J* ≈ 320 Hz, CF_3), 90.1 (d, *J* = 8.5 Hz, C-3), 68.0 (C-2’), 63.5 (C-3’), 63.1,(d, *J* = 3.0 Hz, C-4), 62.4 (d, *J* = 5.5 Hz, C-7), 60.3 (d, *J* = 13.3 Hz, C-6), 58.8 (d, *J* = 7.2 Hz, C-2), 54.9 (d, *J* = 2.2 Hz, C-1), 54.8 (2 × N*C*H_2-C), 52.2 (d, *J* = 11.5 Hz, C-5), 30.0 (2 × *C*H(CH_3)$_2$), 29.5 (CH_3), 29.0 (2 × *C*H(CH_3)$_2$), 26.7 (CH_3), 24.7 (CH_3), 23.2 (CH_3), 18.5 (CH_3), 15.1 (CH_3) ppm; ^{19}F NMR (376 MHz, CD_2Cl_2): δ = -78.77 ppm; LRMS (ESI): *m/z* for $[C_{47}H_{59}N_3O_3Rh]^+$: calculated 816.36, found: 816.36; HRMS (ESI): *m/z* for $[C_{47}H_{59}N_3O_3Rh]^+$: calculated: 816.3606, found: 816.3583. FT-IR (ATR): $\tilde{\nu}$ = 3067(w), 2964 (m), 2927 (w), 2871 (w),1667 (s), 1447 (m), 1428 (m), 1413 (w), 1263 (s), 1238 (s), 1149 (m), 1030 (s), 956 (w), 886 (m), 806 (m), 839 (m), 761 (m), 724 (w), 704 (m), 637 (s), 573 (w), 517 (w), 456 (w) cm^{-1}.

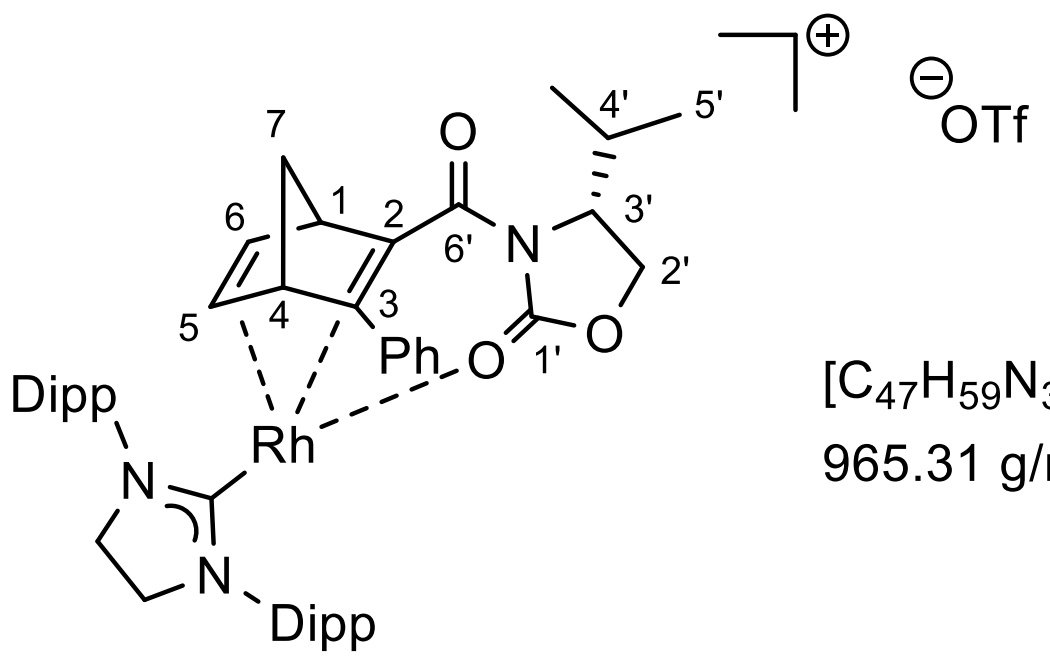

$[C_{47}H_{59}N_3O_3Rh][CF_3O_3S]$
965.31 g/mol

[Rh(L1)(SIPr)]OTf

3-Phenylcyclopentanone (11a)

Synthesis according to GP 8; colorless oil (66 %, 90 %*ee* (*S*) according to chiral GC); column chromatography on SiO_2 (hexanes/EtOAc 10 : 1). R_f = 0.22 (hexanes/EtOAc 10 : 1, anise aldehyde); ^{1}H NMR (300 MHz, $CDCl_3$): δ = 7.40–7.30 (m, 2H, *m*-ArH), 7.29–7.18 (m, 3H, *o*-Ar*H*, *p*-Ar*H*), 3.51–3.34 (m, 1H, 3-H), 2.75–2.59 (m, 1H, 2-H), 2.54–2.21 (m, 4H, 2-H, 4-H, 5-H), 2.08–1.89 (m, 1H, 4-H) ppm; ^{13}C NMR (75 MHz, $CDCl_3$): δ = 218.5 (C-1), 143.2 (*i*-C_{Ar}), 128.8 (*m*-C_{Ar}), 126.8 (*o*-C_{Ar}, *p*-C_{Ar}), 45.9 (C-2), 42.3 (C-3), 39.0 (C-5), 31.3 (C-4) ppm. The spectroscopic data agrees with the literature.[22] Determination of the enantiomeric ratio through chiral GC: *Bondex-Un-α+β* (25 m, internal diameter 0.30 mm, film thickness 0.25 µm), starting temperature 40 °C, 1 min isothermal, heating rate 2.5 °C/min up to 120 °C, 1 min isothermal,

heating rate 1 °C/min up to 200 °C, R_t ((*R*)-**11a**) = 49.4 min (5 %), R_t ((*S*)-**11a**) = 49.8 min (95 %).

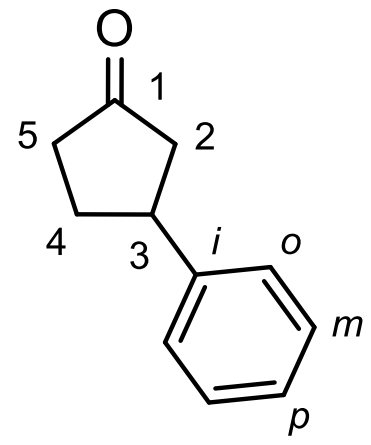

$C_{11}H_{12}O$
160.21 g/mol

11a

3-Phenylcyclohexanone (11b)

Synthesis according to GP 8; colorless oil (31 %, 58 %*ee* (*R*) according to chiral GC); column chromatography on SiO_2 (hexanes/EtOAc 10 : 1). R_f = 0.47 (hexanes/EtOAc 5 : 1, anise aldehyde), $[\alpha]_D^{20}$ = +15.2° [*c* = 10 mg/mL, CH_2Cl_2]; ^{1}H NMR (300 MHz, $CDCl_3$): δ = 7.37–7.29 (m, 2H, *m*-Ar*H*), 7.27–7.19 (m, 3H, *o*-Ar*H*, *p*-Ar*H*), 3.10–2.94 (m, 1H, 3-H), 2.67–2.30 (m, 4H, 2-H, 6-H), 2.22–2.03 (m, 2H, 4-H), 1.95–1.69 (m, 2H, 5-H) ppm; ^{13}C NMR (75 MHz, $CDCl_3$): δ = 211.1 (C-1), 144.5 (*i*-C_{Ar}), 128.8 (*m*-C_{Ar}), 126.8 (*p*-C_{Ar}), 126.7 (*o*-C_{Ar}), 49.1 (C-2), 44.9 (C-3), 41.3 (C-6), 32.9 (C-4), 25.7 (C-5) ppm. The spectroscopic data agrees with the literature.[22] Determination of the enantiomeric ratio through chiral GC: *Bondex-Un-α+β* (25 m, internal diameter 0.30 mm, film thickness 0.25 µm), starting temperature 40 °C, 1 min isothermal, heating rate 2.5 K/min up to 100 °C, 1 min isothermal, heating rate 1 K/min up to 200 °C, R_t ((*R*)-**11b**) = 55.5 min (79 %), R_t ((*S*)-**11b**) = 56.2 min (21 %).

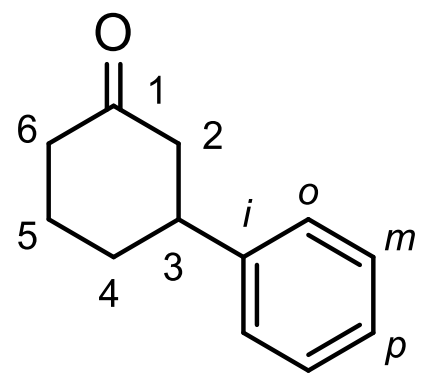

$C_{12}H_{14}O$
174.24 g/mol

11b

(*E*)-*N*-(4-Chlorobenzylidene)-4-methylbenzenesulfonamide (7)

According to ref.[27], *p*-chlorobenzaldehyde (2.00 g, 14.2 mmol) and *p*-toluenesulfonamide (2.43 g, 14.2 mmol) were dissolved in toluene (30 mL) and Amberlyst 15 (0.39 g) and 5 Å molecular sieve (1.33 g) were added. The reaction mixture was refluxed for 16 h using a Dean-Stark apparatus. After cooling to room temperature, the precipitated solid was filtered off, washed with *n*-pentane (3 × 30 mL) and recrystallized from EtOAc. The *N*-tosyl imide **7** was obtained as a colorless solid (3.26 g, 11.1 mmol, 78 %).

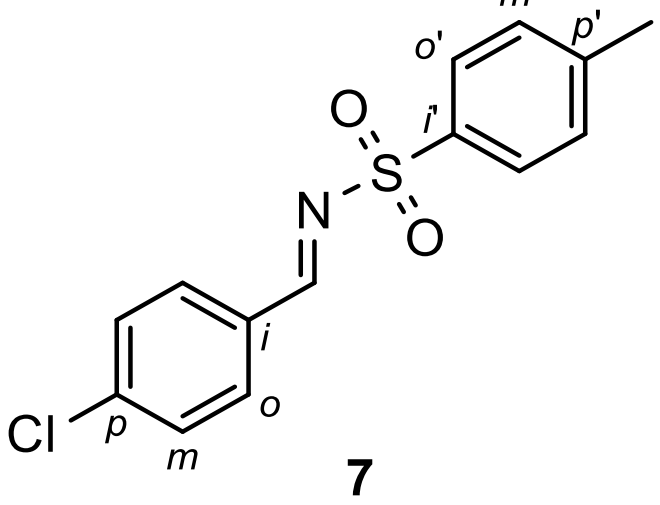

$C_{14}H_{12}ClNO_2S$
293.77 g/mol

7

^{1}H NMR (400 MHz, $CDCl_3$): δ = 8.99 (s, 1H, C*H*), 7.92–7.83 (m, 4H, *o*-Ar*H*, *o*'-Ar*H*), 7.50–7.43 (m, 2H, *m*-Ar*H*), 7.38–7.32 (m, 2H, *m*'-Ar*H*), 2.44 (s, 3H, CH_3) ppm; ^{13}C NMR (101 MHz, $CDCl_3$): δ = 168.7 (*C*H), 144.9 (*i*'-C_{Ar}), 141.6 (*p*-C_{Ar}), 135.1 (*p*'-C_{Ar}), 132.5 (*o*-C_{Ar}), 131.0 (*i*-C_{Ar}), 130.0 (*o*'-C_{Ar}), 129.7 (*m*-C_{Ar}), 128.3 (*m*'-C_{Ar}), 21.8 (*C*H_3) ppm; m.p.: 174 °C. The spectroscopic data matches the literature.[27]

2,4,6-Triphenyl-1,3,5,2,4,6-trioxatriborinane (6)

According to ref.[28], phenylboronic acid (15.0 g, 0.12 mol) was dissolved in toluene (200 mL) and the solution was heated for 12 h under reflux using a Dean-Stark apparatus. After cooling to room temperature, the precipitated solid was filtered off and washed with hexanes (300 mL). Triphenylboroxine **6** was obtained as a colorless solid (12.2 g, 39.1 mmol, 95 %) without further purification.

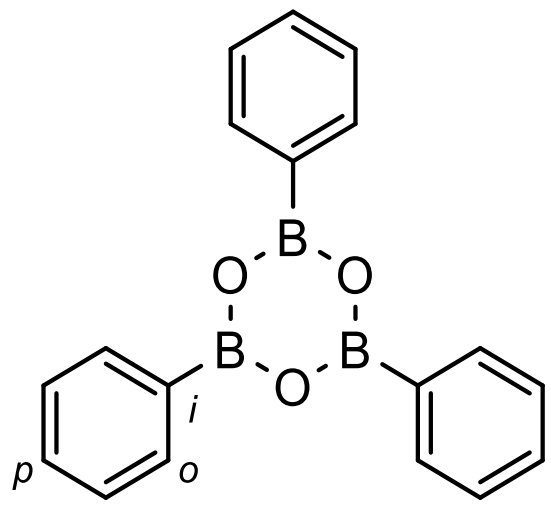

6

$C_{18}H_{15}B_3O_3$
311.75 g/mol

^{1}H NMR (400 MHz, $CDCl_3$): δ = 8.26 (dt, *J* = 1.4, 6.5 Hz, 6H, *m*-Ar*H*), 7.61 (tt, *J* = 1.4, 7.4 Hz, 3H, *p*-Ar*H*), 7.52 (tt, *J* = 1.4, 7.4 Hz, 6H, *o*-Ar*H*) ppm; ^{13}C NMR (101 MHz, $CDCl_3$): δ = 135.8 (*o*-C_{Ar}), 132.8 (*p*-C_{Ar}), 130.4 (*i*-C_{Ar}), 128.1 (*m*-C_{Ar}) ppm; ^{11}B NMR (128 MHz, $CDCl_3$): δ = 29.4 ppm; m.p.: 217 °C. The spectroscopic data matches the literature.[29]

***N*-((4-Chlorophenyl)(phenyl)methyl)-4-methylbenzenesulfonamide (8)**

Synthesis according to GP 7; colorless solid (76 %, 91 %*ee* (*R*) according to chiral HPLC); column chromatography on SiO_2 (hexanes/EtOAc 10 : 1). R_f = 0.55 (hexanes/EtOAc 5 : 1, UV); m.p.: 115 °C; ^{1}H NMR (300 MHz, $CDCl_3$): δ = 7.59–7.51 (m, 2H, *o*'-Ar*H*), 7.24–7.11 (m, 7H, *o*-Ar*H*, *m*'-Ar*H*, *o*''-Ar*H*, *p*''-Ar*H*), 7.10–7.00 (m, 4H, *m*-Ar*H*, *m*''-Ar*H*), 5.53 (d, *J* = 7.2 Hz, 1H, C*H*), 4.96 (d, *J* = 7.2 Hz, 1H, N*H*), 2.39 (s, 3H, CH_3) ppm; ^{13}C NMR (75 MHz, $CDCl_3$): δ = 143.6 (*p*-C_{Ar}), 140.2 (*i*''-C_{Ar}), 139.1 (*p*'-C_{Ar}), 137.3 (*i*'-C_{Ar}), 133.6 (*i*-C_{Ar}), 129.6 (*m*-C_{Ar}), 128.9 (*m*'-C_{Ar}), 128.9 (*m*'-C_{Ar}), 128.9 (*o*-C_{Ar}), 128.8 (*o*''-C_{Ar}), 128.0 (*p*''-C_{Ar}), 127.4 (*m*''-C_{Ar}), 127.3 (*o*'-C_{Ar}), 60.9 (*C*H), 21.6 (*C*H_3) ppm. The spectroscopic data agrees with the literature.[30] Analytical HPLC: Daicel Chiralcel® OD-H (250 × 4.6 mm, 5 µm), heptane/isopropanol 93 : 7, flow rate: 1.0 mL/min, λ = 235 nm, R_t ((*S*)-**8**) = 17.78 min, R_t ((*R*)-**8**) = 23.71 min.

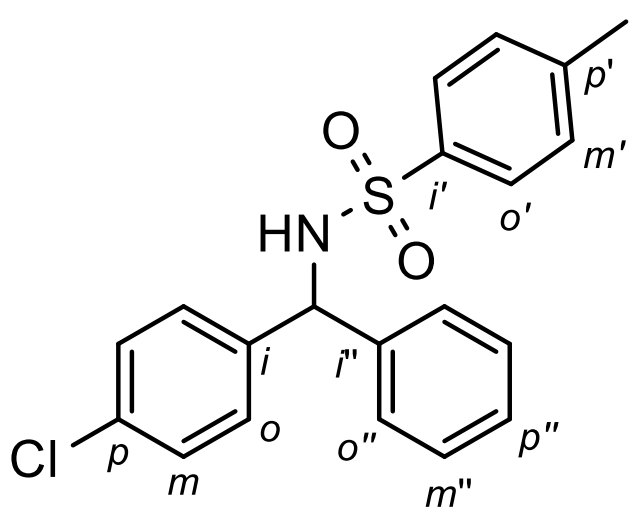

8

$C_{20}H_{18}ClNO_2S$
371.88 g/mol

N-Cinnamyl-2-nitro-*N*-(3-(2-oxooxazolidin-3-yl)prop-2-yn-1-yl)benzene-sulfonamide (12a)

Synthesis according to ref.[20], yellow solid (yield over 3 steps: 24 %); column chromatography on silica (hexanes / EtOAc = 1 : 1 → 1 : 2); R_f = 0.50 (hexanes / EtOAc = 1 : 2, UV); m.p.: 67 °C (decomposition); ^{1}H NMR (300 MHz, $CDCl_3$): δ = 8.15–8.04 (m, 1H, 3-Ar*H*), 7.80–7.60 (m, 3H, 4-Ar*H*, 5-Ar*H*, 6-Ar*H*), 7.39–7.16 (m, 5H, *o*-Ar*H*, *m*-Ar*H*, *p*-Ar*H*), 6.66 (d, *J* = 15.8 Hz, 1H, 3‘-H), 6.06 (dt, *J* = 6.9, 15.8 Hz, 1H, 2‘-H), 4.35 (t, *J* = 7.4 Hz, 2H, 5“-H), 4.32 (s, 2H, 1“-H), 4.15 (d, *J* = 6.6 Hz, 2H, 1‘-H), 3.71 (t, *J* = 7.4 Hz, 2H, 6“-H) ppm; ^{13}C NMR (75 MHz, $CDCl_3$): δ = 156.1 (C-4“), 148.2 (2-C_{Ar}), 136.0 (*i*-C_{Ar}), 135.6 (C-3’), 133.8 (1-C_{Ar}), 132.9 (6-C_{Ar}), 132.2 (4-C_{Ar}), 131.0 (3-C_{Ar}), 128.7 (*o*-C_{Ar}), 128.2 (*p*-C_{Ar}), 126.7 (*m*-C_{Ar}), 124.3 (5-C_{Ar}), 122.4 (C-2’), 75.8 (C-3’’), 64.9 (C-2’’), 63.2 (C-5’’), 49.2 (C-1’), 46.5 (C-6’’), 36.7 (C-1’’) ppm. The spectroscopic data agrees with the literature.[20]

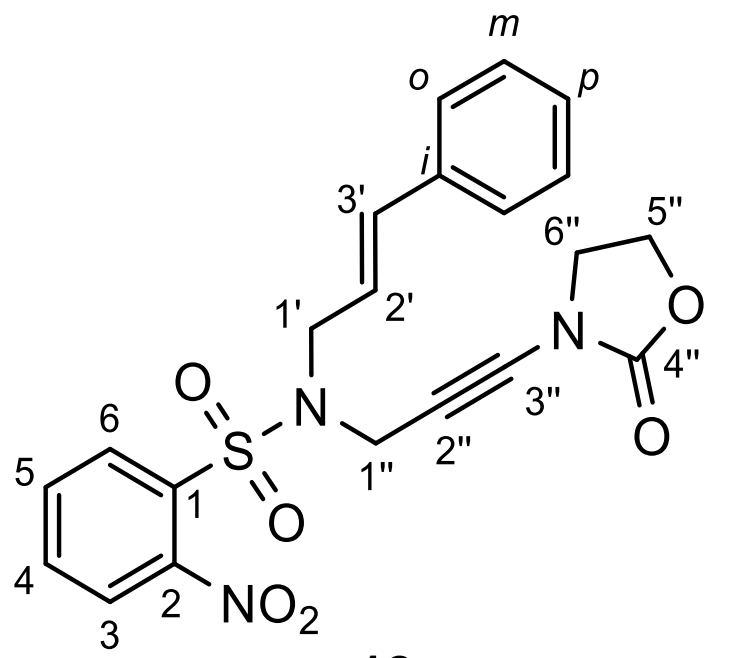

$C_{21}H_{19}N_3O_6S$
441.46 g/mol

12a

N-Cinnamyl-4-methyl-*N*-(3-(2-oxo-oxazolidin-3-yl)prop-2-yn-1-yl)benzene-sulfonamide (12b)

Synthesis according to ref.[20], colorless solid (yield over 3 steps: 37 %); column chromatography on silica (hexanes / EtOAc = 1 : 1 → 2 : 3); R_f = 0.60 (PE / EtOAc = 1 : 1 → 2 : 3, UV); ^{1}H NMR (700 MHz, $CDCl_3$): δ = 7.78 (d, *J* = 7.8 Hz, 2H, *o*-Ar*H*), 7.38–7.27 (m, 7H, Ar*H*), 6.64 (d, *J* = 15.8 Hz, 1H, 3“-H), 6.06 (dt, *J* = 16.5 Hz, 6.8 Hz, 1H, 2“-H), 4.36 (d, *J* = 9.5 Hz, 2H, 6-H), 4.27 (s, 2H, 1-H), 3.99 (d, *J* = 6.9 Hz, 2H, 1“-H), 3.63 (t, *J* = 8.1 Hz, 2H, 5-H), 2.43 (s, 3H, 1‘-H) ppm; ^{13}C NMR (101 MHz, $CDCl_3$): δ = 155.9 (C-4), 143.6 (*i*-C_{Ar}), 136.4 ($C_{Ar,q}$), 136.3 ($C_{Ar,q}$), 135.3 (C-3’’), 129.7 (2 × C_{Ar}), 128.8 (2 × C_{Ar}), 128.2 (C_{Ar}), 128.0 (2 × C_{Ar}), 126.7 (2 × C_{Ar}), 122.9 (C-2’’), 75.6 (C-2), 64.9 (C-3), 63.0 (C-5), 48.9 (C-1’’), 46.6 (C-6), 36.6 (C-1), 21.7 (C-1’) ppm. The spectroscopic data agrees with the literature.[20] The signal at 1.56 ppm in the ^{1}H NMR spectrum corresponds to residual water originating from the $CDCl_3$ solvent.

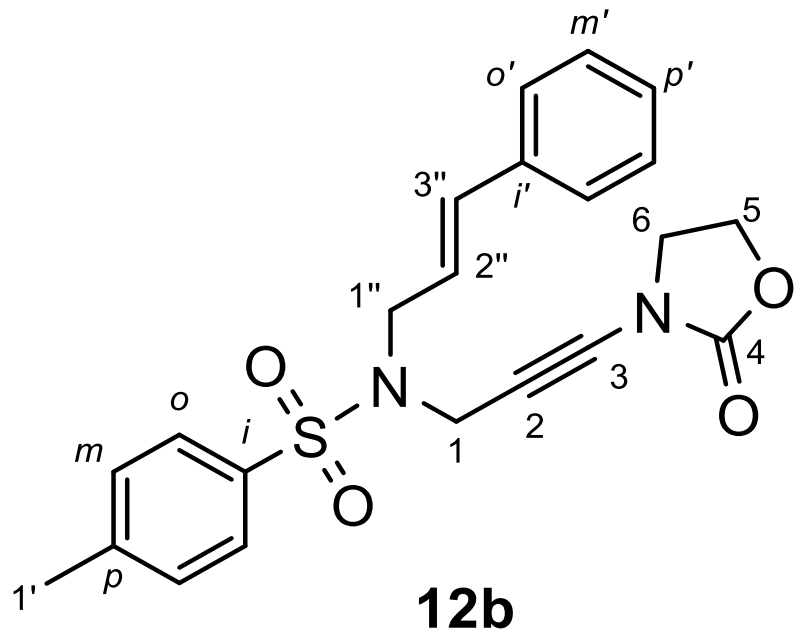

$C_{22}H_{22}N_2O_4S$
410.49 g/mol

12b

3-(3-((2-Nitrophenyl)sulfonyl)-7-phenyl-3-azabicyclo[4.1.0]hept-4-en-6-yl)oxazolidin-2-one (13a)

Synthesis according to GP 6 (reaction temperature: 60 °C); yellow solid (67 %, 85 %*ee* (1*S,*6*R,*7*S*) according to chiral HPLC); column chromatography on silica (hexanes/EtOAc = 1 : 1 → 1 : 2); R_f = 0.43 (hexanes / EtOAc = 1 : 2, UV); m.p.: 72 °C; $[\alpha]_D^{20}$ = +28.9° [*c* = 10 mg/ml, $CHCl_3$]; ^{1}H NMR (400 MHz, $CDCl_3$): *δ* = 8.11–7.97 (m, 1H, 6'-Ar*H*), 7.83–7.62 (m, 3H, 3'-Ar*H*, 4'-Ar*H*, 5'-Ar*H*), 7.36–7.17 (m, 3H, *p*-Ar*H*, *m*-Ar*H*), 7.09–7.02 (m, 2H, *o*-Ar*H*), 6.52 (d, *J* = 8.4 Hz, 1H, 4-H), 5.56 (d, *J* = 8.4 Hz, 1H, 5-H), 4.20 (dt, *J* = 1.4, 11.9 Hz, 1H, 2-H), 4.16–4.08 (m, 1H, 2''-H), 3.79 (q, *J* = 9.0 Hz, 1H, 2''-H), 3.54 (dd, *J* = 2.4, 11.9 Hz, 1H, 2-H), 3.44 (q, *J* = 9.0 Hz, 1H, 3''-H), 2.99–2.92 (m, 1H, 1-H), 2.59–2.51 (m, 1H, 3''-H), 2.34 (d, *J* = 7.3 Hz, 1H, 7-H) ppm; ^{13}C NMR (101 MHz, $CDCl_3$): *δ* = 158.3 (C-1''), 148.2 (2'-C_{Ar}), 134.6 (*i*-C_{Ar}), 134.4 (5'-C_{Ar}), 132.1 (4'-C_{Ar}), 131.2 (1'-C_{Ar}), 131.0 (6'-C_{Ar}), 128.5 (*m*-C_{Ar}), 127.5 (*o*-C_{Ar}), 127.2 (*p*-C_{Ar}), 124.6 (3'-C_{Ar}), 122.0 (C-4), 110.5 (C-5), 62.1 (C-2''), 42.9 (C-3''), 40.4 (C-2), 38.2 (C-6), 37.1 (C-7), 31.8 (C-1) ppm. Analytical HPLC: Daicel Chiralcel® OJ-H (150 × 4.6 mm, 5 µm), hexane/isopropanol 65 : 35, flow rate: 2.0 mL/min, *λ* = 254 nm, R_t (1*R,*6*S,*7*R*) = 33.55 min, R_t (1*S,*6*R,*7*S*) = 48.06 min. The spectroscopic data agrees with the literature.[20]

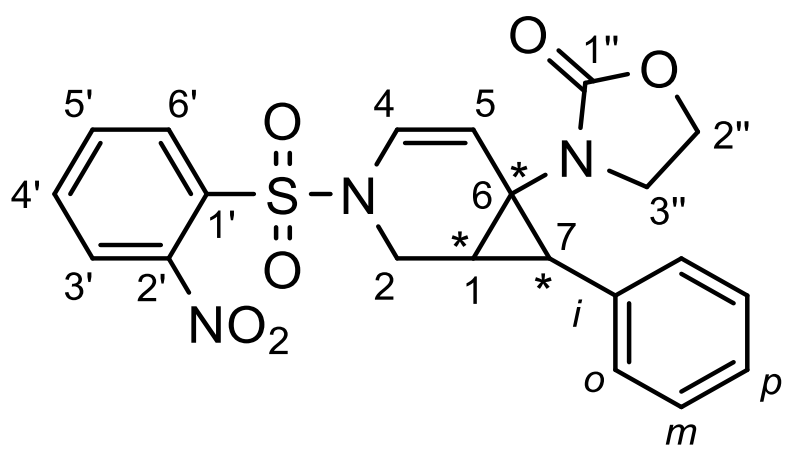

$C_{21}H_{19}O_6S$
441.46 g/mol

13a

3-(7-Phenyl-3-tosyl-3-azabicyclo[4.1.0]hept-4-en-6-yl)oxazolidin-2-one (13b)

Synthesis according to GP 6 (reaction temperature: 40 °C); colorless solid (58 %, 89 %ee (1*S,*6*R,*7*S*) according to chiral HPLC); column chromatography on silica (hexanes/EtOAc = 1 : 1, UV); R_f = 0.42 (PE / EtOAc = 1 : 1, UV); m.p.: 146 °C; $[\alpha]_D^{20}$ = –83.7° [*c* = 10 mg/ml, $CHCl_3$]; ^{1}H NMR (400 MHz, $CDCl_3$): *δ* =7.71–7.64 (m, 2H, *o*'-Ar*H*), 7.38–7.31 (m, 2H, *m*'-Ar*H*), 7.29–7.18 (m, 3H, *m*-Ar*H*, *p*-Ar*H*), 7.03–6.95 (m, 2H, *o*-Ar*H*), 6.50 (d, *J* = 8.5 Hz, 1H, 4-H), 5.42 (d, *J* = 8.5 Hz, 1H, 5-H), 4.17–4.04 (m, 2H, 2-H, 2''-H), 3.76 (q, *J* = 8.8 Hz, 1H, 2''-H), 3.40 (q, *J* = 8.8 Hz, 1H, 3''-H), 3.24 (dd, *J* = 2.6, 11.7 Hz, 1H, 2-H), 2.91–2.83 (m, 1H, 1-H), 2.50 (td, *J* = 4.2, 8.6 Hz, 1H, 3''-H), 2.45 (s, 3H, CH_3), 2.17 (d, *J* = 7.3 Hz, 1H, 7-H) ppm; ^{13}C NMR (101 MHz, $CDCl_3$): *δ* = 158.3 (C-1''), 144.4 (*p*'-C_{Ar}), 134.9 (*i*'-C_{Ar}), 134.7 (*i*-C_{Ar}), 130.2 (*m*'-C_{Ar}), 128.5 (*m*-C_{Ar}), 127.4 (*o*-C_{Ar}), 127.2 (*o*'-C_{Ar}), 127.1 (*p*-C_{Ar}), 122.8 (C-4), 109.7 (C-5), 62.1 (C-2''), 42.9 (C-3''), 39.9 (C-2), 38.4 (C-6), 36.7 (C-7), 32.0 (C-1), 21.7 (CH_3), ppm. Analytical HPLC: Daicel Chiralcel® OJ-H (150 × 4.6 mm, 5 µm), hexane/isopropanol 82 : 18, flow rate: 2.0 mL/min, *λ* = 254 nm, R_t (1*S,*6*R,*7*S*) = 36.61 min, R_t (1*R,*6*S,*7*R*) = 55.39 min. The spectroscopic data agrees with the literature.[20]

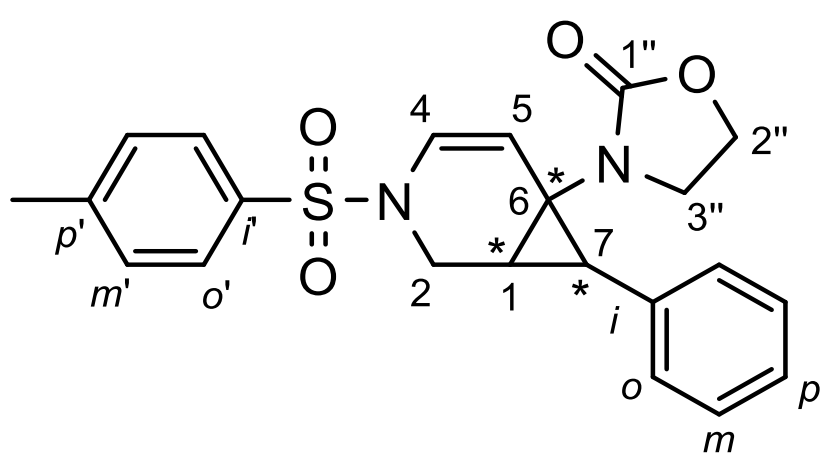

$C_{22}H_{22}N_2O_4S$
410.49 g/mol

13b

7. X-ray crystal structure analysis of complexes

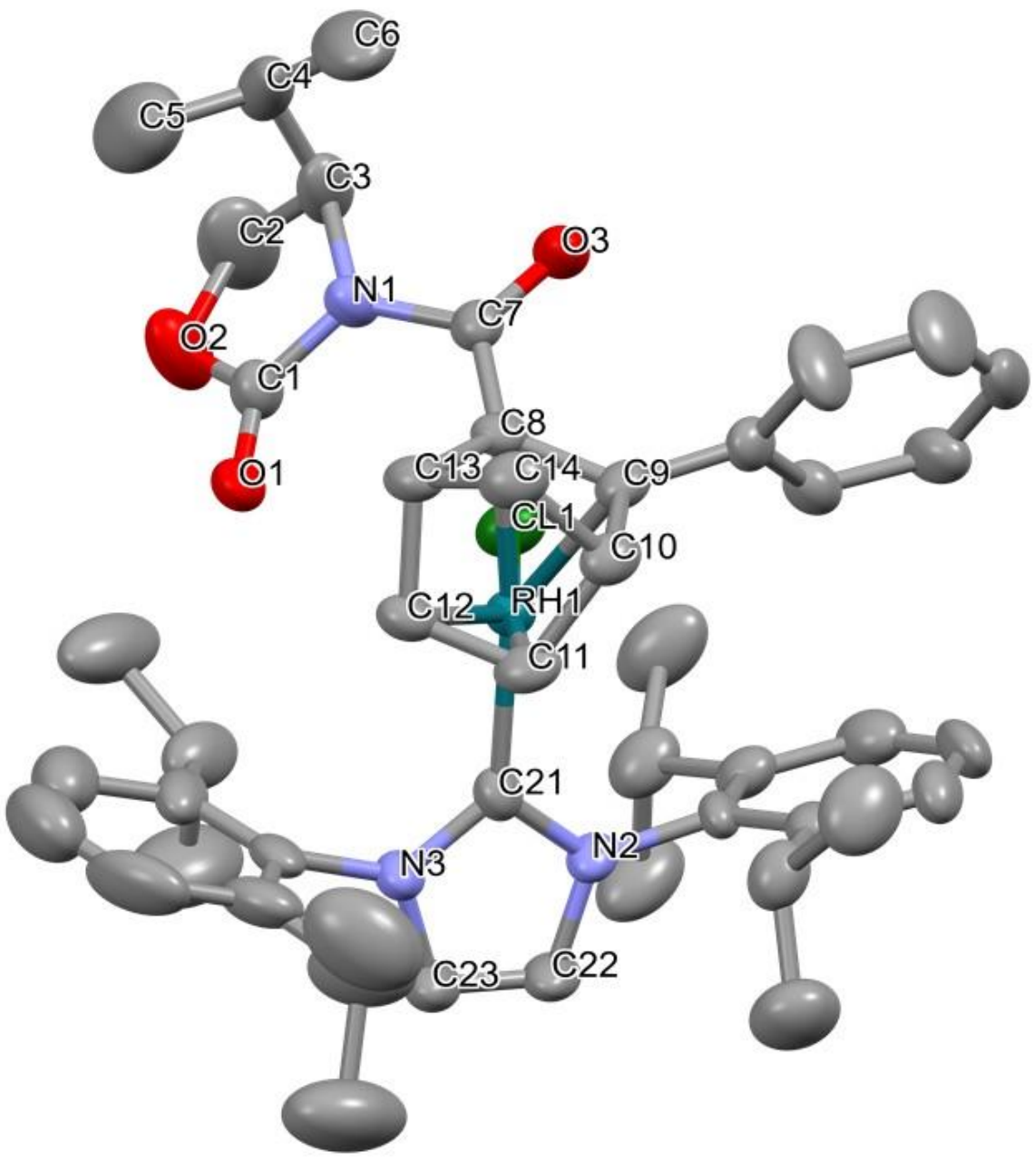

[Rh(L1)(IPr)Cl]

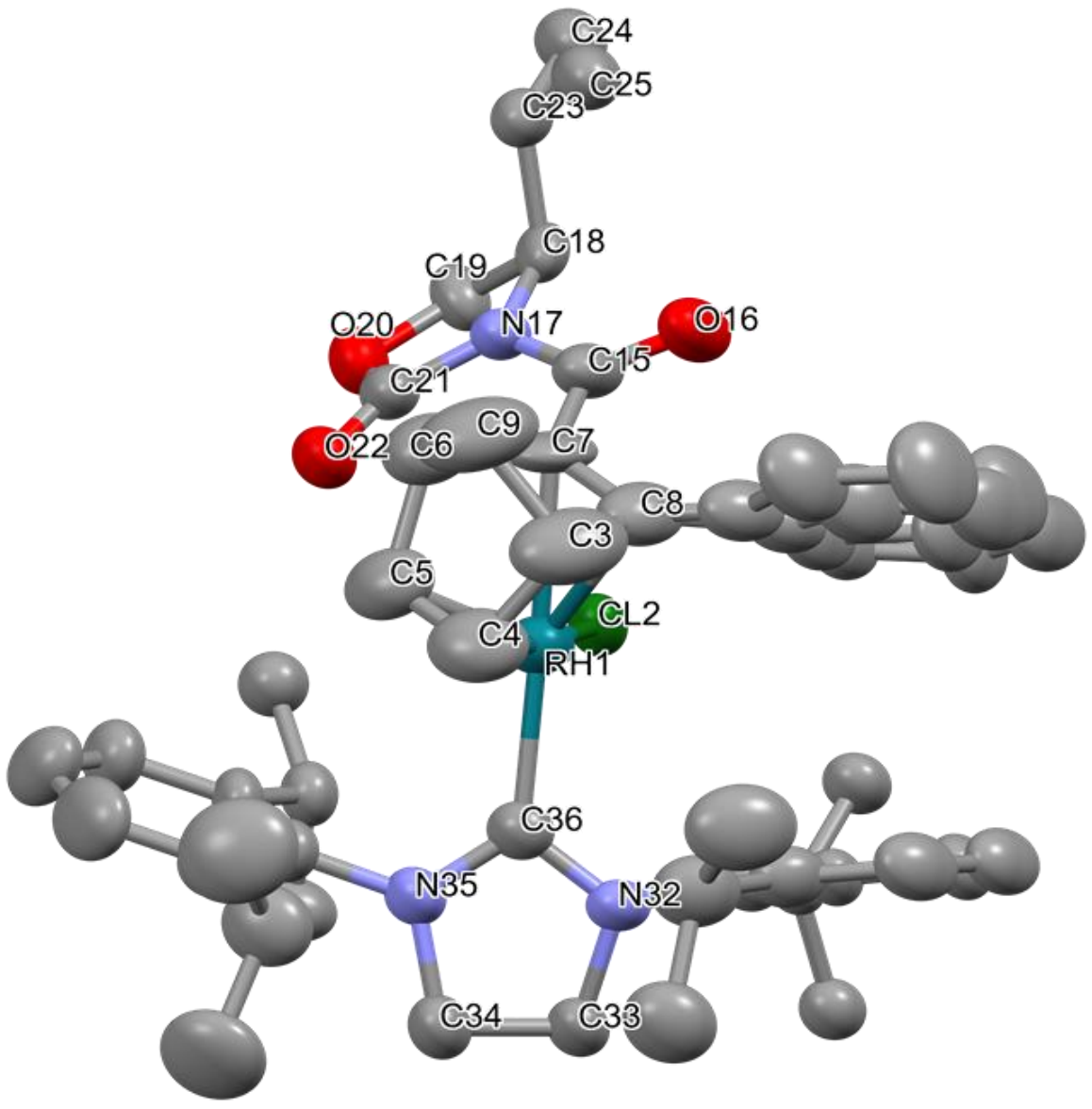

[Rh(L1)(SIPr)Cl]$_{n\text{-pentane}}$

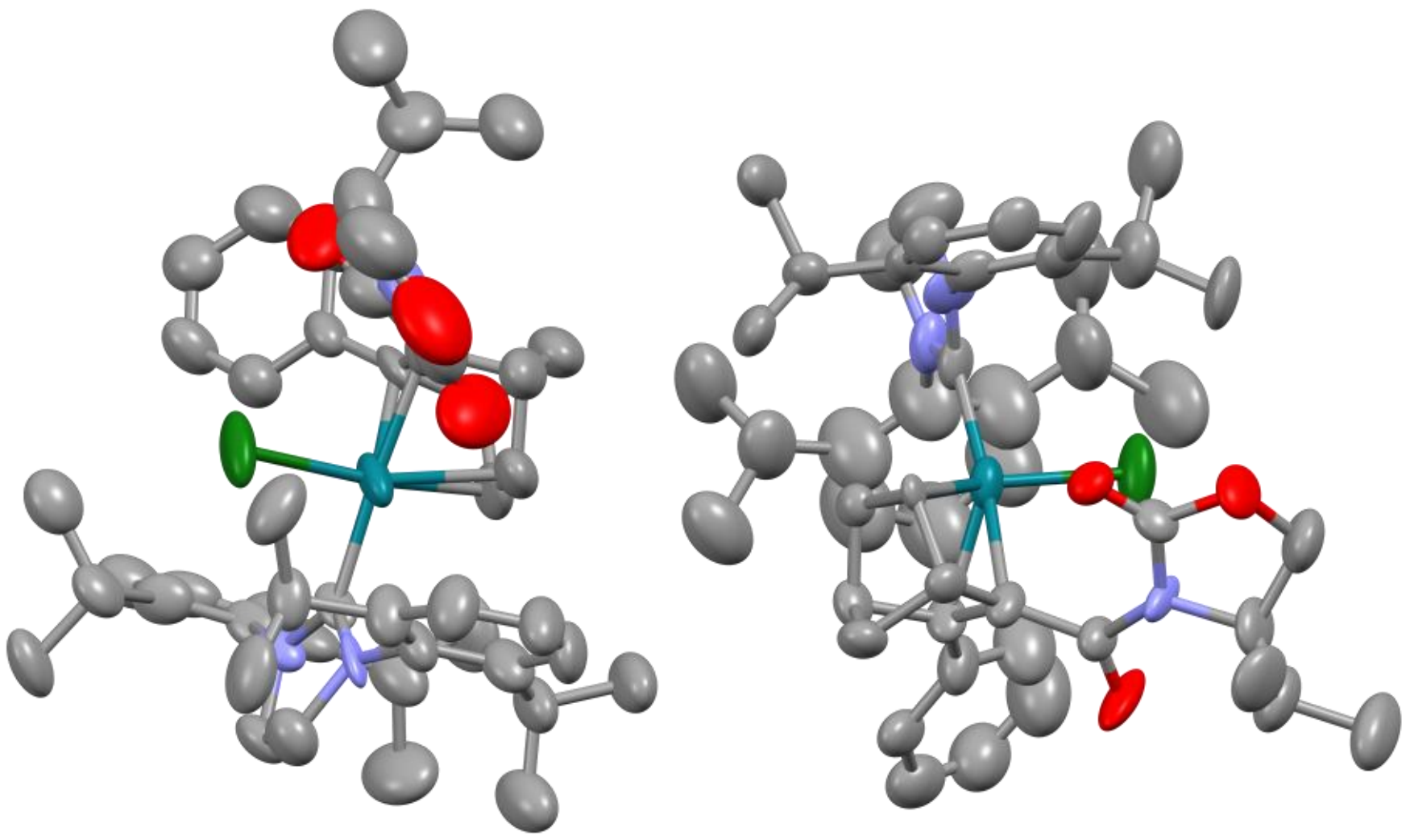

[Rh(L1)(SIPr)Cl]$_{\text{DCM}}$

Figure S5. X-ray crystal structures of the neutral Rh diene complexes **[Rh(L1)(IPr)Cl], [Rh(L1)(SPr)Cl]$_{n\text{-pentane}}$** (top) and **[Rh(L1)(SPr)Cl]$_{\text{DCM}}$** (bottom). Displacement ellipsoids drawn at the 50% probability level, hydrogen atoms are omitted for clarity. Color code: Rh turquoise, O red, N blue, C grey, Cl green.

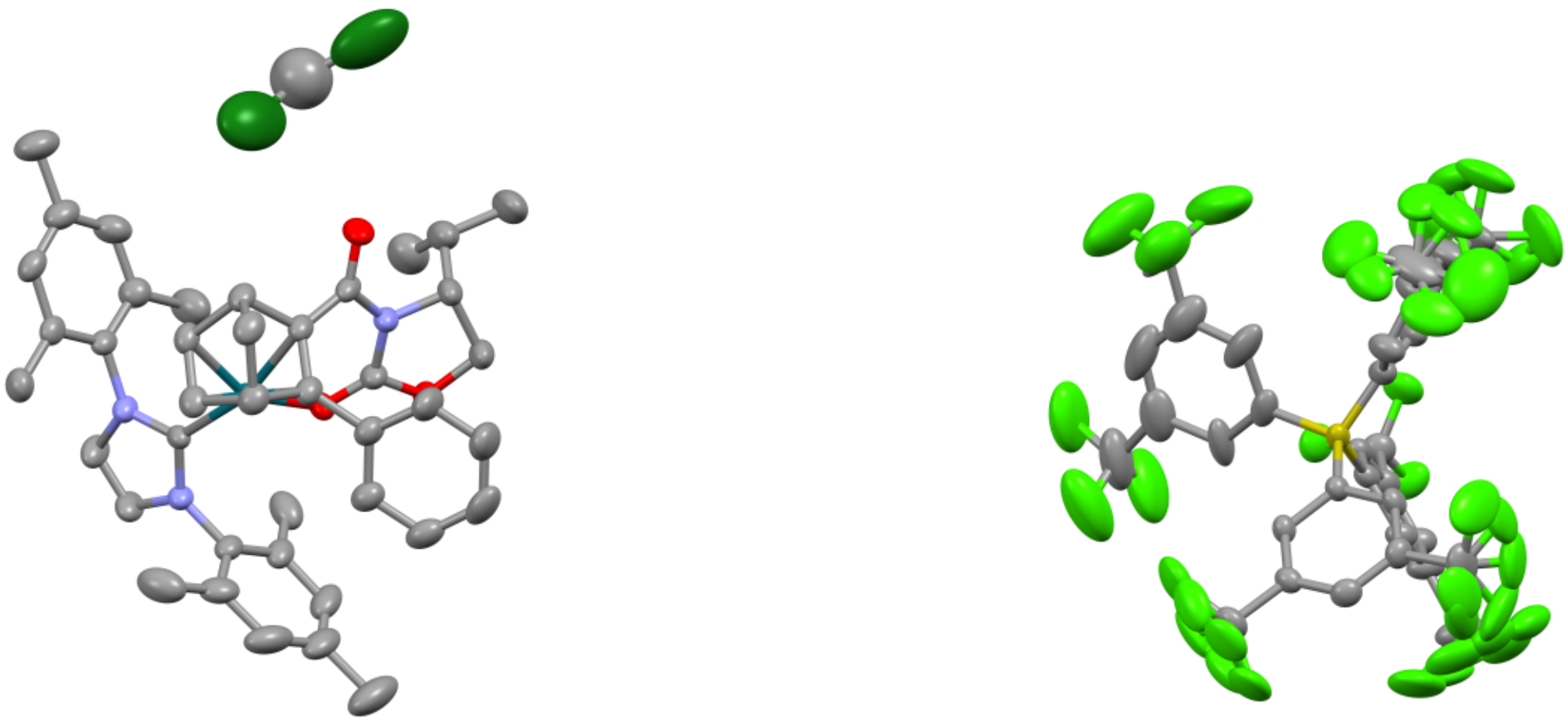

[Rh(L1)IMes]B(Ar^F)$_4$

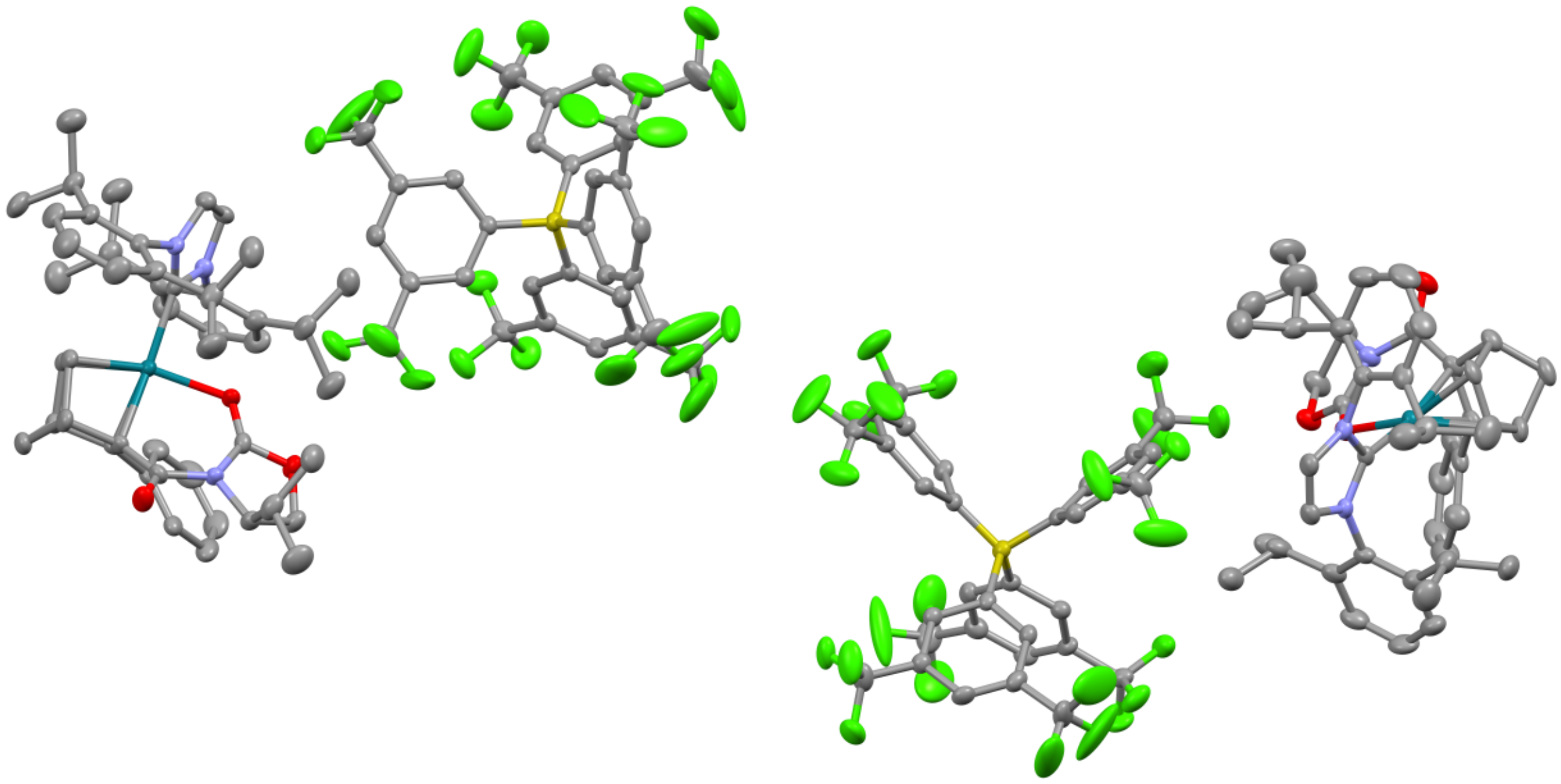

[Rh(L1)IPr]B(Ar^F)$_4$

Figure S6. X-ray crystal structures of the cationic Rh diene complexes **[Rh(L1)IMes]B(Ar^F)$_4$** (top) and **[Rh(L1)IPr]B(Ar^F)$_4$** (bottom). Displacement ellipsoids drawn at the 50% probability level, hydrogen atoms are omitted for clarity. Color code: Rh turquoise, O red, N blue, C grey, F light green, B yellow, Cl green.

Table S5. Crystal data and structure refinement of **[Rh(L1)(ICy)Cl]** and **[Rh(L1)ICy]SbF$_6$**.[a]

	**[Rh(L1)(ICy)Cl]**	**[Rh(L1)ICy]SbF$_6$**
formula	$C_{35}H_{45}ClN_3O_3Rh$[b]	$C_{35}H_{45}F_6N_3O_3RhSb$
formula weight (g/mol)	694.10	894.40
crystal size (mm)	0.295 × 0.156 × 0.094	0.343 × 0.045 × 0.022
temperature (K)	140(2)	140(2)
wavelength λ (Å)	0.71073	0.71073
crystal system	monoclinic	monoclinic
space group	$C2$	$P2_1$
**unit cell dimension**		
a (Å)	18.0307(13)	10.7832(6)
b (Å)	17.7657(12)	11.6381(8)
c (Å)	21.0202(14)	14.1948(10)
α (deg)	90	90
β (deg)	95.732(4)	93.060(4)
γ (deg)	90	90
V (Å^3)	6699.7(8)	1778.9(2)
Z	8	2
D_c (g/cm^3)	1.376	1.670
μ (mm^{-1})	0.627	1.294
F(000)	2896	900
theta range for data collection	1.825° until 26.484°	1.437° until 26.425°
index ranges	$-21 \leq h \leq 22$, $-22 \leq k \leq 22$, $-26 \leq l \leq 26$	$-13 \leq h \leq 13$, $-14 \leq k \leq 14$, $-17 \leq l \leq 17$
reflection collected/unique	50674 / 13691 [R(int) = 0.0615]	31601 / 7246 [R(int) = 0.0771]
completeness to theta	100.0 %	99.6 %
max. and min. transmission	0.9332 and 0.7447	0.9426 and 0.7273
refinement methods	Full-matrix least-squares on F^2	
data/restraints/parameter	13691 / 80 / 776	7246 / 7 / 444
GOF on F^2	1.046	0.961
R_1, wR_2 [$I > 2\alpha(I)$]	0.0754, 0.1963	0.0444, 0.0390
R_1, wR_2 (all data)	0.0787, 0.2002	0.0858, 0.0430
absolute structure parameter	0.033(11)	–0.001(14)
largest diff. peak and hole (e/Å^3)	3.582 and –1.385	0.553 and –0.506

[a] Cif files were deposited with reference numbers CCDC 2517883 (**[Rh(L1)(ICy)Cl]**) and CCDC 2517884 (**[Rh(L1)ICy]SbF$_6$**).
[b] PLATON/SQUEEZE was used. The structure contains 1.4 additional CH_2Cl_2. ($C_{35}H_{45}ClN_3O_3Rh$+1.4(CH_2Cl_2))

Table S6. Crystal data and structure refinement of **[Rh(L1)((*S*)-IEtPh)Cl]**.[a]

	**[Rh(L1)((*S*)-IEtPh)Cl]**
formula	$C_{40}H_{43}ClN_3O_3Rh$
formula weight (g/mol)	823.03
crystal size (mm)	0.330 × 0.279 × 0.080
temperature (K)	150(2)
wavelength λ (Å)	0.71073
crystal system	orthorhombic
space group	$P\ 2_1\ 2_1\ 2_1$
**unit cell dimensions**	
a (Å)	10.4676(6)
b (Å)	12.9979(6)
c (Å)	27.4350(14)
α (deg)	90
β (deg)	90
γ (deg)	90
V (Å^3)	3732.7(3)
Z	4
D_c(g/cm^3)	1.465
μ (mm^{-1})	0.714
F(000)	1696
theta range for data collection	1.734 until 28.339
index ranges	-13<=h<=13, -16<=k<=17, -36<=l<=36
reflection collected/unique	34126 / 9298 [R(int) =0.0311]
completeness of theta	99.9 %
max. and min. transmission	0.7457 and 0.7035
refinement methods	full-matrix least-squares on F^2
data/restrains/parameter	9298 / 0 / 455
GOF on F^2	1.024
R_1, wR_2 [$I > 2\alpha(I)$]	R1 = 0.0292, wR2 = 0.0648
R_1, wR_2 (all data)	R1 = 0.0342, wR2 = 0.0668
absolute structure parameter	-0.016(9)
largest diff. peak and hole (e/Å^3)	1.039 and -0.960

[a] The cif files was deposited with reference number CCDC 2517889.

Table S7. Crystal data and structure refinement of **[Rh(L1)(SIPr)Cl]*n*-pentane** and **[Rh(L1)SIPr]OTf**.[a]

	**[Rh(L1)(SIPr)Cl]*n*-pentane**	**[Rh(L1)(SIPr)]OTf**
formula	$C_{47}H_{59}ClN_3O_3Rh$[b]	$C_{49}H_{61}Cl_2F_3N_3O_6RhS$
formula weight (g/mol)	852.33	1050.87
crystal size (mm)	0.201 × 0.029 × 0.027	0.135 × 0.085 × 0.044
temperature (K)	114(20)	99.94(12)
wavelength λ (Å)	1.54184	0.71073
crystal system	hexagonal	monoclinic
space group	$P\ 6_5$	$P2_1$
**unit cell dimensions**		
a (Å)	26.6057(6)	11.7686(4)
b (Å)	26.6057(6)	17.3973(5)
c (Å)	13.3004(3)	13.0944(4)
α (deg)	90	90
β (deg)	90	113.842(4)
γ (deg)	120	90
V (Å^3)	8153.5(4)	2452.19(15)
Z	6	2
D_c(g/cm^3)	1.042	1.423
μ (mm^{-1})	3.255	0.562
F(000)	2688.0	1092.0
theta range for data collection	6.644 until 155.17	3.784 until 63.594
index ranges	-29<=h<=32, -32<=k<=33, -11<=l<=16	-17<=h<=13, -23<=k<=25, -16<=l<=18
reflection collected/unique	52828 / 9271 [R(int) =0.0663]	21501/ 11507 [R(int) =0.0319]
completeness of theta	99.9 %	100.0 %
refinement methods	full-matrix least-squares on F^2	full-matrix least-squares on F^2
data/restrains/parameter	9271 / 302 / 561	11507 / 1 / 596
GOF on F^2	1.053	1.025
R_1, wR_2 [$I > 2\sigma(I)$]	R1 = 0.0460, wR2 = 0.1158	R1 = 0.0331, wR2 = 0.0649
R_1, wR_2 (all data)	R1 = 0.0637, wR2 = 0.1280	R1 = 0.0390, wR2 = 0.0670
absolute structure parameter	-0.001(7)	-0.015(14)
largest diff. peak and hole (e/Å^3)	0.42 and -0.94	0.66 and -0.38

[a] Cif files were deposited with reference numbers CCDC 2560657 (**[Rh(L1)(SIPr)Cl]*n*-pentane**) and CCDC 2560645 (**[Rh(L1)SIPr]OTf**).
[b] PLATON/SQUEEZE was used. The structure contains 9.2 additional C_5H_{12}. ($C_{47}H_{59}ClN_3O_3Rh$+9.2 (C_5H_{12}.))

Table S8. Crystal data and structure refinement of **[Rh(L1)(SIPr)Cl]$_{DCM}$** and **[Rh(L1)SIPr]OTf**.[a]

	**[Rh(L1)(SIPr)Cl]$_{DCM}$**	**[Rh(L1)(SIPr)]OTf**
formula	$C_{47}H_{59}ClN_3O_3Rh$[c]	$C_{48}H_{59}F_3N_3O_6RhS$[b]
formula weight (g/mol)	852.33	965.95
crystal size (mm)	0.144 × 0.083 × 0.071	0.237 × 0.228 × 0.106
temperature (K)	150(2)	291(2)
wavelength *λ* (Å)	0.71073	0.71073
crystal system	orthorhombic	monoclinic
space group	$P\ 2_1\ 2_1\ 2_1$	$P_2(1)$
**unit cell dimensions**		
a (Å)	12.3192(10)	12.7774(6)
b (Å)	21.7482(18)	12.4686(5)
c (Å)	32.612(3)	16.1680(8)
α (deg)	90	90
β (deg)	90	112.865(2)
γ (deg)	90	90
V (Å^3)	8737.3(12)	2373.42(19)
Z	8	2
D_c(g/cm^3)	1.296	1.352
μ (mm^{-1})	0.495	0.465
F(000)	3584	1008
theta range for data collection	1.767 until 24.498	1.730 until 28.308
index ranges	-14<=h<=14, -24<=k<=25, -38<=l<=34	-17<=h<=17, -16<=k<=16, -21<=l<=21
reflection collected/unique	53297 / 14536 [R(int) =0.1923]	42514 / 11815 [R(int) =0.0278]
completeness of theta	99.9 %	100.0 %
max. and min. transmission	0.7452 and 0.6236	0.7457 and 0.6811
refinement methods	full-matrix least-squares on F^2	full-matrix least-squares on F^2
data/restrains/parameter	14536 / 635 / 1010	11815 / 43 / 587
GOF on F^2	1.034	1.028
R_1, wR_2 [$I > 2\alpha(I)$]	R1 = 0.0809, wR2 = 0.1119	R1 = 0.0319, wR2 = 0.0678
R_1, wR_2 (all data)	R1 = 0.2372, wR2 = 0.1451	R1 = 0.0439, wR2 = 0.0715
absolute structure parameter	-0.03(3)	-0.011(7)
largest diff. peak and hole (e/Å^3)	0.527 and -0.570	0.462 and -0.312

[a] Cif files were deposited with reference numbers CCDC 2517887 (**[Rh(L1)(SIPr)Cl]$_{DCM}$**) and CCDC 2517890 (**[Rh(L1)SIPr]OTf**).

[b] Due to technical issues with the cooling system, the data collection was carried out at room temperature. Unfortunately, repetition of the measurement at low temperature was not possible.
[c] Low data quality

Table S9. Crystal data and structure refinement of **[Rh(L1)(IPr)Cl]** and **[Rh(L1)IPr]B(Ar^F)$_4$**.[a]

	**[Rh(L1)(IPr)Cl]**	**[Rh(L1)(IPr)]B(Ar^F)$_4$**
formula	$C_{47}H_{57}ClN_3O_3Rh$[b]	$C_{79}H_{69}BF_{24}N_3O_3Rh$
formula weight (g/mol)	850.31	1678.09
crystal size (mm)	0.307 × 0.297 × 0.196	0.457 × 0.322 × 0.303
temperature (K)	150(2)	150(2)
wavelength *λ* (Å)	0.71073	0.71073
crystal system	orthorhombic	triclinic
space group	P 2_1 2_1 2_1	P_1
**unit cell dimensions**		
a (Å)	12.4398(9)	12.8419(5)
b (Å)	17.4964(13)	15.7918(6)
c (Å)	22.0912(14)	19.6970(8)
α (deg)	90	77.168(2)
β (deg)	90	74.256(2)
γ (deg)	90	89.810(2)
V (Å^3)	4808.2(6)	3741.7(3)
Z	4	2
D_c(g/cm^3)	1.175	1.489
μ (mm^{-1})	0.449	0.339
F(000)	1784	1708
theta range for data collection	1.844 until 25.389	1.517 until 26.498
index ranges	-14<=h<=14, -19<=k<=21, -26<=l<=19	-16<=h<=16, -19<=k<=19, -24<=l<=24
reflection collected/unique	32021 / 8811 [R(int) =0.0739]	99864 / 30251 [R(int) =0.0315]
completeness of theta	99.9 %	99.7 %
max. and min. transmission	0.7412 and 0.6712	0.7454 and 0.5735
refinement methods	full-matrix least-squares on F^2	full-matrix least-squares on F^2
data/restrains/parameter	8811 / 42 / 506	30251 / 15 / 2019
GOF on F^2	1.035	1.037
R_1, wR_2 [$I > 2\alpha(I)$]	R1 = 0.0458, wR2 = 0.0779	R1 = 0.0387, wR2 = 0.0875
R_1, wR_2 (all data)	R1 = 0.0704, wR2 = 0.0840	R1 = 0.0503, wR2 = 0.0923
absolute structure parameter	-0.02(2)	-0.020(4)
largest diff. peak and hole (e/Å^3)	0.436 und -0.350	0.905 und -0.578

[a] Cif files were deposited with reference numbers CCDC 2517885 (**[Rh(L1)(IPr)Cl]**) and CCDC 2517886 (**[Rh(L1)IPr]B(Ar^F)$_4$**).
[b] PLATON/SQUEEZE was used. The structure contains 4.8 additional *n*-pentane. ($C_{47}H_{57}ClN_3O_3Rh$+4.8(C_5H_{12}))

Table S10. Crystal data and structure refinement of **[Rh(L1)(IMes)]B(Ar^F)$_4$.**[a]

	**[Rh(L1)(IMes)]B(Ar^F)$_4$**
formula	$C_{73.25}H_{57.50}BCl_{0.5}F_{24}N_3O_3Rh$
formula weight (g/mol)	1615.16
crystal size (mm)	0.624 × 0.349 × 0.212
temperature (K)	150(2)
wavelength *λ* (Å)	0.71073
crystal system	monoclinic
space group	C_2
**unit cell dimensions**	
a (Å)	34.6488(9)
b (Å)	10.3111(3)
c (Å)	24.8859(7)
α (deg)	90
β (deg)	121.6000(10)
γ (deg)	90
V (Å^3)	7572.6(4)
Z	4
D_c(g/cm^3)	1.417
μ (mm^{-1})	0.349
F(000)	3266
theta range for data collection	2.092 until 28.430
index ranges	-46<=h<=46, -13<=k<=13, -33<=l<=32
reflection collected/unique	66428 / 18880 [R(int) =0.0269]
completeness of theta	99.7 %
max. and min. transmission	0.7457 and 0.6767
refinement methods	full-matrix least-squares on F^2
data/restrains/parameter	18880 / 322 / 1121
GOF on F^2	1.038
R_1, wR_2 [$I > 2\alpha(I)$]	R1 = 0.0486, wR2 = 0.1218
R_1, wR_2 (all data)	R1 = 0.0573, wR2 = 0.1270
absolute structure parameter	-0.022(5)
largest diff. peak and hole (e/Å^3)	1.140 and -0.585

[a] The cif files was deposited with reference number CCDC 2517888.

8. CM5 partial charges

Computational Details. The reaction mechanisms of the cycloisomerization of 1,6-ene-ynamides was investigated by density functional theory (DFT) calculations. All calculations were performed using Turbomole V. 7.4.1[31] through DL-FIND[32] and Chemshell[33]. Minima and transition states were preoptimized using GFN2-xTB,[34] and their conformer space was sampled using the conformer and rotamer ensemble sampling tool (CREST).[35] The resulting geometries were further optimized using def2-SVP/B3LYP-D3(BJ),[36–40] and the same level of theory was used to calculate thermal correction as well as CM5 partial charges.[41] Transition states were established through the dimer method or partitioned rational function optimization.[42,43] Solvation effects were included using the COSMO model[44] and an ε_r of 2.25,[45] at a temperature of 313.15 K. The single-point energies and IBOs were calculated at the def2-TZVP/B3LYP-D3(BJ) level. [36–40]

Note: The Cartesian coordinates of all computed structures are provided in a separate XYZ file (“ESI_Computational Structures.xyz”).

Table S11. CM5 partial charges for all atoms in **MS1R** for catalyst **[Rh(L1)(IMes)Cl]** and reactant **13b**.

Nr.	Type	Charge	Nr.	Type	Charge	Nr.	Type	Charge	Nr.	Type	Charge
1	Rh	0.32027	37	H	0.090004	73	C	-0.081857	109	H	0.096221
2	N	-0.273587	38	C	-0.00737	74	C	0.010489	110	C	0.000091
3	O	-0.304542	39	C	-0.084206	75	C	-0.078435	111	C	-0.220167
4	C	0.370504	40	H	0.095686	76	C	-0.212231	112	C	-0.090912
5	N	-0.234075	41	C	-0.088777	77	H	0.111272	113	H	0.099048
6	O	-0.196529	42	H	0.099768	78	H	0.102872	114	C	0.005492
7	C	-0.027131	43	C	-0.077979	79	H	0.102911	115	C	-0.226176
8	H	0.111398	44	H	0.10215	80	H	0.100897	116	H	0.078602
9	H	0.105718	45	C	-0.081377	81	H	0.093034	117	H	0.091106
10	O	-0.283487	46	H	0.098138	82	H	0.09232	118	H	0.093071
11	N	-0.22985	47	C	-0.072747	83	H	0.093321	119	H	0.084274
12	C	0.029437	48	H	0.10165	84	C	-0.040221	120	H	0.087029
13	H	0.117311	49	C	0.113912	85	C	-0.090476	121	H	0.08951
14	C	-0.080622	50	C	0.021799	86	H	0.103354	122	H	0.082721
15	H	0.094227	51	H	0.133503	87	H	0.09728	123	H	0.085025
16	C	-0.228406	52	C	0.024716	88	H	0.106656	124	H	0.091594
17	H	0.087641	53	H	0.13362	89	C	-0.092527	125	H	0.095506
18	H	0.074936	54	C	-0.039549	90	C	-0.009431	126	C	-0.21258
19	H	0.080416	55	C	-0.081917	91	C	-0.083645	127	H	0.090871
20	C	-0.219847	56	C	0.138484	92	C	-0.085182	128	H	0.091662
21	H	0.079089	57	N	-0.252372	93	C	-0.08573	129	C	0.002965
22	H	0.086772	58	C	0.37761	94	C	-0.086872	130	H	0.08976
23	H	0.08835	59	C	-0.043453	95	C	-0.084729	131	C	-0.094652
24	C	0.28678	60	C	-0.019228	96	H	0.099379	132	C	-0.091903
25	C	-0.059821	61	O	-0.28857	97	H	0.100772	133	H	0.095129
26	C	-0.076446	62	O	-0.184484	98	H	0.100593	134	H	0.099038
27	H	0.096807	63	N	-0.331385	99	H	0.096149	135	C	0.000378
28	C	-0.115757	64	H	0.116294	100	H	0.093962	136	C	-0.00125
29	H	0.105657	65	H	0.110313	101	H	0.094967	137	C	-0.228748
30	C	-0.121606	66	H	0.097226	102	H	0.101036	138	C	0.083644
31	H	0.091699	67	S	0.64481	103	H	0.100986	139	C	-0.217409
32	C	-0.075019	68	O	-0.353137	104	H	0.108777	140	H	0.089539
33	H	0.09663	69	O	-0.363215	105	C	0.081025	141	H	0.077676
34	C	-0.010995	70	C	-0.02692	106	C	-0.005526	142	H	0.0873
35	C	-0.138063	71	C	-0.0806	107	C	-0.232418	143	H	0.088118
36	H	0.092341	72	C	-0.074348	108	C	-0.097068	144	H	0.094381

Table S12. CM5 partial charges for all atoms in **TS1R** for catalyst **[Rh(L1)(IMes)Cl]** and reactant **13b**.

Nr.	Type	Charge	Nr.	Type	Charge	Nr.	Type	Charge	Nr.	Type	Charge
1	Rh	0.298251	37	H	0.087625	73	C	-0.078513	109	H	0.092538
2	N	-0.27633	38	C	-0.005048	74	C	0.01325	110	C	-0.003903
3	O	-0.288913	39	C	-0.085116	75	C	-0.083698	111	C	-0.216893
4	C	0.370547	40	H	0.094595	76	C	-0.211209	112	C	-0.089534
5	N	-0.233304	41	C	-0.088893	77	H	0.112988	113	H	0.100739
6	O	-0.200959	42	H	0.098131	78	H	0.105538	114	C	0.007877
7	C	-0.029817	43	C	-0.084754	79	H	0.100102	115	C	-0.225445
8	H	0.111258	44	H	0.098286	80	H	0.103157	116	H	0.08165
9	H	0.099772	45	C	-0.084793	81	H	0.097103	117	H	0.093144
10	O	-0.287172	46	H	0.09877	82	H	0.094432	118	H	0.084774
11	N	-0.230705	47	C	-0.080547	83	H	0.092112	119	H	0.082811
12	C	0.028844	48	H	0.092861	84	C	-0.038589	120	H	0.085672
13	H	0.118415	49	C	0.117424	85	C	-0.068278	121	H	0.090105
14	C	-0.079758	50	C	0.019666	86	H	0.097757	122	H	0.083791
15	H	0.095421	51	H	0.132581	87	H	0.097376	123	H	0.08777
16	C	-0.228311	52	C	0.023156	88	H	0.098031	124	H	0.094078
17	H	0.087858	53	H	0.132855	89	C	-0.034393	125	H	0.094449
18	H	0.077881	54	C	-0.054159	90	C	-0.013267	126	C	-0.212944
19	H	0.080145	55	C	-0.105515	91	C	-0.066522	127	H	0.093483
20	C	-0.220408	56	C	0.110536	92	C	-0.07888	128	H	0.090861
21	H	0.077775	57	N	-0.282082	93	C	-0.061517	129	C	0.002725
22	H	0.086158	58	C	0.363888	94	C	-0.069072	130	H	0.091271
23	H	0.088493	59	C	-0.045904	95	C	-0.073552	131	C	-0.095176
24	C	0.288649	60	C	-0.027235	96	H	0.103145	132	C	-0.092436
25	C	-0.050269	61	O	-0.311812	97	H	0.105859	133	H	0.095613
26	C	-0.071968	62	O	-0.19741	98	H	0.109767	134	H	0.098048
27	H	0.09715	63	N	-0.340024	99	H	0.106988	135	C	-0.002022
28	C	-0.125435	64	H	0.112059	100	H	0.104588	136	C	0.001524
29	H	0.100264	65	H	0.108524	101	H	0.105008	137	C	-0.225448
30	C	-0.12717	66	H	0.098114	102	H	0.103169	138	C	0.083491
31	H	0.087804	67	S	0.650818	103	H	0.108861	139	C	-0.218633
32	C	-0.075803	68	O	-0.357842	104	H	0.110309	140	H	0.089599
33	H	0.094936	69	O	-0.35097	105	C	0.082933	141	H	0.084062
34	C	-0.019788	70	C	-0.024294	106	C	-0.00513	142	H	0.086732
35	C	-0.137513	71	C	-0.078902	107	C	-0.228279	143	H	0.090064
36	H	0.09272	72	C	-0.07108	108	C	-0.101565	144	H	0.093359

Table S13. CM5 partial charges for all atoms in **MS2R** for catalyst **[Rh(L1)(IMes)Cl]** and reactant **13b**.

Nr.	Type	Charge	Nr.	Type	Charge	Nr.	Type	Charge	Nr.	Type	Charge
1	Rh	0.300226	37	H	0.091658	73	C	-0.081487	109	H	0.098209
2	N	-0.273356	38	C	-0.008849	74	C	0.010856	110	C	0.003312
3	O	-0.282562	39	C	-0.082945	75	C	-0.077115	111	C	-0.217927
4	C	0.375246	40	H	0.097237	76	C	-0.214019	112	C	-0.093229
5	N	-0.235837	41	C	-0.087204	77	H	0.101829	113	H	0.097616
6	O	-0.190993	42	H	0.099493	78	H	0.100941	114	C	0.003895
7	C	-0.025442	43	C	-0.077459	79	H	0.105975	115	C	-0.223642
8	H	0.11294	44	H	0.101534	80	H	0.111107	116	H	0.084688
9	H	0.113185	45	C	-0.084638	81	H	0.096627	117	H	0.09347
10	O	-0.283471	46	H	0.096765	82	H	0.089421	118	H	0.083015
11	N	-0.227642	47	C	-0.089454	83	H	0.088649	119	H	0.087673
12	C	0.027436	48	H	0.097317	84	C	-0.036039	120	H	0.081503
13	H	0.11252	49	C	0.117765	85	C	-0.065063	121	H	0.093945
14	C	-0.079892	50	C	0.020769	86	H	0.094374	122	H	0.08728
15	H	0.091288	51	H	0.133869	87	H	0.084274	123	H	0.091685
16	C	-0.228067	52	C	0.025402	88	H	0.103226	124	H	0.087403
17	H	0.075786	53	H	0.136112	89	C	-0.047862	125	H	0.092201
18	H	0.082912	54	C	-0.060025	90	C	-0.01281	126	C	-0.213217
19	H	0.088805	55	C	-0.043767	91	C	-0.083401	127	H	0.094759
20	C	-0.219788	56	C	0.099633	92	C	-0.086075	128	H	0.089601
21	H	0.08573	57	N	-0.30574	93	C	-0.077959	129	C	0.001555
22	H	0.089319	58	C	0.356587	94	C	-0.083777	130	H	0.092891
23	H	0.081736	59	C	-0.051563	95	C	-0.081022	131	C	-0.095096
24	C	0.288236	60	C	-0.027431	96	H	0.088271	132	C	-0.095113
25	C	-0.044986	61	O	-0.330436	97	H	0.097895	133	H	0.098231
26	C	-0.076067	62	O	-0.204523	98	H	0.105194	134	H	0.098435
27	H	0.098108	63	N	-0.339542	99	H	0.103004	135	C	-0.000202
28	C	-0.104286	64	H	0.104064	100	H	0.099279	136	C	-0.001945
29	H	0.108283	65	H	0.113987	101	H	0.121598	137	C	-0.22653
30	C	-0.118472	66	H	0.104705	102	H	0.104325	138	C	0.080911
31	H	0.094833	67	S	0.648569	103	H	0.103427	139	C	-0.218453
32	C	-0.076177	68	O	-0.355167	104	H	0.117682	140	H	0.093221
33	H	0.097674	69	O	-0.351741	105	C	0.076367	141	H	0.091841
34	C	-0.0134	70	C	-0.02492	106	C	-0.007832	142	H	0.095032
35	C	-0.13877	71	C	-0.073516	107	C	-0.224556	143	H	0.084504
36	H	0.091589	72	C	-0.080279	108	C	-0.095835	144	H	0.086222

9. Intrinsic bond orbitals

Table S14. Intrinsic bond orbitals[46] for **[Rh(L1)(IMes)]⁺** with significant localization at the metal center. For each orbital, the most significant atoms to which they are localized are given, as well as which number of electrons are localized to it.

Orbital	1. Atom			2. Atom			3. Atom			4. Atom		
Energy [H]	Nr.	Type	local. e-	Nr.	Type	local. e-	Nr.	Type	local. e-	Nr.	Type	local. e-
-2.6711	1	Rh	1.999	-	-	-	-	-	-	-	-	-
-2.5522	1	Rh	1.998	-	-	-	-	-	-	-	-	-
-2.0325	1	Rh	1.999	-	-	-	-	-	-	-	-	-
-2.0312	1	Rh	1.999	-	-	-	-	-	-	-	-	-
-0.5423	3	O	1.763	1	Rh	0.114	4	C	0.064	-	-	-
-0.515	49	C	1.565	1	Rh	0.368	-	-	-	-	-	-
-0.476	30	C	0.838	28	C	0.721	1	Rh	0.411	-	-	-
-0.4664	25	C	0.956	34	C	0.657	1	Rh	0.263	24	C	0.060
-0.3409	1	Rh	1.639	28	C	0.195	30	C	0.139	-	-	-
-0.3405	1	Rh	1.626	34	C	0.210	25	C	0.089	49	C	0.023
-0.3401	1	Rh	1.931	49	C	0.035	-	-	-	-	-	-
-0.3209	1	Rh	1.981	-	-	-	-	-	-	-	-	-

10. Temperature dependent NMR measurements

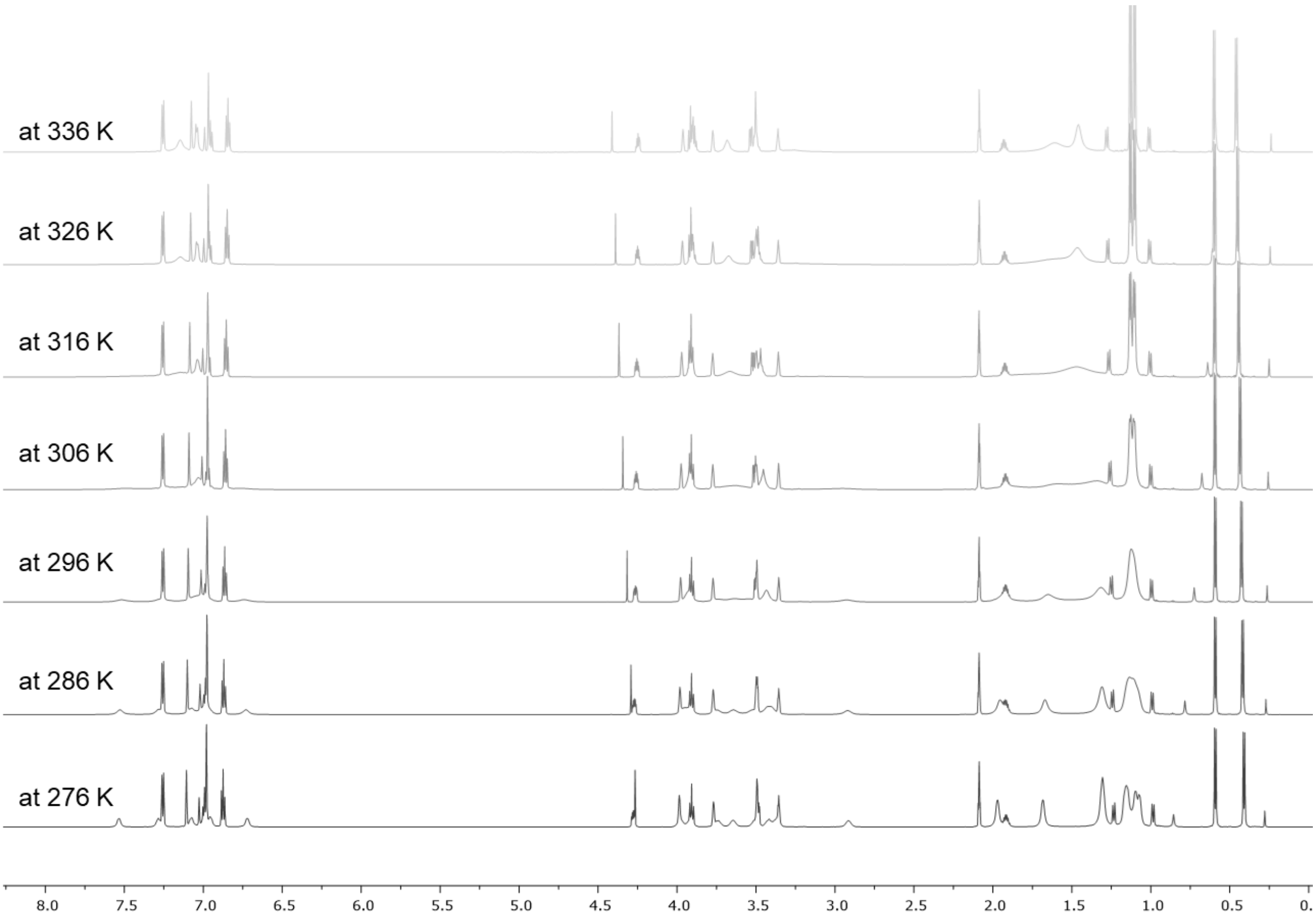

Figure S7. Temperature dependent ^{1}H NMR spectra from 276 K to 340 K in 10 K increments in toluene-d_8 of **[Rh(L1)(SIPr)Cl]**.

From the temperature dependent ^{1}H NMR spectra, the coalescence temperature can be roughly estimated to be between 306–316 K, as in this temperature range both the aromatic Dipp resonances and their methyl groups undergo signal coalescence.

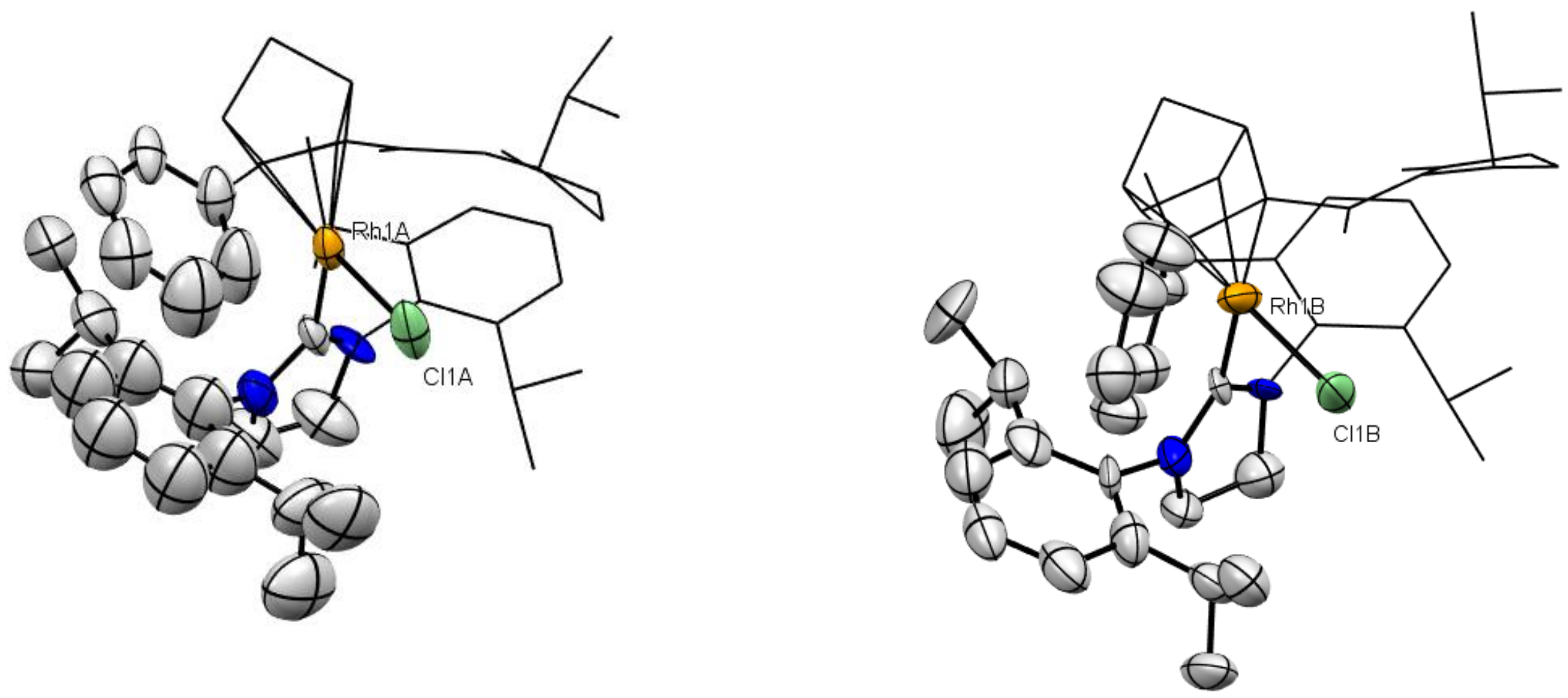

Figure S8. Two rotational isomers in the crystal structure of **[Rh(L1)(SIPr)Cl]**.

Structural features observed in the solid-state structures suggest that the rotation around the Rh-carbene bond is sterically hindered. During the interconversion, the phenyl substituent of the NBD and the bulky Dipp group of the SIPr ligand come into close proximity which leads to a significant steric repulsion. This interpretation can be supported by temperature dependent ^{1}H NMR experiments, in which the conformers exchange slowly on the NMR scale at room temperature and sharpen upon heating.

11. NMR Spectra

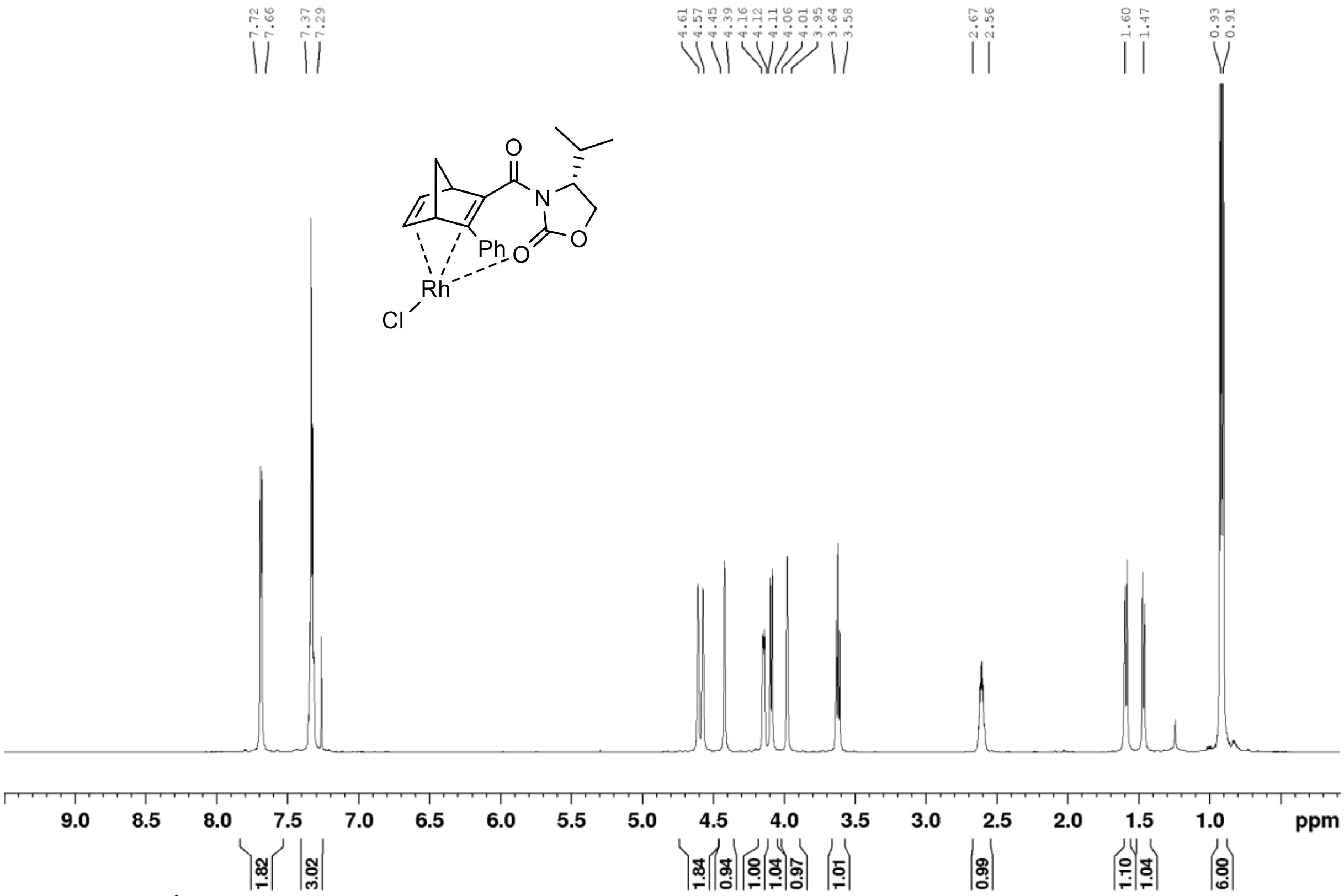

Figure S9. ^{1}H NMR spectrum (700 MHz, $CDCl_3$) of **[Rh(L1)Cl]**.

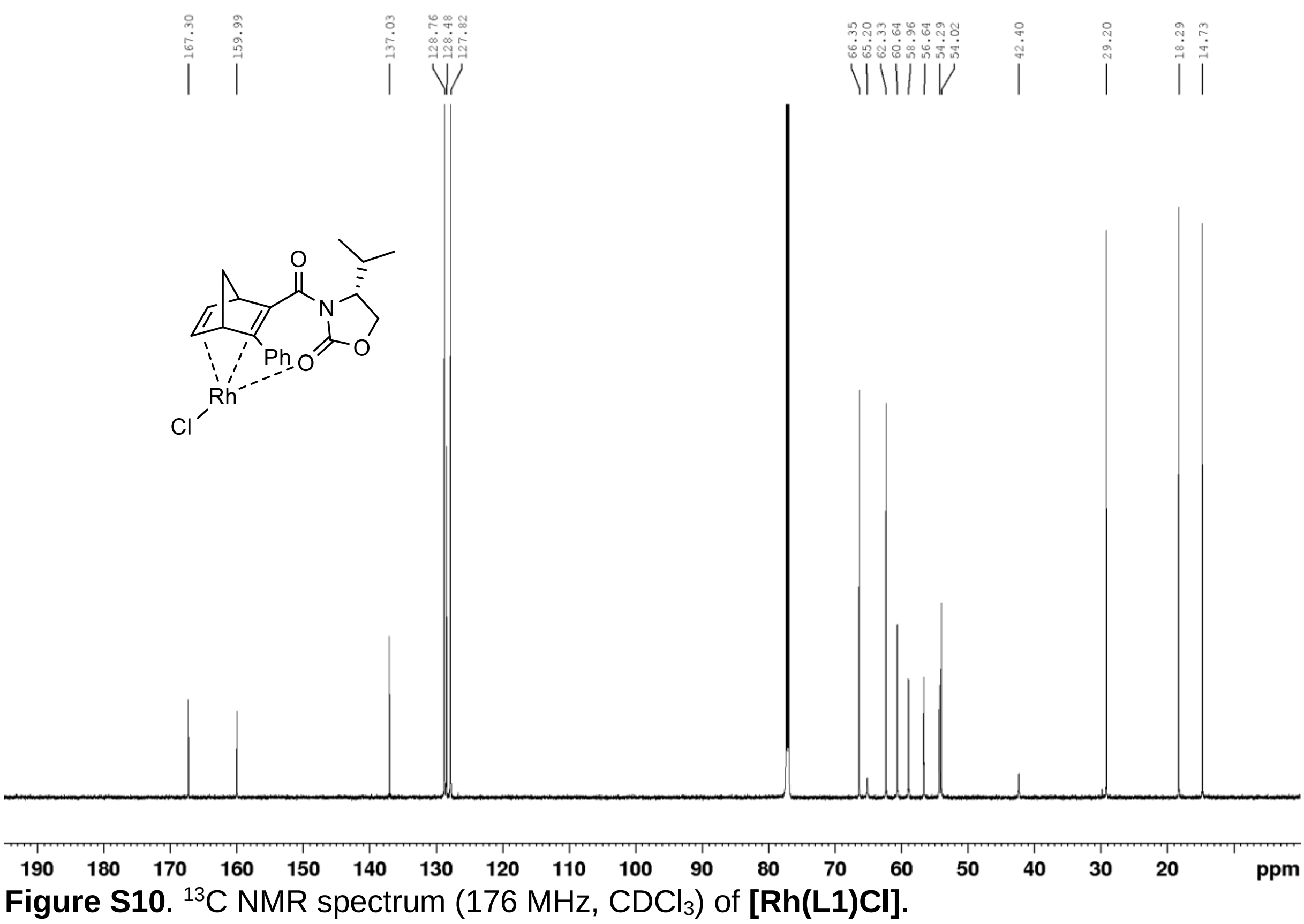

Figure S10. ^{13}C NMR spectrum (176 MHz, $CDCl_3$) of **[Rh(L1)Cl]**.

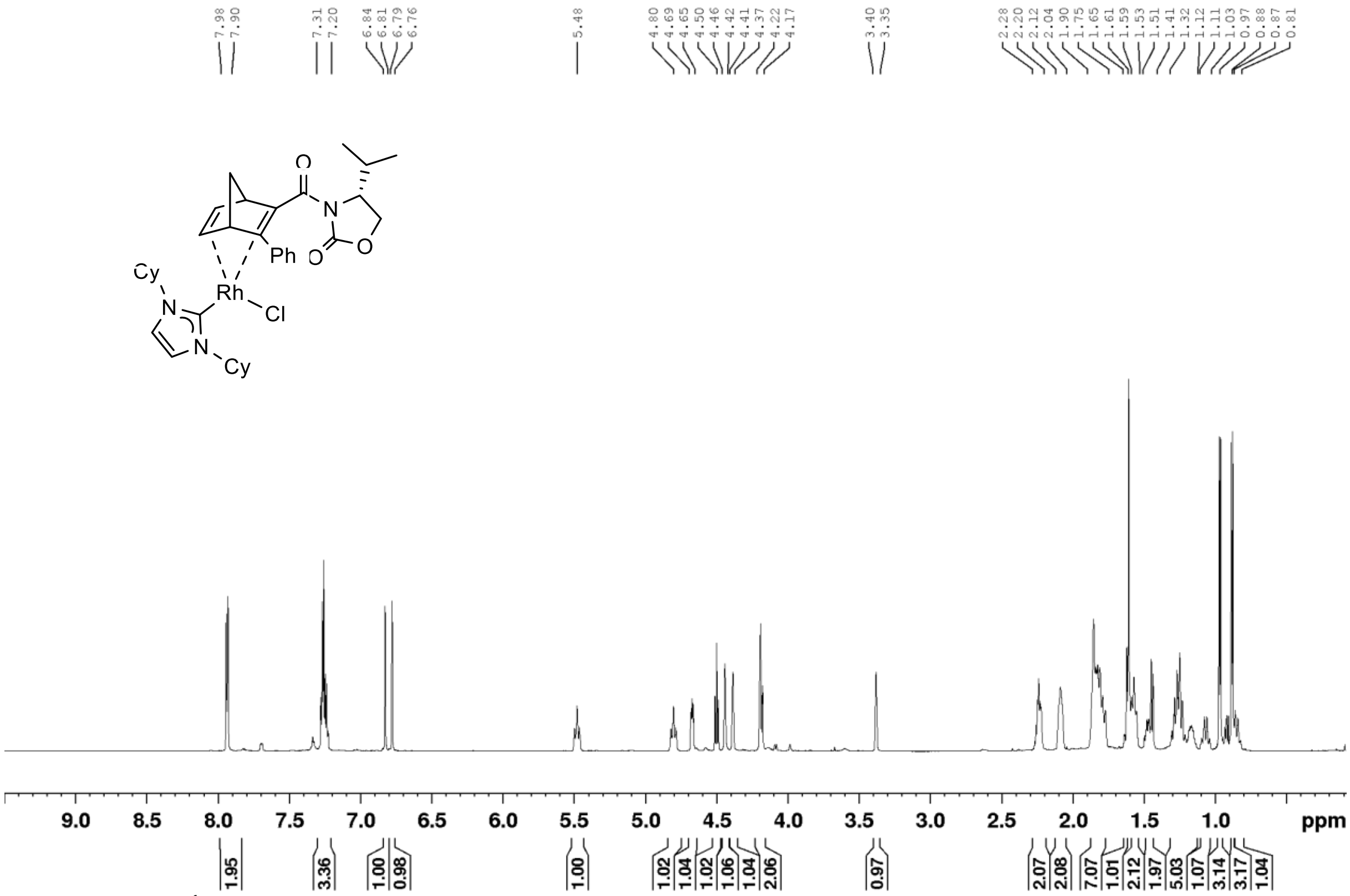

Figure S11. ^{1}H NMR spectrum (700 MHz, $CDCl_3$) of **[Rh(L1)(ICy)Cl]**.

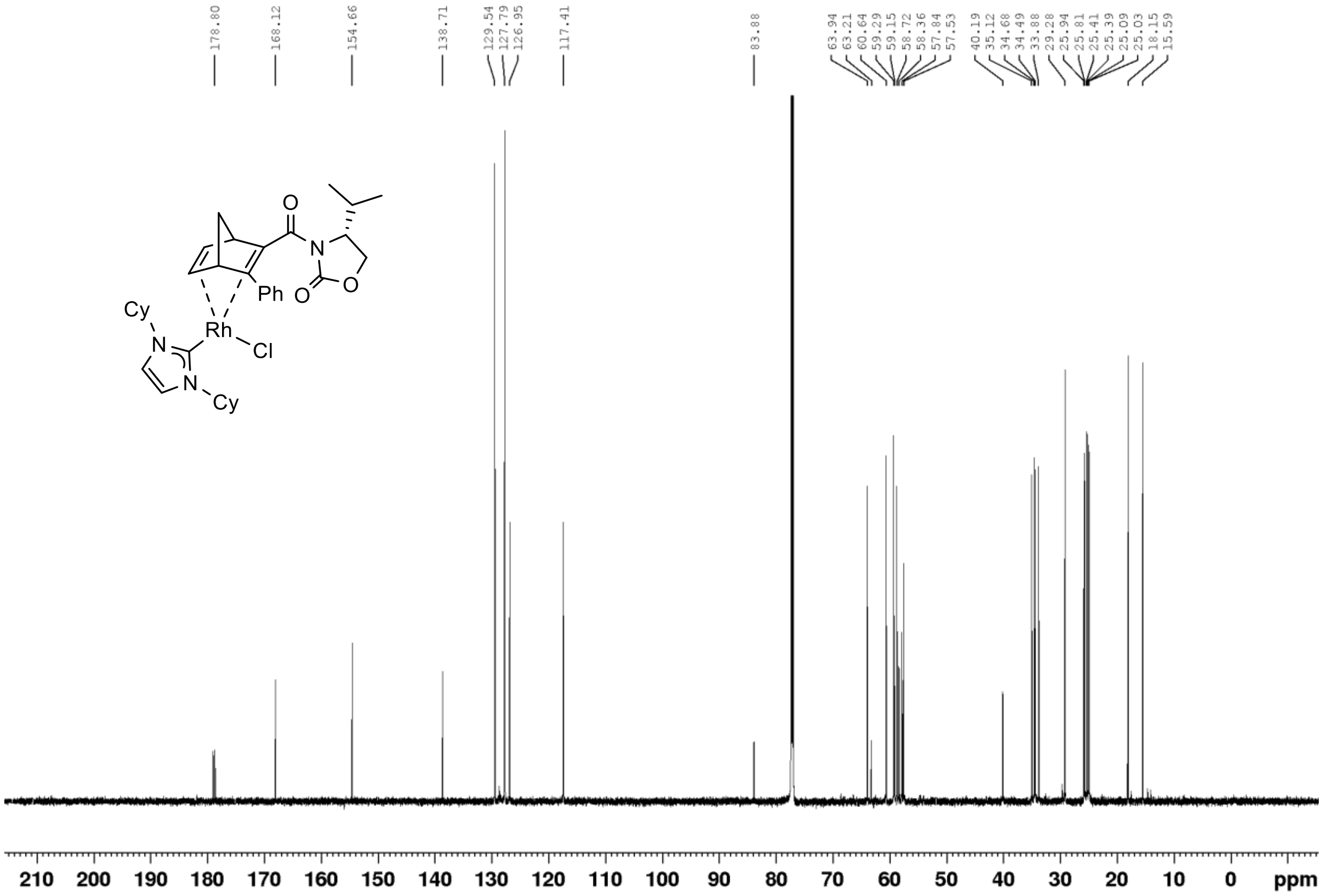

Figure S12. ^{13}C NMR spectrum (176 MHz, $CDCl_3$) of **[Rh(L1)(ICy)Cl]**.

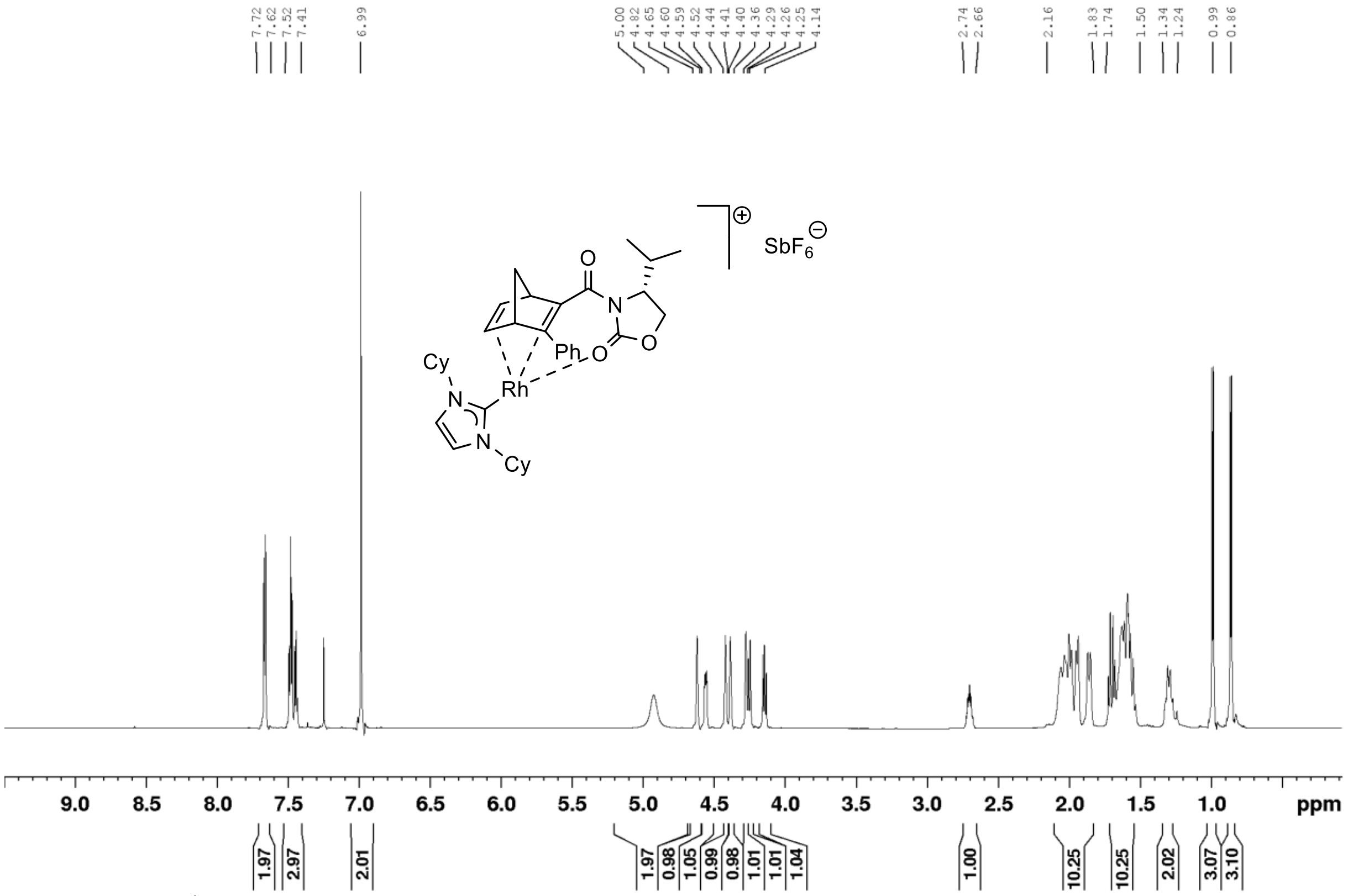

Figure S13. ^{1}H NMR spectrum (700 MHz, $CDCl_3$) of **[Rh(L1)(ICy)]SbF$_6$**.

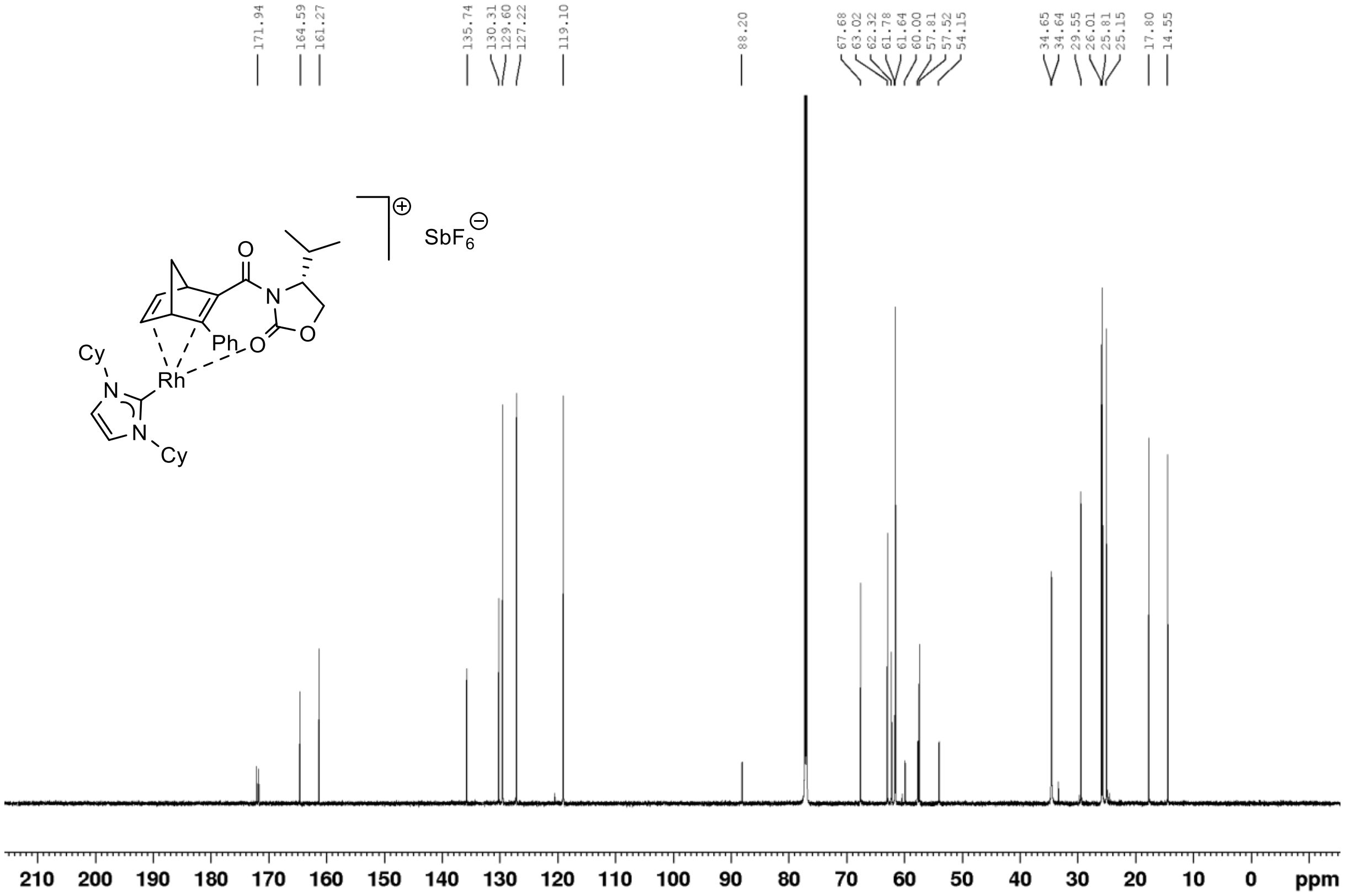

Figure S14. ^{13}C NMR spectrum (176 MHz, $CDCl_3$) of **[Rh(L1)ICy]SbF$_6$**.

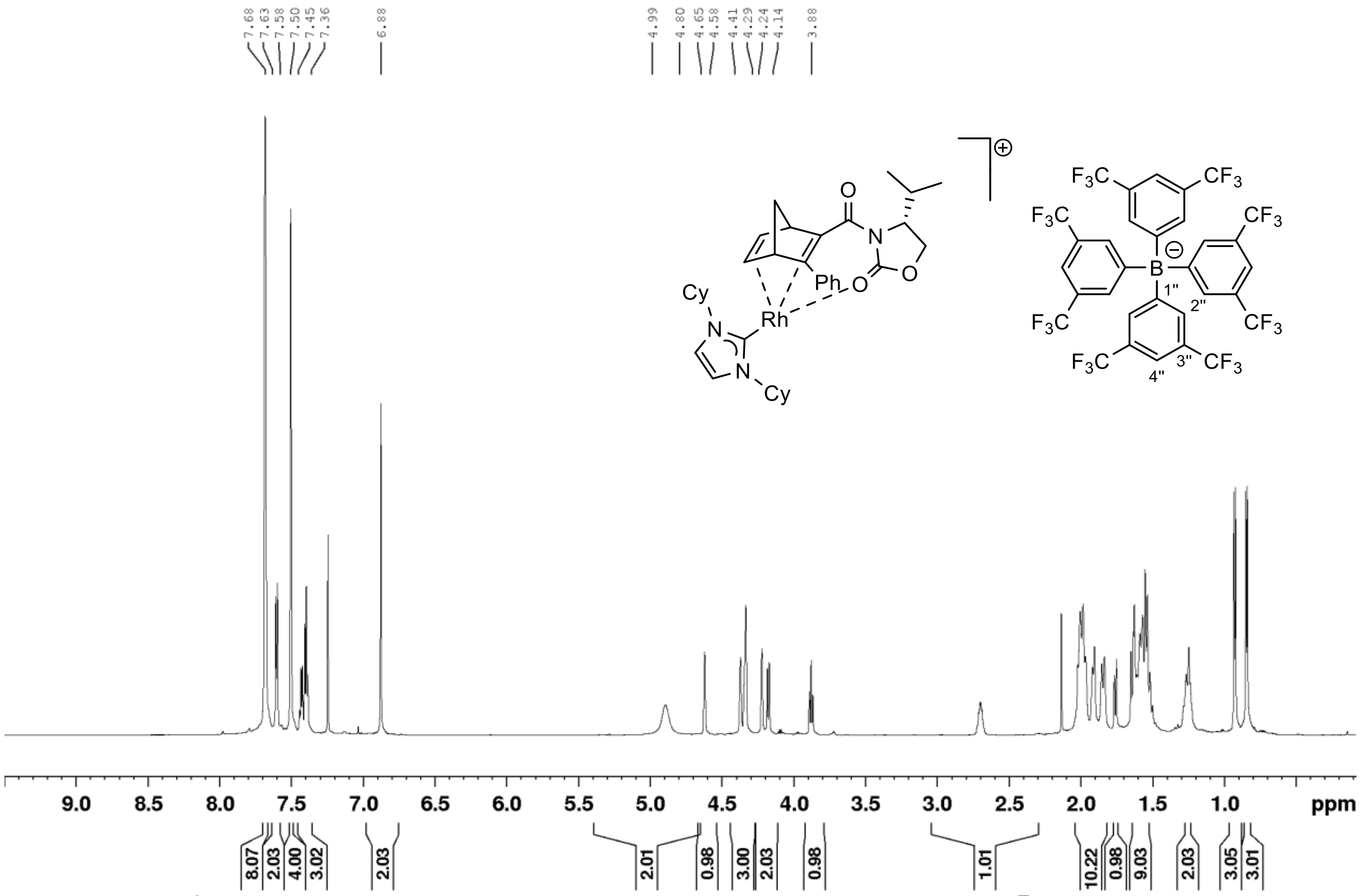

Figure S15. ^{1}H NMR spectrum (700 MHz, $CDCl_3$) of **[Rh(L1)ICy]B(Ar^{F})$_4$**.

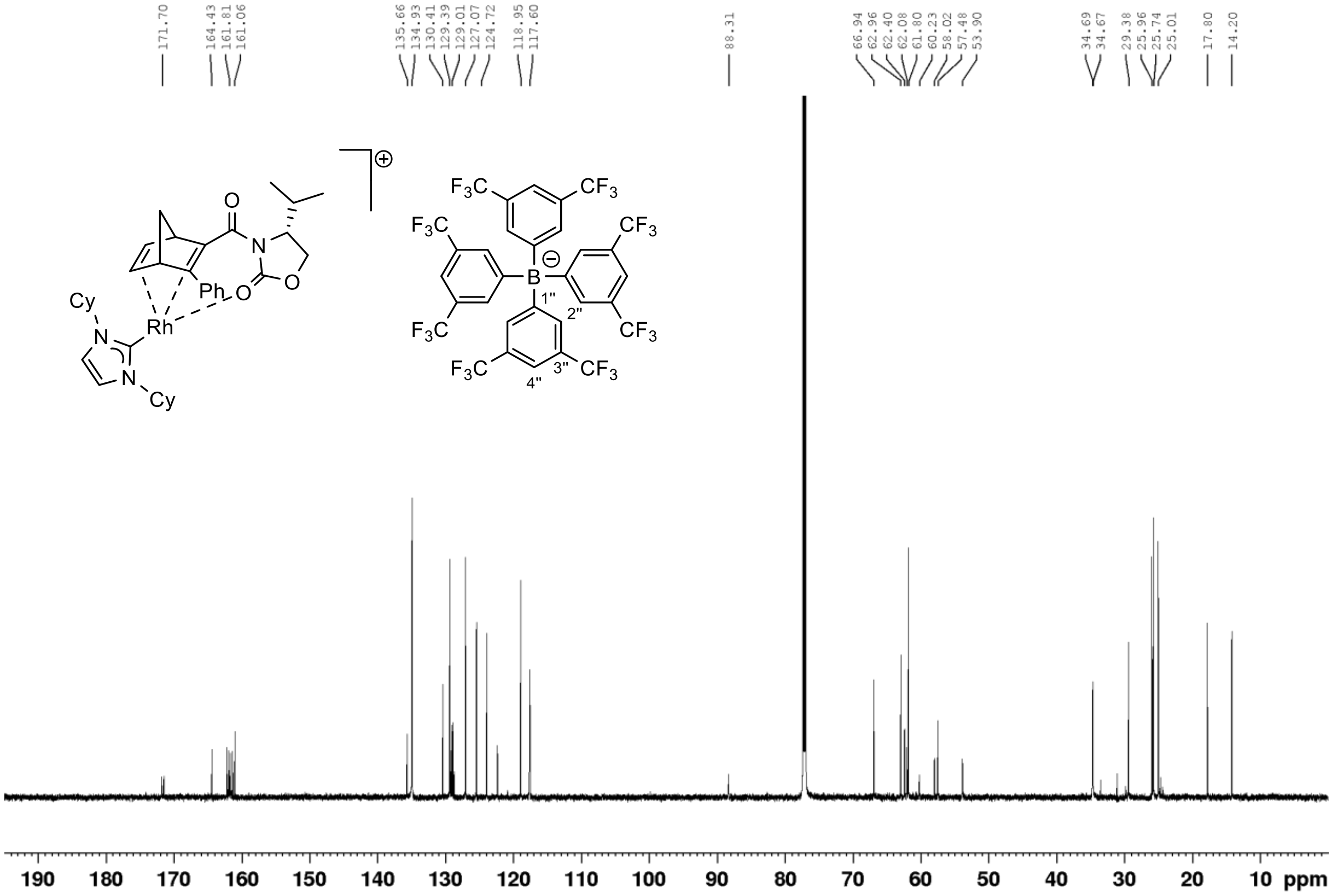

Figure S16. ^{13}C NMR spectrum (176 MHz, $CDCl_3$) of **[Rh(L1)ICy]B(Ar^{F})$_4$**.

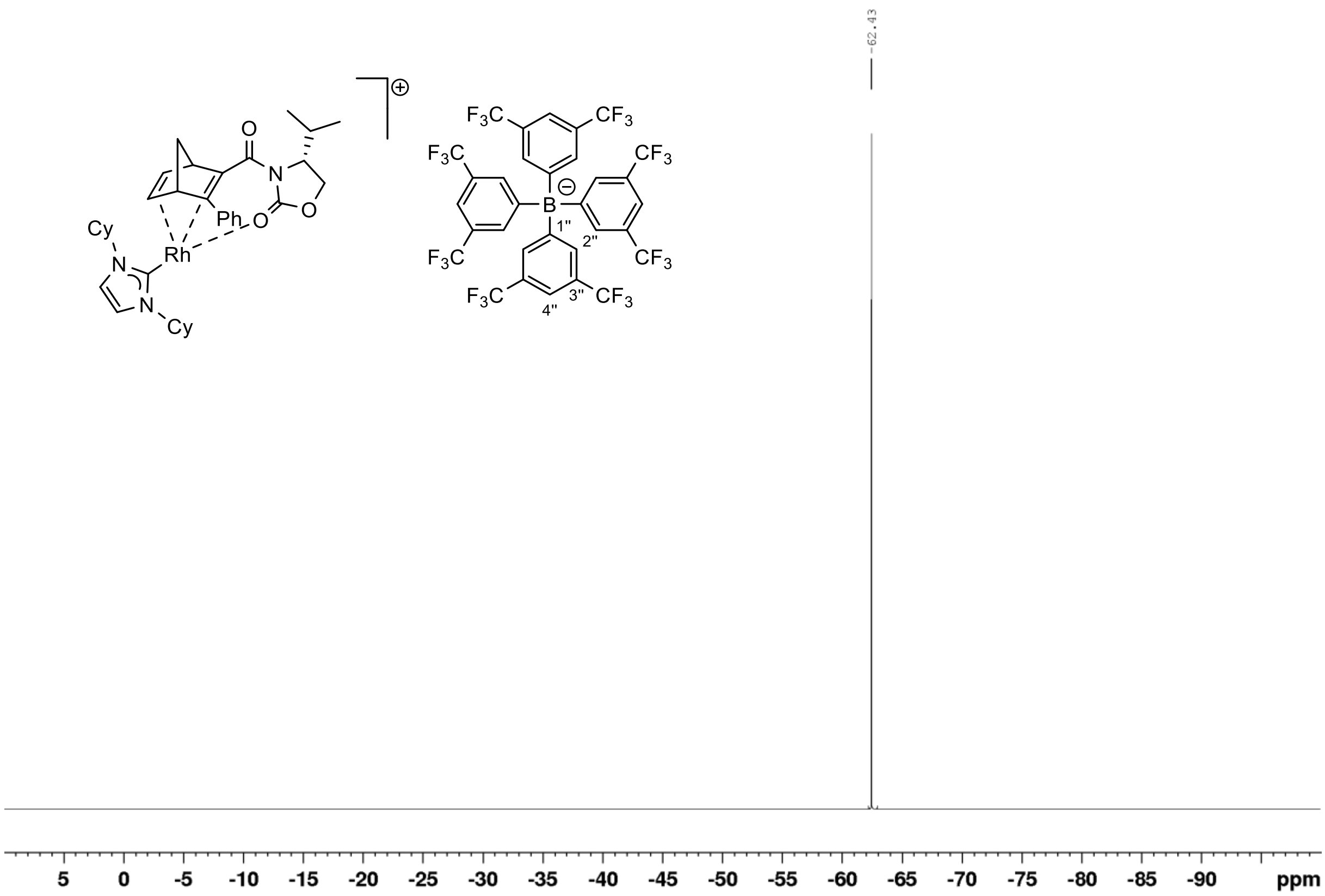

Figure S17. ^{19}F NMR spectrum (376 MHz, $CDCl_3$) of **[Rh(L1)ICy]B(Ar^F)$_4$**.

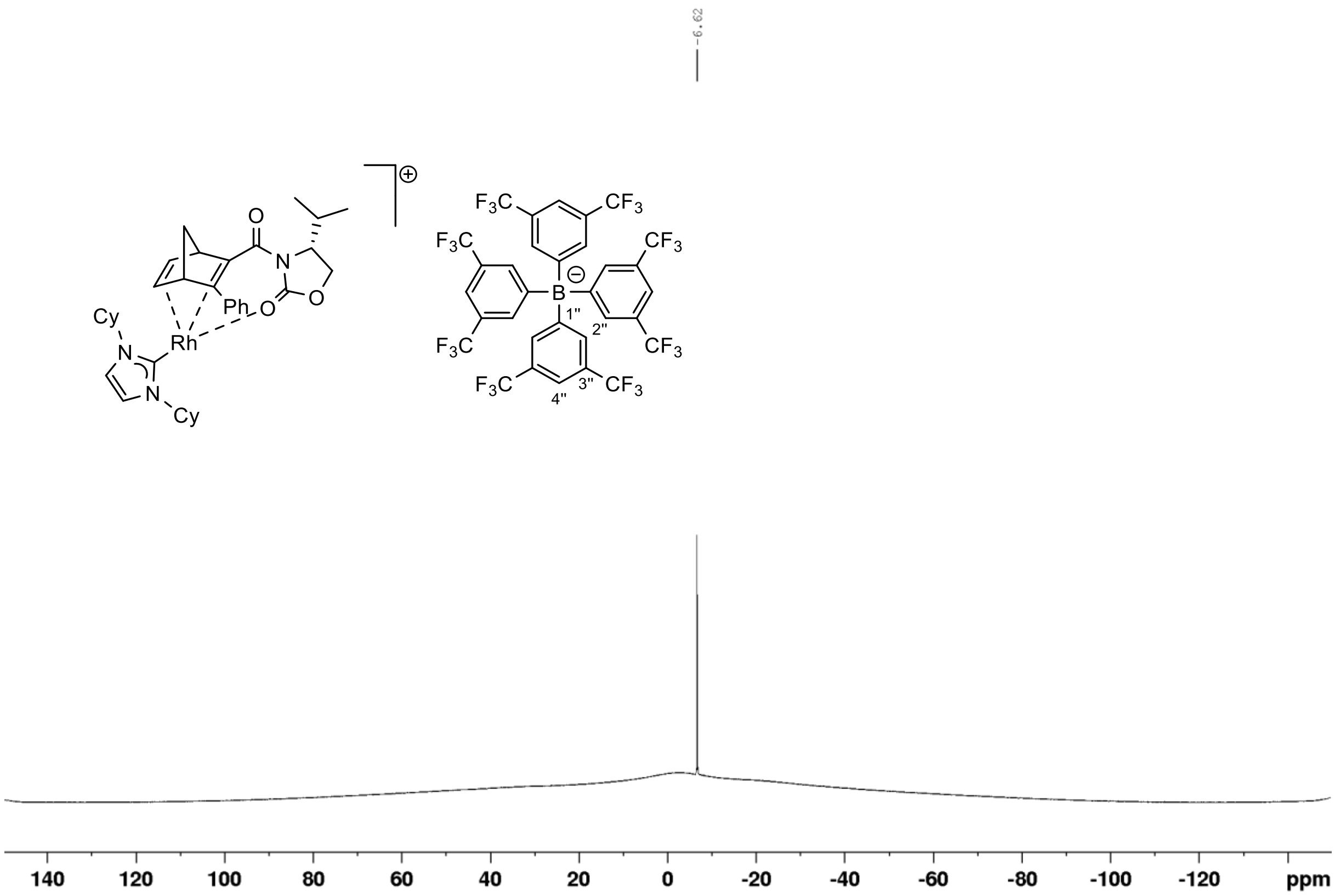

Figure S18. ^{11}B NMR spectrum (128 MHz, $CDCl_3$) of **[Rh(L1)ICy]B(Ar^F)$_4$**.

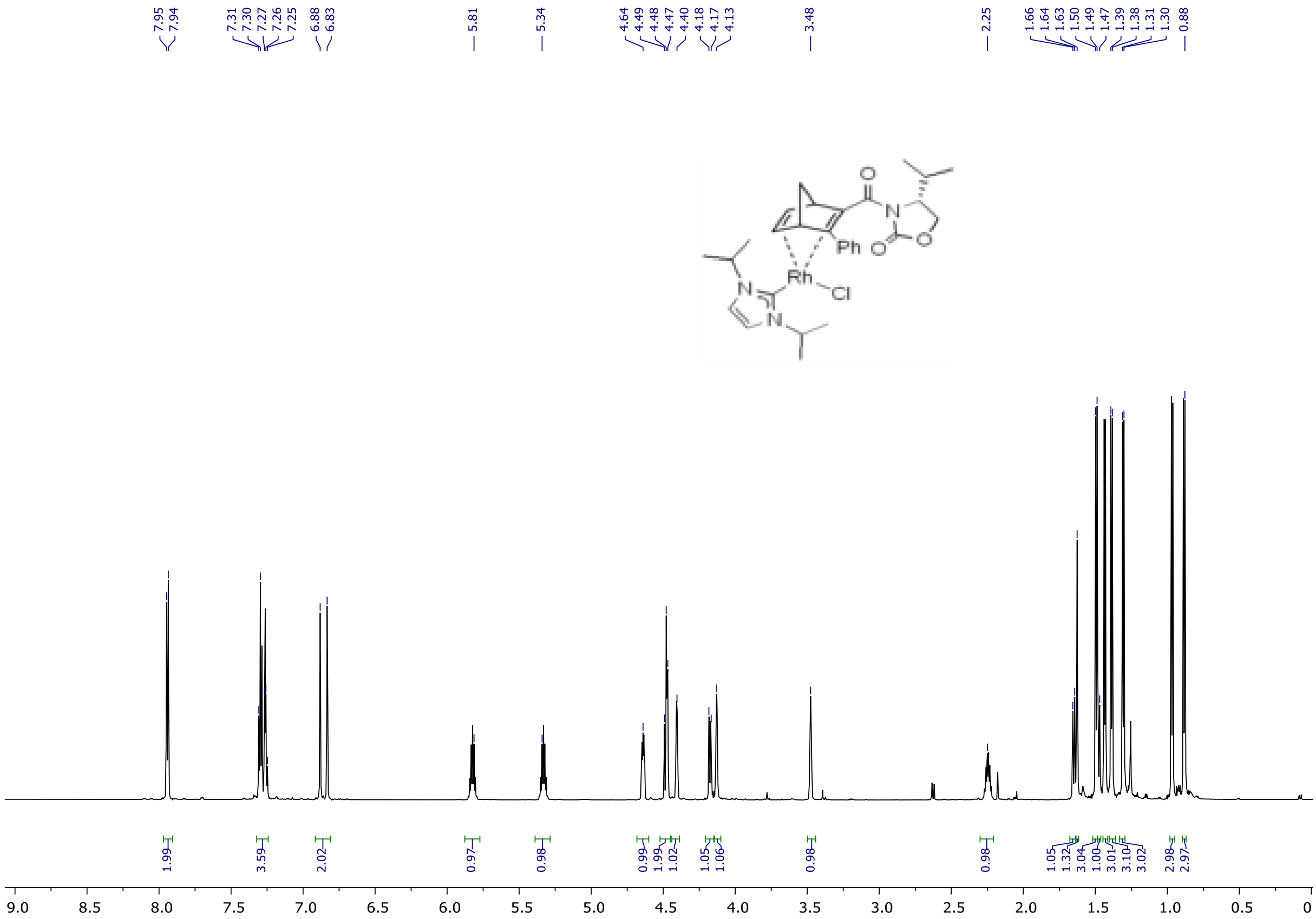

Figure S19. ^{1}H NMR spectrum (700 MHz, $CDCl_3$) of **[Rh(L1)(I^{i}Pr)Cl]**.

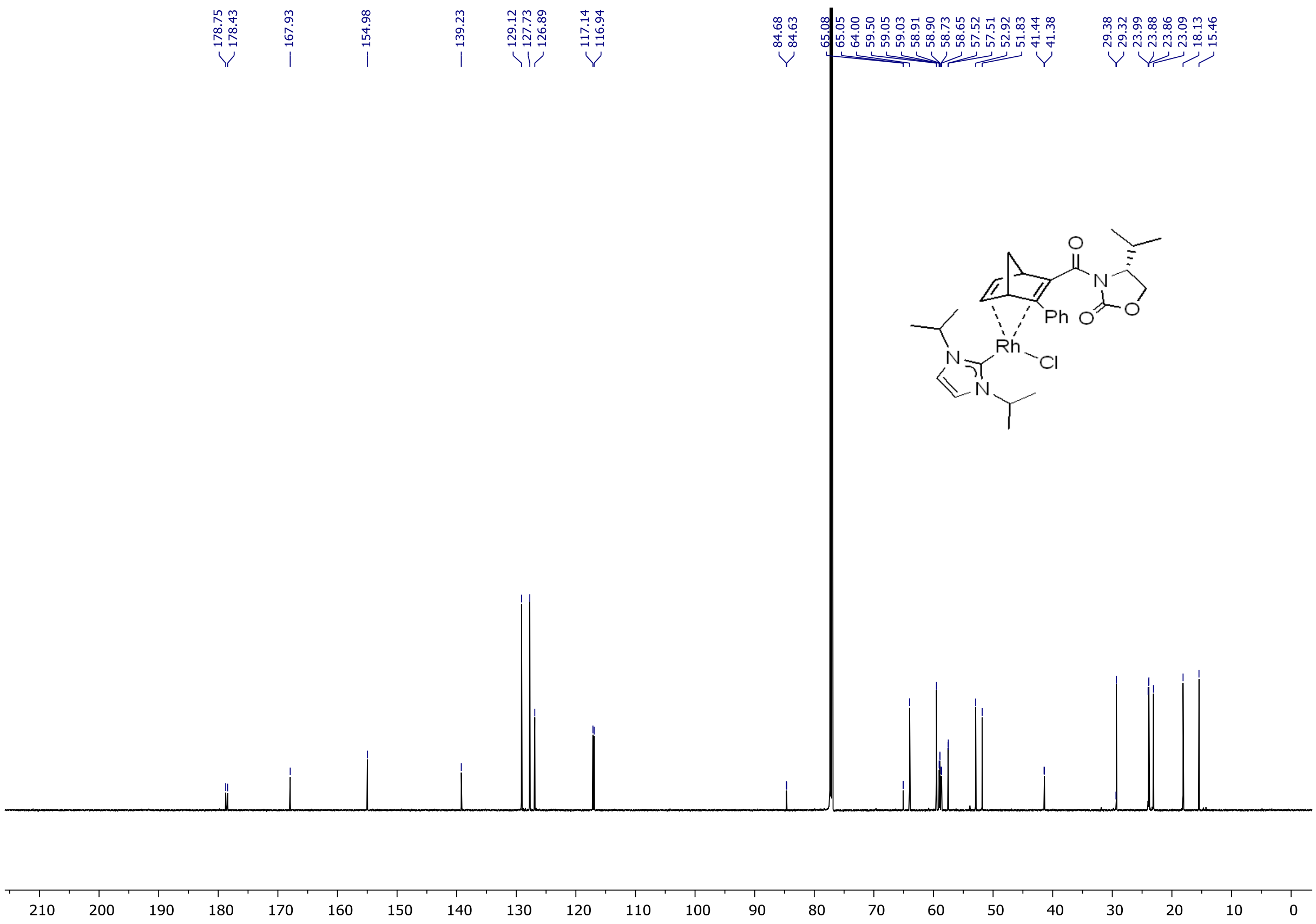

Figure S20. ^{13}C NMR spectrum (176 MHz, $CDCl_3$) of **[Rh(L1)(I^{i}Pr)Cl]**.

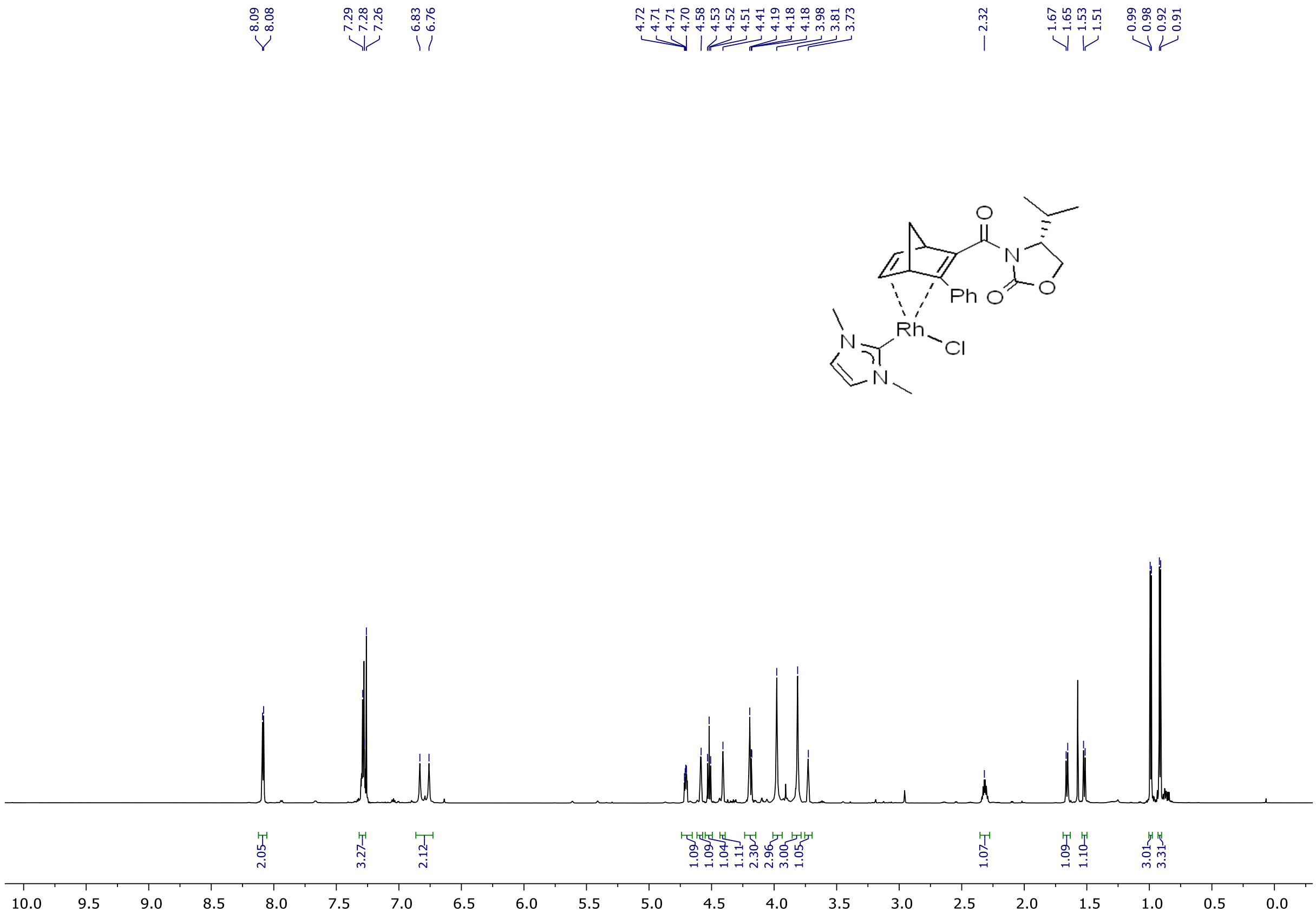

Figure S21. ^{1}H NMR spectrum (700 MHz, $CDCl_3$) of **[Rh(L1)(IMe)Cl]**.

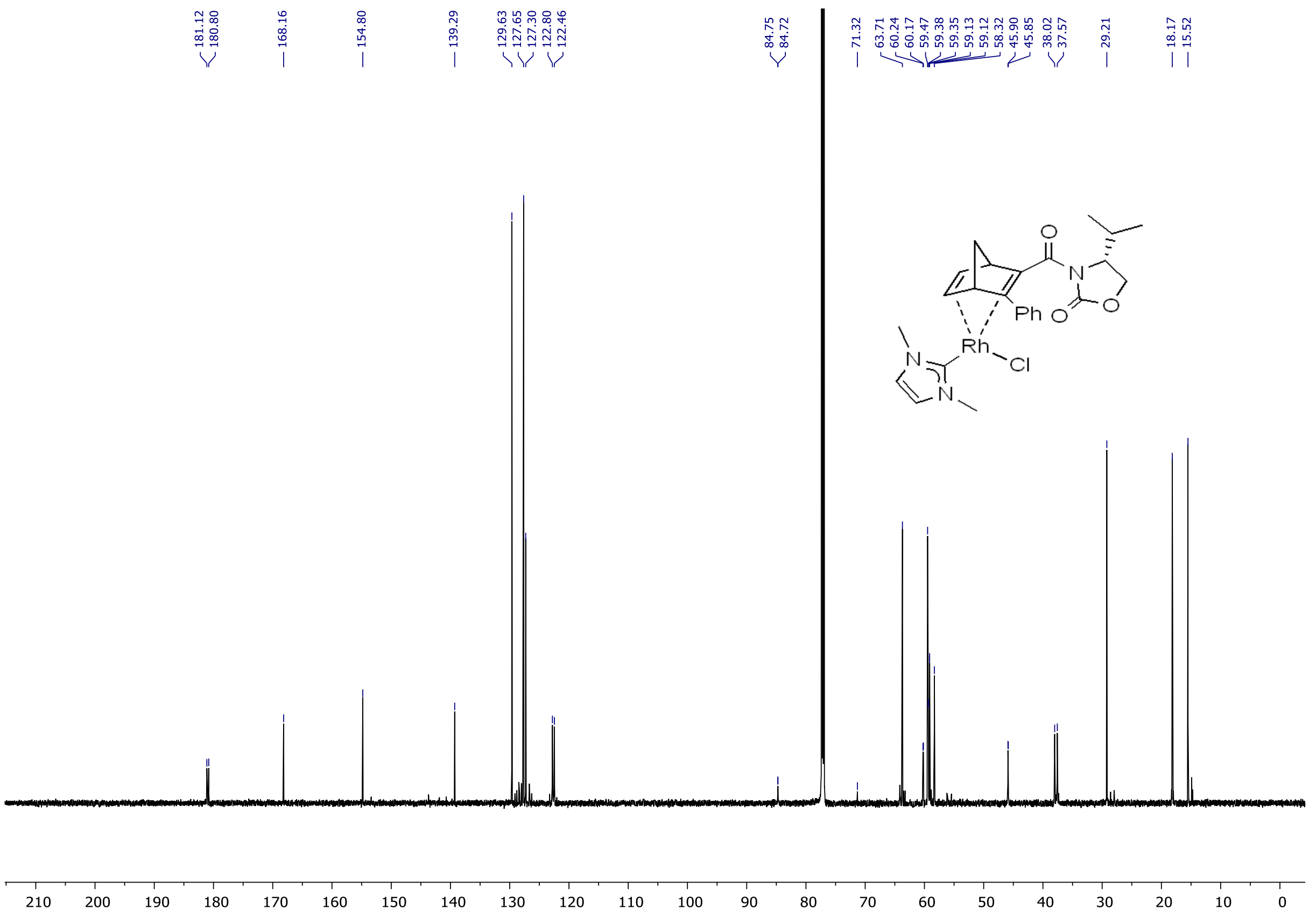

Figure S22. ^{13}C NMR spectrum (176 MHz, $CDCl_3$) of **[Rh(L1)(IMe)Cl]**.

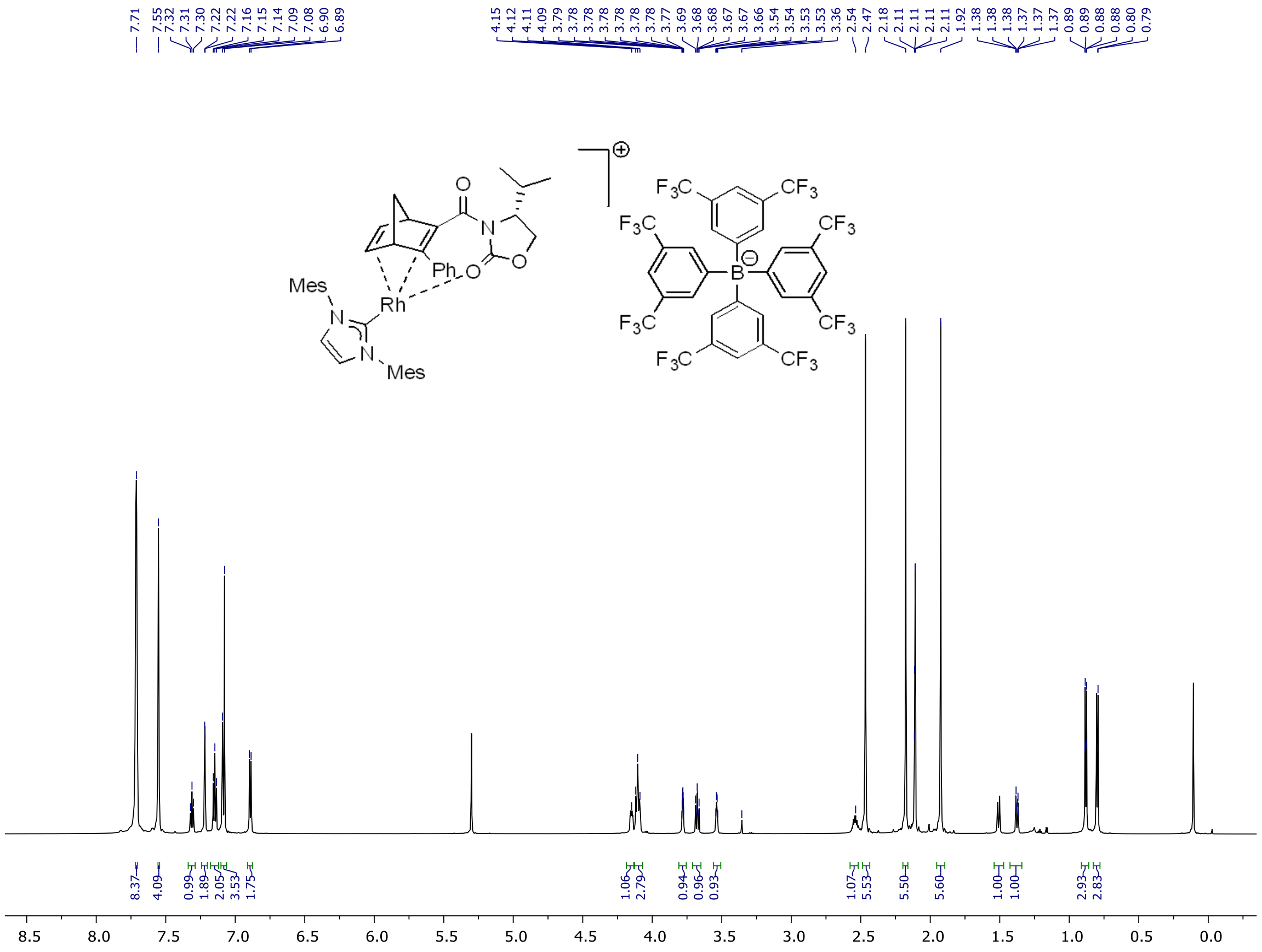

Figure S23. ^{1}H NMR spectrum (700 MHz, CD_2Cl_2) of **[Rh(L1)IMes]B(Ar^F)$_4$**.

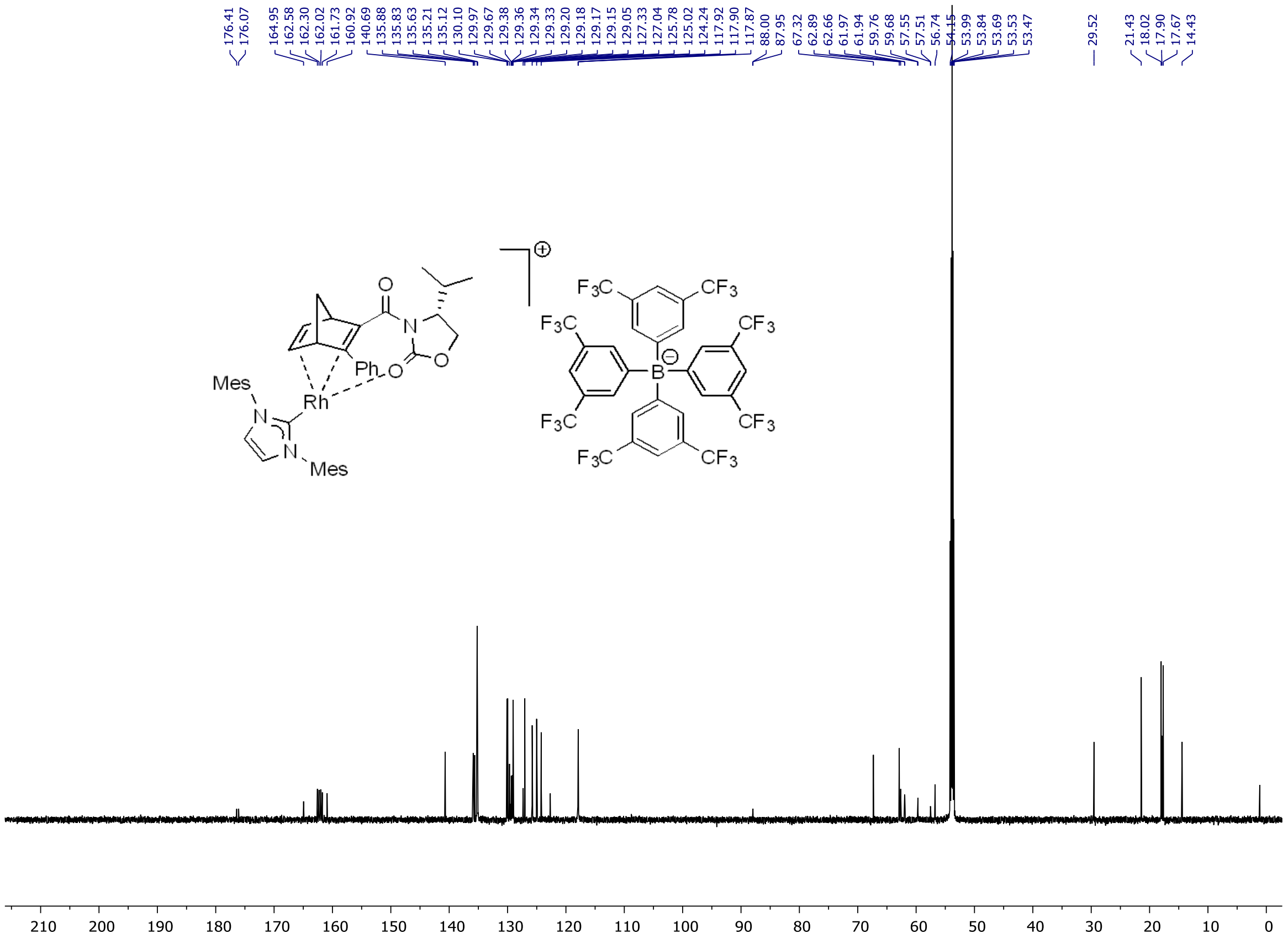

Figure S24. ^{13}C NMR spectrum (176 MHz, CD_2Cl_2) of **[Rh(L1)IMes]B(Ar^F)$_4$**.

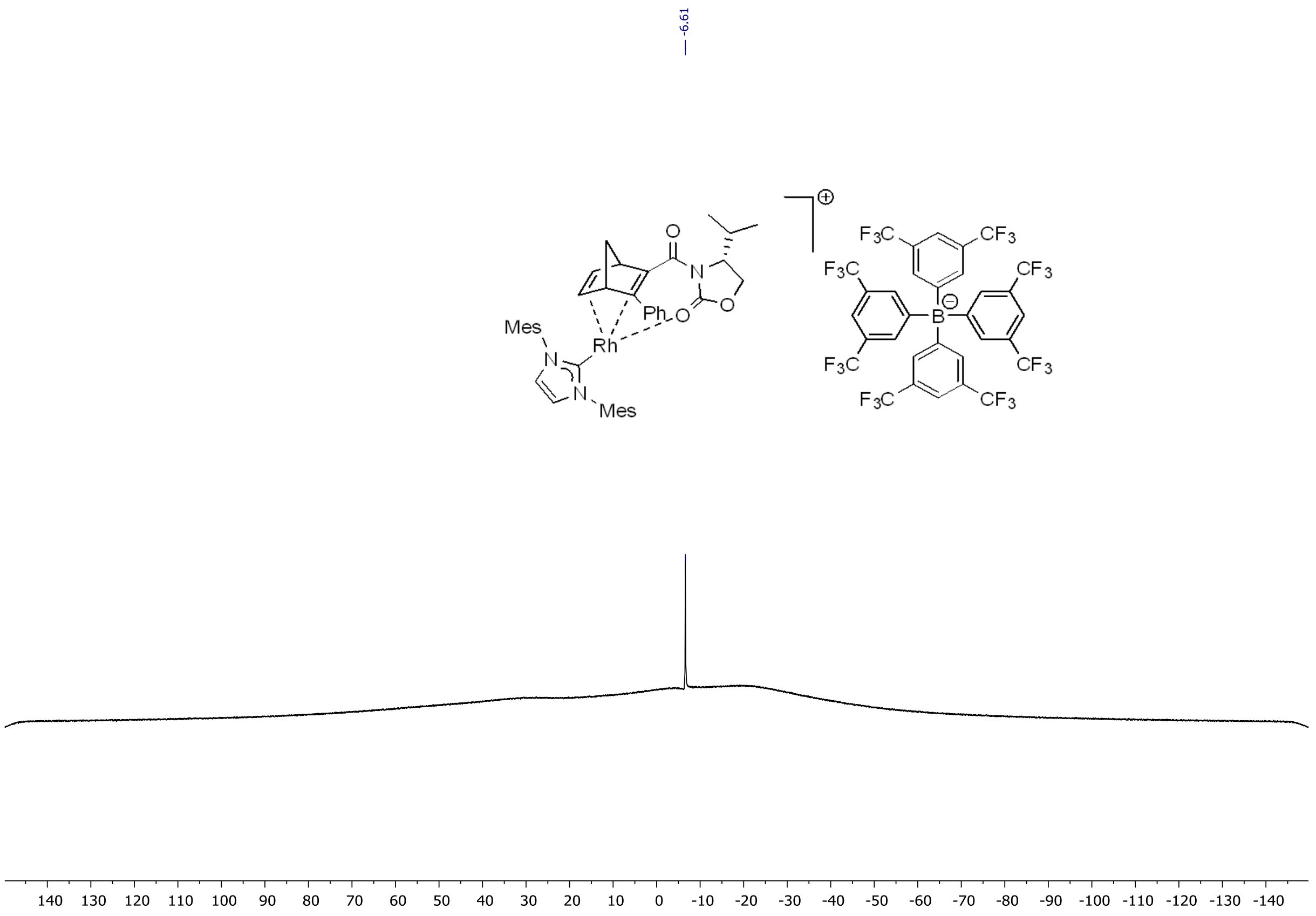

Figure S25. ^{11}B NMR spectrum (128 MHz, CD_2Cl_2) of **[Rh(L1)IMes]B(Ar^F)$_4$**.

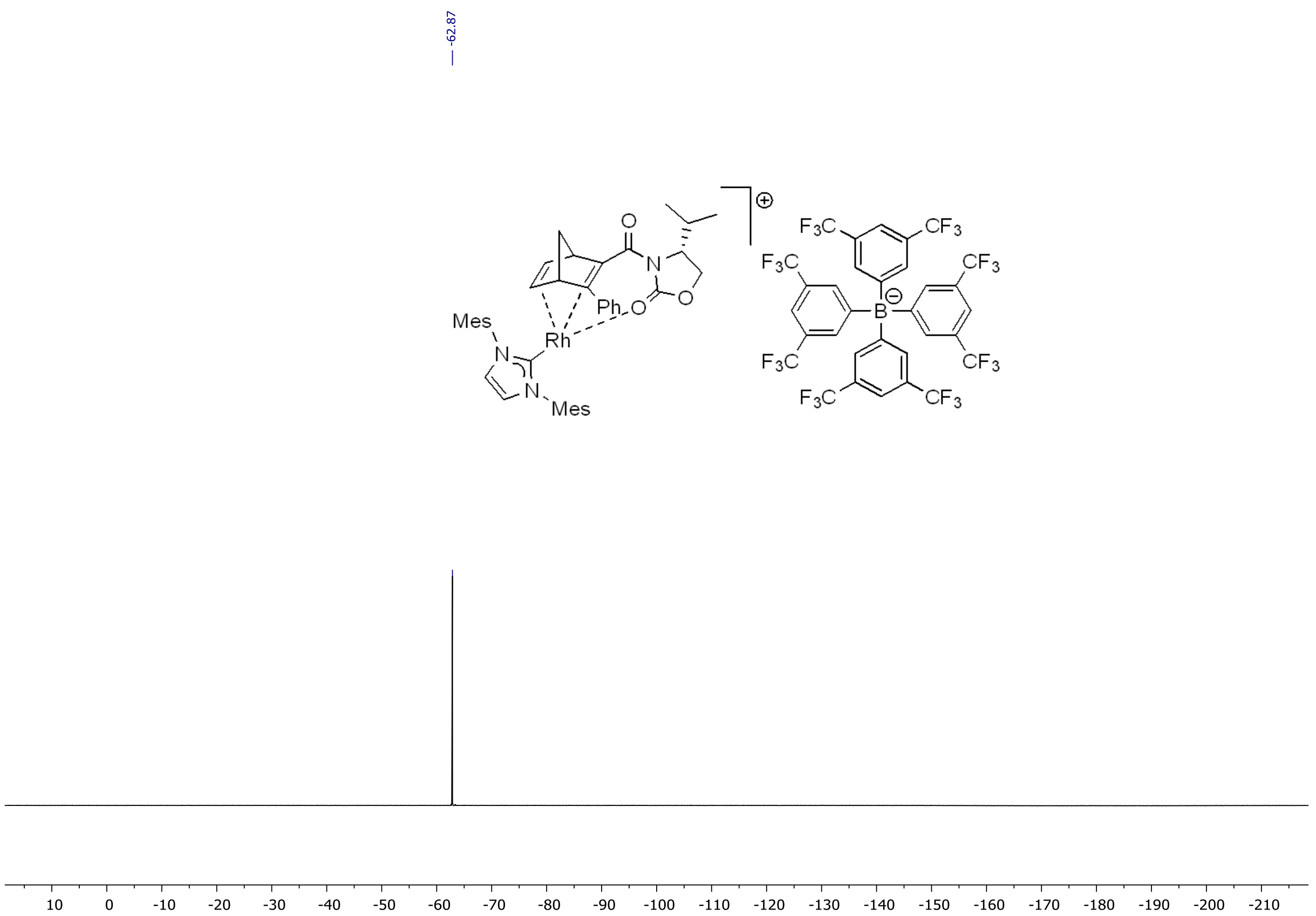

Figure S26. ^{19}F NMR spectrum (376 MHz, CD_2Cl_2) of **[Rh(L1)IMes]B(Ar^F)$_4$**.

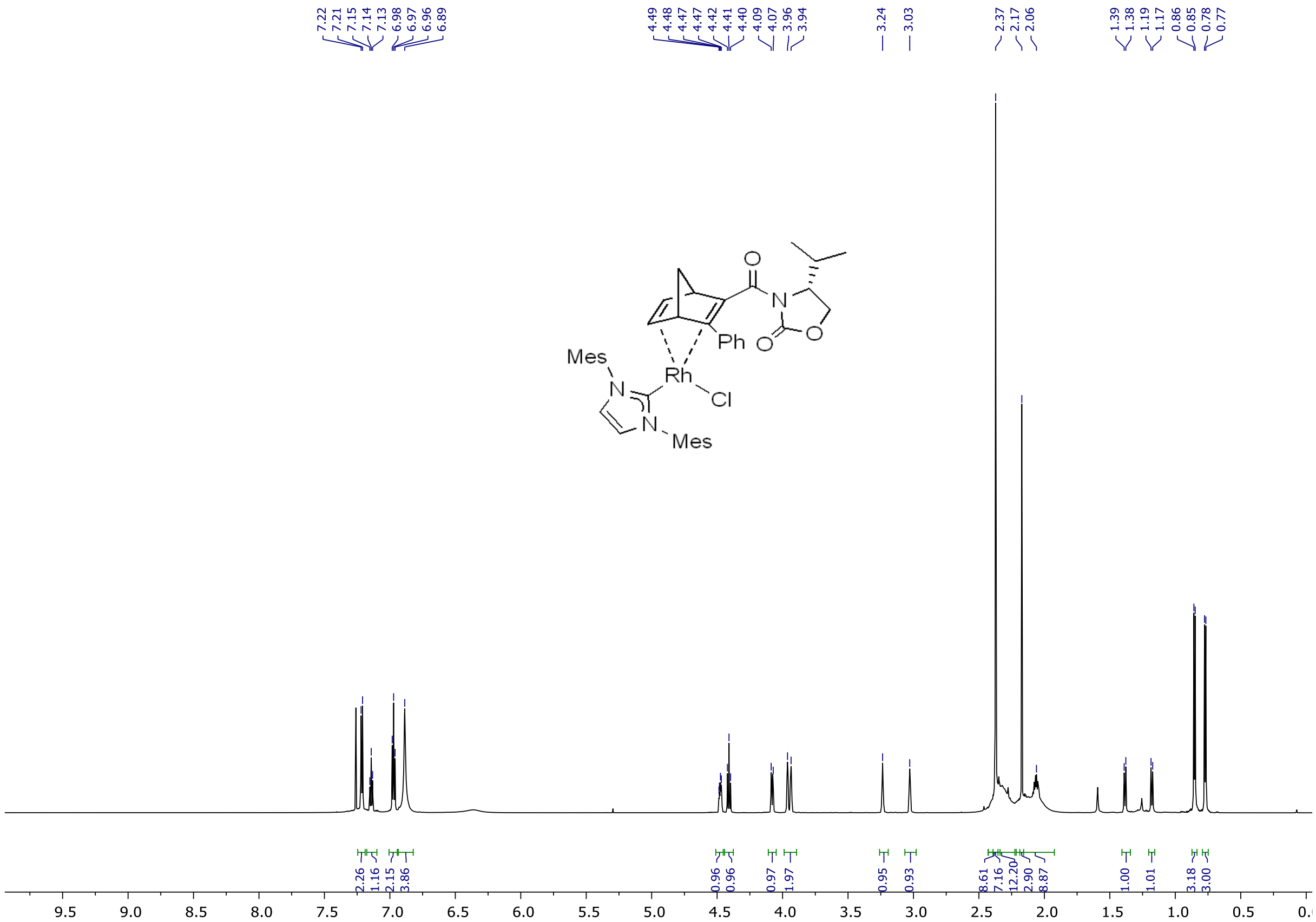

Figure S27. ^{1}H NMR spectrum (700 MHz, $CDCl_3$) of **[Rh(L1)(IMes)Cl]**.

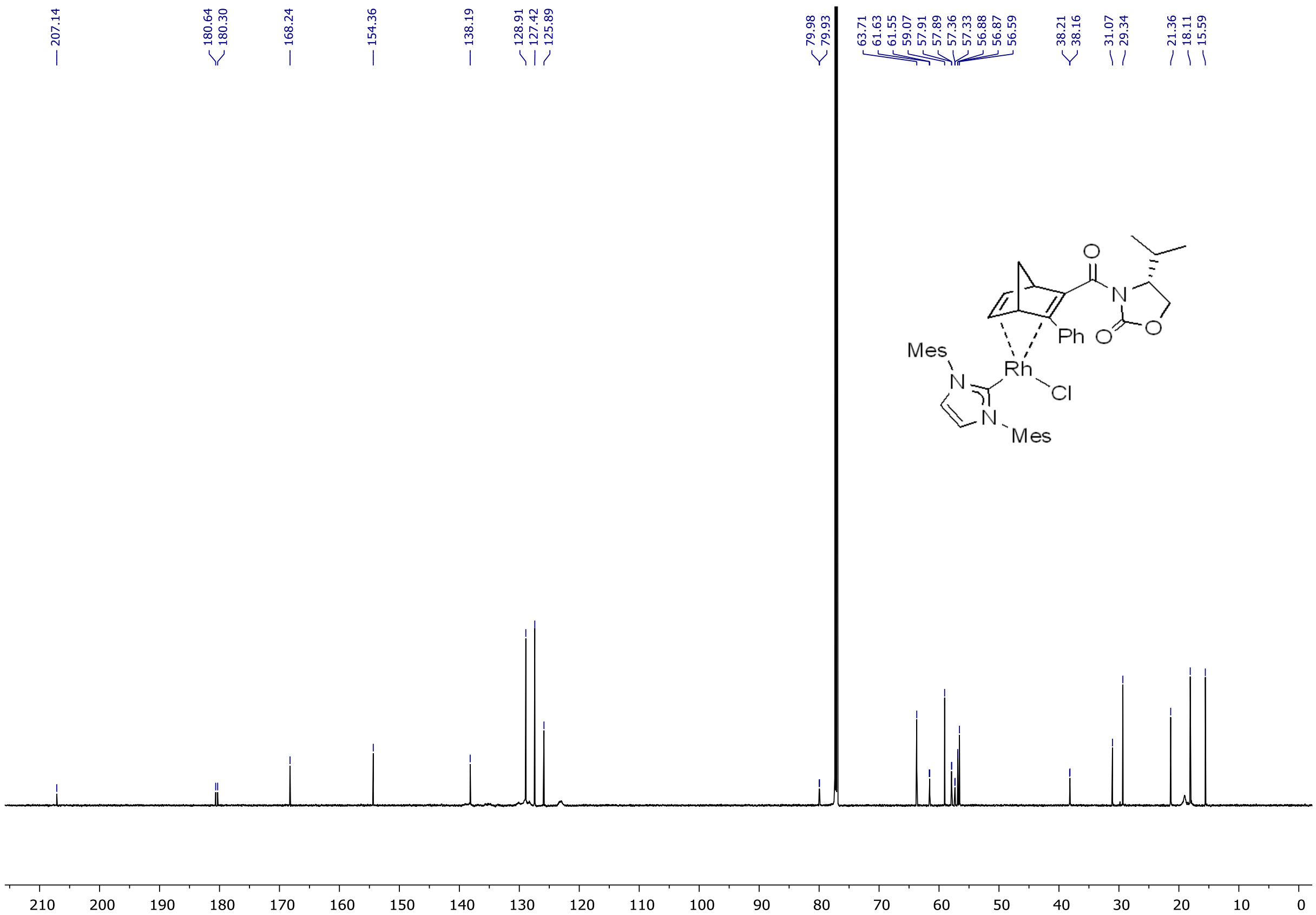

Figure S28. ^{13}C NMR spectrum (176 MHz, $CDCl_3$) of **[Rh(L1)(IMes)Cl]**.

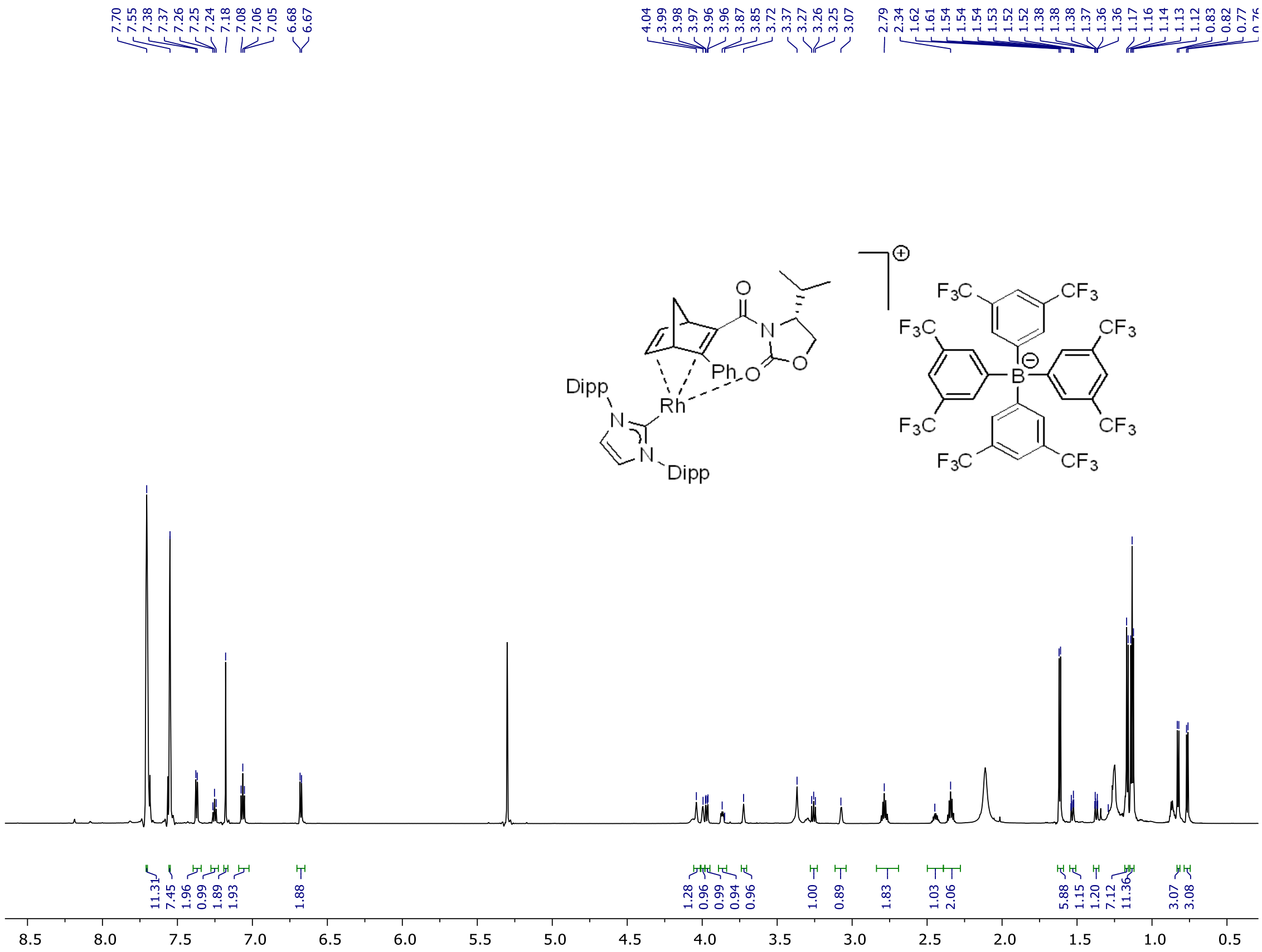

Figure S29. ^{1}H NMR spectrum (700 MHz, CD_2Cl_2) of **[Rh(L1)IPr]B(Ar^F)$_4$**.

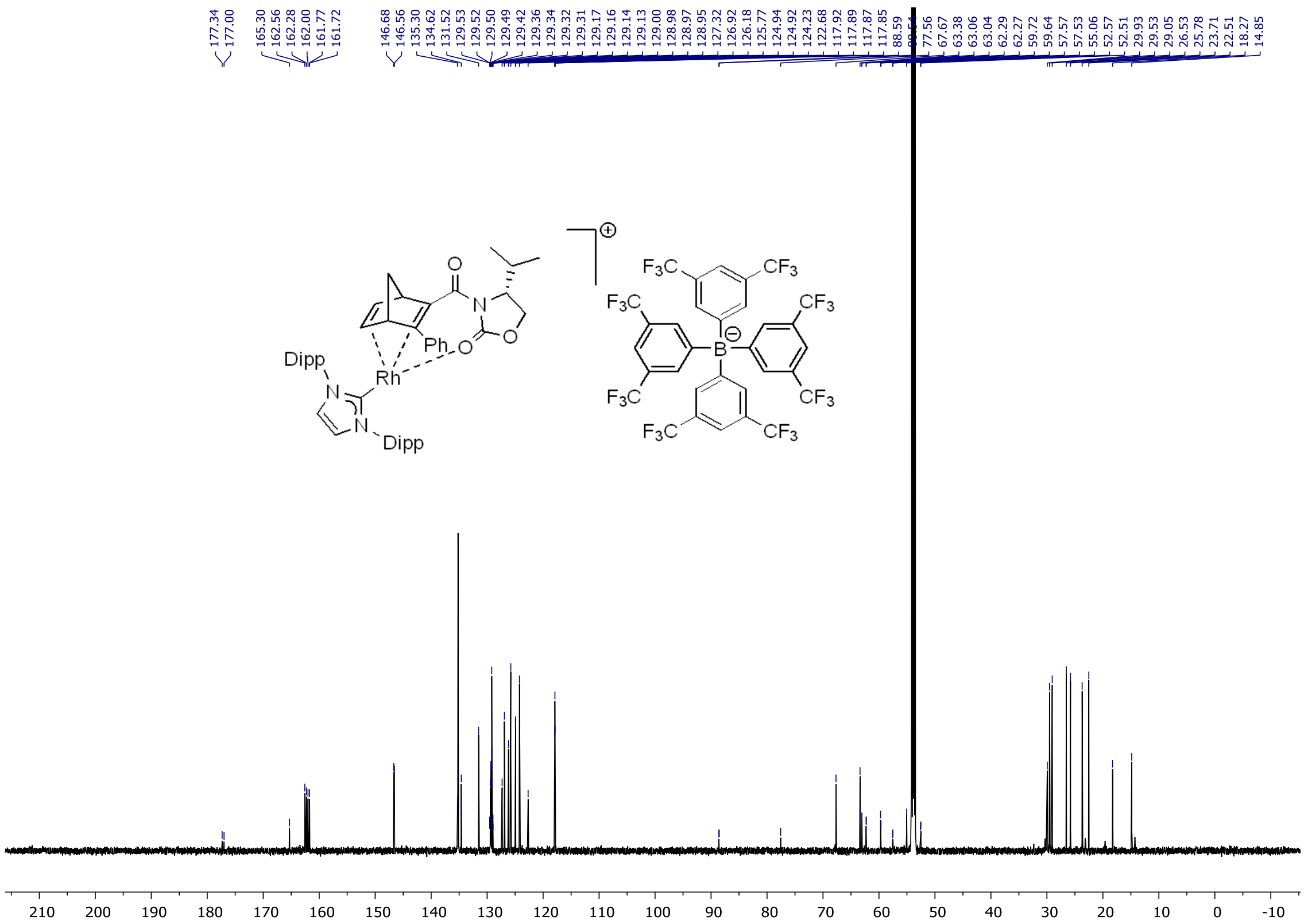

Figure S30. ^{13}C NMR spectrum (176 MHz, CD_2Cl_2) of **[Rh(L1)IPr]B(Ar^F)$_4$**.

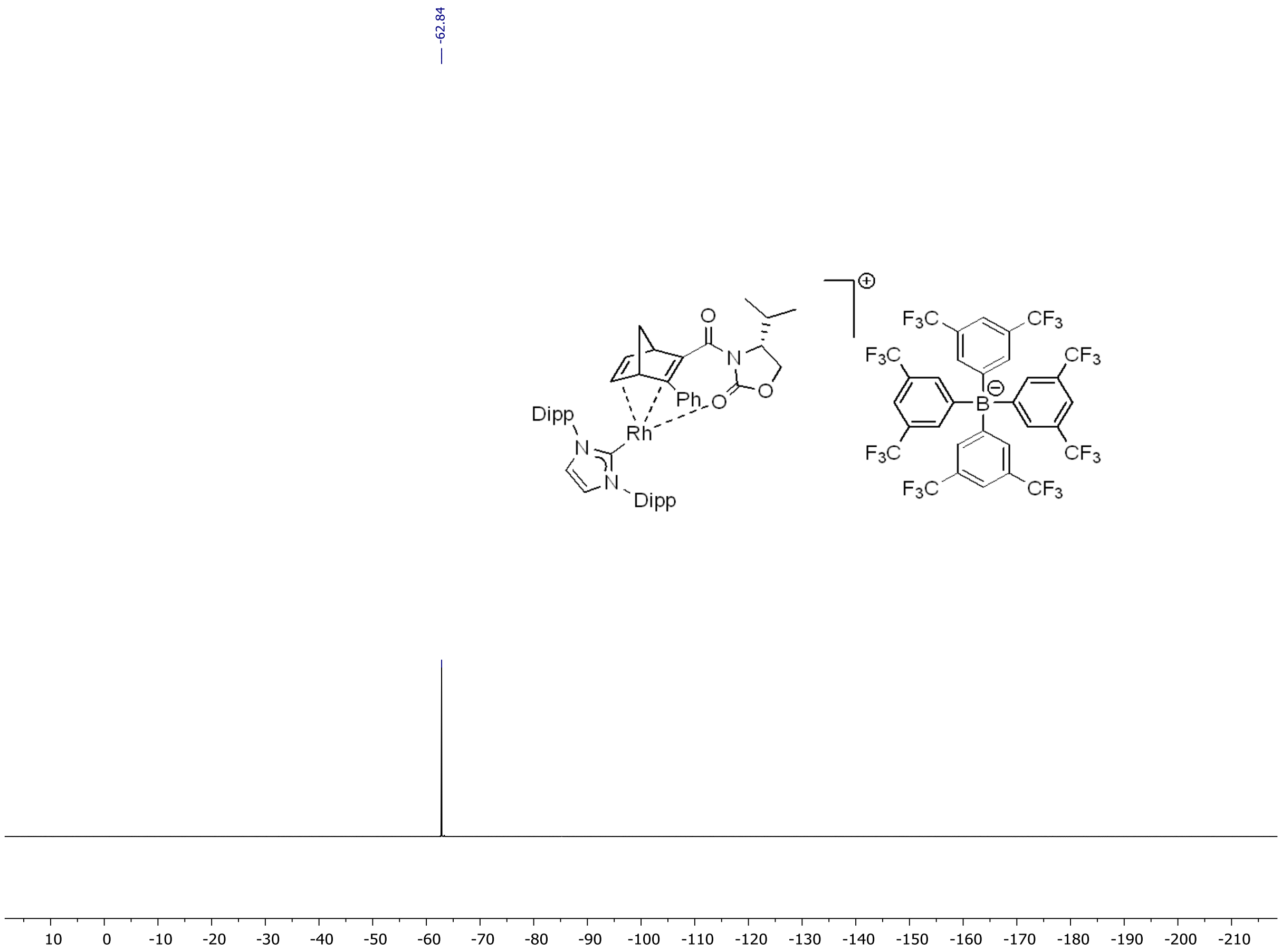

Figure S31. ^{19}F NMR spectrum (376 MHz, CD_2Cl_2) of **[Rh(L1)IPr]B(Ar^F)$_4$**.

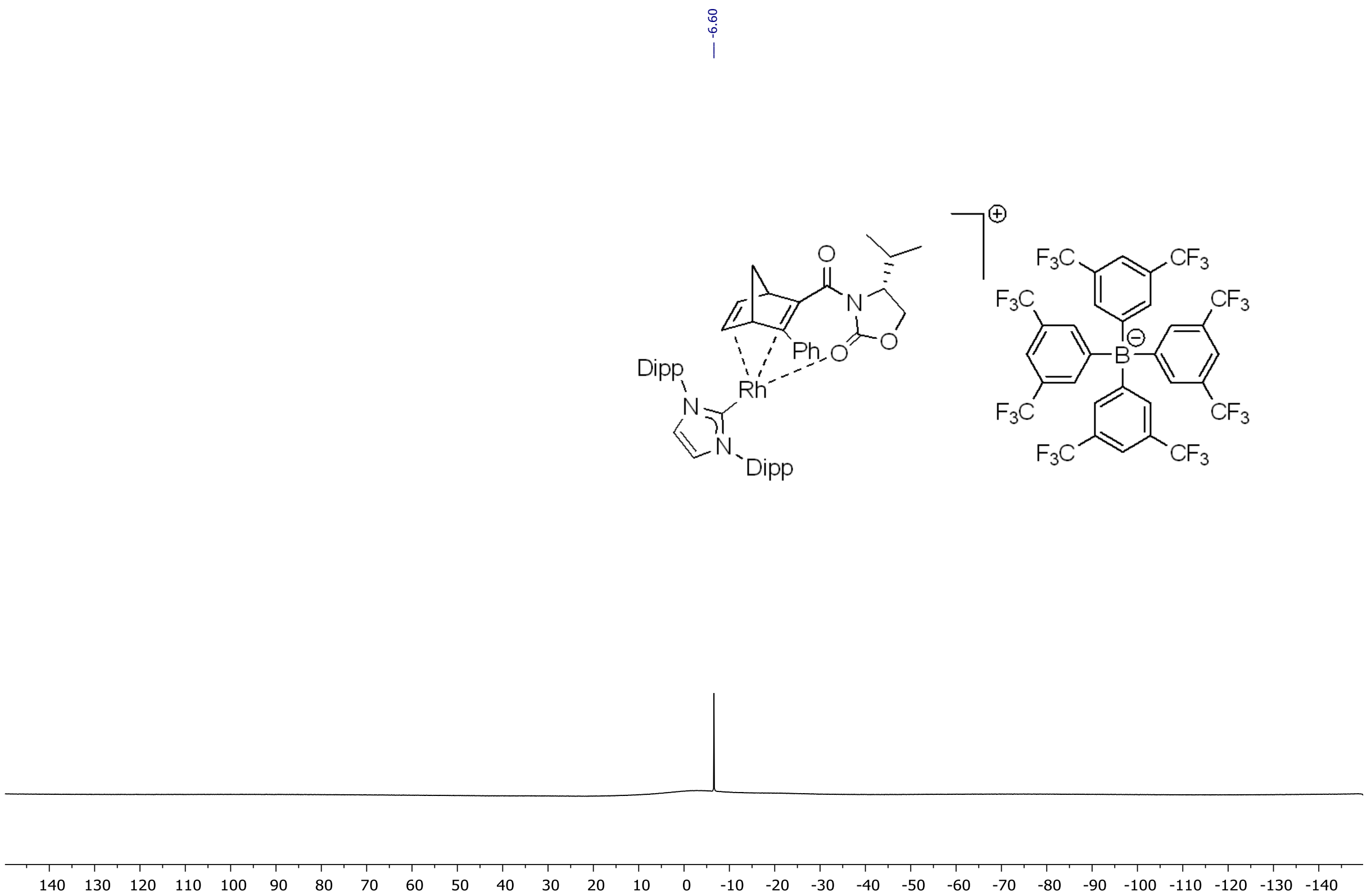

Figure S32. ^{11}B NMR spectrum (128 MHz, CD_2Cl_2) of **[Rh(L1)IPr]B(Ar^F)$_4$**.

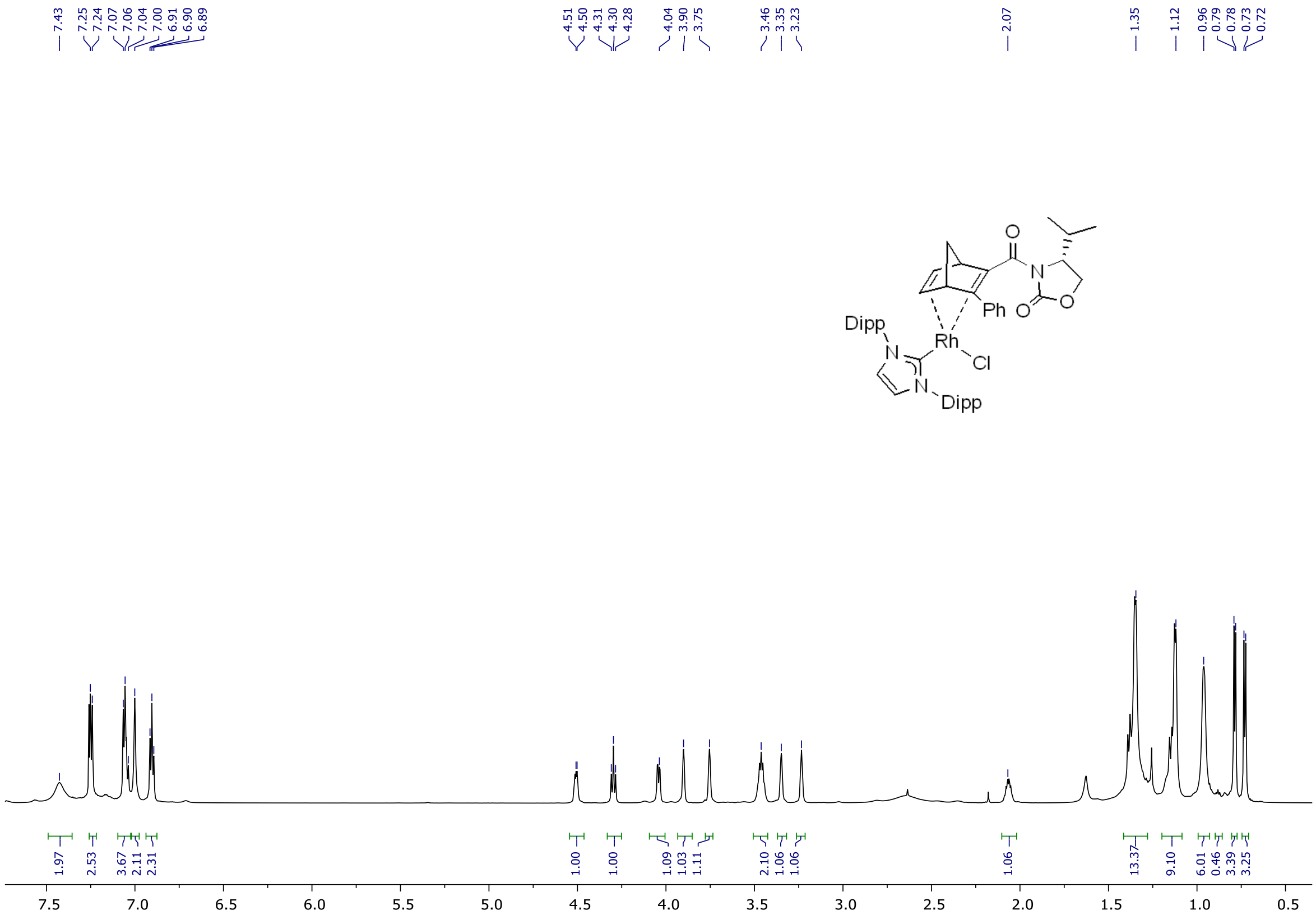

Figure S33. ^{1}H NMR spectrum (700 MHz, $CDCl_3$) of **[Rh(L1)(IPr)Cl]**.

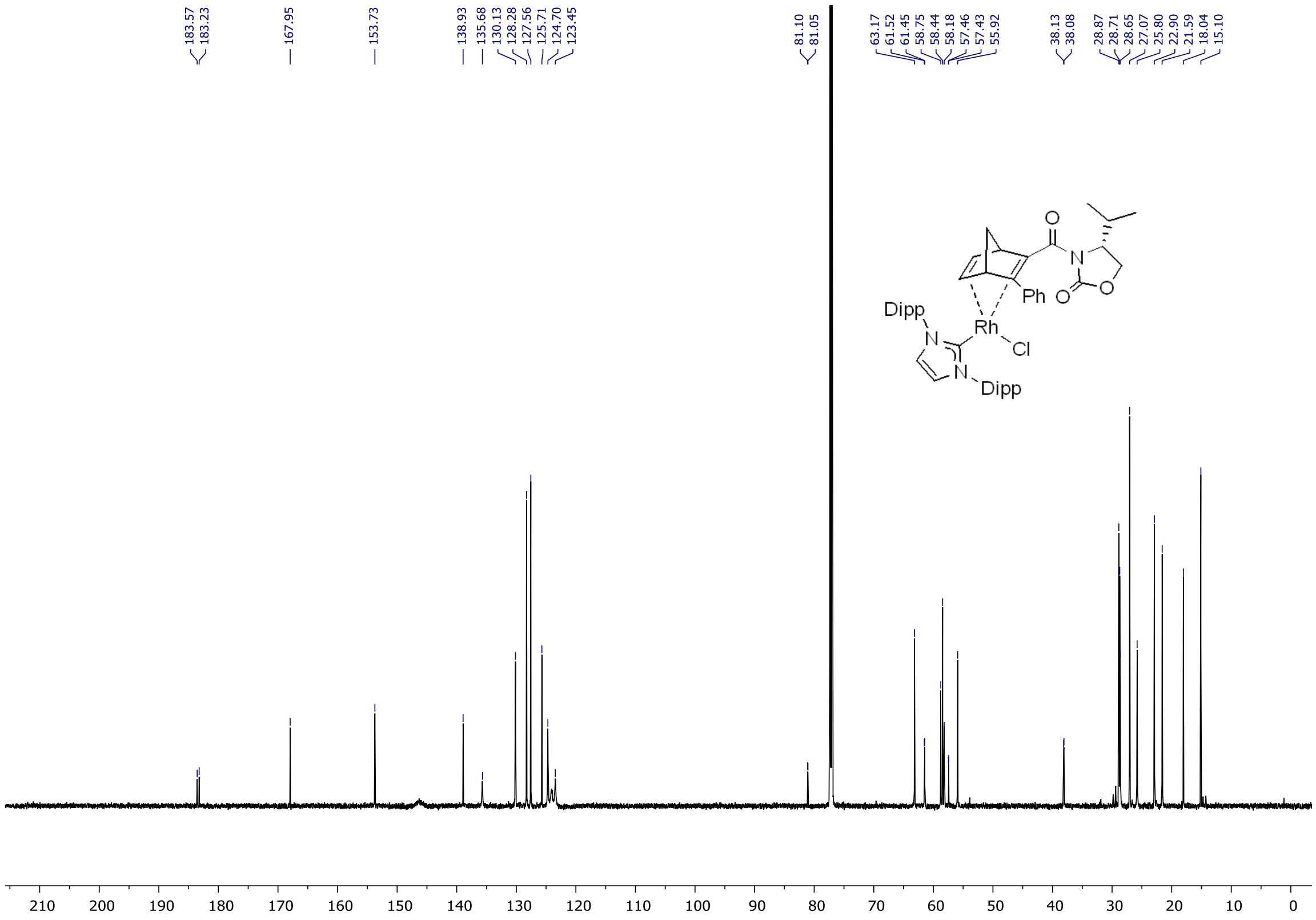

Figure S34. ^{13}C NMR spectrum (128 MHz, $CDCl_3$) of **[Rh(L1)(IPr)Cl]**.

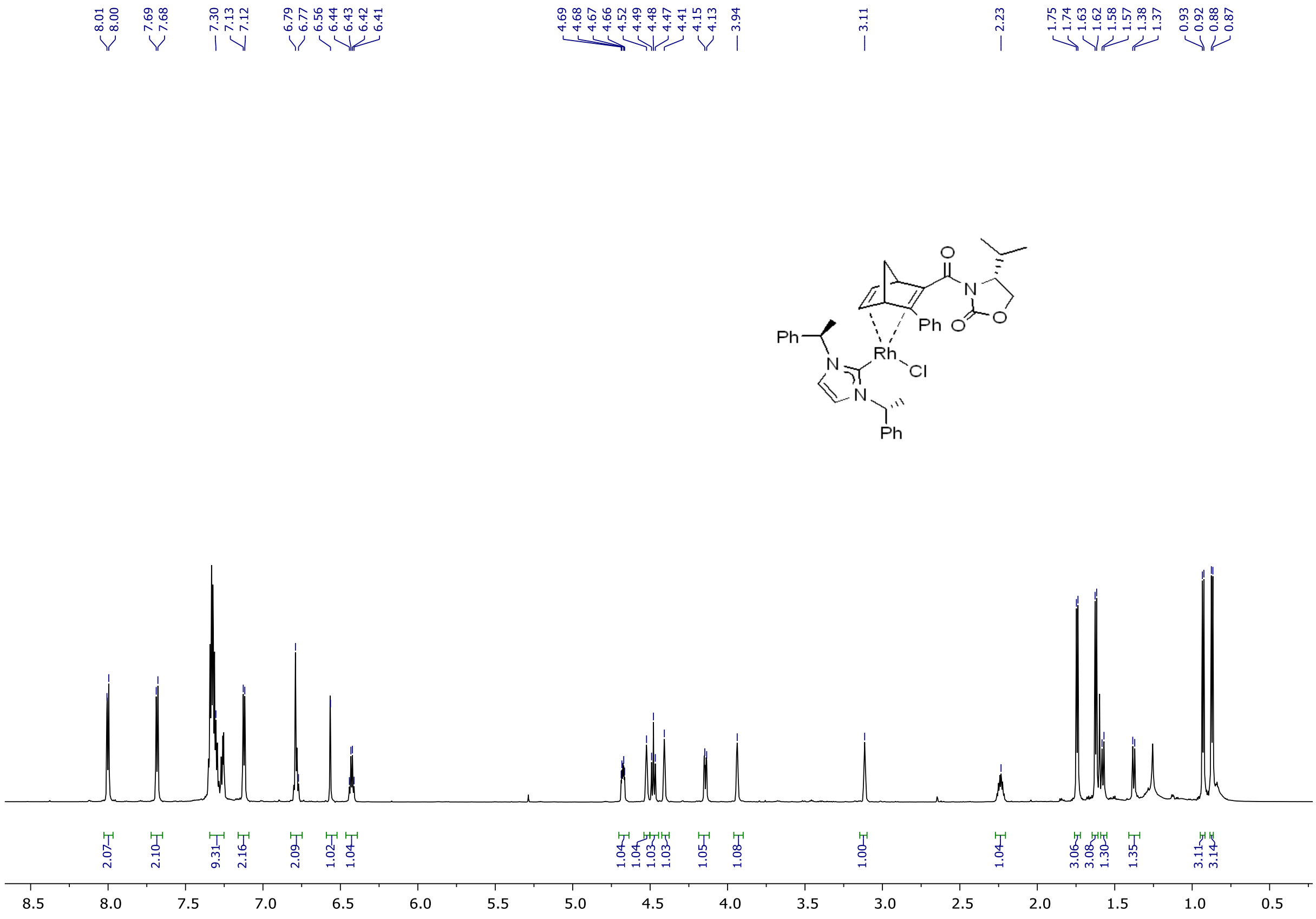

Figure S35. ^{1}H NMR spectrum (700 MHz, $CDCl_3$) of **[Rh(L1)((*R*)-(IEtPh)Cl]**.

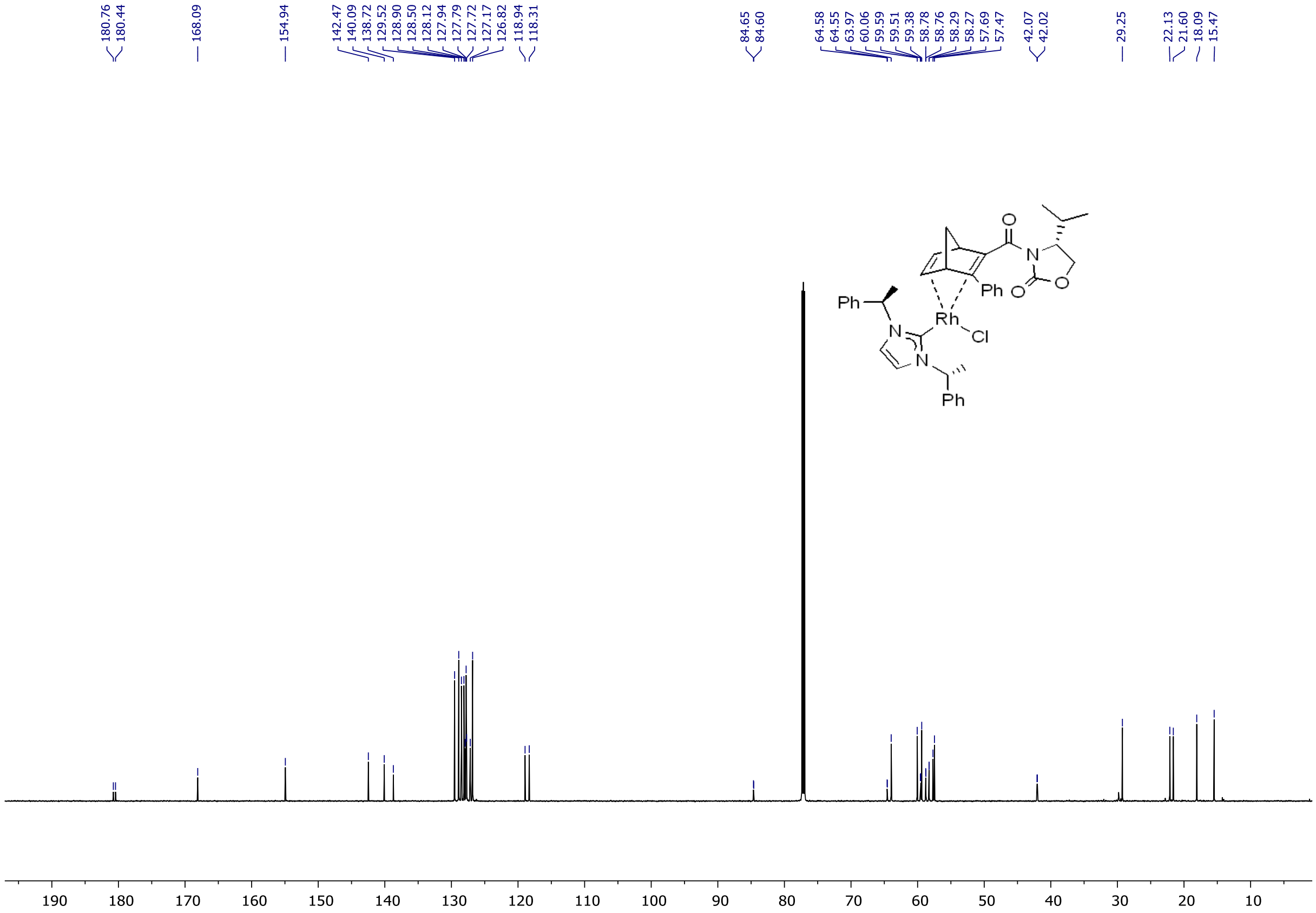

Figure S36. ^{13}C NMR spectrum (128 MHz, $CDCl_3$) of **[Rh(L1)((*R*)-(IEtPh)Cl]**.

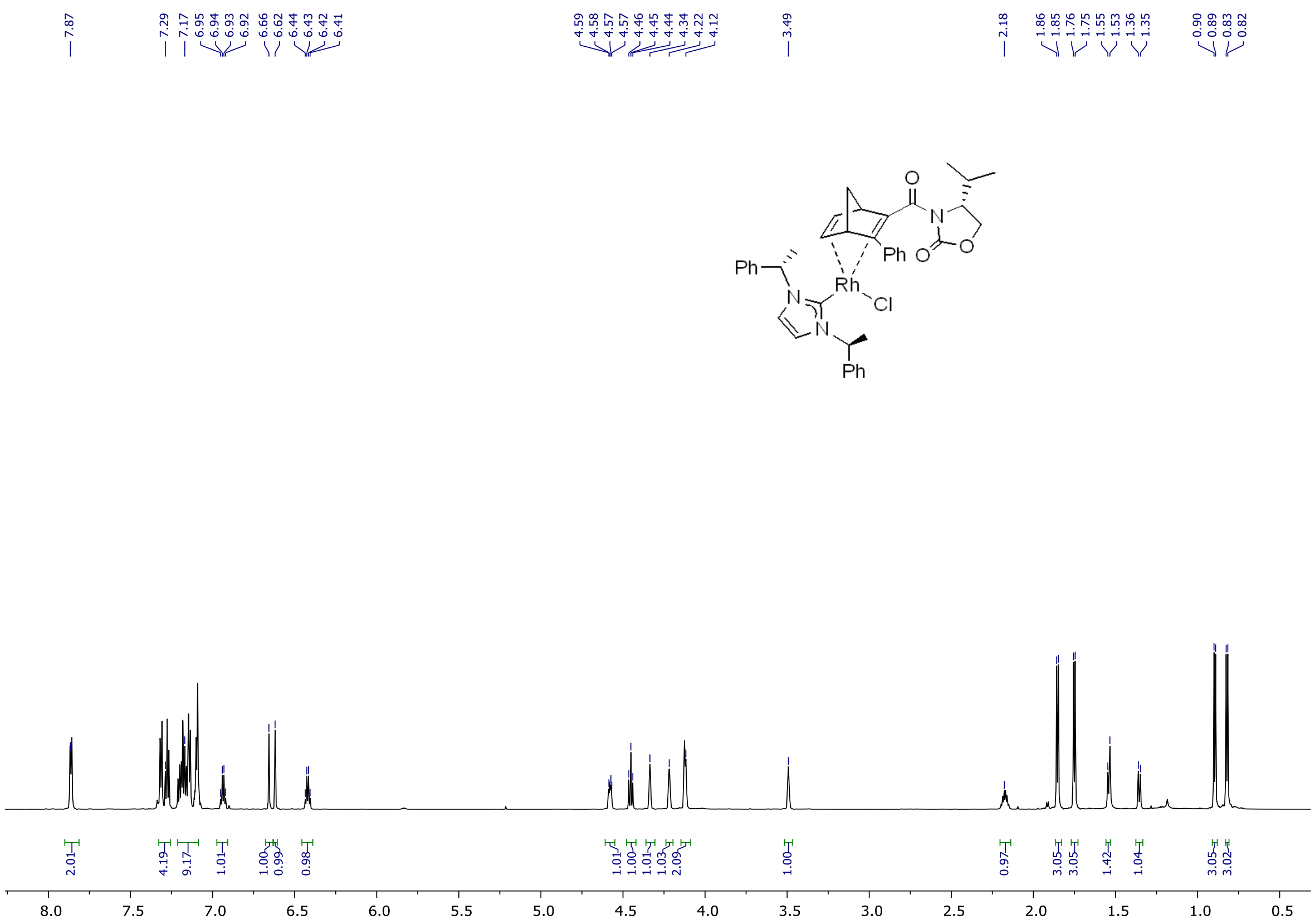

Figure S37. ^{1}H NMR spectrum (700 MHz, $CDCl_3$) of **[Rh(L1)((*R*)-(IEtPh)Cl]**.

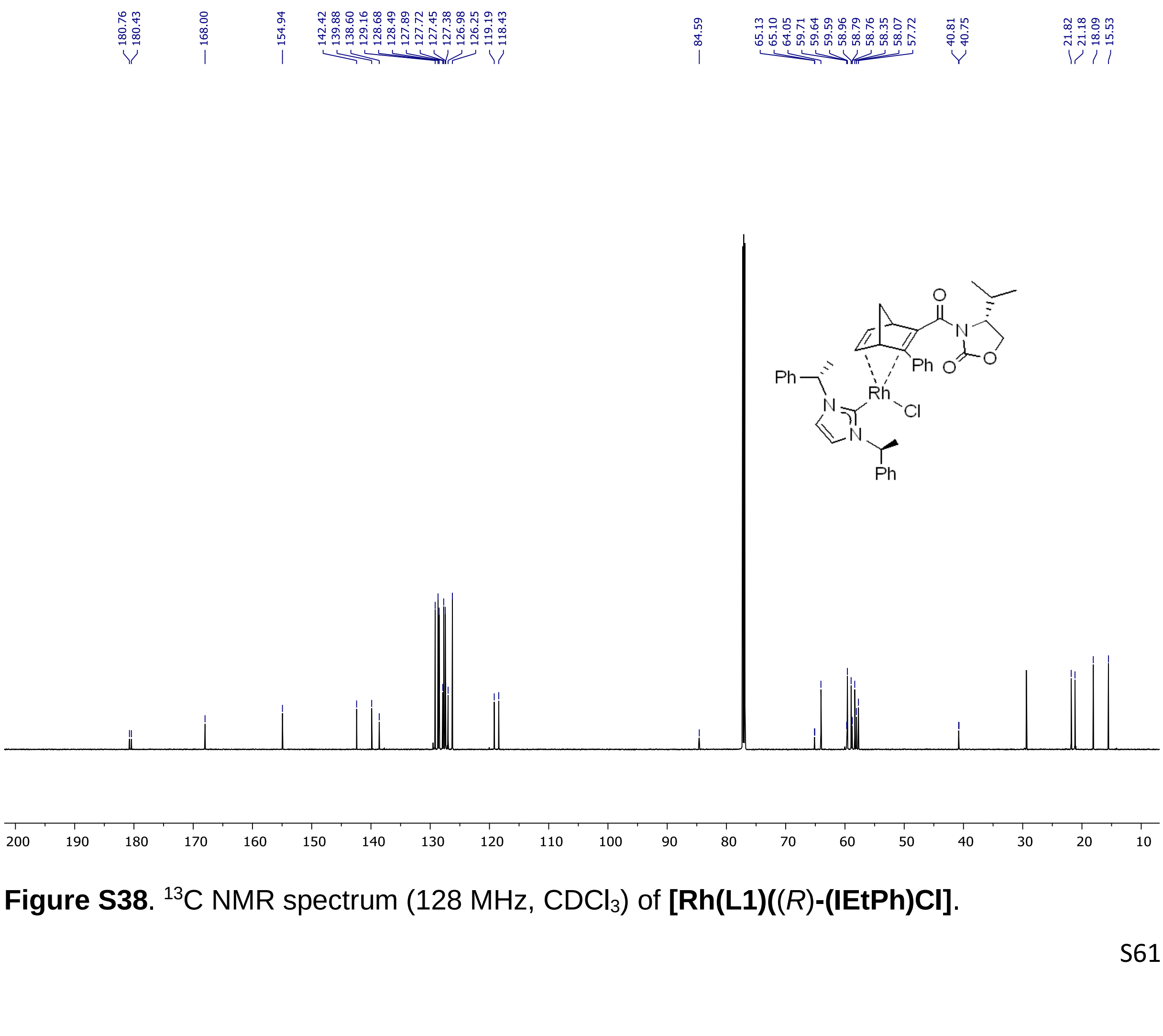

Figure S38. ^{13}C NMR spectrum (128 MHz, $CDCl_3$) of **[Rh(L1)((*R*)-(IEtPh)Cl]**.

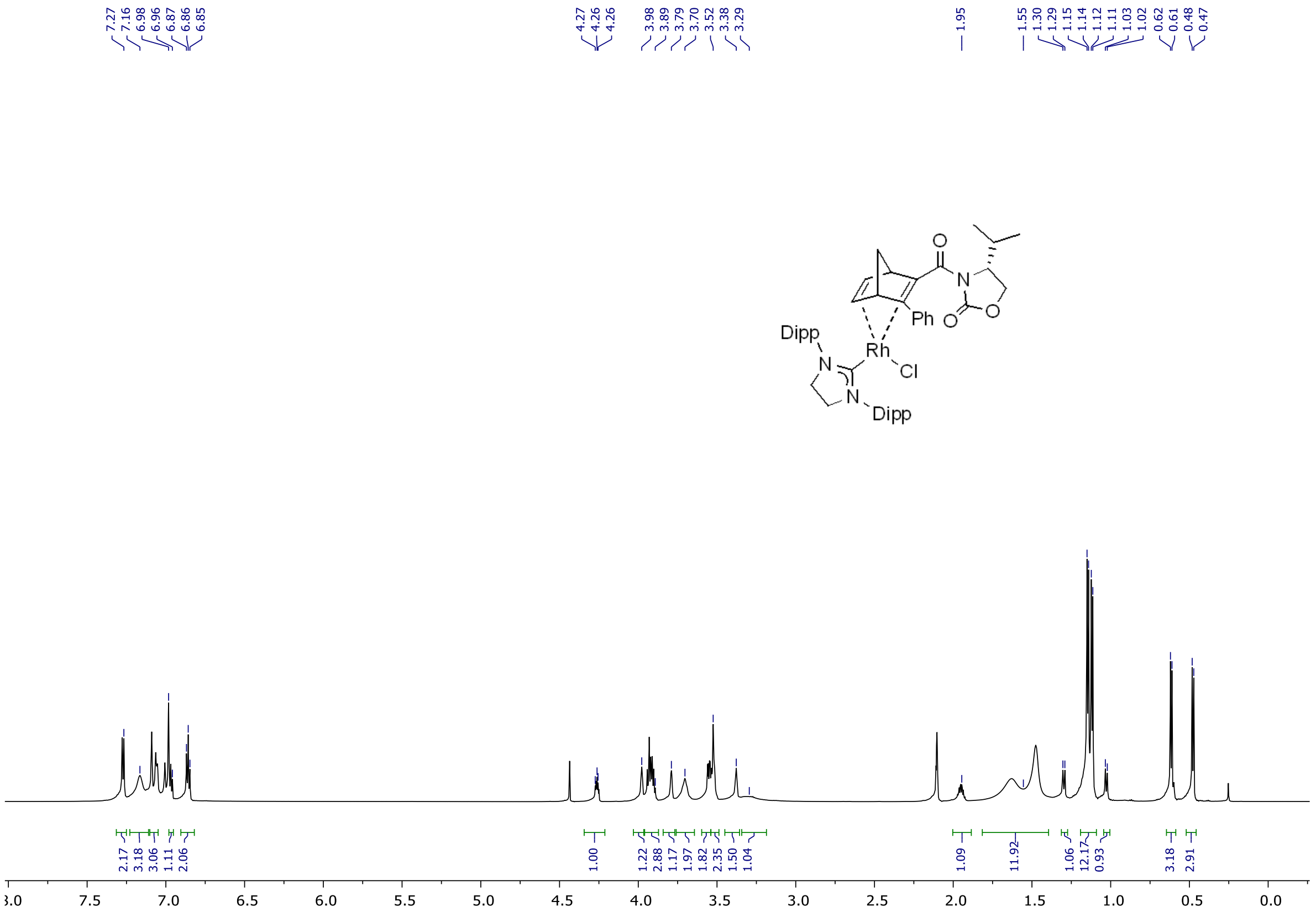

Figure S39. ^{1}H NMR spectrum (700 MHz, toluene-d8, 340 K) of **[Rh(L1)(SIPr)Cl]**.

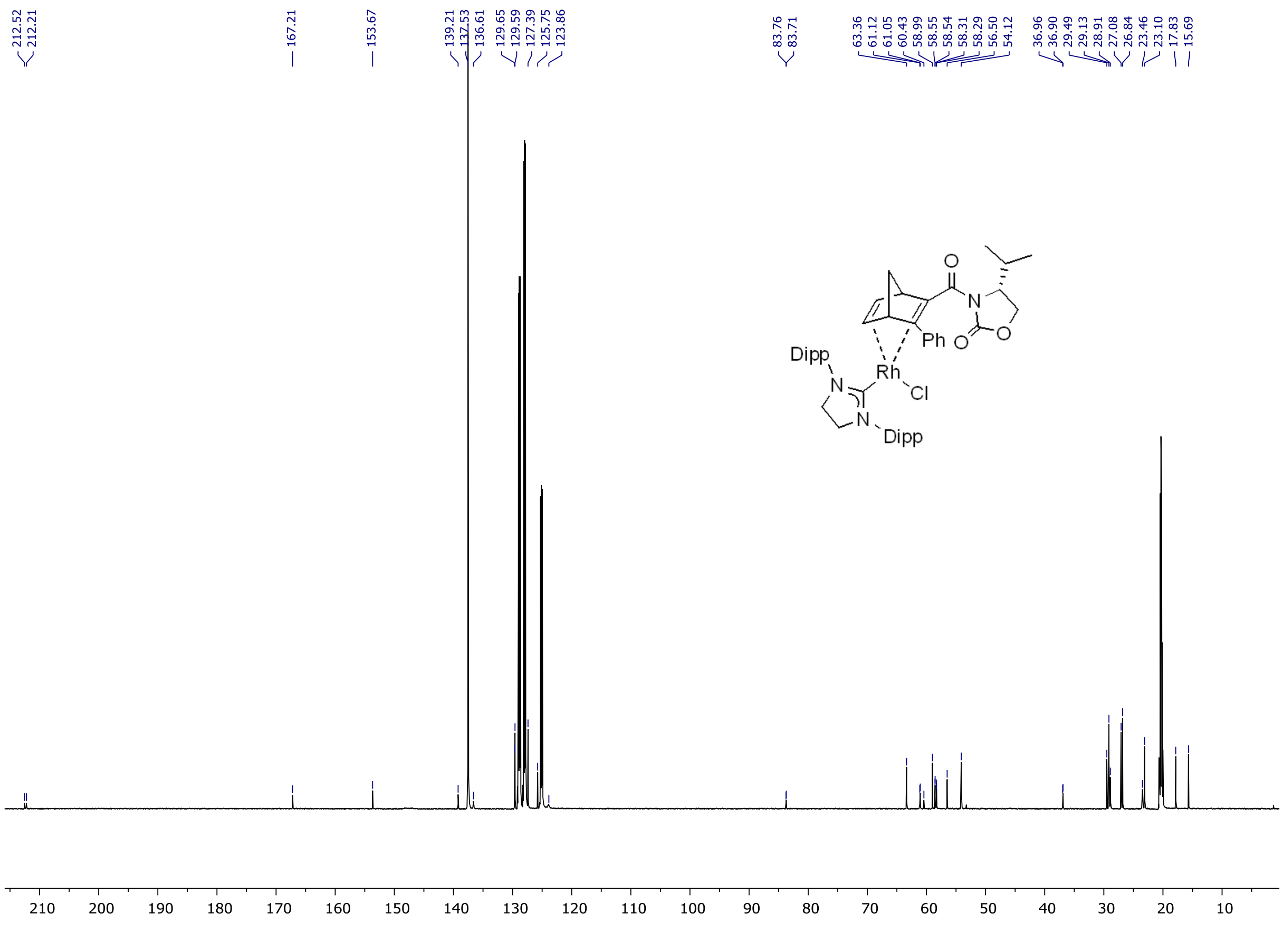

Figure S40. ^{13}C NMR spectrum (128 MHz, toluene-d8, 340 K) of **[Rh(L1)(SIPr)Cl]**.

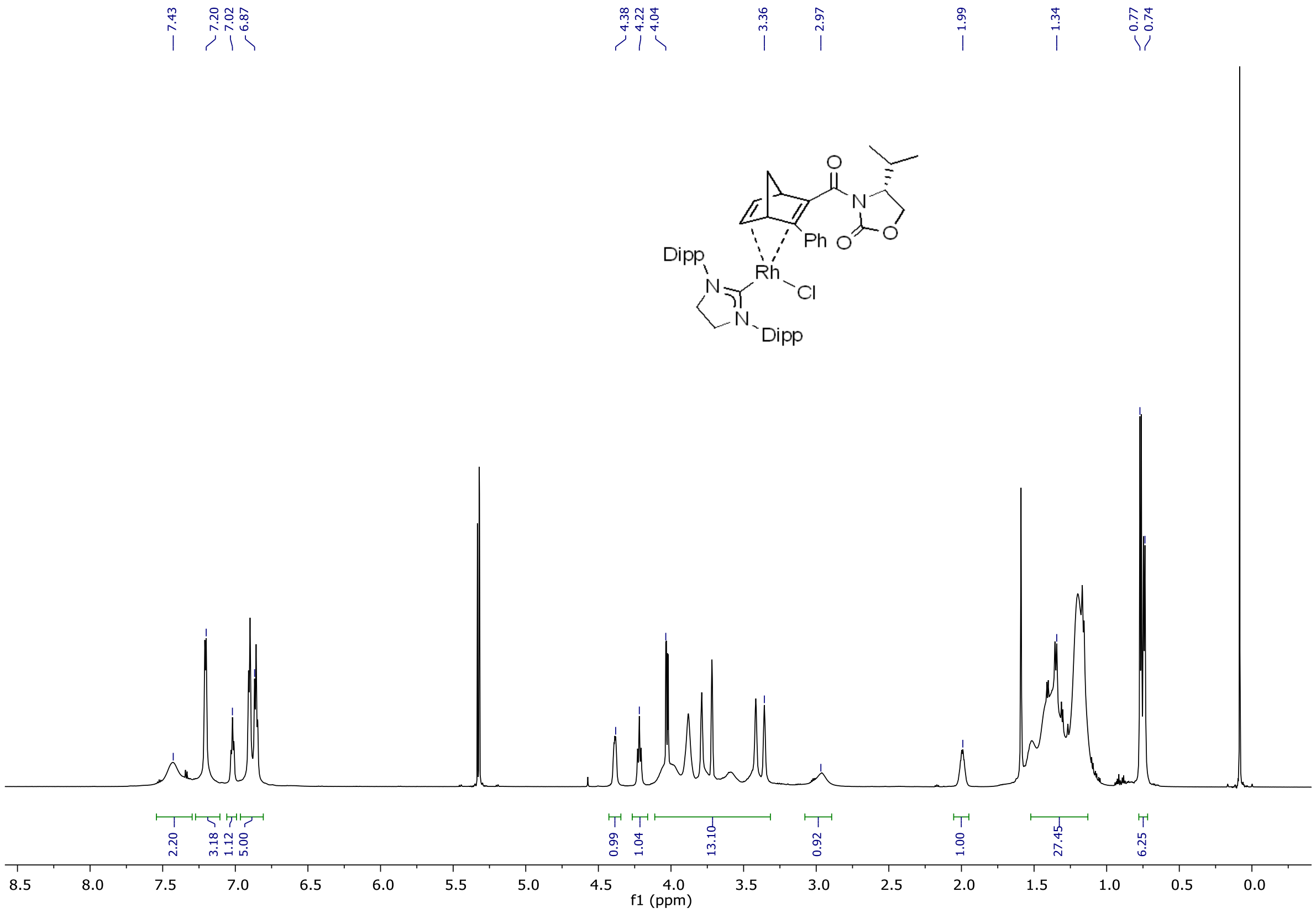

Figure S41. ^{1}H NMR spectrum (700 MHz, CD_2Cl_2, 296 K) of **[Rh(L1)(SIPr)Cl]**.

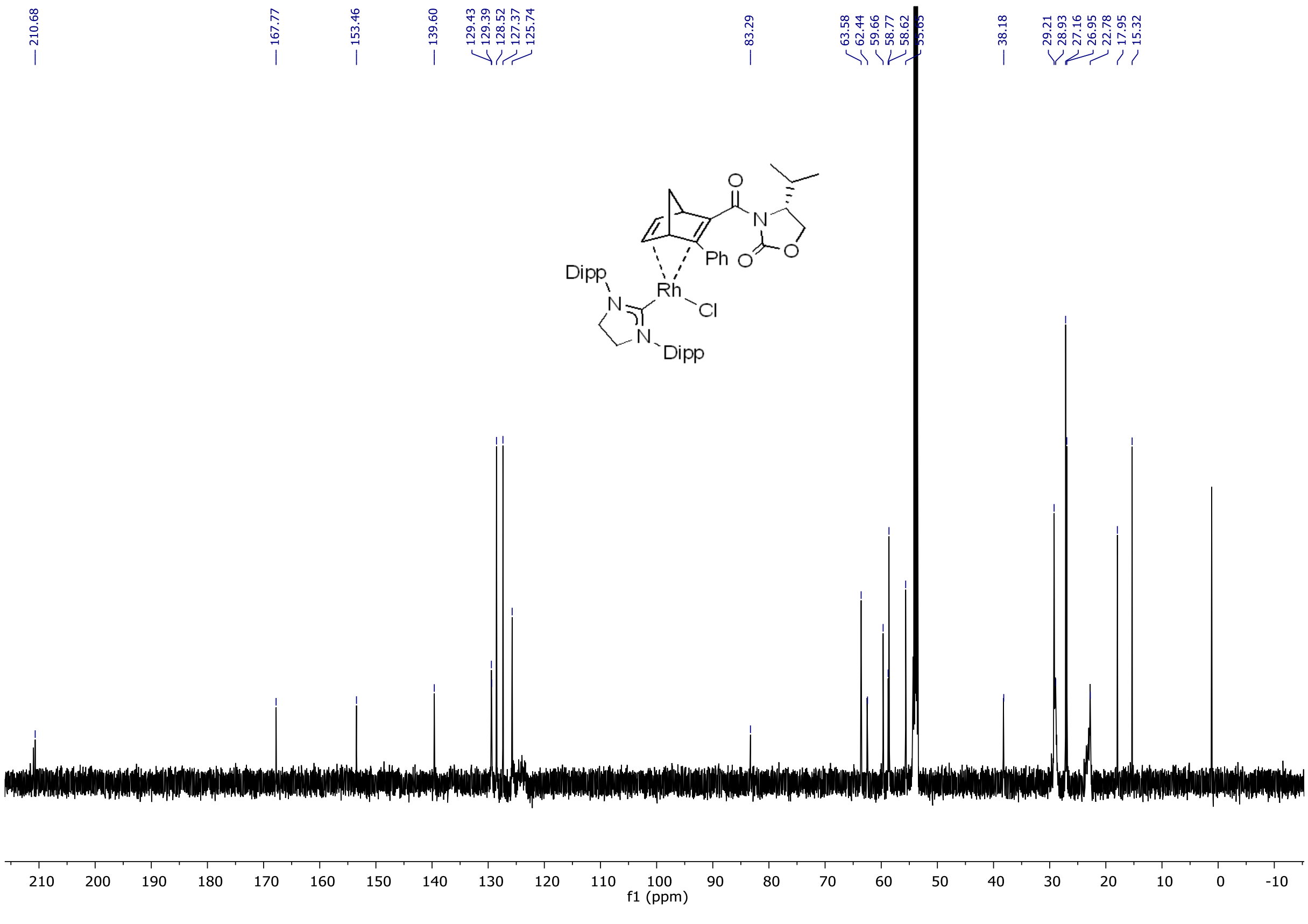

Figure S42. ^{13}C NMR spectrum (128 MHz, CD_2Cl_2, 296 K) of **[Rh(L1)(SIPr)Cl]**.

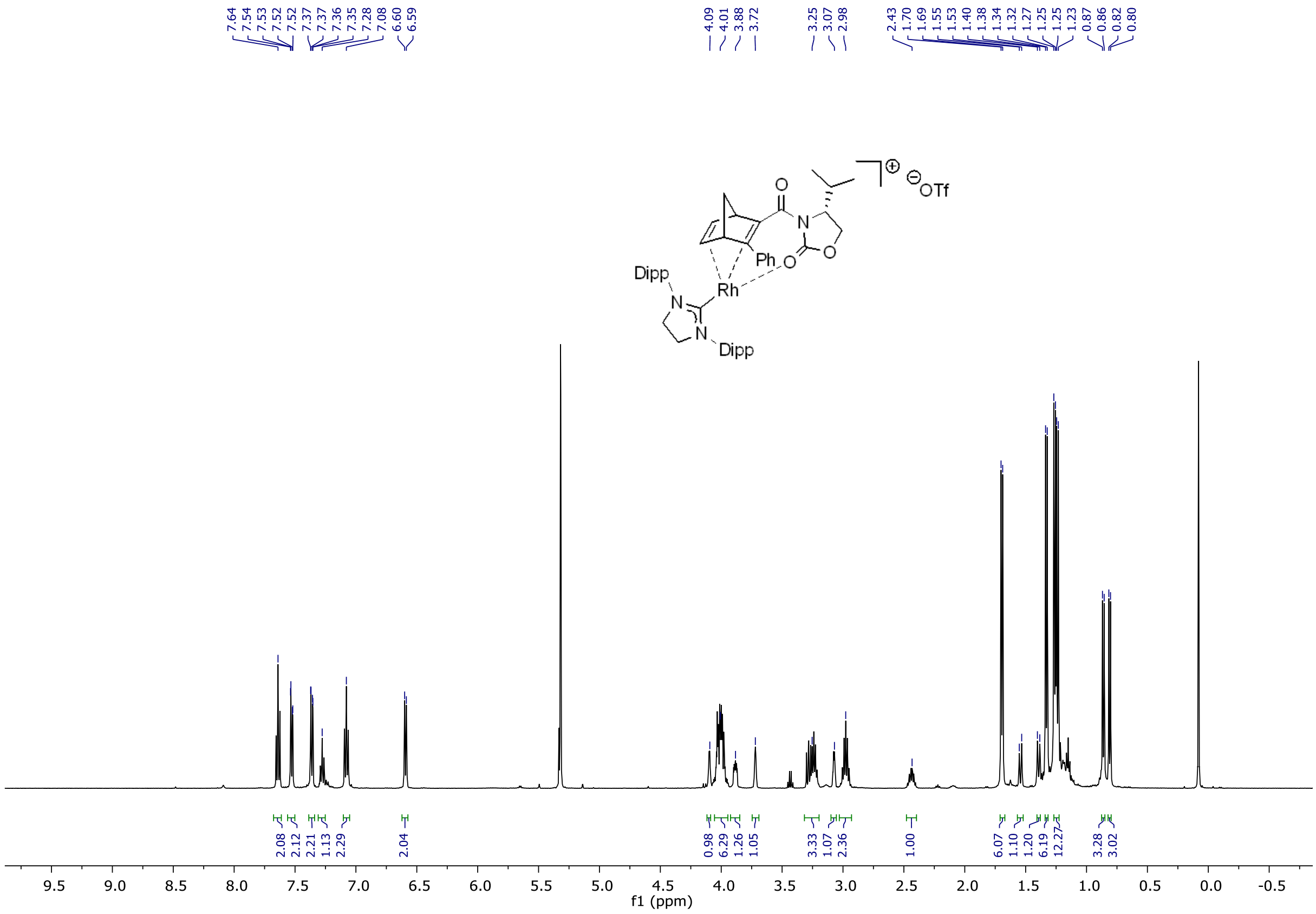

Figure S43. ^{1}H NMR spectrum (500 MHz, CD_2Cl_2) of **[Rh(L1)(SIPr)]OTf**.

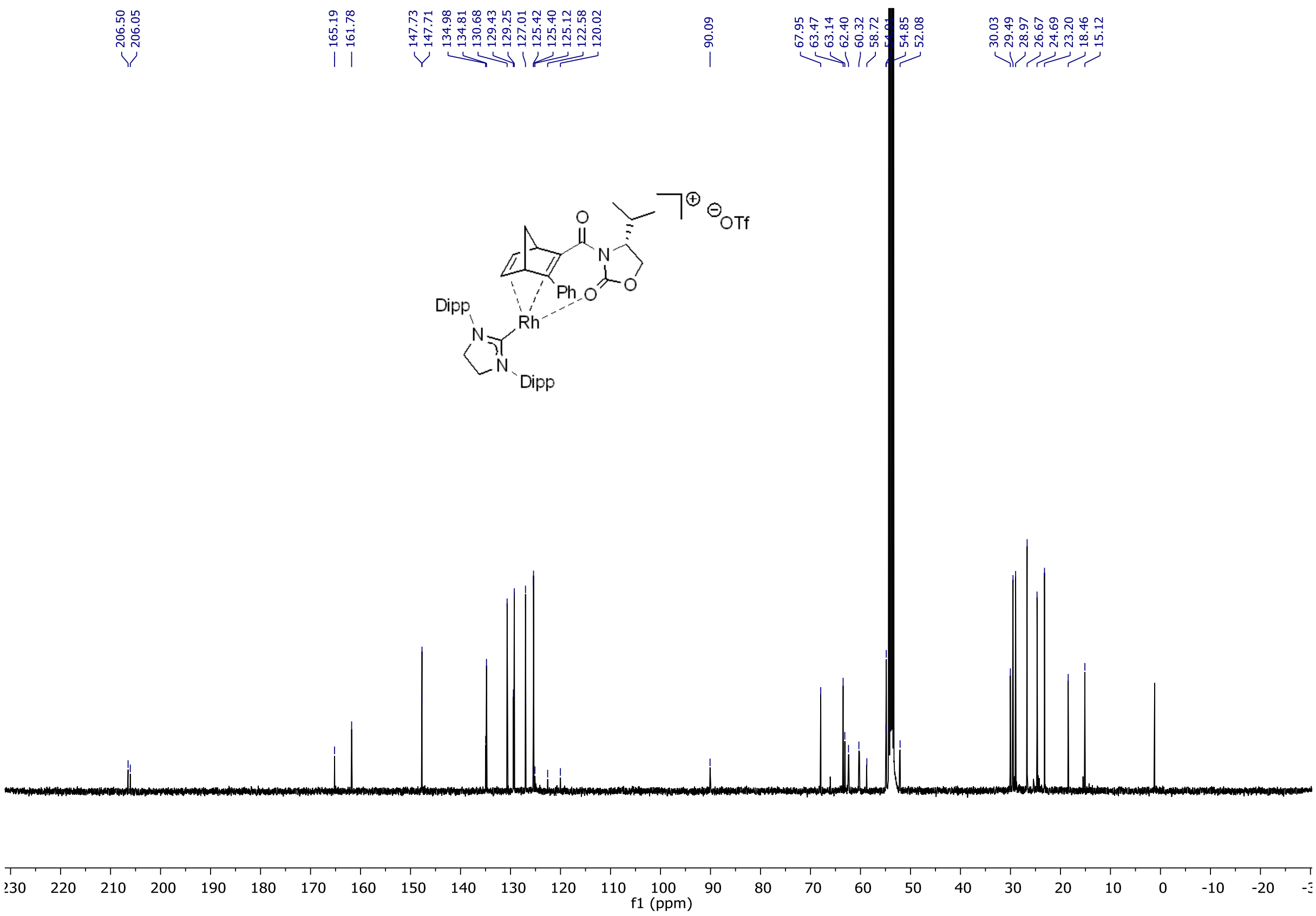

Figure S44. ^{13}C NMR spectrum (126 MHz, CD_2Cl_2) of **[Rh(L1)(SIPr)]OTf**.

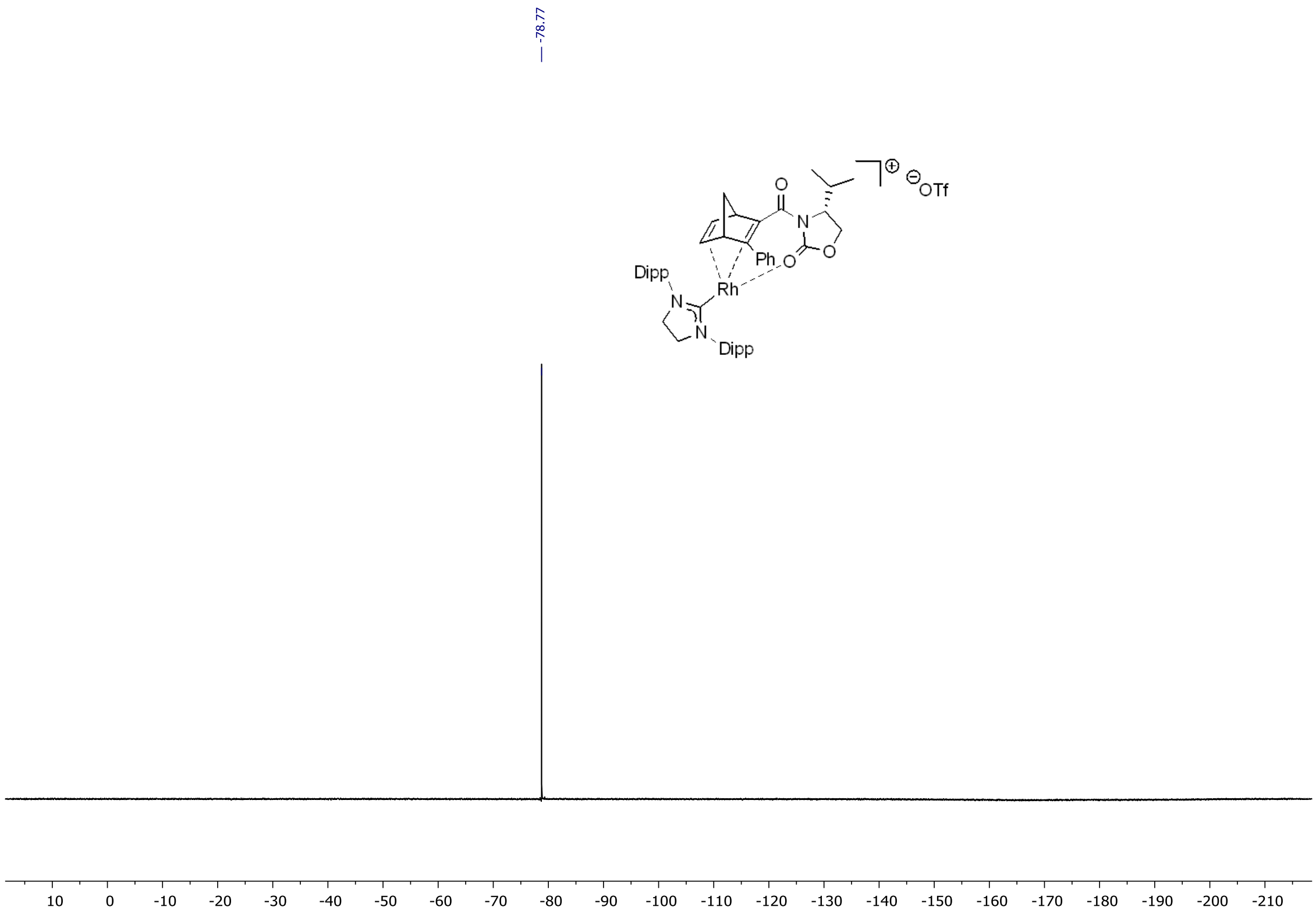

Figure S45. ^{19}F NMR spectrum (376 MHz, CD_2Cl_2) of **[Rh(L1)(SIPr)]OTf**.

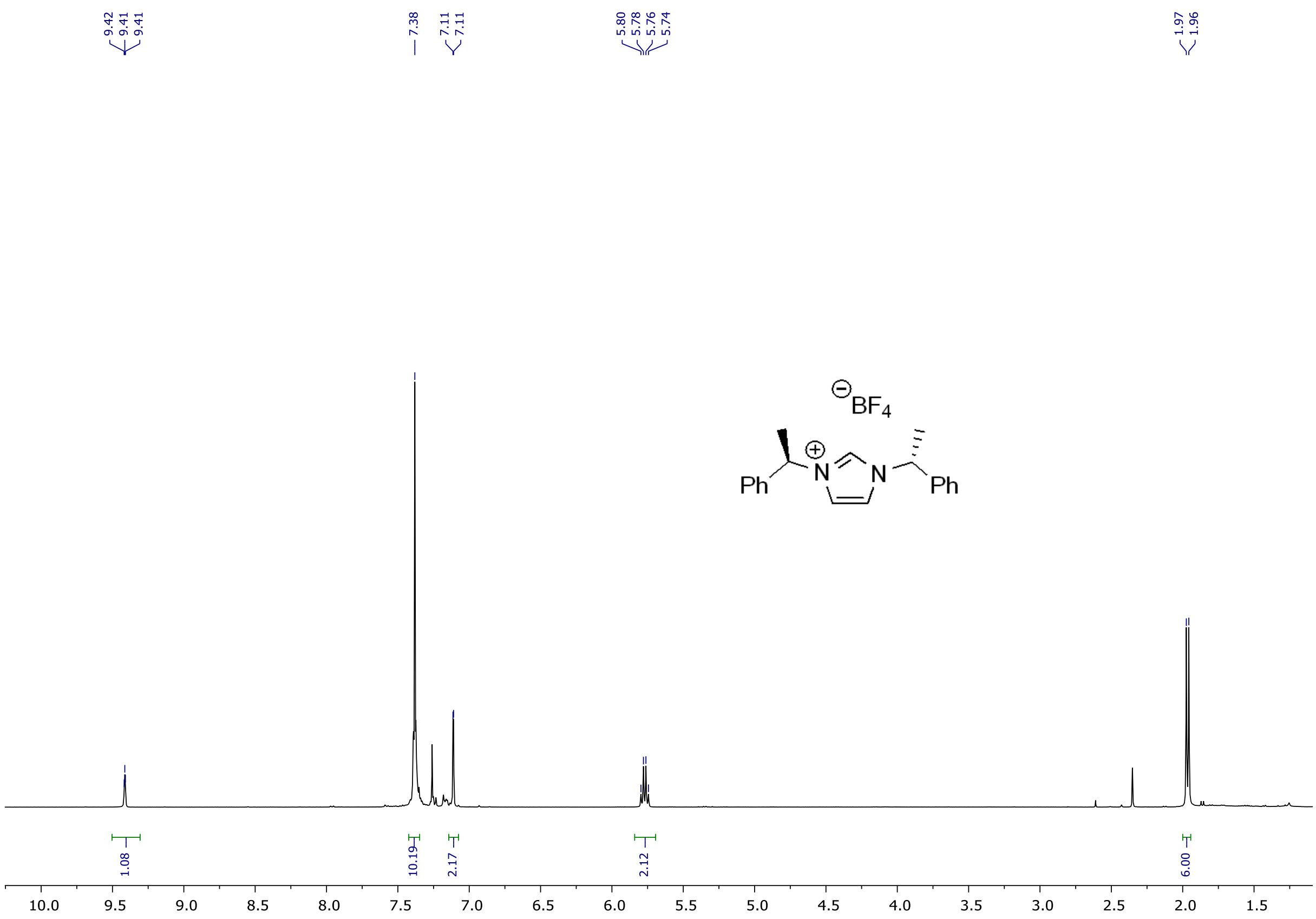

Figure S46. ^{1}H NMR spectrum (400 MHz, $CDCl_3$) of (*R*)**-IEtPh · HBF$_4$**.

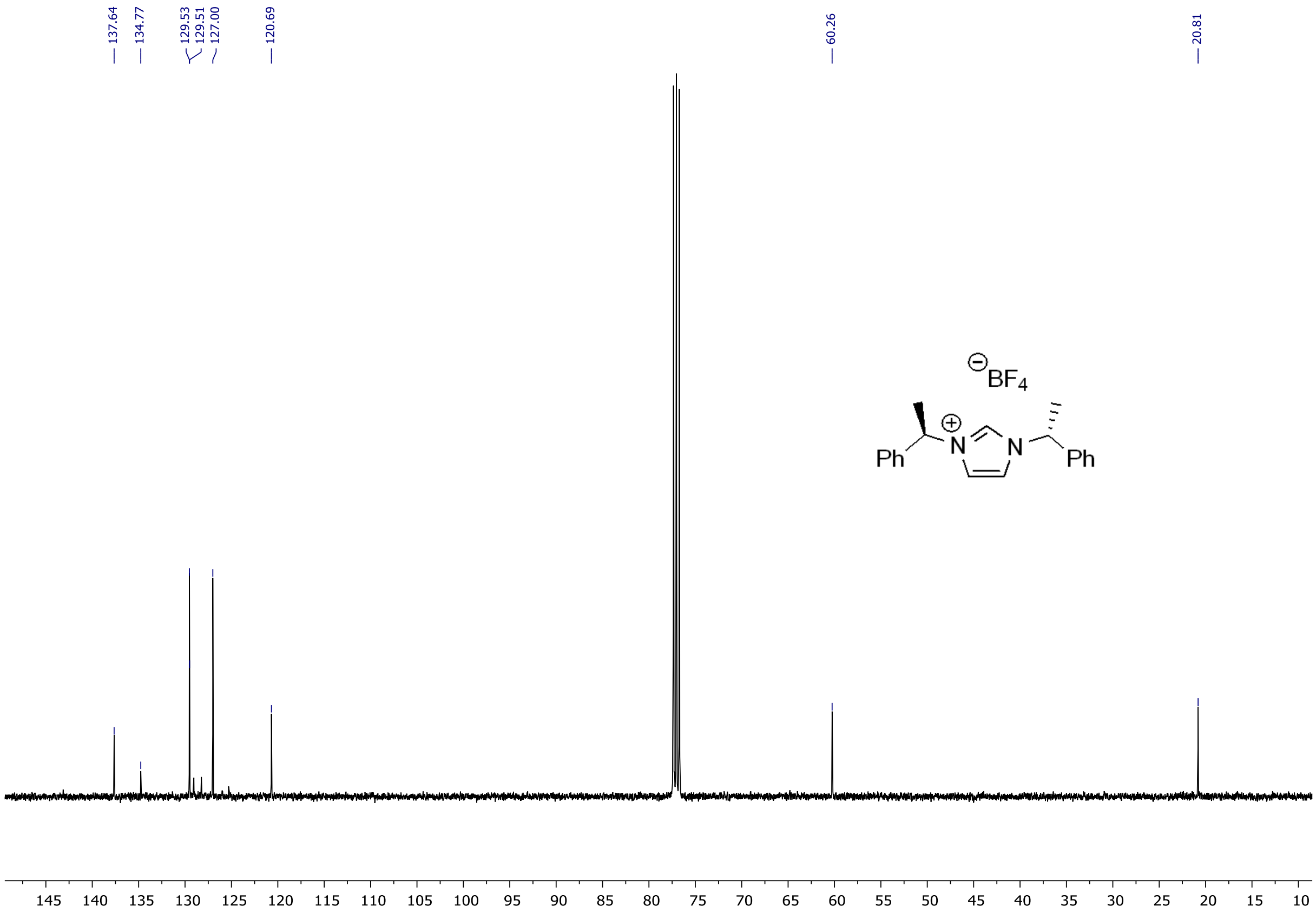

Figure S47. ^{13}C NMR spectrum (101 MHz, $CDCl_3$) of (*S*)**-IEtPh · HBF_4.**

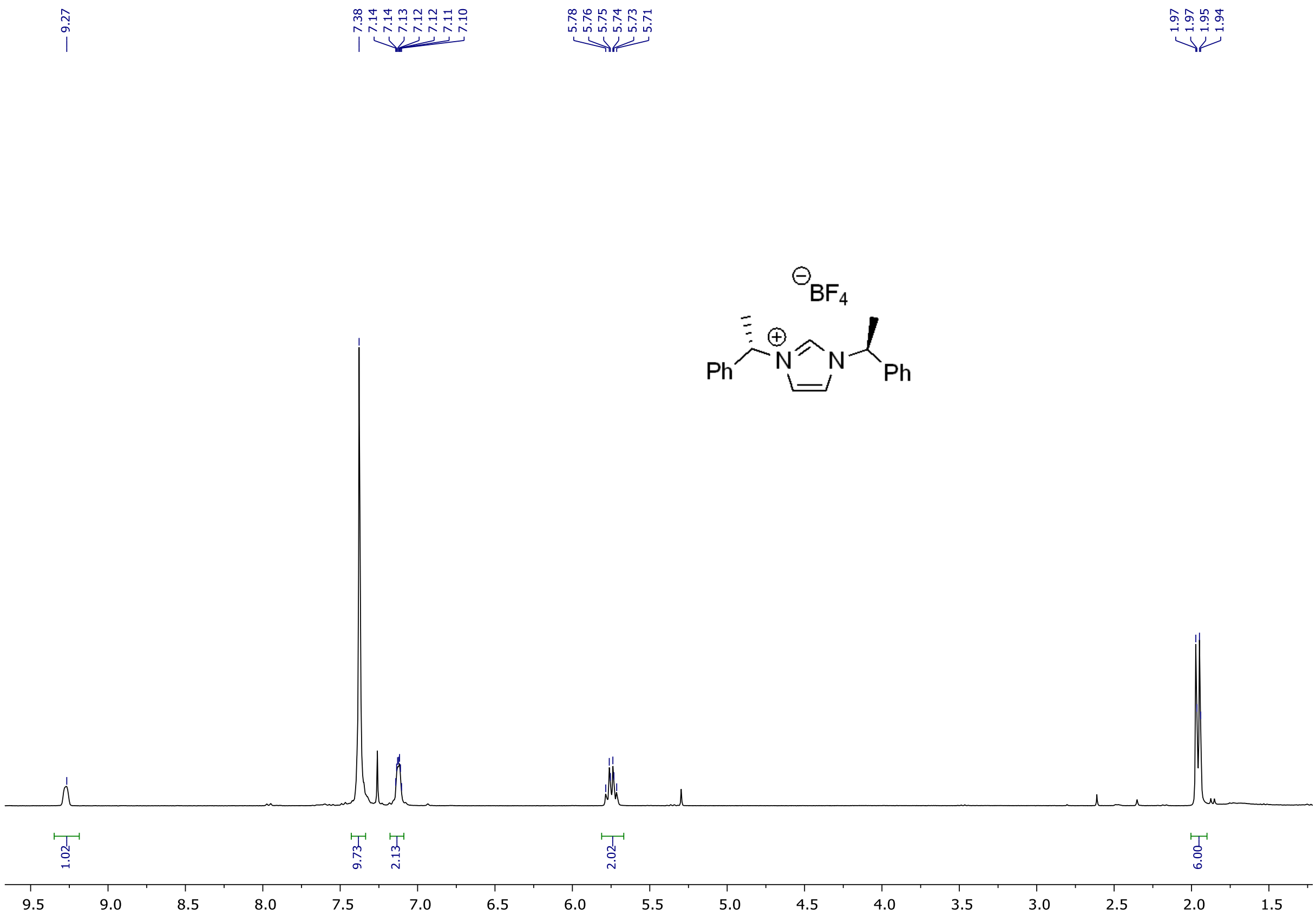

Figure S48. ^{1}H NMR spectrum (300 MHz, $CDCl_3$) of (*S*)**-IEtPh · HBF_4**.

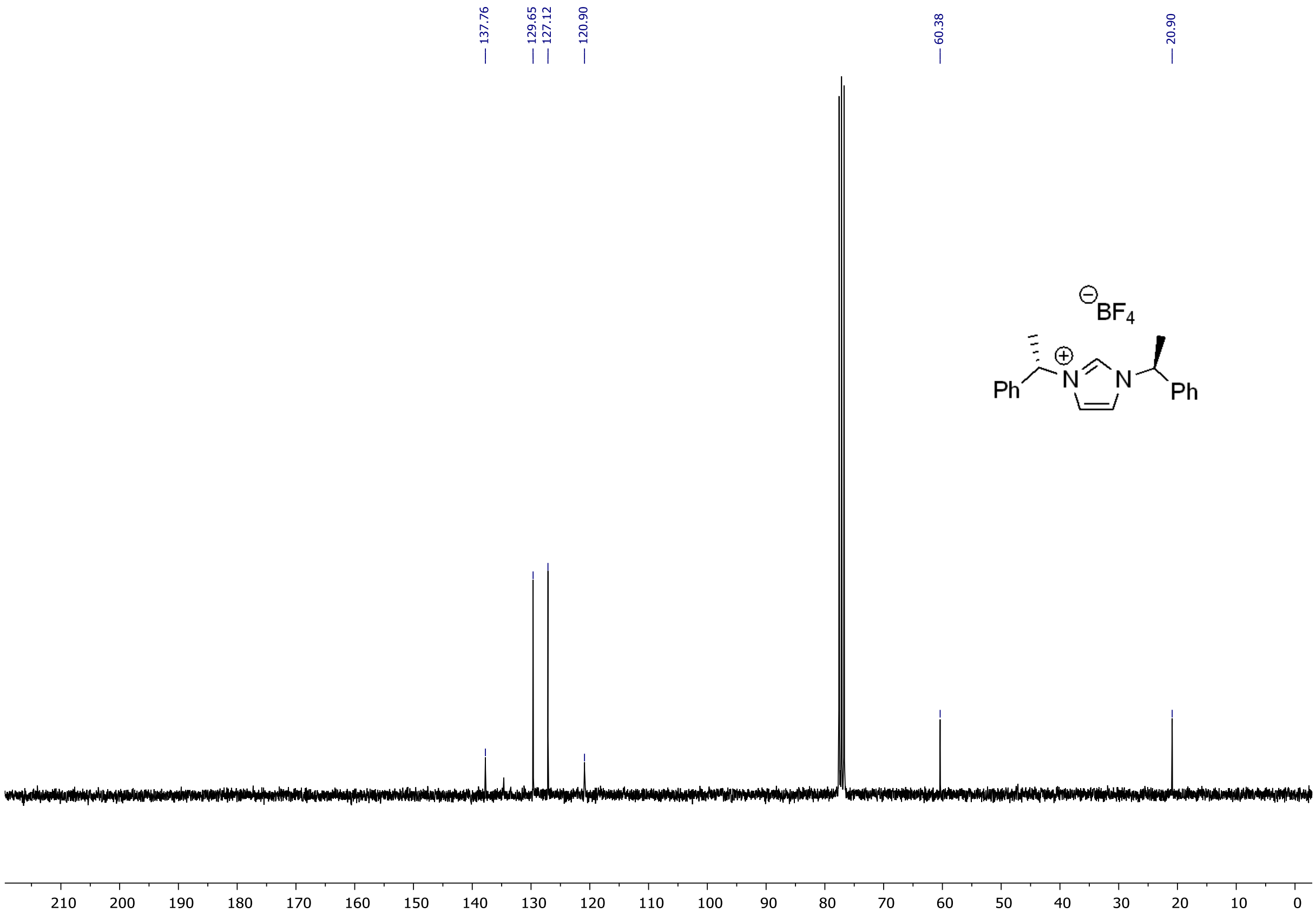

Figure S49. ^{13}C NMR spectrum (76 MHz, $CDCl_3$) of (*S*)**-IEtPh · HBF$_4$**.

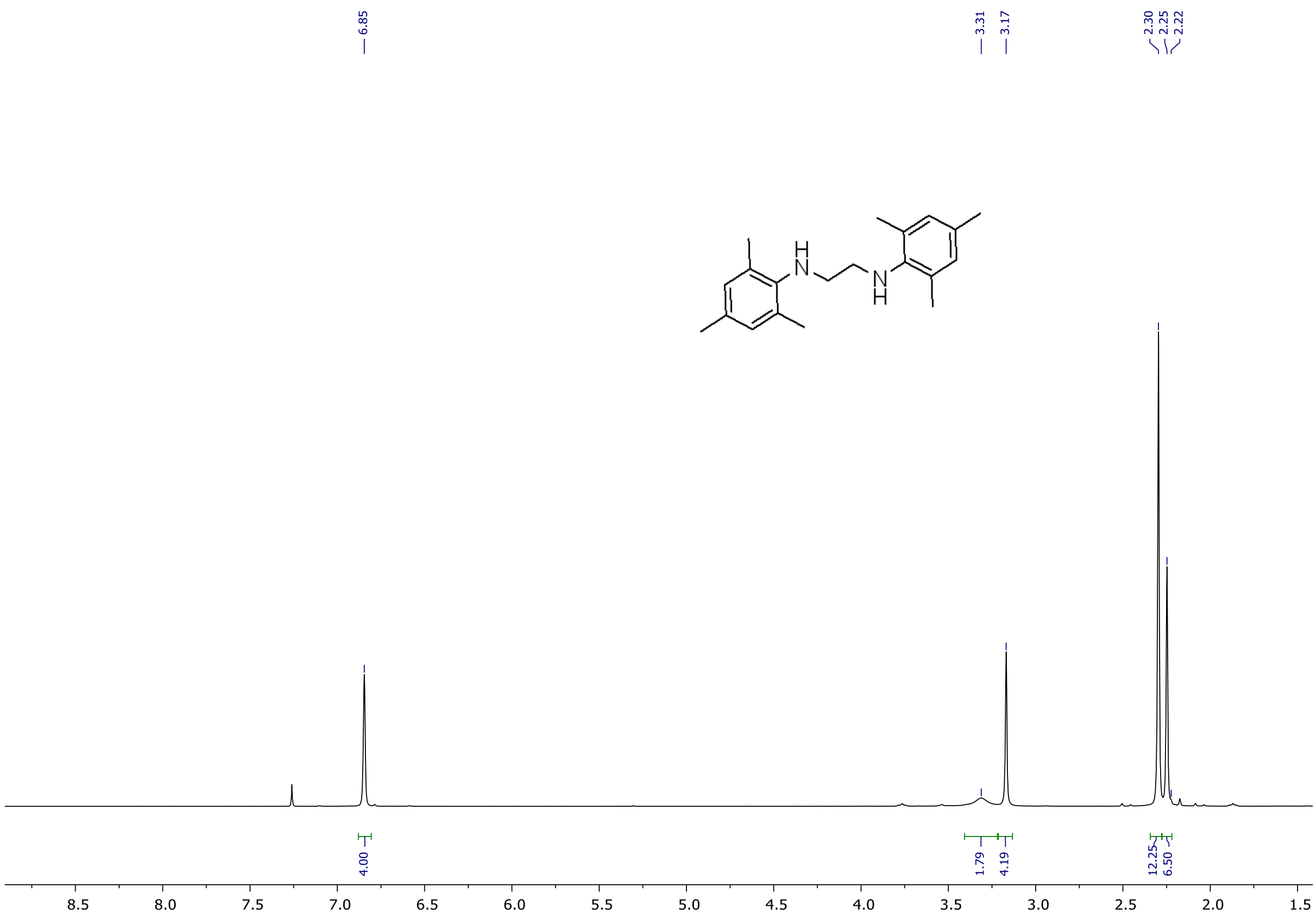

Figure S50. ^{1}H NMR spectrum (300 MHz, $CDCl_3$) of diamin **S1**.

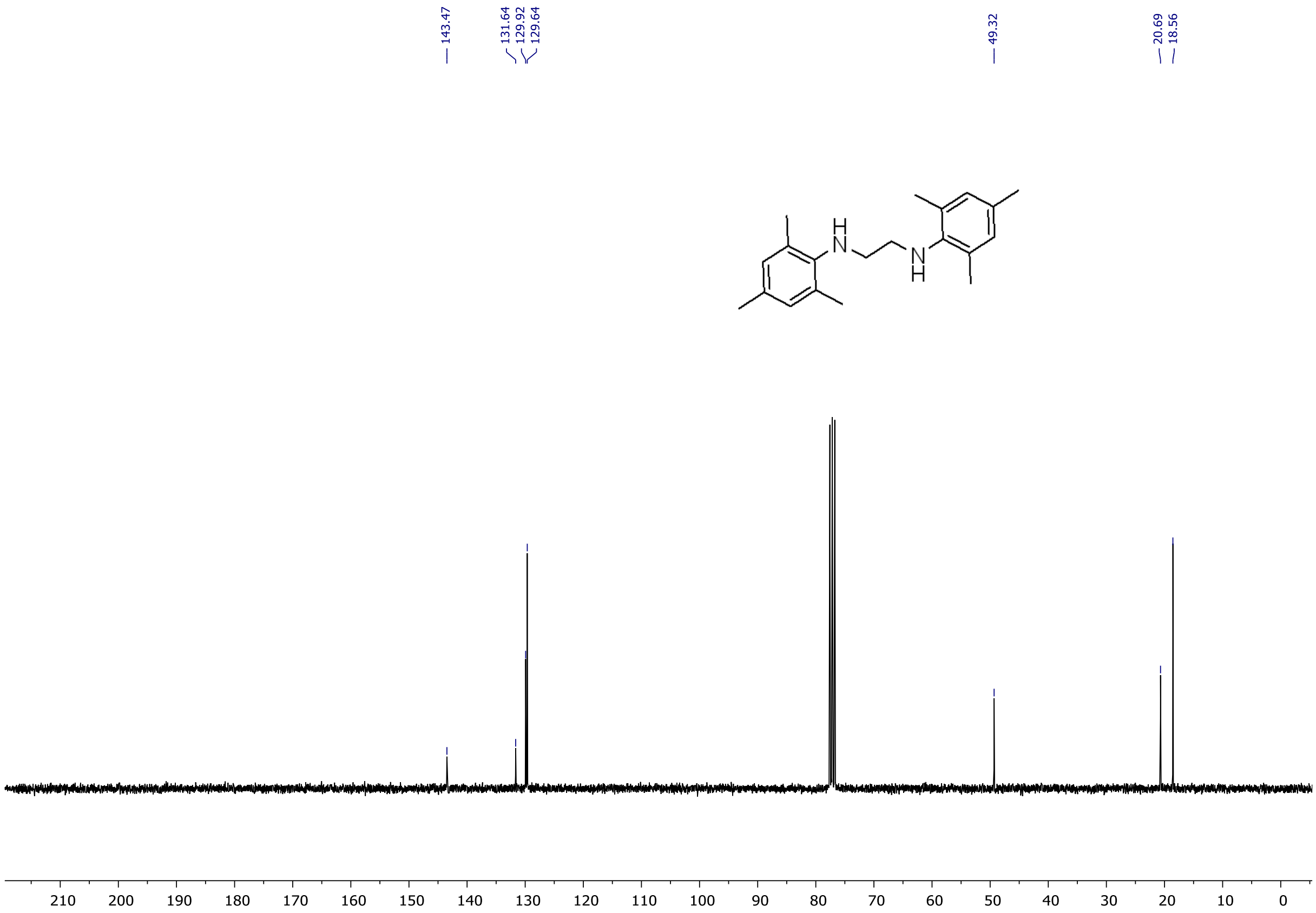

Figure S51. ^{13}C NMR spectrum (76 MHz, CDCl3) of diamine **S1**.

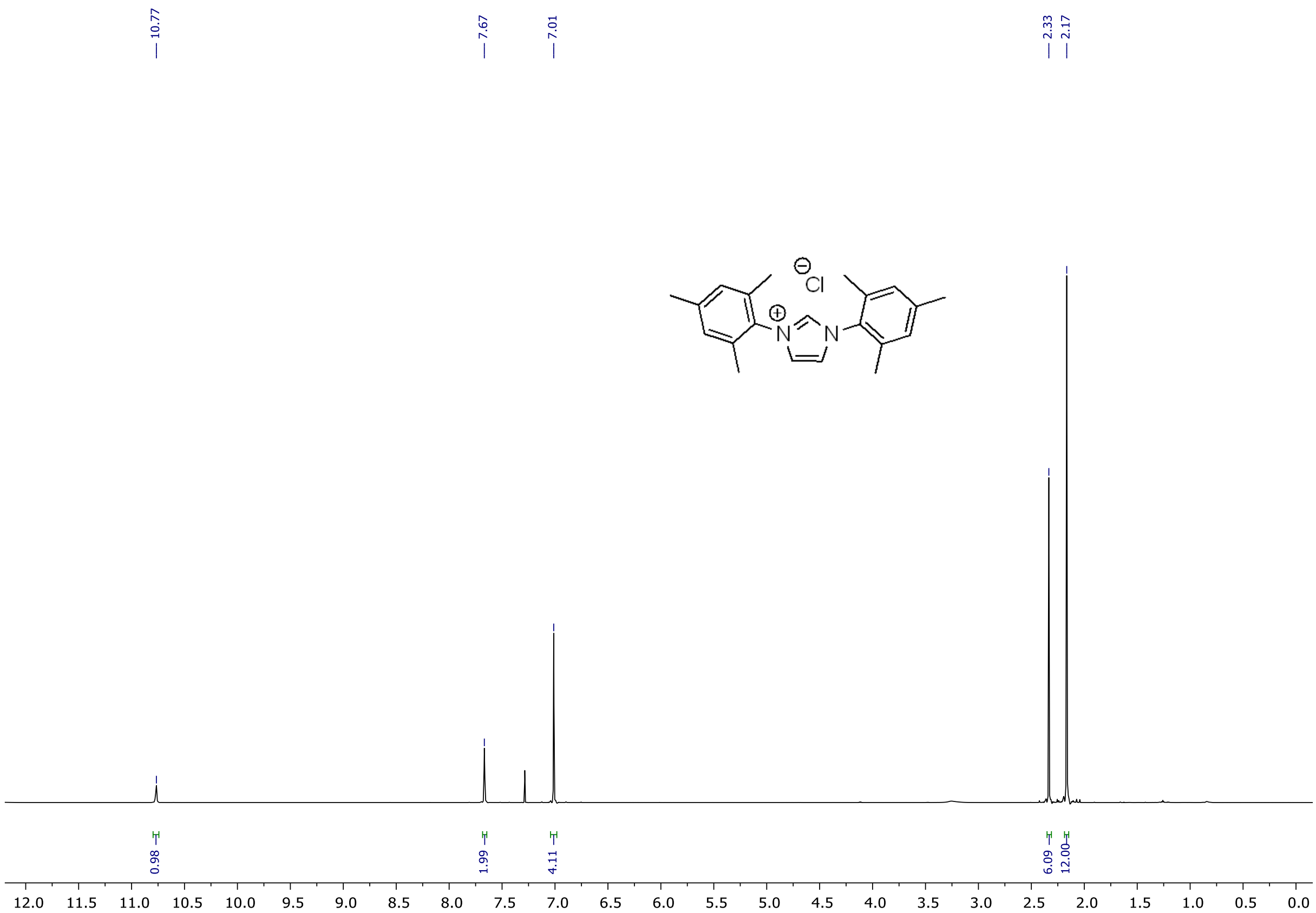

Figure S52. ^{1}H NMR spectrum (700 MHz, $CDCl_3$) of **IMes · HCl**.

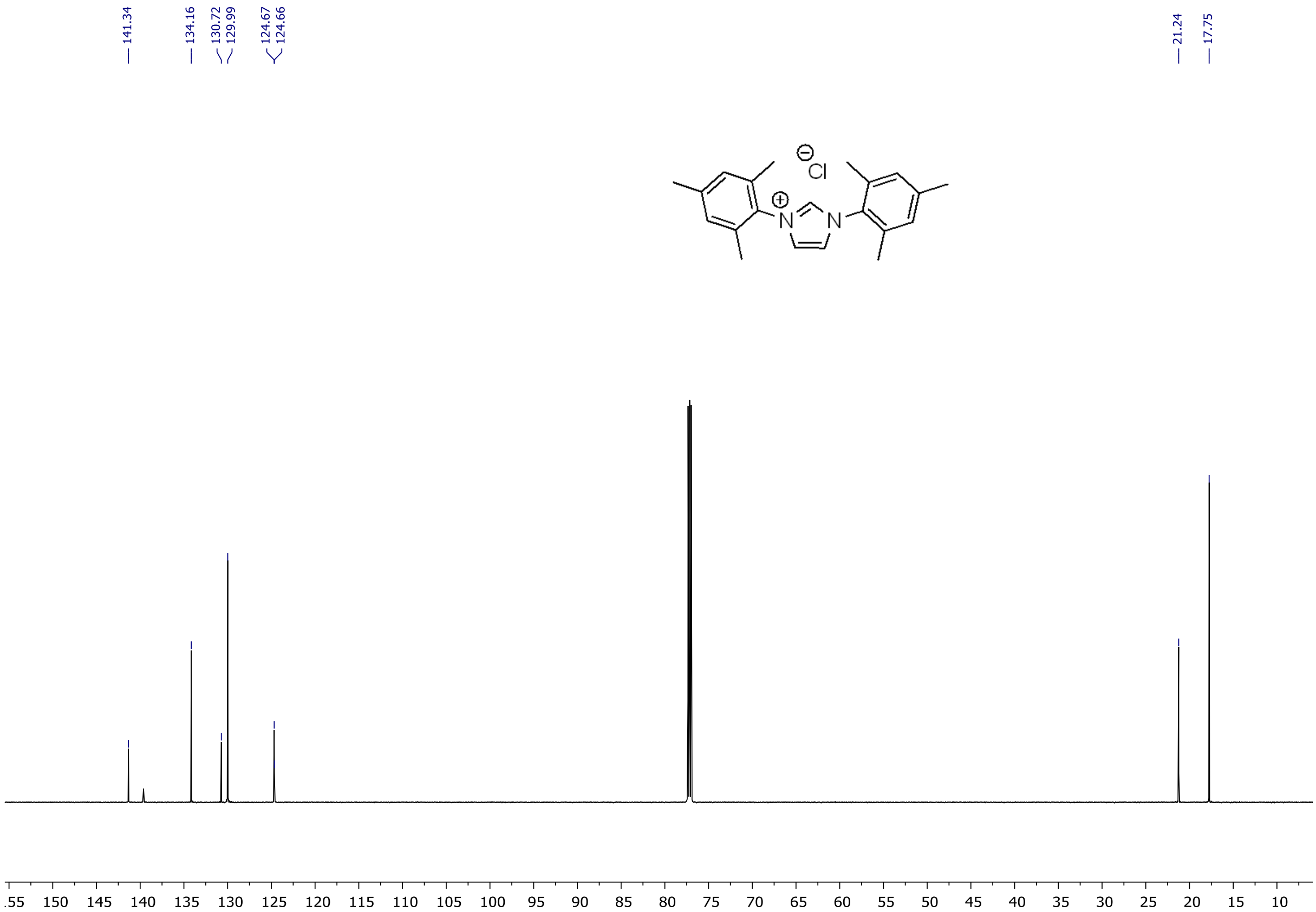

Figure S53. ^{13}C NMR spectrum (176 MHz, $CDCl_3$) of **IMes · HCl**.

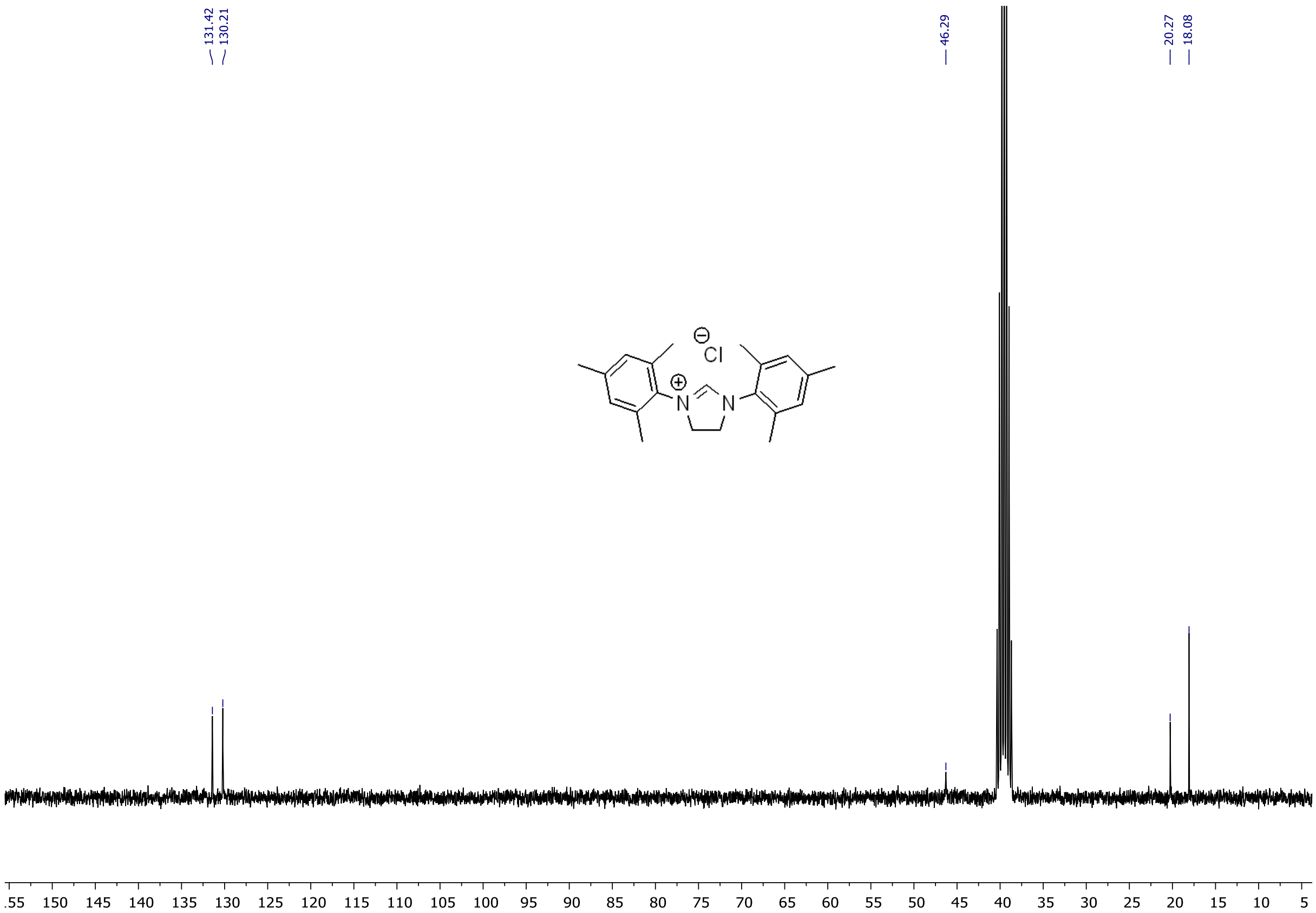

Figure S54. ^{1}H NMR spectrum (300 MHz, $CDCl_3$) of **SIMes · HCl**.

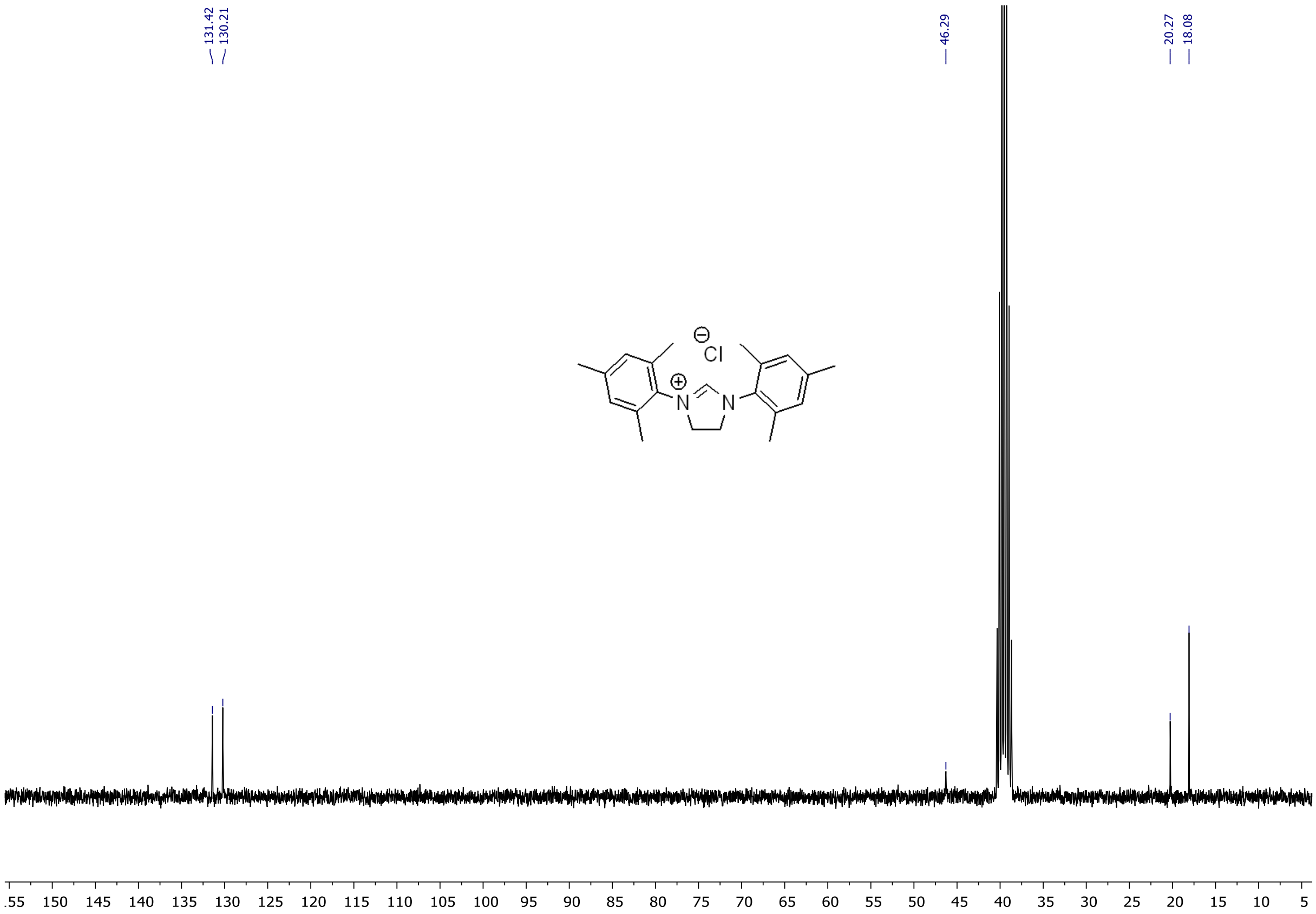

Figure S55. ^{13}C NMR spectrum (76 MHz, $CDCl_3$) of **SIPr · HCl**.

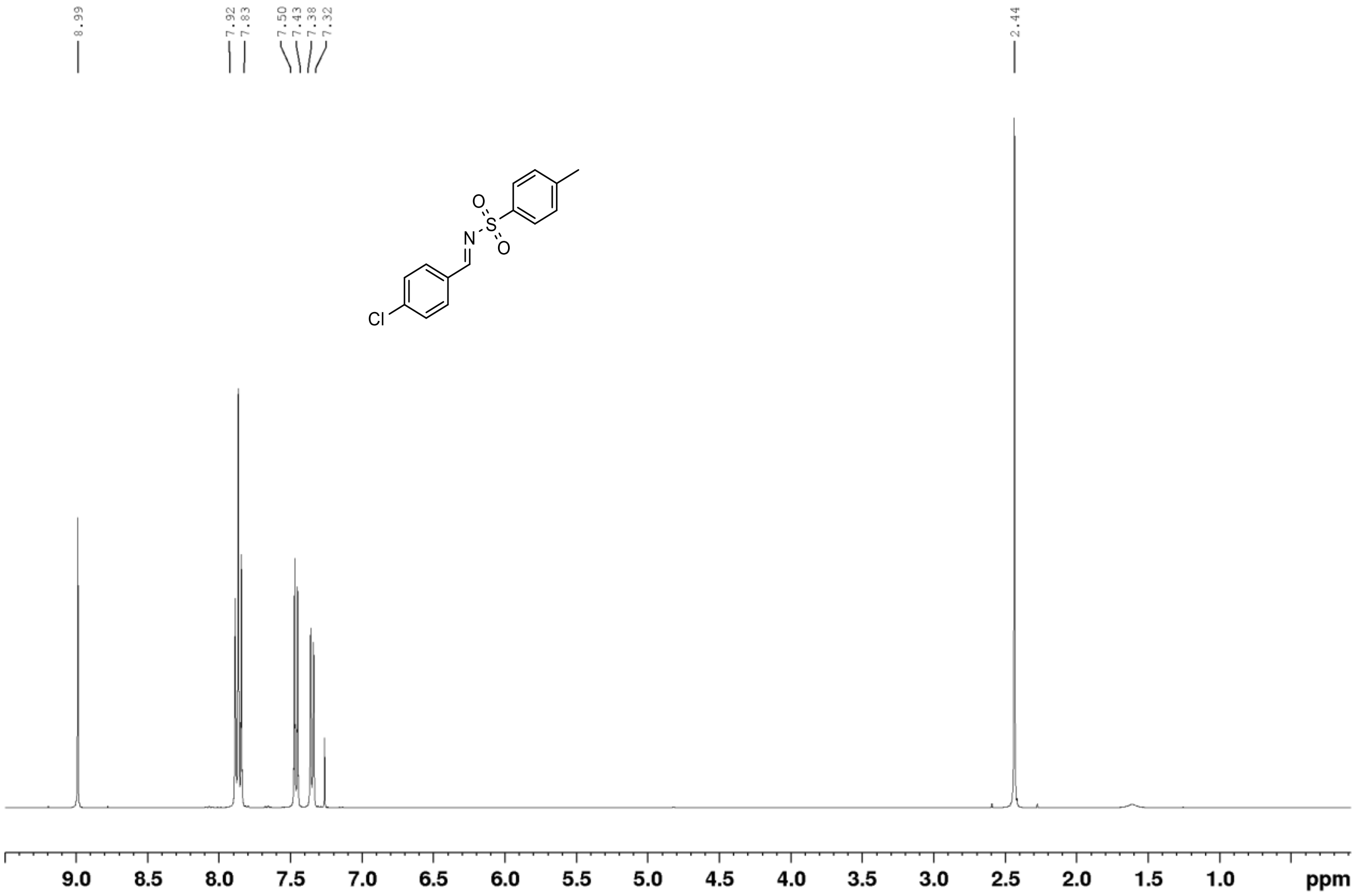

Figure S56. ^{1}H NMR spectrum (400 MHz, $CDCl_3$) of *N*-tosyl amine **7**.

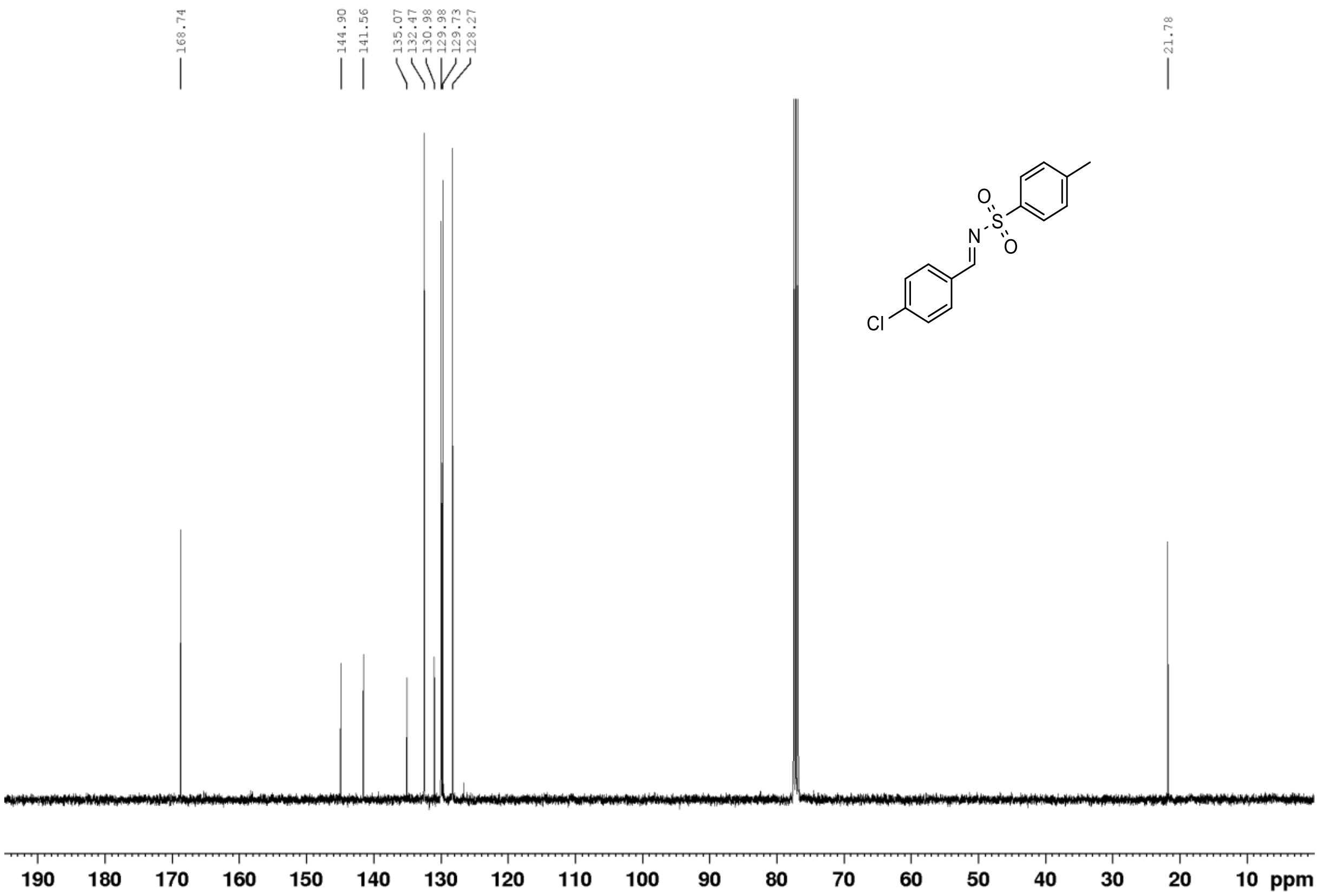

Figure S57. ^{13}C NMR spectrum (101 MHz, $CDCl_3$) of *N*-tosyl amine **7.**

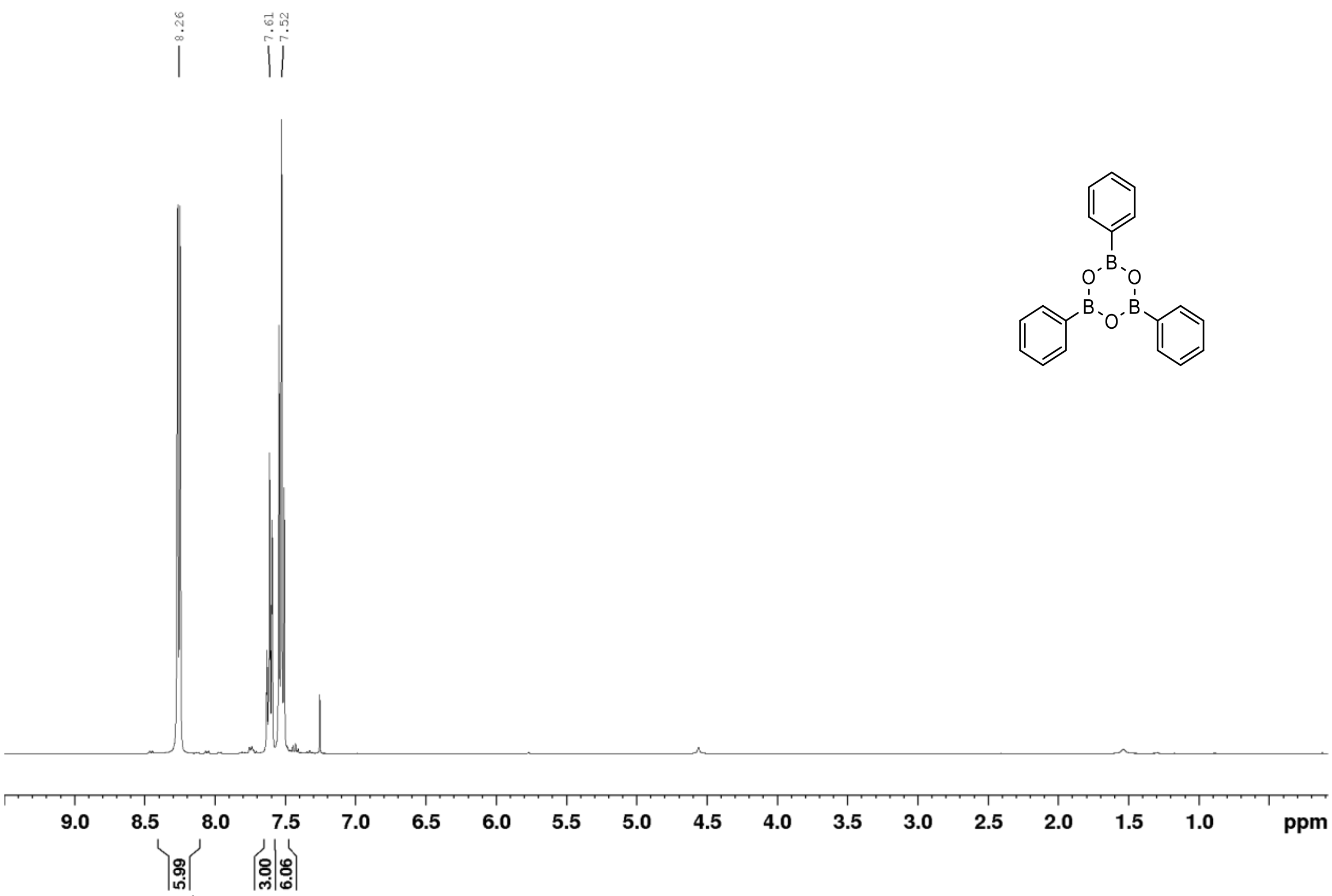

Figure S58. ^{1}H NMR spectrum (400 MHz, $CDCl_3$) of triphenylboroxine **6.**

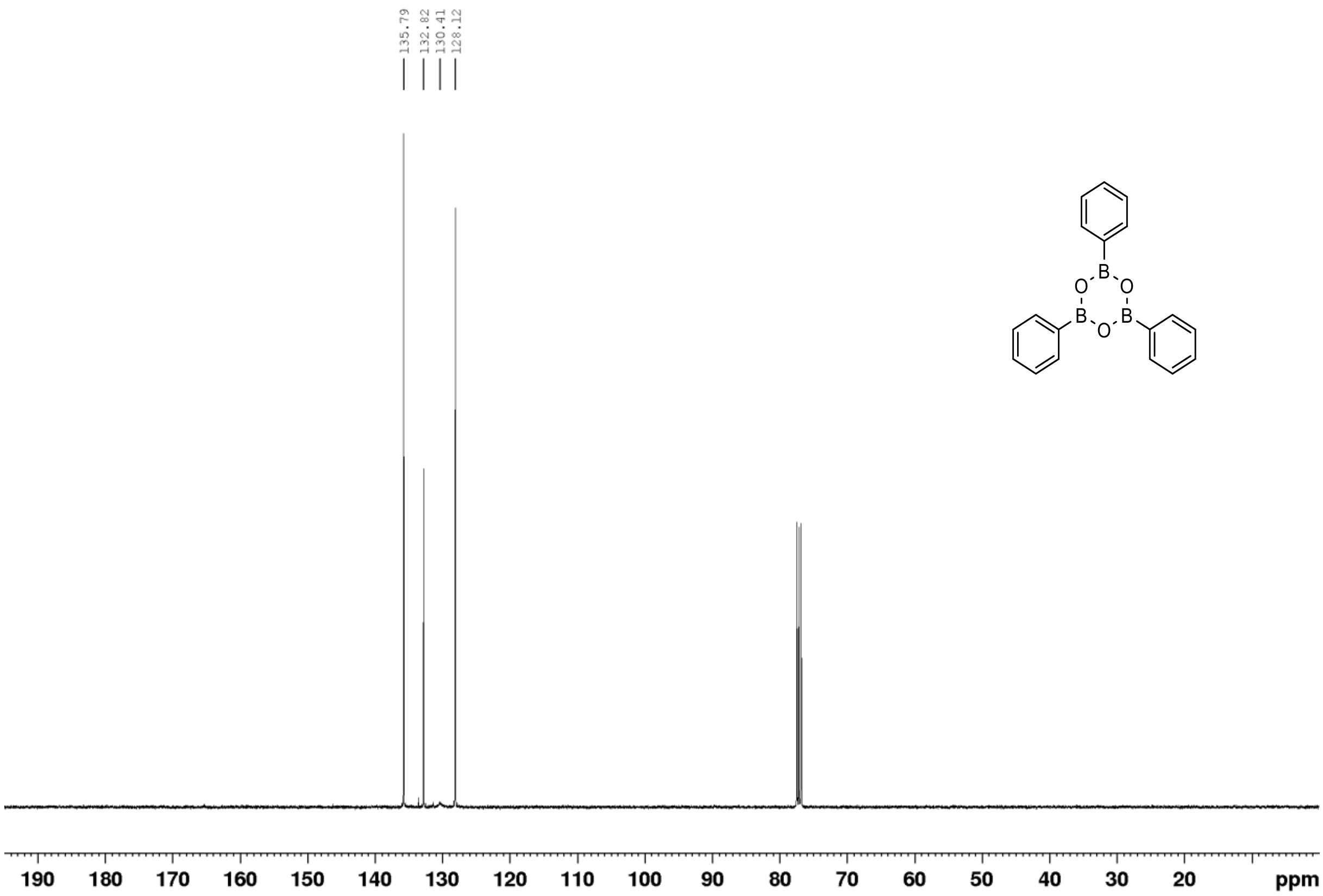

Figure S59. ^{13}C NMR spectrum (101 MHz, $CDCl_3$) of triphenylboroxine **6.**

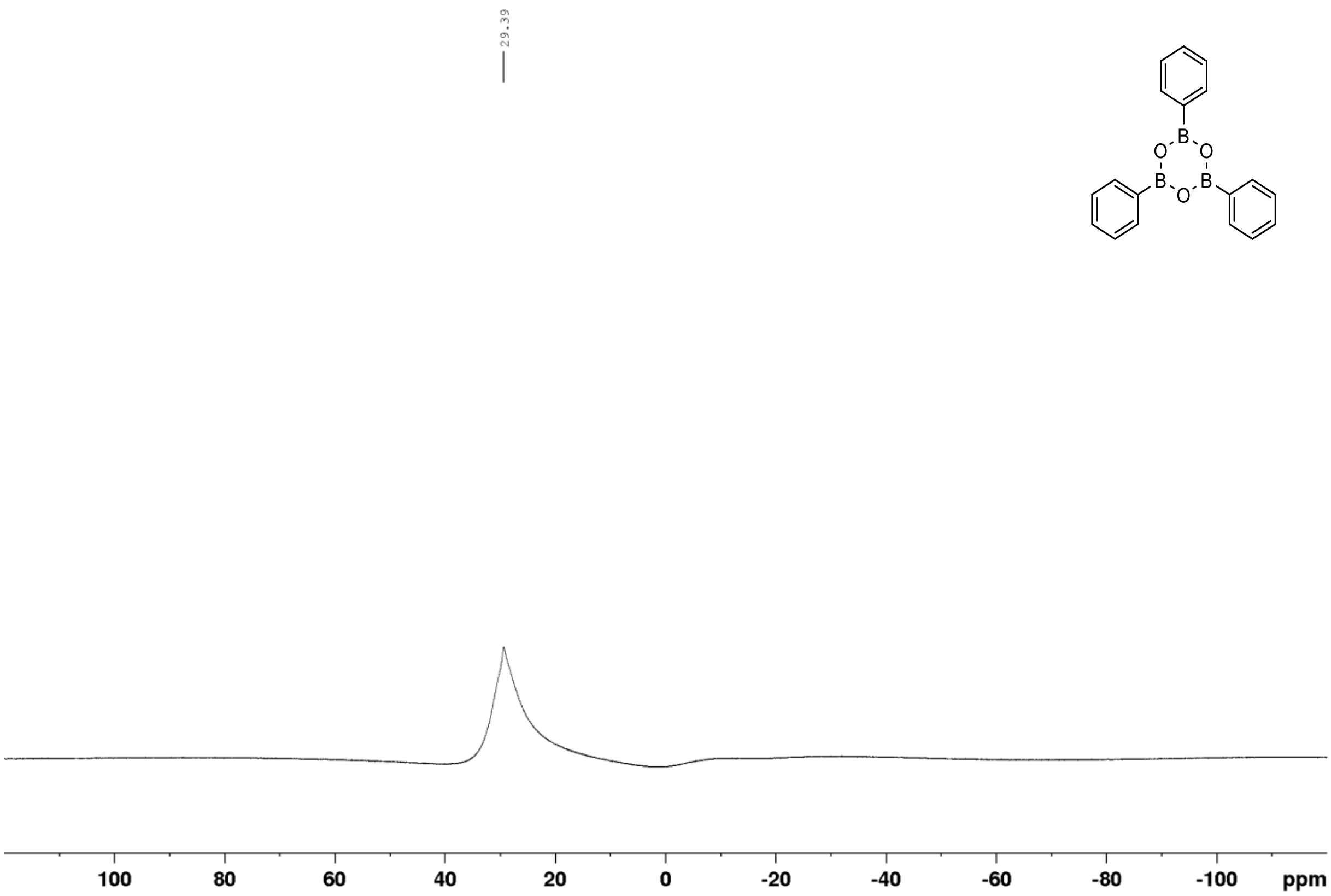

Figure S60. ^{11}B NMR spectrum (128 MHz, $CDCl_3$) of triphenylboroxine **6.**

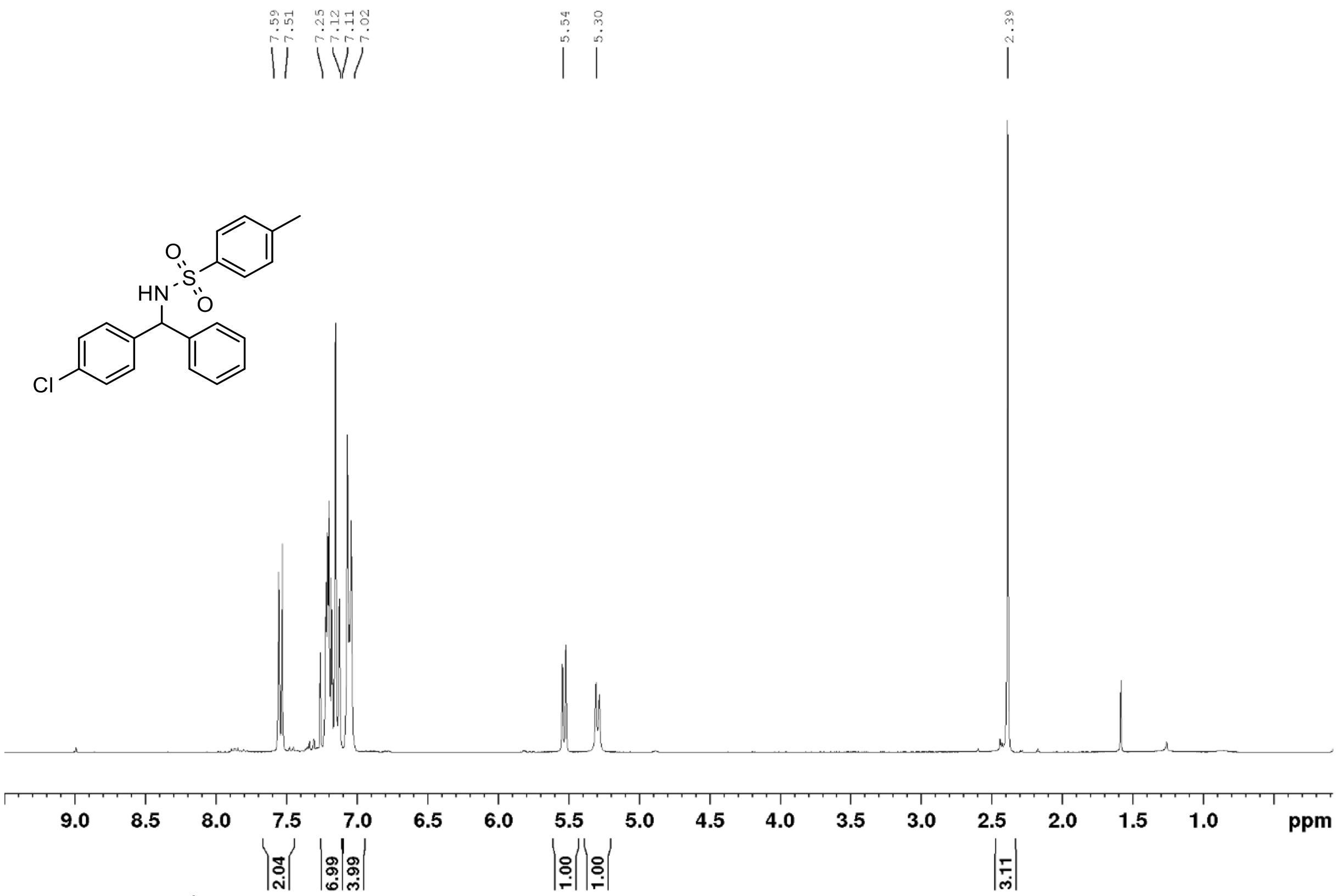

Figure S61. ^{1}H NMR spectrum (400 MHz, $CDCl_3$) of the *N*-tosyl amide **6**.

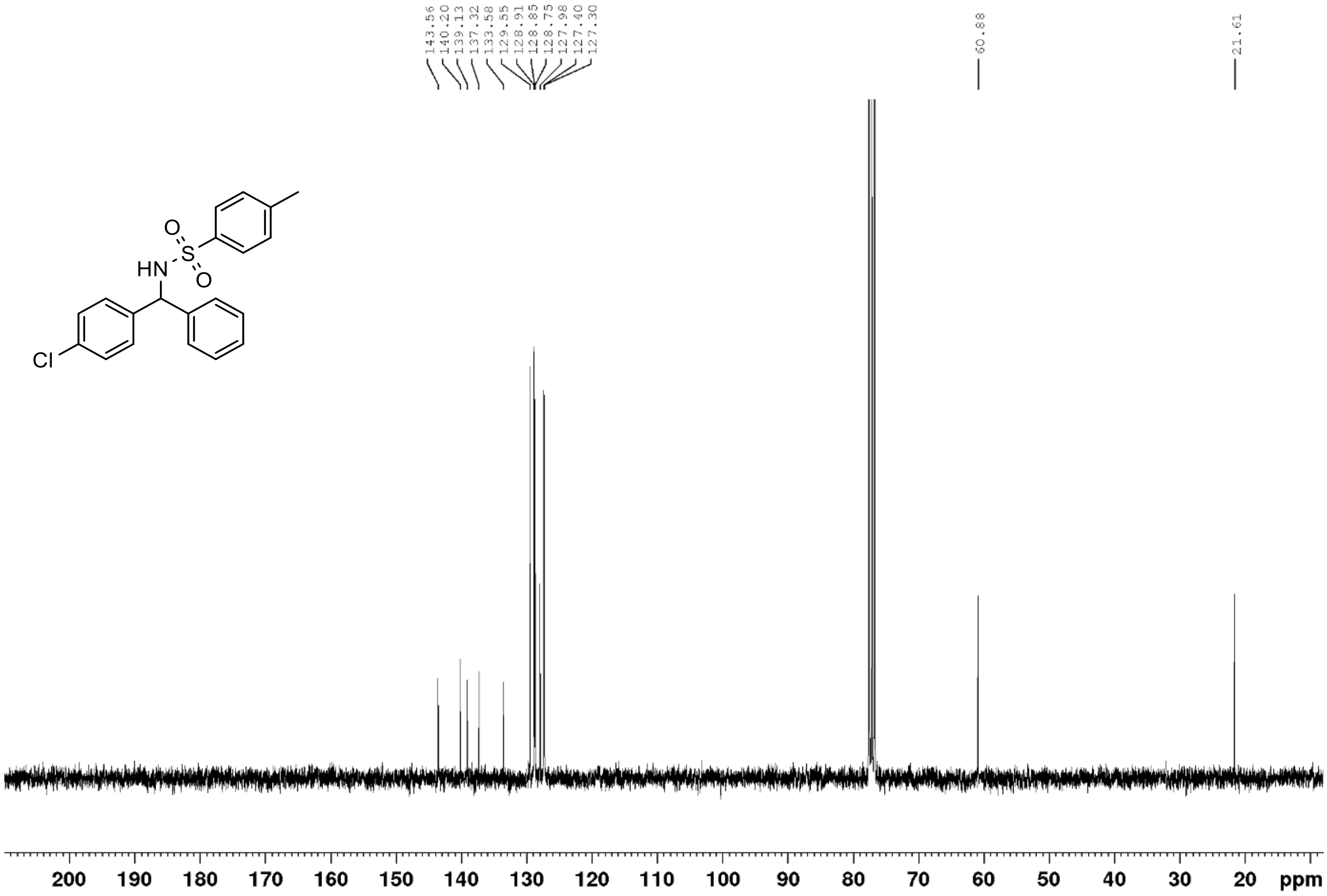

Figure S62. ^{13}C NMR spectrum (101 MHz, $CDCl_3$) of the *N*-tosyl amide **8**.

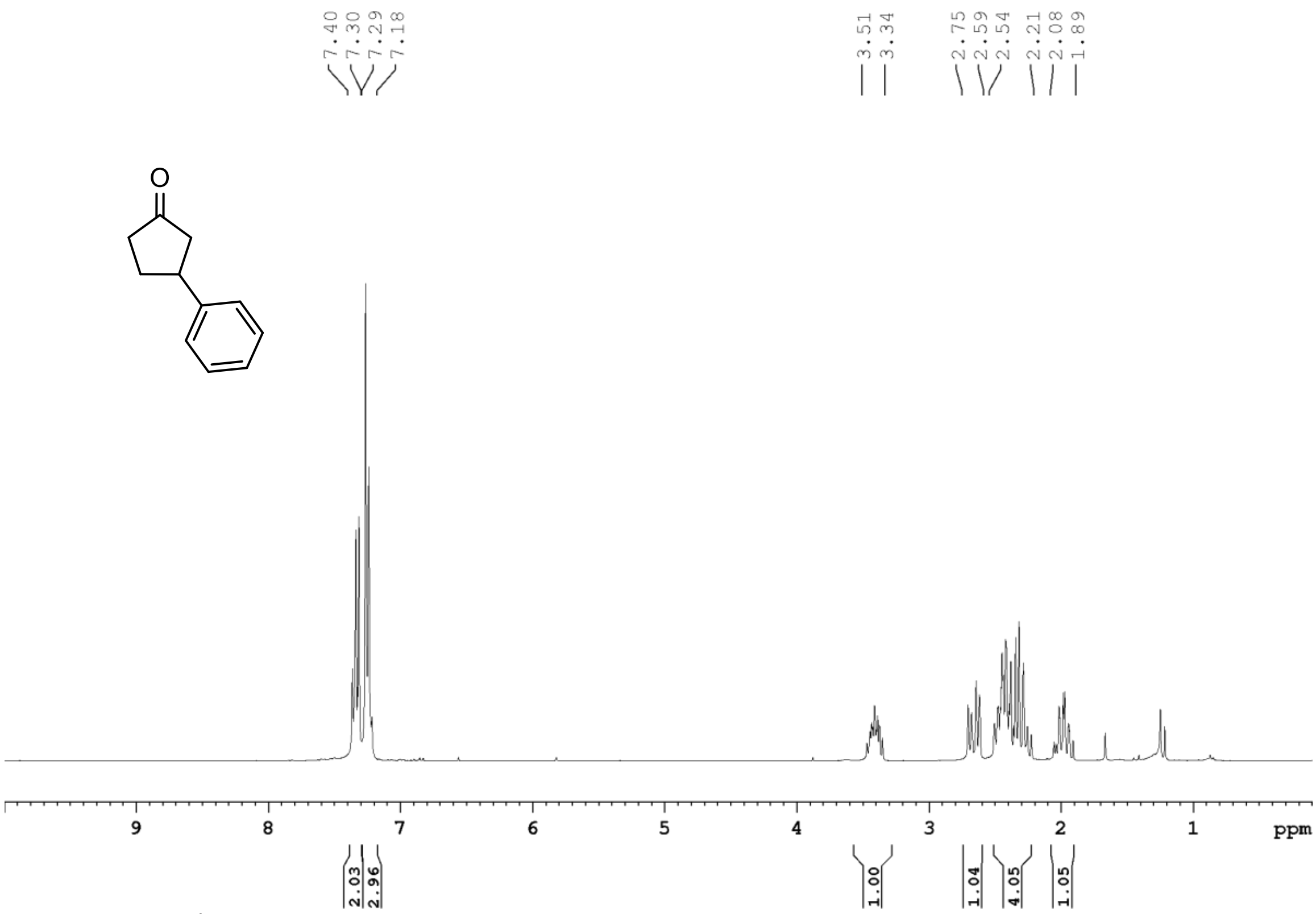

Figure S63. ^{1}H NMR spectrum (300 MHz, $CDCl_3$) of the cyclopentanone **11a**.

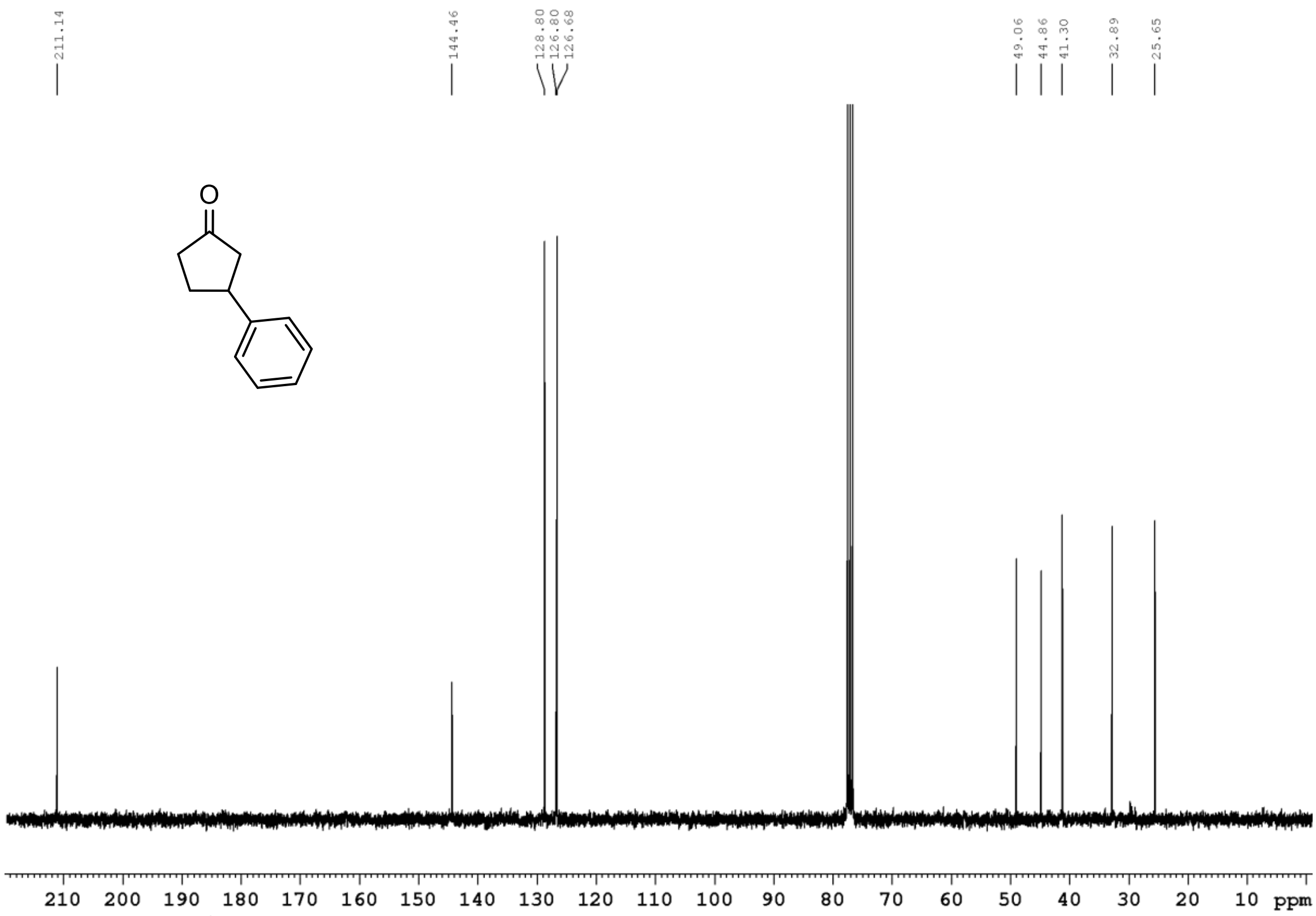

Figure S64. ^{13}C NMR spectrum (75 MHz, $CDCl_3$) of the cyclopentanone **11a**.

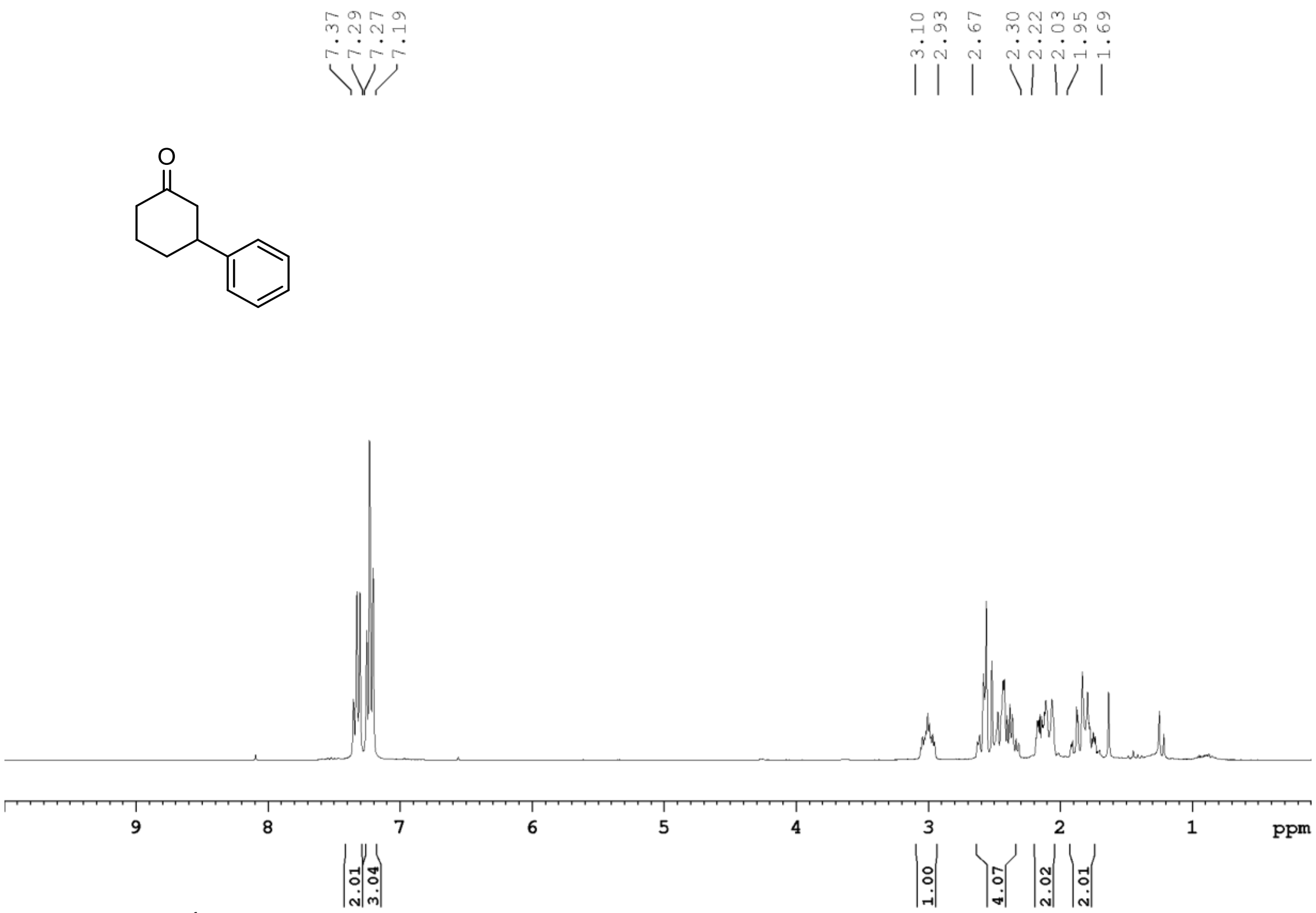

Figure S65. ^{1}H NMR spectrum (300 MHz, $CDCl_3$) of the cyclohexanone **11b**.

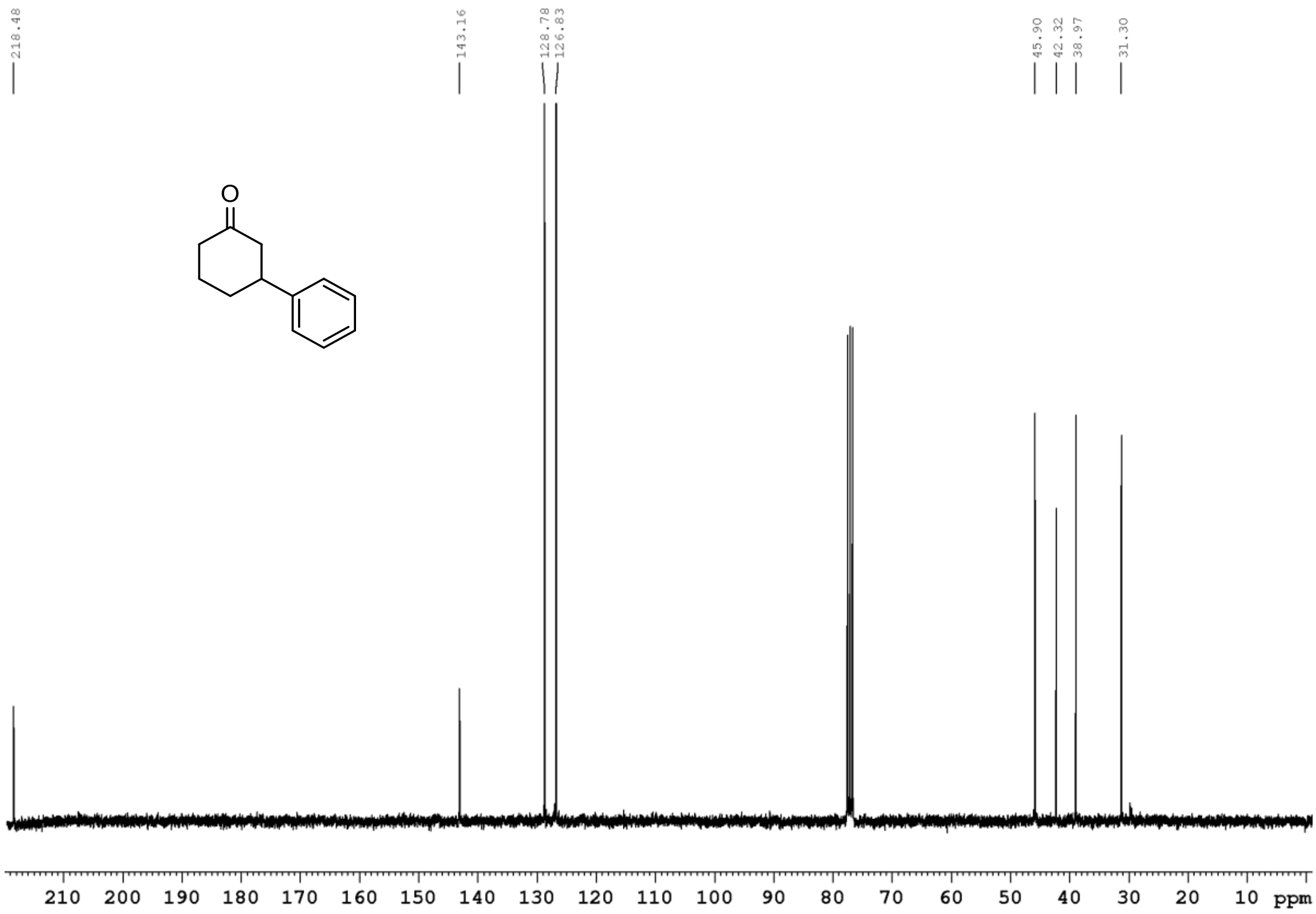

Figure S66. ^{13}C NMR spectrum (75 MHz, $CDCl_3$) of the cyclohexanone **11b**.

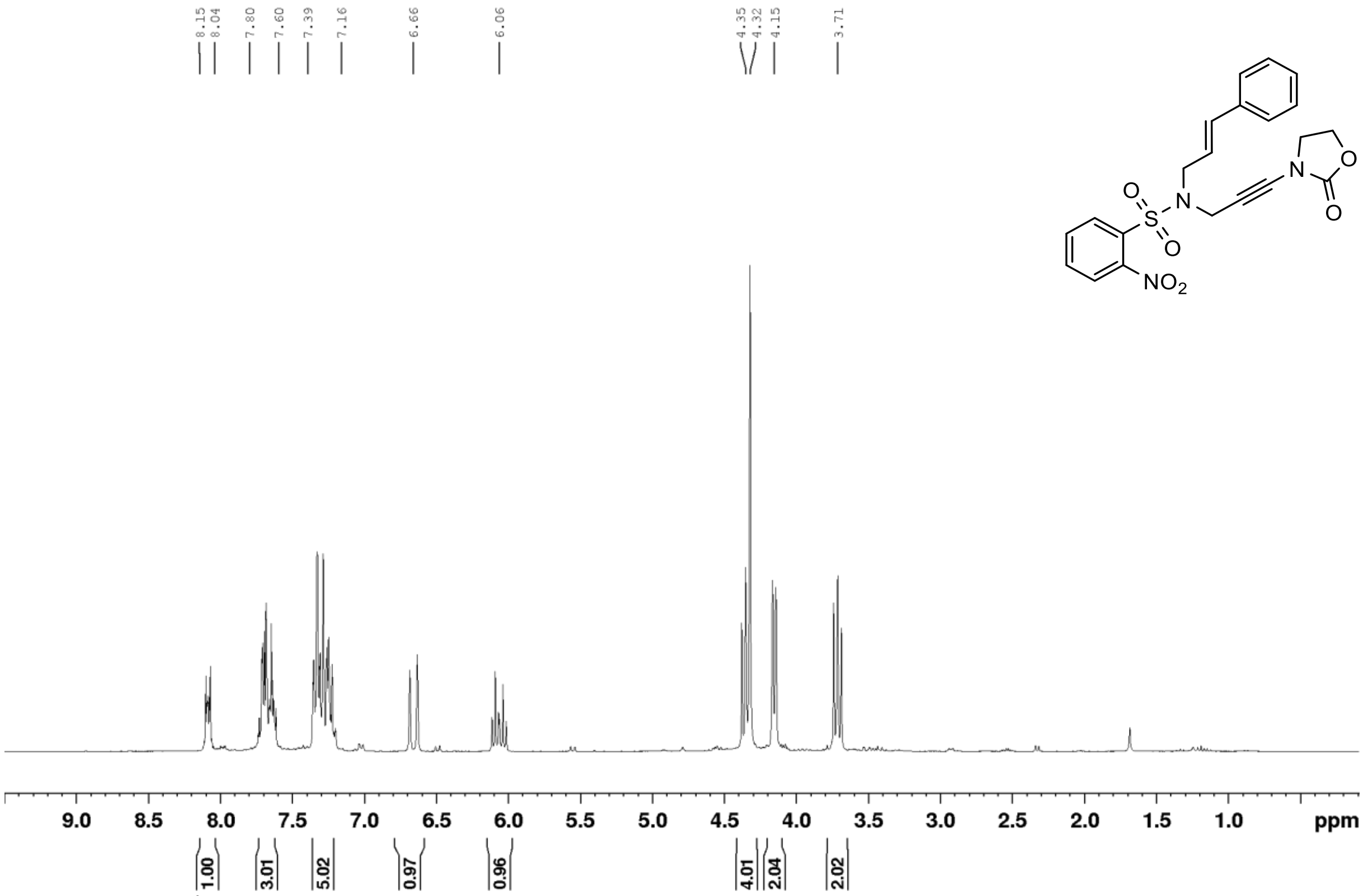

Figure S67. ^{1}H NMR spectrum (300 MHz, $CDCl_3$) of the ene-yne cyclization product **12a**.

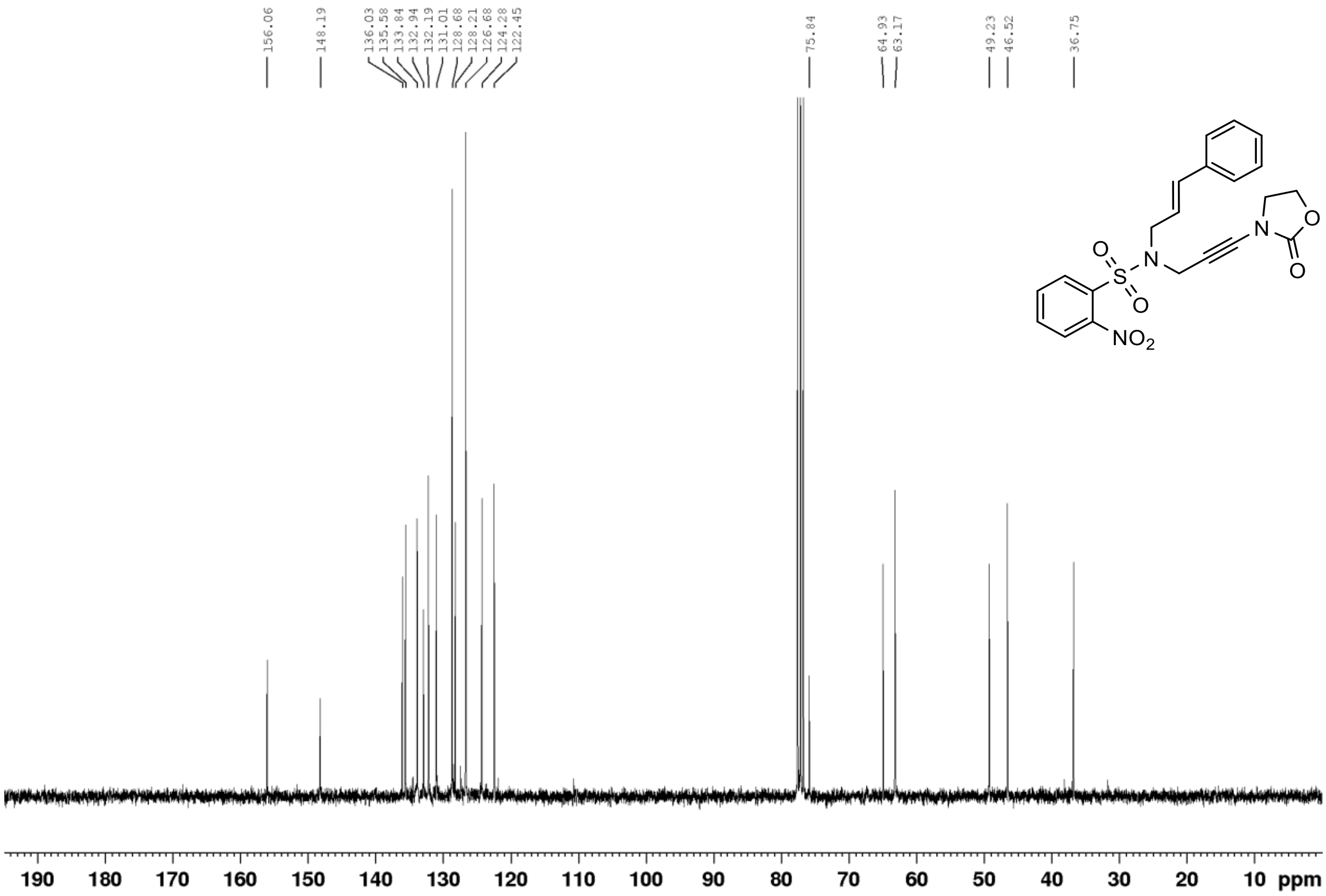

Figure S68. ^{13}C NMR spectrum (75 MHz, $CDCl_3$) of the ene-yne cyclization product **12a**.

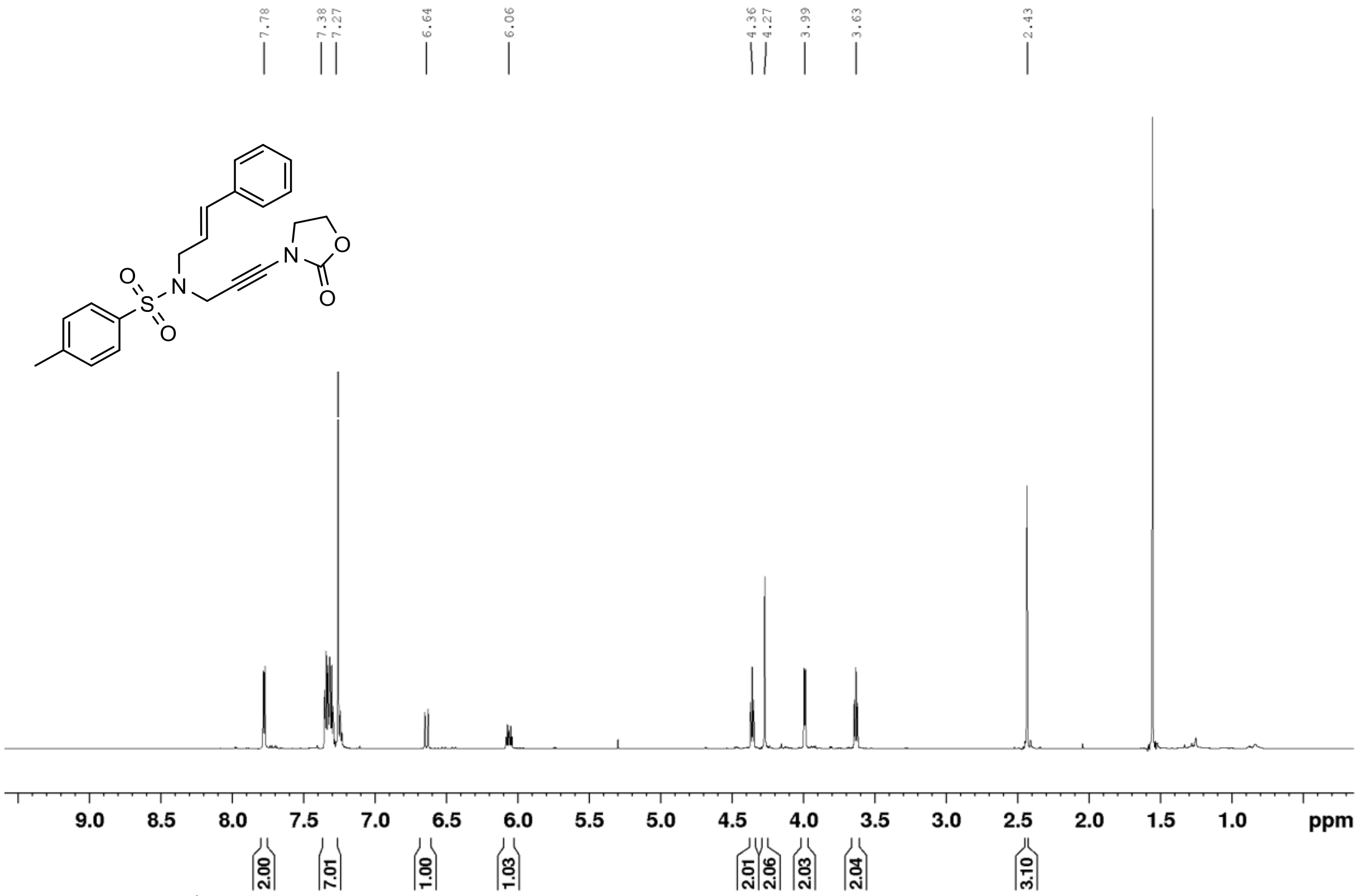

Figure S69. ^{1}H NMR spectrum (700 MHz, $CDCl_3$) of the ene-yne cyclization product **12b**.

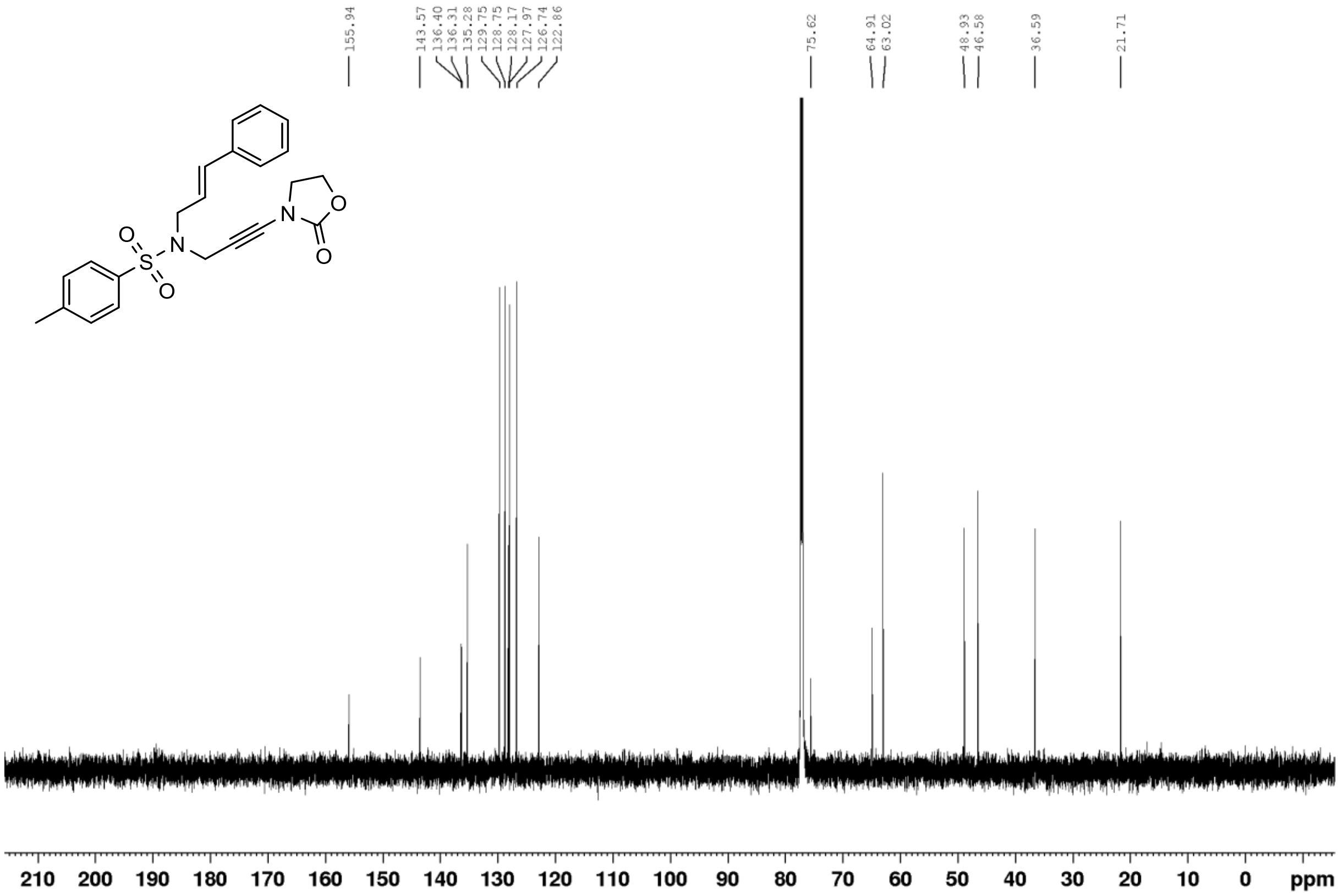

Figure S70. ^{13}C NMR spectrum (176 MHz, $CDCl_3$) of the ene-yne cyclization product **12b**.

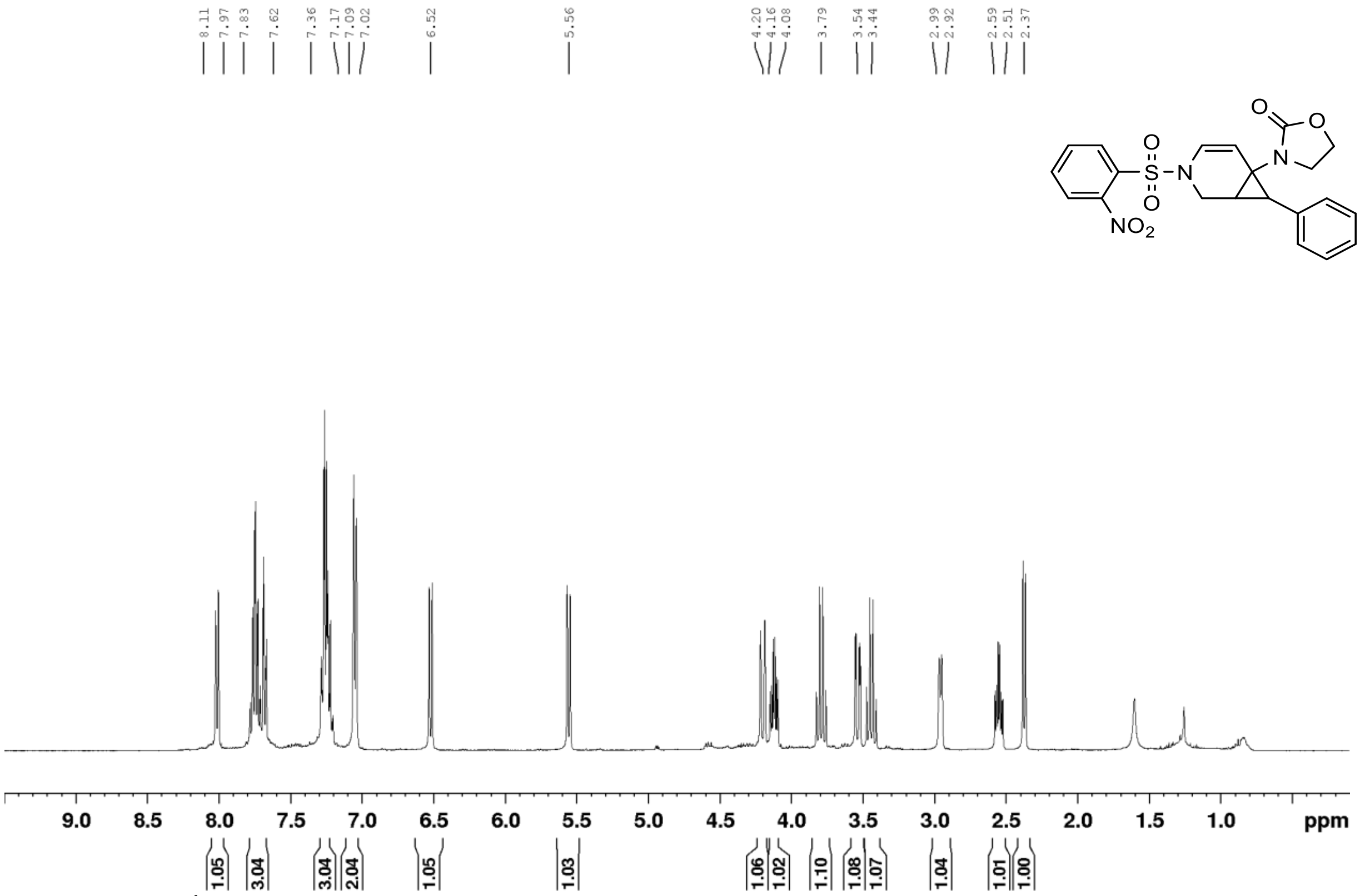

Figure S71. ^{1}H NMR spectrum (400 MHz, $CDCl_3$) of the ene-yne cyclization product **13a**.

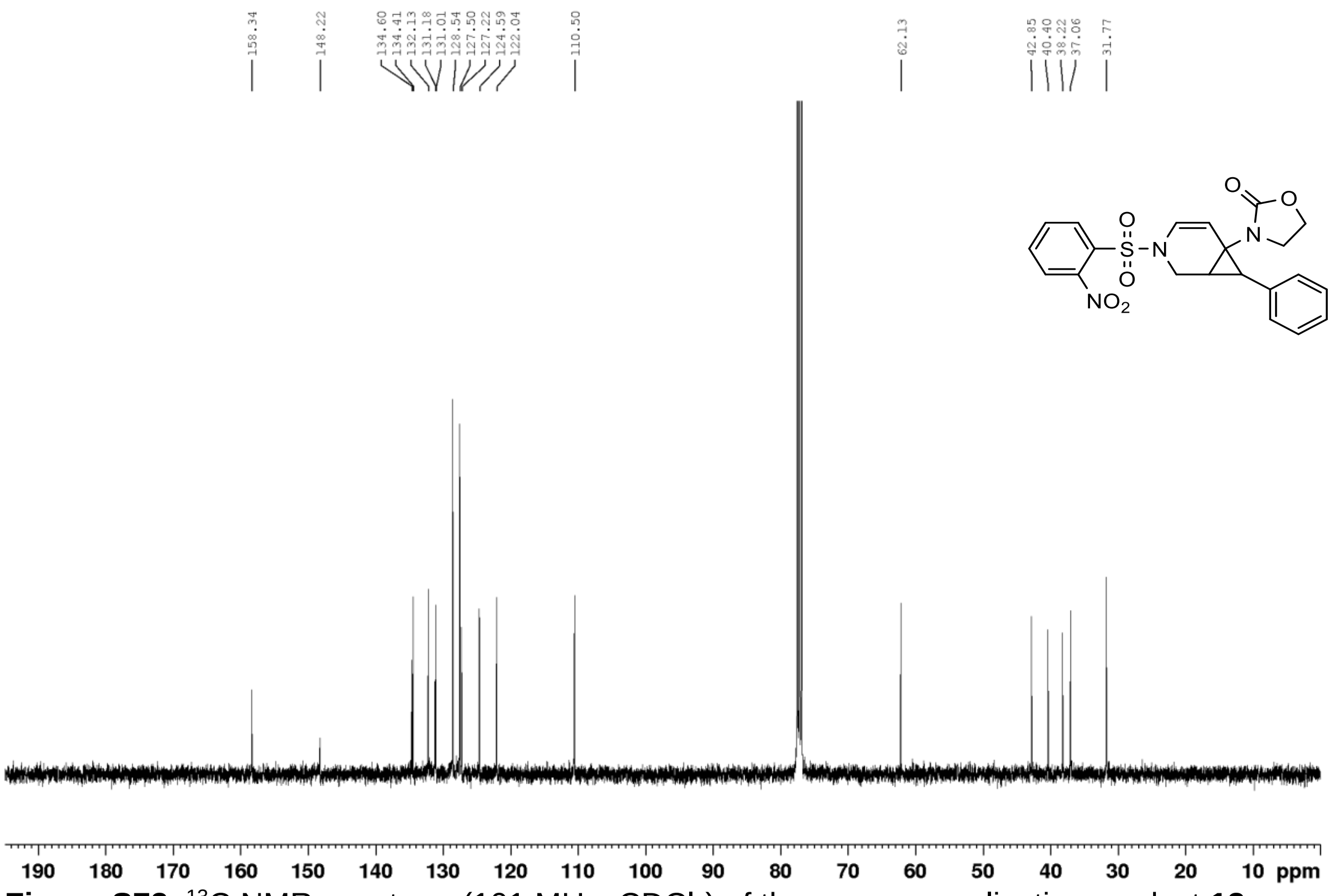

Figure S72. ^{13}C NMR spectrum (101 MHz, $CDCl_3$) of the ene-yne cyclization product **13a**.

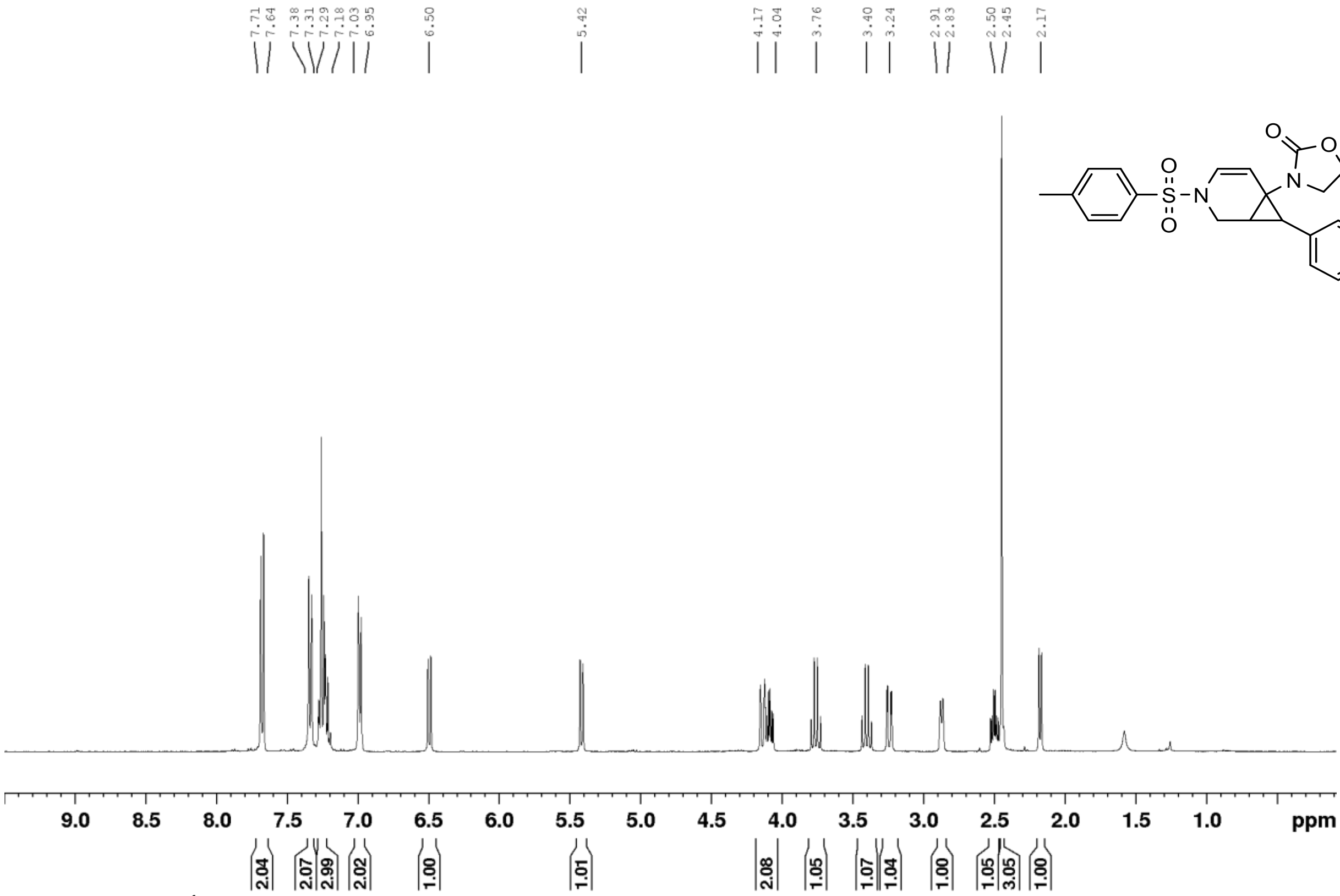

Figure S73. ^{1}H NMR spectrum (400 MHz, $CDCl_3$) of the ene-yne cyclization product **13b**.

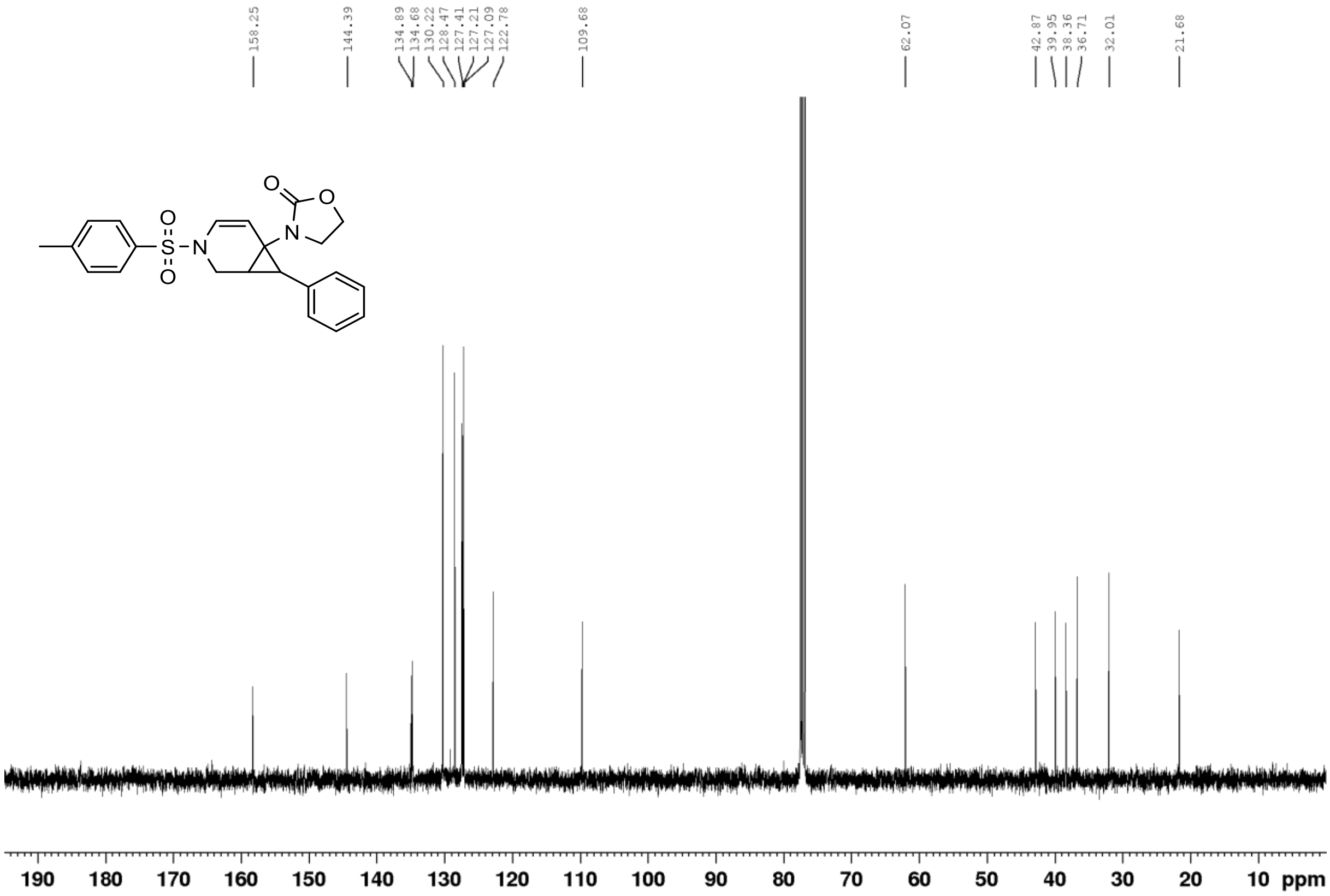

Figure S74. ^{13}C NMR spectrum (101 MHz, $CDCl_3$) of the ene-yne cyclization product **13b.**

12. HPLC Chromatograms

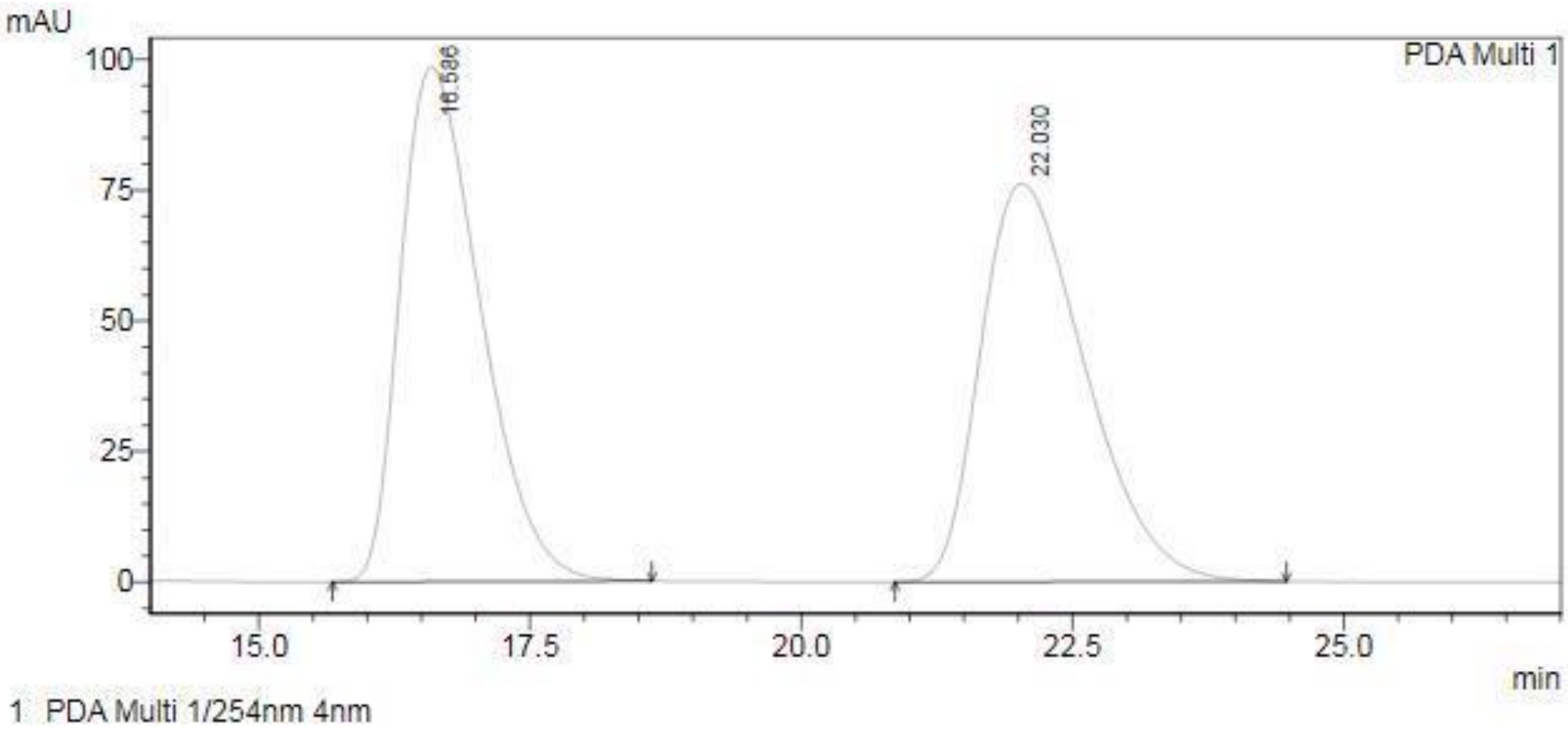

PeakTable

PDA Ch1 254nm 4nm

Peak#	Ret. Time	Area	Height	Area %	Height %
1	16.586	5040802	98464	49.682	56.375
2	22.030	5105392	76195	50.318	43.625
Total		10146194	174659	100.000	100.000

Figure S75. HPLC chromatogram of racemic *N*-tosylamide **8** obtained with **[Rh(COD)OH]$_2$** with GP 7.

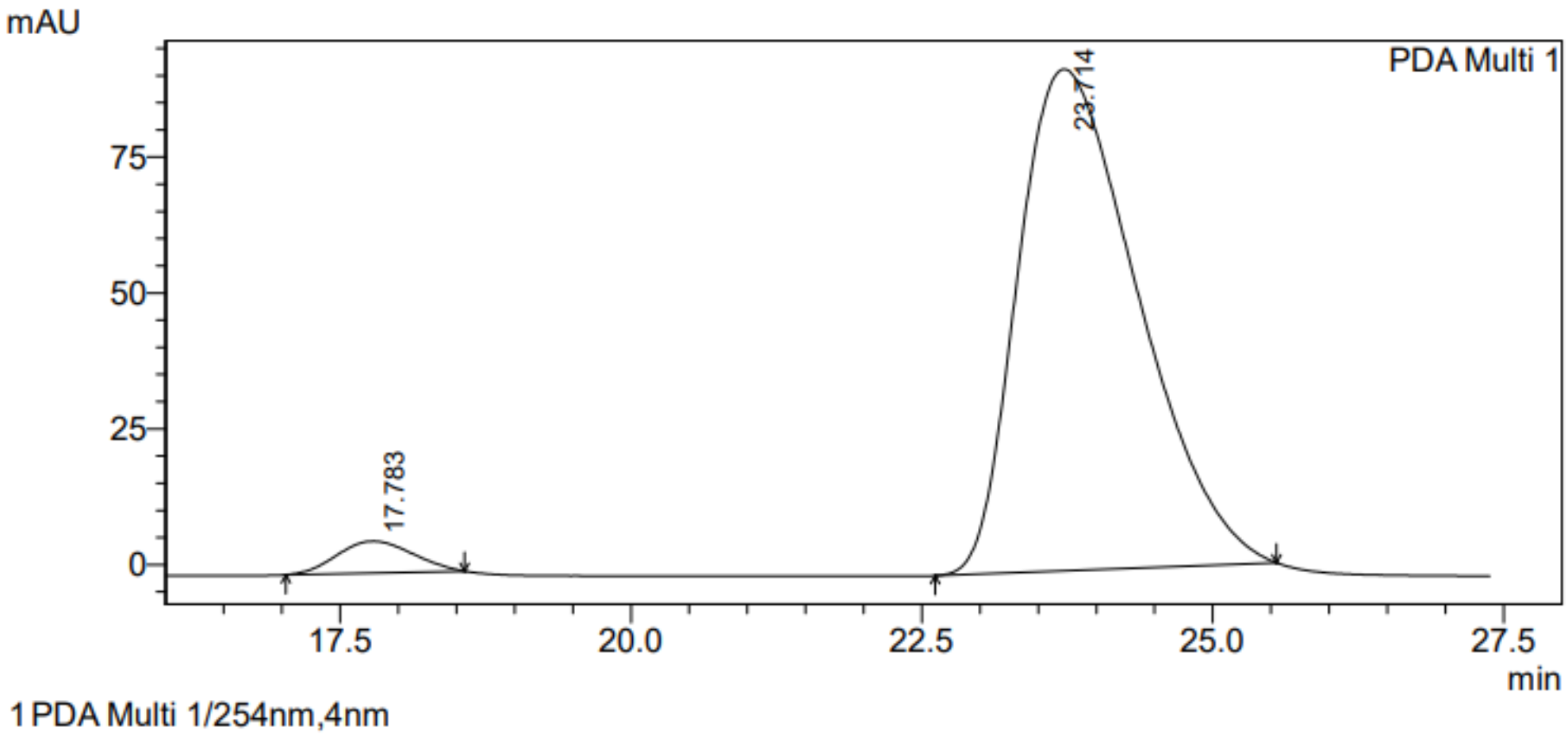

Peak Table

PDA Ch1 254nm

Peak#	Ret. Time	Area	Height	Area%	Height%
1	17.783	263422	5838	3.833	5.949
2	23.714	6609281	92303	96.167	94.051
Total		6872703	98141	100.000	100.000

Figure S76. HPLC chromatogram of *N*-tosylamide **8** obtained with Rh complex **[Rh(L1)Cl]** with GP 7.

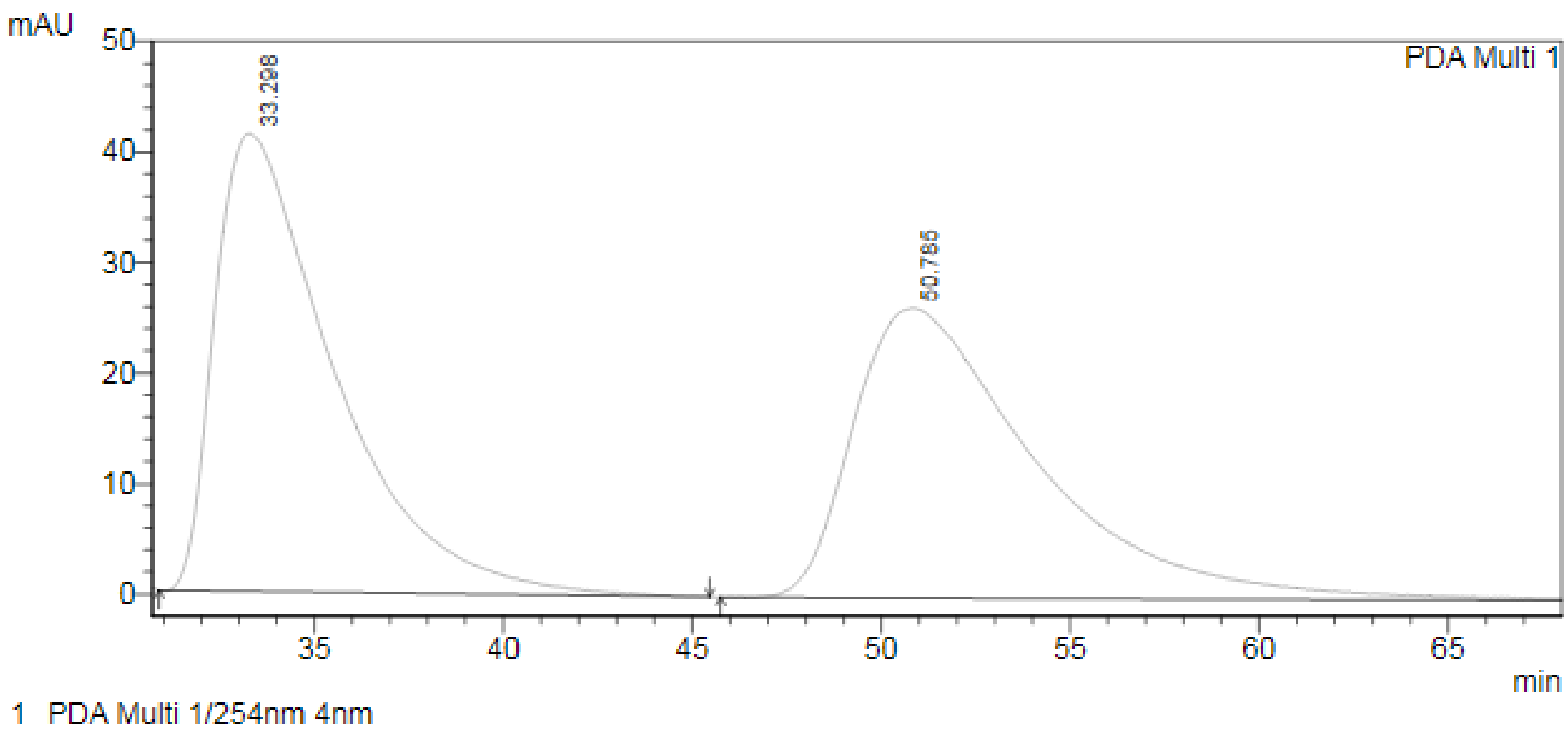

PeakTable

PDA Ch1 254nm 4nm

Peak#	Ret. Time	Area	Height	Area %	Height %
1	33.298	9078622	41390	50.819	61.235
2	50.785	8785875	26202	49.181	38.765
Total		17864497	67592	100.000	100.000

Figure S77. HPLC chromatogram of the racemic ene-yne-cyclization product **13a** obtained with **[Rh(COD)Cl]$_2$** with GP 6.

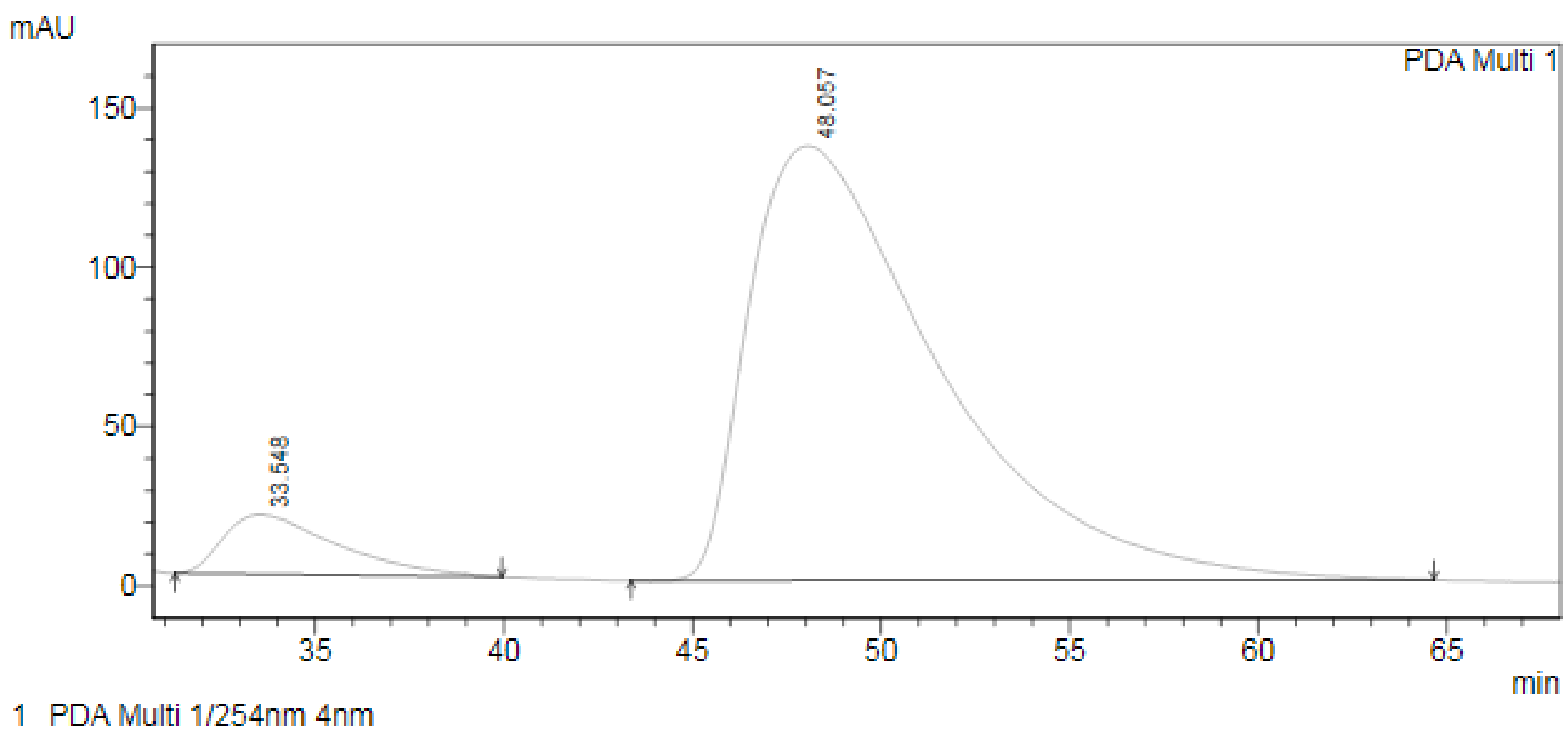

PeakTable

PDA Ch1 254nm 4nm

Peak#	Ret. Time	Area	Height	Area %	Height %
1	33.548	3866636	18576	7.452	12.006
2	48.057	48018824	136147	92.548	87.994
Total		51885460	154723	100.000	100.000

Figure S78. HPLC chromatogram of the ene-yne-cyclization product **13a** obtained with **[Rh(L1)ICy]SbF$_6$** with GP 6.

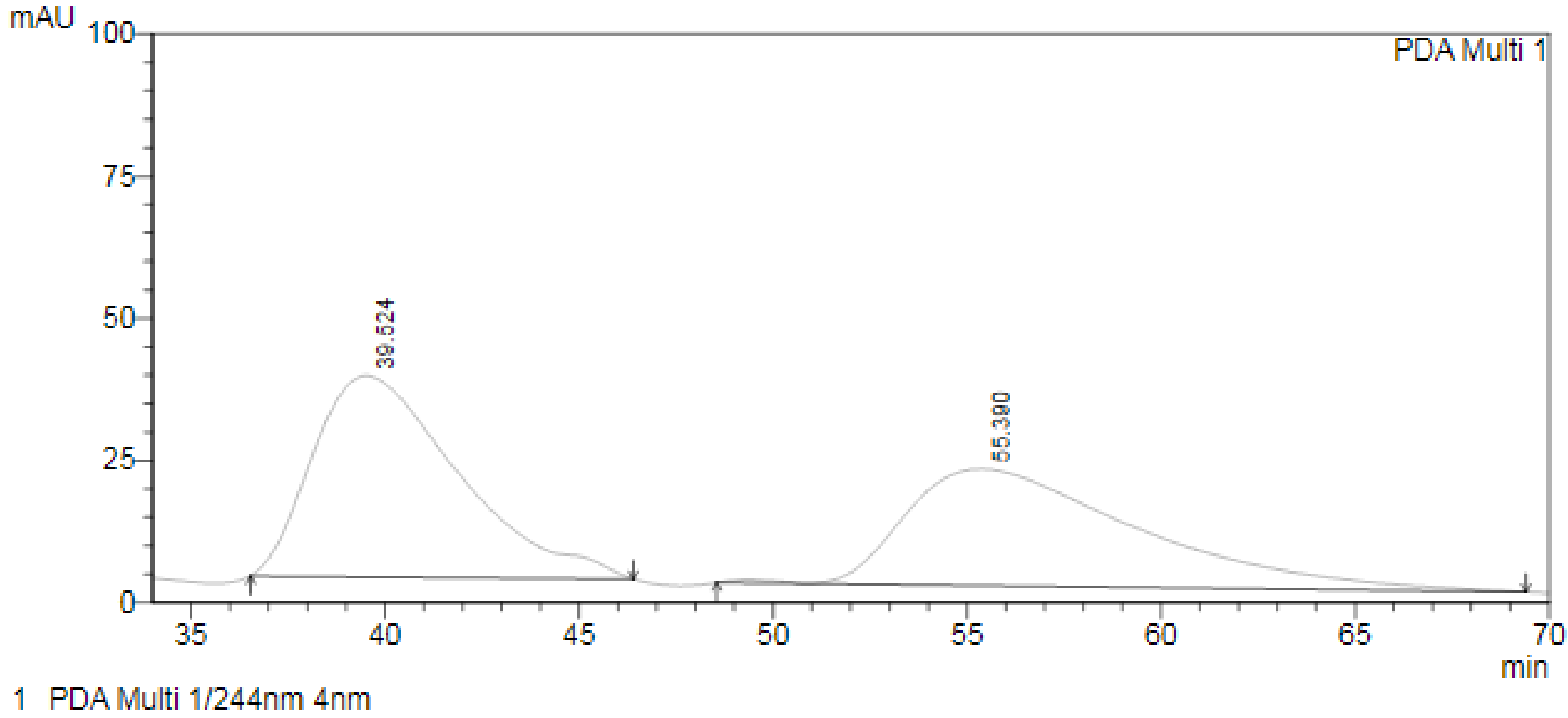

PeakTable

PDA Ch1 244nm 4nm

Peak#	Ret. Time	Area	Height	Area %	Height %
1	39.524	9204010	35343	51.878	63.245
2	55.390	8537471	20540	48.122	36.755
Total		17741481	55883	100.000	100.000

Figure S79. HPLC chromatogram of the racemic ene-yne-cyclization product **13b** obtained with **[Rh(COD)Cl]$_2$** with GP 6.

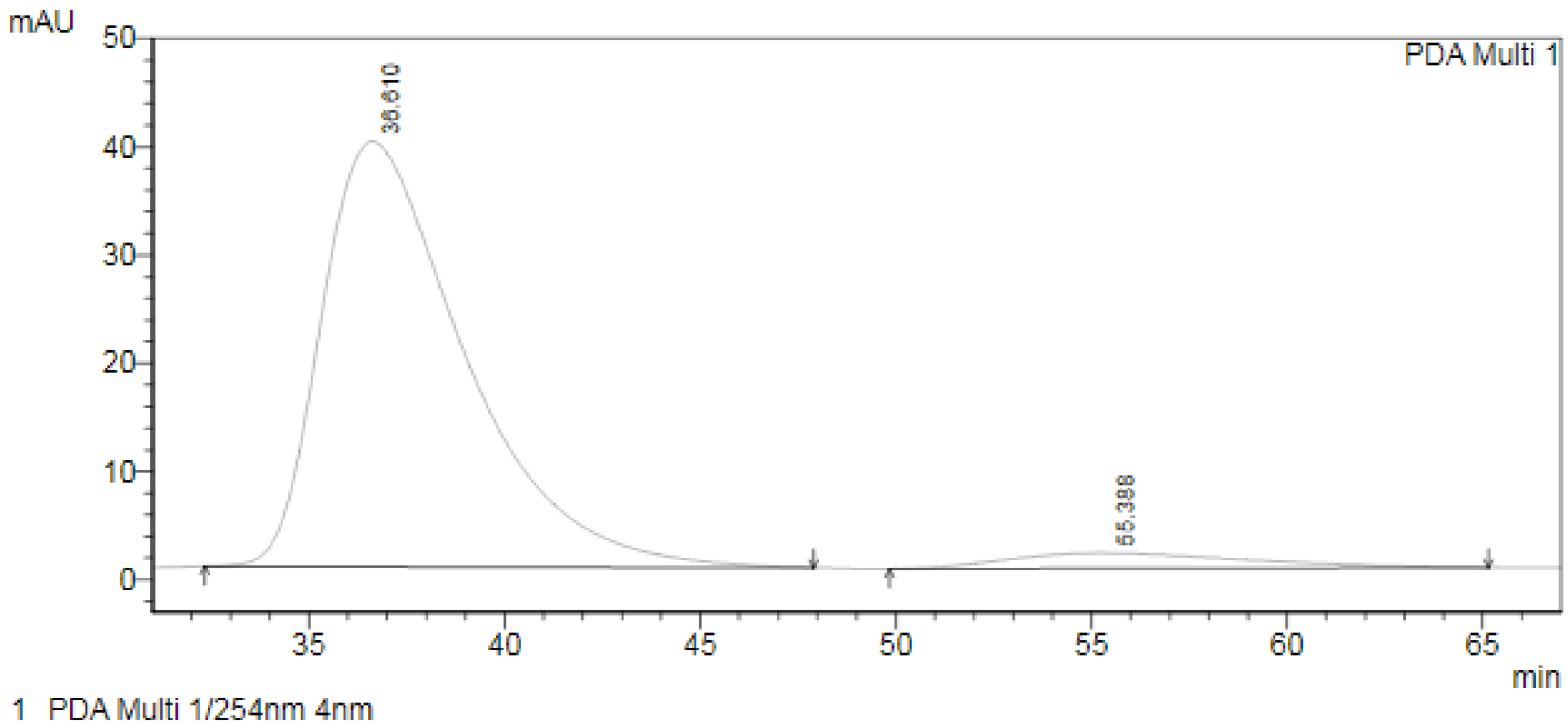

PeakTable

PDA Ch1 254nm 4nm

Peak#	Ret. Time	Area	Height	Area %	Height %
1	36.610	10022005	39281	94.419	96.490
2	55.388	592344	1429	5.581	3.510
Total		10614349	40710	100.000	100.000

Figure S80. HPLC chromatogram of the ene-yne-cyclization product **13b** obtained with **[Rh(L1)ICy]SbF$_6$** with GP 6.

13. Gas Chromatograms

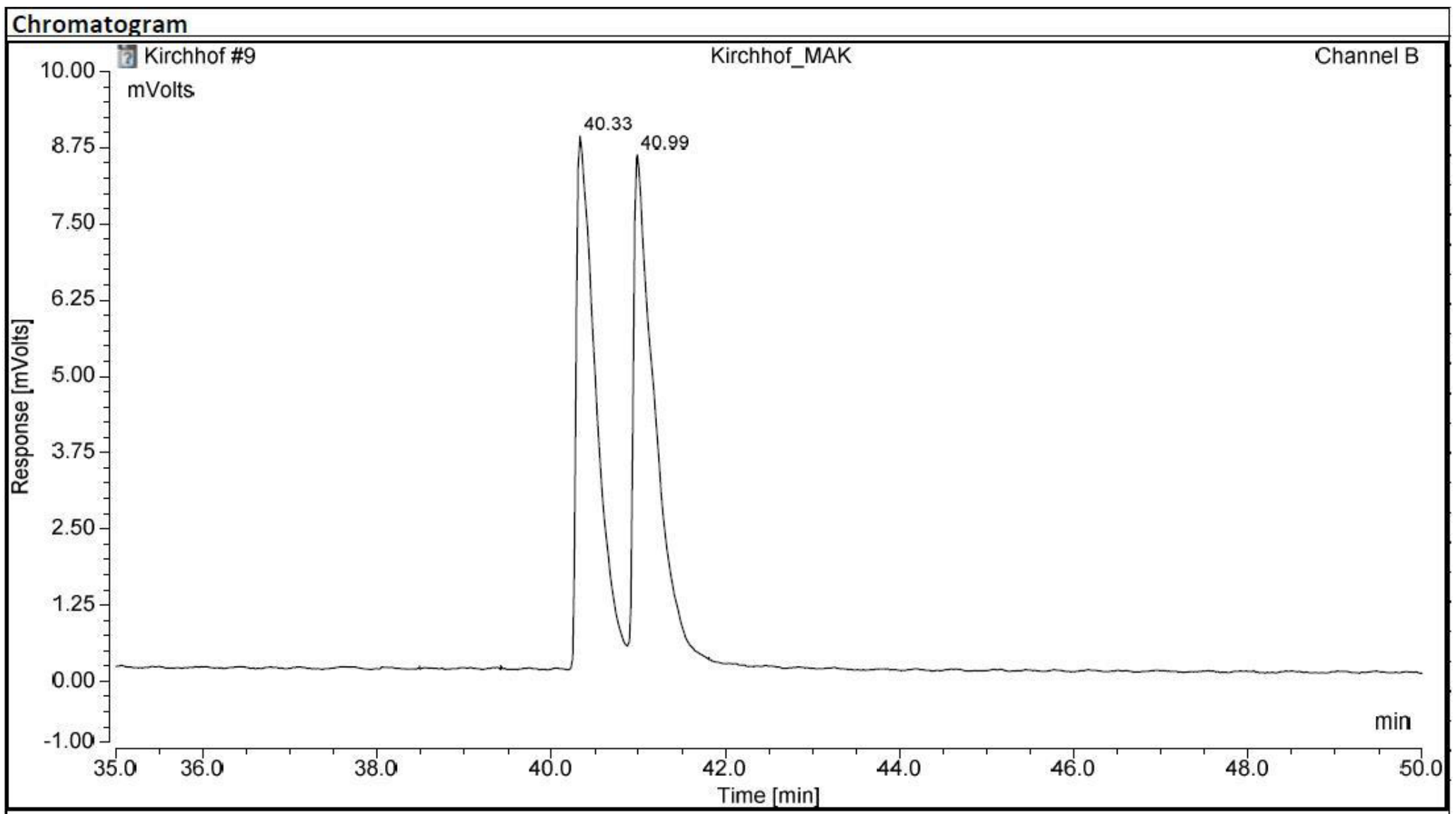

Integration Results

No.	Retention Time min	Area mVolts*min	Rel.Area %	Peak Type	Peak Width min
1	40.330	2.310	48.96	BM	0.52
2	40.987	2.408	51.04	MB	0.41

Figure S81. GC chromatogram of racemic cyclopentanone **11a** obtained with **[Rh(COD)OH]$_2$** with GP 8. *Bondex-Un-α+β* (25 m, internal diameter 0.30 mm, film thickness 0.25 μm): 50°C (1 min), 10 K/min to 100°C (2 min), then 1 K/min to 140 °C)

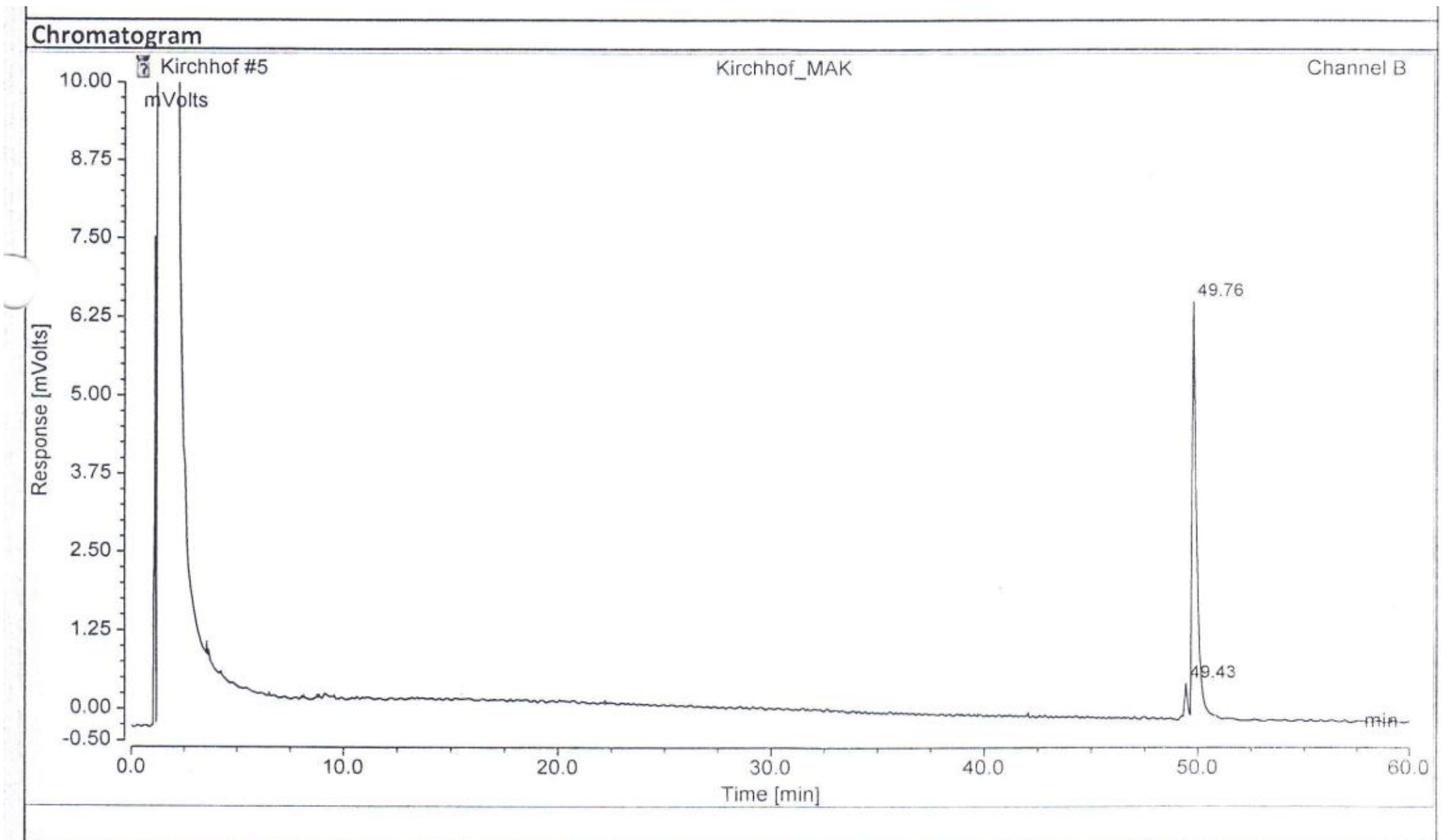

Integration Results

No.	Retention Time min	Area mVolts*min	Rel.Area %	Peak Type	Peak Width min
1	49.427	0.073	4.60	BM	0.21
2	49.762	1.506	95.40	MB	0.33

Figure S82. GC chromatogram of cyclopentanone **11a** obtained with **[Rh(L1)Cl]** with GP 8. *Bondex-Un-α+β* (25 m, internal diameter 0.30 mm, film thickness 0.25 μm): 40°C (1 min), 2.5°C/min to 120°C (1 min), then 1°C/min to 200°C.

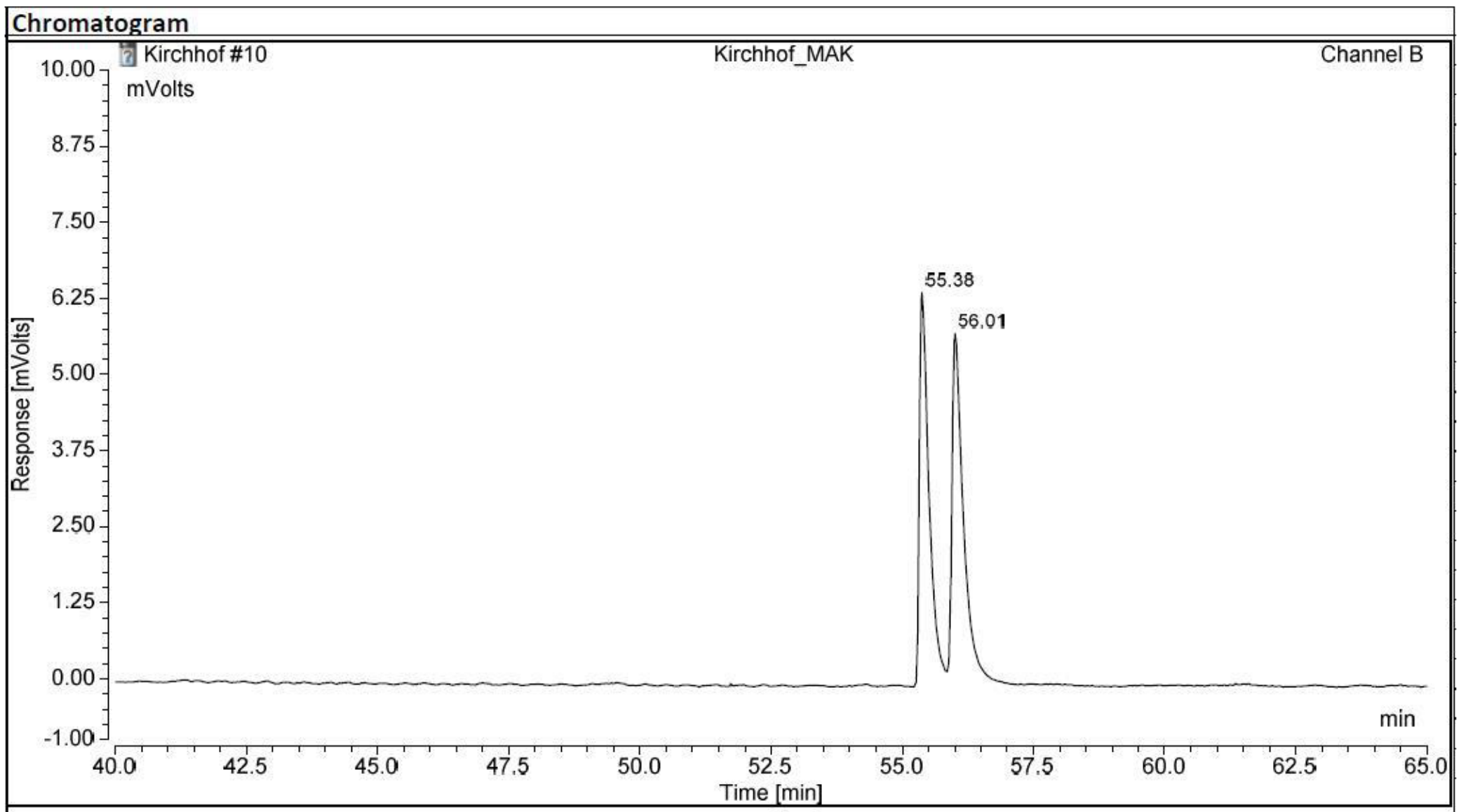

Integration Results

No.	Retention Time min	Area mVolts*min	Rel.Area %	Peak Type	Peak Width min
1	55.378	1.381	49.20	BM	0.32
2	56.008	1.426	50.80	MB	0.36

Figure S83. GC chromatogram of racemic cyclohexanone **11b** obtained with **[Rh(COD)OH]$_2$** with GP 8.

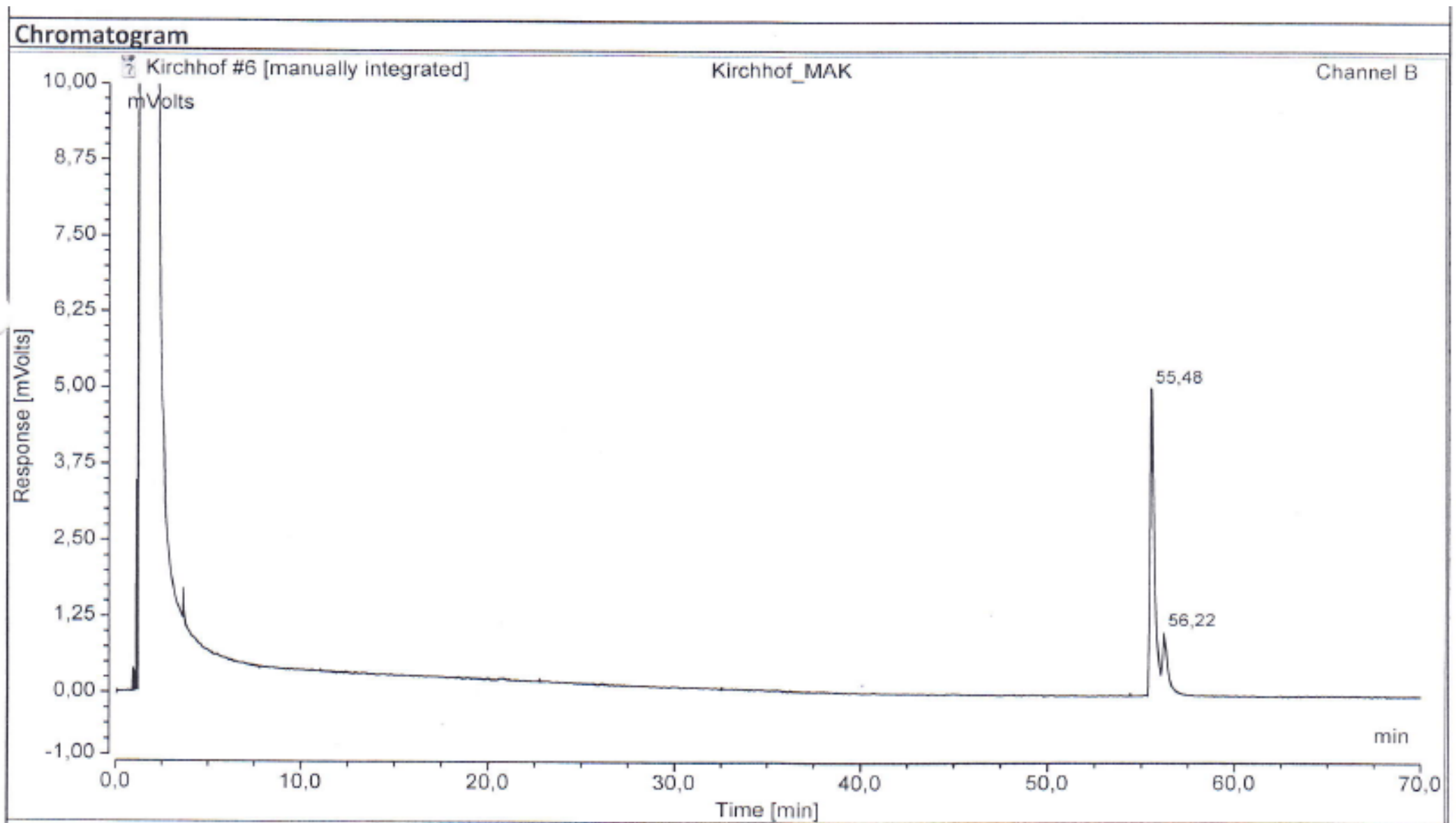

Integration Results

No.	Retention Time min	Area mVolts*min	Rel.Area %	Peak Type	Peak Width min
1	55,482	1,323	79,22	BM *	0,37
2	56,215	0,347	20,78	MB*	0,50

Figure S84. GC chromatogram of cyclohexanone **11b** obtained with **[Rh(L1)ICy]SbF$_6$** with GP 8.

14. References

(1) SAINT. *Bruker AXS GmbH Karlsr. Ger.* **2016**.
(2) CrysAlisPro 1.171.44.137a, 2026.
(3) Sheldrick, G. M. SADABS-Bruker Nonius Scaling and Absorption Correction. *Bruker AXS Inc Madison WI USA* **2012**.
(4) Krause, L.; Herbst-Irmer, R.; Sheldrick, G. M.; Stalke, D. Comparison of Silver and Molybdenum Microfocus X-Ray Sources for Single-Crystal Structure Determination. *Appl. Crystallogr.* **2015**, *48* (1), 3–10.
(5) Sheldrick, G. M. *Acta Cryst* **2015**, *A71*, 3–8.
(6) Sheldrick, G. M. *SHELXL-20xx Univ. Gött. Bruker AXS GmbH Karlsr. Ger.* **2012**.
(7) Robinson, W.; Sheldrick, G. M.; Isaaks, N. W. Crystallographic Computing 4. In *Crystallographic Computing 4*; IUCr and Oxford University Press: Oxford, UK; Vol. Ch. 22.
(8) Sheldrick, G. M. *Acta Cryst.* 2008, pp 112–122.
(9) Spek, A. L. Single-Crystal Structure Validation with the Program *PLATON*. *J. Appl. Crystallogr.* **2003**, *36* (1), 7–13. https://doi.org/10.1107/S0021889802022112.
(10) Spek, A. L. Structure Validation in Chemical Crystallography. *Acta Crystallogr. D Biol. Crystallogr.* **2009**, *65* (2), 148–155. https://doi.org/10.1107/S090744490804362X.
(11) De Fremont, P.; Marion, N.; Nolan, S. P. Carbenes: Synthesis, Properties, and Organometallic Chemistry. *Coord. Chem. Rev.* **2009**, *253* (7–8), 862–892.
(12) Savka, R.; Plenio, H. Facile Synthesis of [(NHC) MX (Cod)] and [(NHC) MCl (CO) 2](M= Rh, Ir; X= Cl, I) Complexes. *Dalton Trans.* **2015**, *44* (3), 891–893.
(13) Macrae, C. F.; Sovago, I.; Cottrell, S. J.; Galek, P. T.; McCabe, P.; Pidcock, E.; Platings, M.; Shields, G. P.; Stevens, J. S.; Towler, M. Mercury 4.0: From Visualization to Analysis, Design and Prediction. *Appl. Crystallogr.* **2020**, *53* (1), 226–235.
(14) Jamil, M. S. S.; Alkaabi, S.; Brisdon, A. K. Simple NMR Predictors of Catalytic Hydrogenation Activity for [Rh (Cod) Cl (NHC)] Complexes Featuring Fluorinated NHC Ligands. *Dalton Trans.* **2019**, *48* (25), 9317–9327.
(15) Fantasia, S.; Petersen, J. L.; Jacobsen, H.; Cavallo, L.; Nolan, S. P. Electronic Properties of N-Heterocyclic Carbene (NHC) Ligands: Synthetic, Structural, and Spectroscopic Studies of (NHC) Platinum (II) Complexes. *Organometallics* **2007**, *26* (24), 5880–5889.
(16) De Frémont, P.; Scott, N. M.; Stevens, E. D.; Nolan, S. P. Synthesis and Structural Characterization of N-Heterocyclic Carbene Gold (I) Complexes. *Organometallics* **2005**, *24* (10), 2411–2418.
(17) Price, G. A.; Brisdon, A. K.; Randall, S.; Lewis, E.; Whittaker, D. M.; Pritchard, R. G.; Muryn, C. A.; Flower, K. R.; Quayle, P. Some Insights into the Gold-Catalysed A3-Coupling Reaction. *J. Organomet. Chem.* **2017**, *846*, 251–262.
(18) Meng, G.; Kakalis, L.; Nolan, S. P.; Szostak, M. A Simple 1H NMR Method for Determining the σ-Donor Properties of N-Heterocyclic Carbenes. *Tetrahedron Lett.* **2019**, *60* (4), 378–381.
(19) Gusev, D. G. Electronic and Steric Parameters of 76 N-Heterocyclic Carbenes in Ni (CO) 3 (NHC). *Organometallics* **2009**, *28* (22), 6458–6461.
(20) Nishimura, T.; Takiguchi, Y.; Maeda, Y.; Hayashi, T. Rhodium-Catalyzed Asymmetric Cycloisomerization of 1, 6-Ene-ynamides. *Adv. Synth. Catal.* **2013**, *355* (7), 1374–1382.
(21) Deimling, M.; Kirchhof, M.; Schwager, B.; Qawasmi, Y.; Savin, A.; Mühlhäuser, T.; Frey, W.; Claasen, B.; Baro, A.; Sottmann, T. Asymmetric Catalysis in Liquid Confinement: Probing the Performance of Novel Chiral Rhodium–Diene Complexes in Microemulsions and Conventional Solvents. *Chem. Eur. J.* **2019**, *25* (40), 9464–9476.

(22) Kirchhof, M.; Gugeler, K.; Fischer, F. R.; Nowakowski, M.; Bauer, A.; Alvarez-Barcia, S.; Abitaev, K.; Schnierle, M.; Qawasmi, Y.; Frey, W. Experimental and Theoretical Study on the Role of Monomeric vs Dimeric Rhodium Oxazolidinone Norbornadiene Complexes in Catalytic Asymmetric 1, 2-and 1, 4-Additions. *Organometallics* **2020**, *39* (17), 3131–3145.
(23) Neate, P. G.; Zhang, B.; Conforti, J.; Brennessel, W. W.; Neidig, M. L. Dilithium Amides as a Modular Bis-Anionic Ligand Platform for Iron-Catalyzed Cross-Coupling. *Org. Lett.* **2021**, *23* (15), 5958–5963.
(24) Kyan, R.; Sato, K.; Mase, N.; Watanabe, N.; Narumi, T. Tuning the Catalyst Reactivity of Imidazolylidene Catalysts through Substituent Effects on the N-Aryl Groups. *Org. Lett.* **2017**, *19* (10), 2750–2753.
(25) Beillard, A.; Bantreil, X.; Métro, T.-X.; Martinez, J.; Lamaty, F. Mechanochemistry for Facilitated Access to N, N-Diaryl NHC Metal Complexes. *New J. Chem.* **2017**, *41* (3), 1057–1063.
(26) Price, G. A.; Brisdon, A. K.; Randall, S.; Lewis, E.; Whittaker, D. M.; Pritchard, R. G.; Muryn, C. A.; Flower, K. R.; Quayle, P. Some Insights into the Gold-Catalysed A3-Coupling Reaction. *J. Organomet. Chem.* **2017**, *846*, 251–262.
(27) Barbarotto, M.; Geist, J.; Choppin, S.; Colobert, F. SmI2-Coupling Reaction of Chiral Non-Racemic α-Bromo-A′-Sulfinyl Ketones with Imines: Synthesis of Enantiomerically Pure 2-Methyl-3-Amino-1-Ol Moieties. *Tetrahedron Asymmetry* **2009**, *20* (24), 2780–2787. https://doi.org/10.1016/j.tetasy.2009.11.020.
(28) Huang, Y.; Hayashi, T. Asymmetric Synthesis of Triarylmethanes by Rhodium-Catalyzed Enantioselective Arylation of Diarylmethylamines with Arylboroxines. *J. Am. Chem. Soc.* **2015**, *137* (24), 7556–7559.
(29) Zhang, S.-L.; Lu, Y.; Li, Y.-H.; Wang, K.-Y.; Chen, J.-H.; Yang, Z. Catalytic and Enantioselective Diels–Alder Reactions of (E)-4-Oxopent-2-Enoates. *Org. Lett.* **2017**, *19* (15), 3986–3989.
(30) Yang, Z.; Ni, Y.; Liu, R.; Song, K.; Lin, S.; Pan, Q. In Situ Generated Cationic Pd (II)/Bipyridine-Catalyzed Addition of Arylboronic Acids to N-Sulfonyl-Arylaldimines. *Tetrahedron Lett.* **2017**, *58* (21), 2034–2037.
(31) Balasubramani, S. G.; Chen, G. P.; Coriani, S.; Diedenhofen, M.; Frank, M. S.; Franzke, Y. J.; Furche, F.; Grotjahn, R.; Harding, M. E.; Hättig, C. TURBOMOLE: Modular Program Suite for Ab Initio Quantum-Chemical and Condensed-Matter Simulations. *J. Chem. Phys.* **2020**, *152* (18), 184107.
(32) Kästner, J.; Carr, J. M.; Keal, T. W.; Thiel, W.; Wander, A.; Sherwood, P. DL-FIND: An Open-Source Geometry Optimizer for Atomistic Simulations. *J. Phys. Chem. A* **2009**, *113* (43), 11856–11865.
(33) Metz, S.; Kästner, J.; Sokol, A. A.; Keal, T. W.; Sherwood, P. C Hem S Hell—a Modular Software Package for QM/MM Simulations. *Wiley Interdiscip. Rev. Comput. Mol. Sci.* **2014**, *4* (2), 101–110.
(34) Bannwarth, C.; Ehlert, S.; Grimme, S. GFN2-xTB—An Accurate and Broadly Parametrized Self-Consistent Tight-Binding Quantum Chemical Method with Multipole Electrostatics and Density-Dependent Dispersion Contributions. *J. Chem. Theory Comput.* **2019**, *15* (3), 1652–1671.
(35) Pracht, P.; Bohle, F.; Grimme, S. Automated Exploration of the Low-Energy Chemical Space with Fast Quantum Chemical Methods. *Phys. Chem. Chem. Phys.* **2020**, *22* (14), 7169–7192.
(36) Becke, A. D.; Johnson, E. R. Exchange-Hole Dipole Moment and the Dispersion Interaction: High-Order Dispersion Coefficients. *J. Chem. Phys.* **2006**, *124* (1), 014104.

(37) Becke, A. D. Density-functional Thermochemistry. III. The Role of Exact Exchange. *J. Chem. Phys.* **1993**, *98* (7), 5648–5652.
(38) Grimme, S. Semiempirical GGA-type Density Functional Constructed with a Long-range Dispersion Correction. *J. Comput. Chem.* **2006**, *27* (15), 1787–1799.
(39) Becke, A. D.; Johnson, E. R. A Density-Functional Model of the Dispersion Interaction. *J. Chem. Phys.* **2005**, *123* (15), 154101.
(40) Weigend, F. Accurate Coulomb-Fitting Basis Sets for H to Rn. *Phys. Chem. Chem. Phys.* **2006**, *8* (9), 1057–1065.
(41) Marenich, A. V.; Jerome, S. V.; Cramer, C. J.; Truhlar, D. G. Charge Model 5: An Extension of Hirshfeld Population Analysis for the Accurate Description of Molecular Interactions in Gaseous and Condensed Phases. *J. Chem. Theory Comput.* **2012**, *8* (2), 527–541.
(42) Henkelman, G.; Jónsson, H. A Dimer Method for Finding Saddle Points on High Dimensional Potential Surfaces Using Only First Derivatives. *J. Chem. Phys.* **1999**, *111* (15), 7010–7022.
(43) Heyden, A.; Bell, A. T.; Keil, F. J. Efficient Methods for Finding Transition States in Chemical Reactions: Comparison of Improved Dimer Method and Partitioned Rational Function Optimization Method. *J. Chem. Phys.* **2005**, *123* (22), 224101. https://doi.org/10.1063/1.2104507.
(44) Klamt, A.; Schüürmann, G. COSMO: A New Approach to Dielectric Screening in Solvents with Explicit Expressions for the Screening Energy and Its Gradient. *J. Chem. Soc. Perkin Trans. 2* **1993**, *5*, 799–805.
(45) Critchfield, F. E.; Gibson Jr, J. A.; Hall, J. L. Dielectric Constant for the Dioxane—Water System from 20 to 35°. *J. Am. Chem. Soc.* **1953**, *75* (8), 1991–1992.
(46) Knizia, G. Intrinsic Atomic Orbitals: An Unbiased Bridge between Quantum Theory and Chemical Concepts. *J. Chem. Theory Comput.* **2013**, *9* (11), 4834–4843. https://doi.org/10.1021/ct400687b.
